# 2013 Annual Report of the American Association of Poison Control Centers’ National Poison Data System (NPDS): 31st Annual Report

**DOI:** 10.3109/15563650.2014.987397

**Published:** 2015-01-06

**Authors:** James B. Mowry, Daniel A. Spyker, Louis R. Cantilena, Naya McMillan, Marsha Ford

## Abstract

***Background:*** This is the 31st Annual Report of the American Association of Poison Control Centers’ (AAPCC) National Poison Data System (NPDS). As of January 1, 2013, 57 of the nation's poison centers (PCs) uploaded case data automatically to NPDS. The upload interval was 8.08 [7.10, 11.63] (median [25%, 75%]) minutes, creating a near real-time national exposure and information database and surveillance system.

***Methodology:*** We analyzed the case data tabulating specific indices from NPDS. The methodology was similar to that of previous years. Where changes were introduced, the differences are identified. Poison center (PC) cases with medical outcomes of death were evaluated by a team of 38 medical and clinical toxicologist reviewers using an ordinal scale of 1–6 to assess the Relative Contribution to Fatality (RCF) of the exposure to the death.

***Results:*** In 2013, 3,060,122 closed encounters were logged by NPDS: 2,188,013 human exposures, 59,496 animal exposures, 806,347 information calls, 6,116 human-confirmed nonexposures, and 150 animal-confirmed nonexposures. Total encounters showed a 9.3% decline from 2012, while health care facility human exposure calls were essentially flat, decreasing by 0.1%.All information calls decreased 21.4% and health care facility (HCF) information calls decreased 8.5%, medication identification requests (drug ID) decreased 26.8%, and human exposures reported to US PCs decreased 3.8%. Human exposures with less serious outcomes have decreased 3.7% per year since 2008 while those with more serious outcomes (moderate, major or death) have increased by 4.7% per year since 2000.

The top five substance classes most frequently involved in all human exposures were analgesics (11.5%), cosmetics/personal care products (7.7%), household cleaning substances (7.6%), sedatives/hypnotics/antipsychotics (5.9%), and antidepressants (4.2%). Sedative/hypnotics/antipsychotics exposures as a class increased most rapidly (2,559 calls/year) over the last 13 years for cases showing more serious outcomes. The top five most common exposures in children of 5 years or less were cosmetics/personal care products (13.8%), household cleaning substances (10.4%), analgesics (9.8%), foreign bodies/toys/miscellaneous (6.9%), and topical preparations (6.1%). Drug identification requests comprised 50.7% of all information calls. NPDS documented 2,477 human exposures resulting in death with 2,113 human fatalities judged related (RCF of 1, undoubtedly responsible; 2, probably responsible; or 3, contributory).

***Conclusions:*** These data support the continued value of PC expertise and need for specialized medical toxicology information to manage the more severe exposures, despite a decrease in calls involving less severe exposures. Unintentional and intentional exposures continue to be a significant cause of morbidity and mortality in the United States. The near real-time, always current status of NPDS represents a national public health resource to collect and monitor US exposure cases and information calls. The continuing mission of NPDS is to provide a nationwide infrastructure for public health surveillance for all types of exposures, public health event identification, resilience response and situational awareness tracking. NPDS is a model system for the nation and global public health.

**WARNING:** Comparison of exposure or outcome data from previous AAPCC Annual Reports is problematic. In particular, the identification of fatalities (attribution of a death to the exposure) differed from pre-2006 Annual Reports (see Fatality Case Review—Methods). Poison center death cases are described as all cases resulting in death and those determined to be exposure-related fatalities. Likewise, Table 22 (Exposure Cases by Generic Category) since year 2006 restricts the breakdown including deaths to single-substance cases to improve precision and avoid misinterpretation.

## Introduction

This is the 31st Annual Report of the American Association of Poison Control Centers’ (AAPCC; http://www.aapcc.org) National Poison Data System (NPDS).(1) On 1 January 2013, fifty-seven regional poison centers (PCs) serving the entire population of the 50 United States, American Samoa, District of Columbia, Federated States of Micronesia, Guam, Puerto Rico, and the US Virgin Islands submitted information and exposure case data collected during the course of providing telephonic patient-tailored exposure management and poison information.

NPDS is the data warehouse for the nation's 57 PCs. PCs place emphasis on exposure management, accurate data collection and coding, and responding to the continuing need for poison related public and professional education. The PC's health care professionals are available free of charge to users, 24-hours a day, every day of the year. PCs respond to questions from the public, health care professionals, and public health agencies. The continuous staff dedication at the PCs is manifest as the number of exposure, and information call encounters exceeds 3.0 million annually. PC encounters involve either an exposed human or animal (exposure call) or a request for information with no person or animal exposed to any foreign body, viral, bacterial, venomous, or chemical agent or commercial product (information call).

### The NPDS Products Database

The NPDS products database contains over 400,000 products ranging from viral and bacterial agents to commercial chemical and drug products. The product database is maintained and continuously updated by data analysts at the Micromedex Poisindex^®^System (Micromedex Healthcare Series [Internet database]; Greenwood Village, CO: Truven Health Analytics). A robust generic coding system categorizes the products data into 1,081 generic codes. These generic codes collapse into Nonpharmaceutical (562) and Pharmaceutical (519) groups. These two groups are divided into Major (68) and Minor (172) categories. The generic coding schema undergoes continuous improvement through the work of the AAPCC—Micromedex Joint Coding Group. The group consists of AAPCC members and editorial and lexicon staff working to meet best terminology practices. The generic code system provides enhanced report granularity as reflected in Table 22. The following 30 generic codes were introduced in 2013:

**Table:** Generic Codes Added in 2013.

1	Baclofen
2	Bacterial Diseases
3	Bupropion
4	Citalopram
5	Clomipramine
6	Duloxetine
7	Escitalopram
8	Fluoxetine
9	Fluvoxamine
10	Food Additives
11	Food Products
12	Fungal Diseases
13	Isocarboxazid
14	Loxapine
15	Metaxalone
16	Mirtazapine
17	Nefazodone
18	Other Types of Serotonin Norepinephrine Reuptake Inhibitor (SNRI)
19	Oxygen Absorbers
20	Parasitic Diseases
21	Paroxetine
22	Phenelzine
23	Prion Diseases
24	Selegiline
25	Sertraline
26	Tizanidine
27	Tranylcypromine
28	Trimipramine
29	Venlafaxine
30	Viral Diseases

Because the new codes were added at different times during the year, the numbers in Table 22 for these generic codes do not reflect the entire year. For completeness, certain codes of these categories require customized data retrieval until these categories have been in place for a year or more.

## Methods

### Characterization of Participating PCs and Population Served

Fifty-seven participating centers submitted data to AAPCC through 30 September, 2013, when one participating center closed with its calls picked up by another PC in its state, leaving 56 participating centers as of 31 December 2013.Fifty-four centers (95%) were accredited by AAPCC as of 1 July 2013. The entire population of the 50 states, American Samoa, the District of Columbia, Federated States of Micronesia, Guam, Puerto Rico, and the US Virgin Islands was served by the US PC network in 2013.(2,3,4,5).

The average number of human exposure cases managed per day by all US PCs was 5,995. Similar to other years, higher volumes were observed in the warmer months, with a mean of 6,365 cases per day in July compared with 5,424 per day in December. On average, US PCs received a call about an actual human exposure every 14.4 seconds.

### Call Management—Specialized Poison Exposure Emergency Providers

Most PC operation management, clinical education, and instruction are directed by managing directors (most are PharmDs and RNs with American Board of Applied Toxicology [ABAT] board certification). Medical direction is provided by medical directors who are board-certified physician medical toxicologists. At some PCs, the managing and medical director positions are held by the same person.

Calls received at US PCs are managed by health care professionals who have received specialized training in toxicology and managing exposure emergencies. These providers include medical and clinical toxicologists, registered nurses, doctors of pharmacy, pharmacists, chemists, hazardous materials specialists, and epidemiologists. Specialists in Poison Information (SPIs) are primarily registered nurses, PharmDs, and pharmacists who direct the public to the most appropriate level of care while also providing the most up-to-date management recommendations to health care providers caring for exposed patients. They may work under the supervision of a Certified Specialist in Poison Information (CSPI). SPIs must log a minimum of 2,000 calls over a 12-month period to become eligible to take the CSPI examination for certification in poison information. Poison information providers (PIPs) are allied health care professionals. They manage information-type and low acuity (non-hospital) calls and work under the supervision of a CSPI. Of note is the fact that no nursing or pharmacy school offers a toxicology curriculum designed for PC work and SPIs must be trained in programs offered by their respective PC. PCs undergo a rigorous accreditation process administered by the AAPCC and must be reaccredited every 5 years.

### NPDS—Near Real-time Data Capture

Launched on 12 April 2006, NPDS is the data repository for all of the US PCs. In 2013, all 57 US PCs uploaded case data automatically to NPDS. All PCs submitted data in near real-time, making NPDS one of the few operational systems of its kind. PC staff record calls contemporaneously in 1 of 4 case data management systems. Each PC uploads case data automatically. The time to upload data for all PCs is 8.08 [7.10, 11.63] (median [25%, 75%]) minutes creating a near real-time national exposure database and surveillance system.

The web-based NPDS software facilitates detection, analysis, and reporting of NPDS surveillance anomalies. System software offers a myriad of surveillance uses allowing AAPCC, its member centers, and public health agencies to utilize NPDS US exposure data. Users are able to access local and regional data for their own areas and view national aggregate data. Custom surveillance definitions are available along with ad hoc reporting tools. Information in the NPDS database is dynamic. Each year the database is locked prior to extraction of annual report data to prevent inadvertent changes and ensure consistent, reproducible reports. The 2013 database was locked on 27 October 2014 at 17:00 EDT.

### Annual Report Case Inclusion Criteria

The information in this report reflects only those cases that are not duplicates and classified by the PC as CLOSED. A case is closed when the PC has determined that no further follow-up/recommendations are required or no further information is available. Exposure cases are followed to obtain the most precise medical outcome possible. Depending on the case specifics, most calls are “closed” within a few hours of the initial call. Some calls regarding complex hospitalized patients or cases resulting in death may remain open for weeks or months while data continue to be collected. Follow-up calls provide a proven mechanism for monitoring the appropriateness of management recommendations, augmenting patient guidelines and providing poison prevention education, enabling continual updates of case information as well as obtaining final/known medical outcome status to make the data collected as accurate and complete as possible.

### Statistical Methods

All tables except Tables 3B and 17B were generated directly by the NPDS web-based application and can thus be reproduced by each center. The figures and statistics in Tables 3B and 17B were created using SAS JMP version 9.0.0 (SAS Institute, Cary, NC) on summary counts generated by the NPDS web-based application.

### NPDS Surveillance

As previously noted, all of the active US PCs upload case data automatically to NPDS. This unique near real-time upload is the foundation of the NPDS surveillance system. This makes possible both spatial and temporal case volume and case based surveillance. NPDS software allows creation of volume and case-based definitions. Definitions can be applied to national, regional, state, or ZIP code coverage areas. Geocentric definitions can also be created. This functionality is available not only to the AAPCC surveillance team, but to every PC. PCs also have the ability to share NPDS real-time surveillance technology with external organizations such as their state and local health departments or other regulatory agencies. Another NPDS feature is the ability to generate system alerts on adverse drug events and other drug or commercial products of public health interest like contaminated food or product recalls. Thus, NPDS can provide real-time adverse event monitoring and surveillance of resilience response and situational awareness.

Surveillance definitions can be created to monitor a variety of volume parameters or case-based definitions on any desired substance or commercial product in the Micromedex Poisindex products database and/or set of clinical effects or other parameters. The products database contains over 400,000 entries. Surveillance definitions may be constructed using volume or case-based definitions with a variety of mathematical options and historical baseline periods from 1 to 13 years. NPDS surveillance tools include the following:

- Volume Alert Surveillance Definitions
- Total Call Volume
- Human Exposure Call Volume
- Animal Exposure Call Volume
- Information Call Volume
- Clinical Effects Volume (signs and symptoms, or laboratory abnormalities)
- Case-Based Surveillance Definitions utilizing various NPDS data fields linked in Boolean expressions
  - Substance
  - Clinical Effects
  - Species
  - Medical Outcome and Others
- Syndromic Surveillance Definitions allow Boolean-based definitions utilizing various NPDS data fields to be run based on historical trends for user-defined periods of interest.

Incoming data are monitored continuously and anomalous signals generate an automated email alert to the AAPCC's surveillance team or designated PC or public health agency staff. These anomaly alerts are reviewed daily by the AAPCC surveillance team, the PC, or the public health agency that created the surveillance definition. When reports of potential public health significance are detected, additional information is obtained via the NPDS surveillance correspondence system or phone as appropriate from reporting PCs. The PC then alerts their respective state or local health departments. Public health issues are brought to the attention of the Health Studies Branch, National Center for Environmental Health, Centers for Disease Control and Prevention (HSB/NCEH/CDC). This unique near real-time tracking ability is a unique feature offered by NPDS and the PCs.

Clinical and medical toxicologists of the AAPCC surveillance team review surveillance definitions on a regular basis to fine-tune the queries. CDC, as well as State and local health departments with NPDS access as granted by their respective PCs, also have the ability to create surveillance definitions for routine surveillance tasks or to respond to emerging public health events.

### Fatality Case Review and Abstract Selection

NPDS fatality cases can be recorded as DEATH or DEATH (INDIRECT REPORT). Medical outcome of death is given by direct report. Deaths (indirect reports) are deaths that the PC acquired from medical examiners or media, but did not manage nor answer any questions related specifically to that death.

Although PCs may report death as an outcome, the death may not be the direct result of the exposure. We define exposure-related fatality as a death judged by the AAPCC Fatality Review Team to be at least contributory to the exposure. The definitions used for the Relative Contribution to Fatality (RCF) classification are given in Appendix B and the methods for selecting abstracts for publications are described in Appendix C. For details on the AAPCC fatality review process, see the 2008 annual report.(1)

### Pediatric Fatality Case Review

A focused Pediatric Fatality Review team, comprised of 4 pediatric toxicologists, evaluated cases of patients of 19 years and under. The panel reviewed the documentation of all such cases, with specific focus on the conditions behind the poisoning exposure and on finding commonality which might inform efforts at prevention. The pediatric fatality cases reviewed exhibited a bimodal age distribution. Exposures causing death in children ≤ 5 years of age were mostly coded as “Unintentional-General”, while those in ages over 12 years were mostly as “Intentional”. Often the Reason Code did not capture the complexities of the case. For example, there were few mentions of details such as the involvement of law enforcement or child protective services. While there were some complete and informative reports, in many narratives the circumstances which preceded the exposure thought responsible for the death were unclear or absent. In response to these findings, the pediatric fatality review team developed and distributed Pediatric Narrative Guidelines, with specific attention to the root cause of these cases. PCs are requested to heed these guidelines and the need for a more in-depth investigation of “causality.”

## Results

### Information Calls to Poison Centers

Data from 806,347 information calls to PCs in 2013 (Table 1C) was transmitted to NPDS, including calls in optional reporting categories such as prevention/safety/education (24,249), administrative (25,878), and caller referral (47,682).

Figure 2 shows that all drug ID calls decreased dramatically in mid-2009, again in late 2010 and late 2011, and continue to decrease in 2012 and 2013. Law enforcement drug ID calls also showed a decline. The most frequent information call was for drug ID, comprising 408,711calls to PCs during the year. Of these, 239,364 (58.6%) were identified as drugs with known abuse potential; however, these cases were categorized based on the drug's abuse potential without any knowledge of whether abuse was actually intended.

While the number of drug information calls decreased 21.4% from 2012 (144,267 calls) to 2013 (113,378 calls), the distribution of these call types remained steady at 14.1% of all information request calls. The most common drug information requests were about drug–drug interactions, followed by other drug information, therapeutic use and indications, questions about dosage, and inquiries of adverse effects. Environmental inquiries comprised 2.3% of all information calls. Of these environmental inquiries, specific questions related to cleanup of mercury (thermometers and other) remained the most common followed by questions involving pesticides.

Of all the information calls, poison information comprised 7.0% of the requests with inquiries involving general toxicity the most common followed by questions involving food preparation practices, safe use of household products, and plant toxicity.

### Exposure Calls to Poison Centers

In 2013, the participating PCs logged 3,060,122 total encounters including 2,188,013 closed human exposure cases (Table 1A), 59,496 animal exposures (Table 1B), 806,347 information calls (Table 1C), 6,116 human confirmed non-exposures, and 150 animal confirmed non-exposures. An additional 570 calls were still open at the time of database lock. The cumulative AAPCC database now contains more than 60 million human exposure case records (Table 1A). A total of 16,392,826 information calls have been logged by NPDS since the year 2000.

**Table 1A.** AAPCC Population Served and Reported Exposures (1983–2013).

**Year**	**No. of participating centers**	**Population served (in millions)**	**Human exposures**	**Exposures per thousand population**
1983	16	43.1	251,012	5.8
1984	47	99.8	730,224	7.3
1985	56	113.6	900,513	7.9
1986	57	132.1	1,098,894	8.3
1987	63	137.5	1,166,940	8.5
1988	64	155.7	1,368,748	8.8
1989	70	182.4	1,581,540	8.7
1990	72	191.7	1,713,462	8.9
1991	73	200.7	1,837,939	9.2
1992	68	196.7	1,864,188	9.5
1993	64	181.3	1,751,476	9.7
1994	65	215.9	1,926,438	8.9
1995	67	218.5	2,023,089	9.3
1996	67	232.3	2,155,952	9.3
1997	66	250.1	2,192,088	8.8
1998	65	257.5	2,241,082	8.7
1999	64	260.9	2,201,156	8.4
2000	63	270.6	2,168,248	8.0
2001	64	281.3	2,267,979	8.1
2002	64	291.6	2,380,028	8.2
2003	64	294.7	2,395,582	8.1
2004	62	293.7	2,438,643	8.3
2005	61	296.4	2,424,180	8.2
2006	61	299.4	2,403,539	8.0
2007	61	305.6	2,482,041	8.1
2008	61	308.5 ^b^	2,491,049	8.1
2009	60	310.9 ^b^	2,479,355	8.0
2010	60^a^	313.3^b^	2,384,825	7.6
2011	57^c^	315.7^b^	2,334,004	7.4
2012	57	318.0^b^	2,275,141	7.2
2013	57^d^	320.2^e^	2,188,013	6.8
**Total**			**60,117,368**	

^a^As of 1 July 2010 there were 60 participating centers.

^b^AAPCC total as of 1 July mid-year US Census (2012 data for 50 United States, District of Columbia and Puerto Rico; 2011 data for Guam; 2010 data for American Samoa, Federated States of Micronesia, and the US Virgin Islands)

^c^As of 1 July 2011 there were 57 participating centers.

^d^One participating center closed in September 2013. Its data are included in the 2013 totals.

^e^AAPCC Total as of 1 July mid-year US Census (2013 data for 50 United States, District of Columbia and Puerto Rico, Guam, American Samoa, Federated States of Micronesia, and the US Virgin Islands) (2,3).

**Table 1B.** Non-Human Exposures by Animal Type.

**Animal**	**N**	**%**
Dog	53,760	90.36
Cat	5,015	8.43
Bird	163	0.27
Rodent/lagomorph	141	0.24
Horse	111	0.19
Sheep/goat	39	0.07
Cow	30	0.05
Aquatic	17	0.03
Other	220	0.37
**Total**	**59,496**	**100.00**

**Table 1C.** Distribution of Information Calls.

**Information call type**	**N**	**% of Information. calls**
**Drug identification**		
Public inquiry: Drug sometimes involved in abuse	188,220	23.34
Public inquiry: Drug not known to be abused	84,857	10.52
Public inquiry: Unknown abuse potential	2,777	0.34
Public inquiry: Unable to identify	39,795	4.94
HCP inquiry: Drug sometimes involved in abuse	2,266	0.28
HCP inquiry: Drug not known to be abused	4,362	0.54
HCP inquiry: Unknown abuse potential	142	0.02
HCP inquiry: Unable to identify	1,710	0.21
Law Enf. Inquiry: Drug sometimes involved in abuse	48,878	6.06
Law Enf. Inquiry: Drug not known to be abused	26,818	3.33
Law Enf. Inquiry: Unknown abuse potential	875	0.11
Law Enf. Inquiry: Unable to identify	6,349	0.79
Other drug ID	1,662	0.21
**Subtotal**	**408,711**	**50.69**
**Drug information**		
Adverse effects (no known exposure)	8,566	1.06
Brand/generic name clarifications	2,205	0.27
Calculations	134	0.02
Compatibility of parenteral medications	238	0.03
Compounding	286	0.04
Contraindications	1,373	0.17
Dietary supplement, herbal, and homeopathic	536	0.07
Dosage	11,093	1.38
Dosage form / formulation	1,800	0.22
Drug use during breast-feeding	2,097	0.26
Drug–drug interactions	22,519	2.79
Drug–food interactions	1,601	0.20
Foreign drug	300	0.04
Generic substitution	451	0.06
Indications/therapeutic use	20,234	2.51
Medication administration	5,144	0.64
Medication availability	571	0.07
Medication disposal	3,057	0.38
Pharmacokinetics	1,700	0.21
Pharmacology	1,090	0.14
Regulatory	3,229	0.40
Stability / storage	2,424	0.30
Therapeutic drug monitoring	533	0.07
Other drug info	22,197	2.75
**Subtotal**	**113,378**	**14.06**
**Environmental information**		
Air quality	1,445	0.18
Carbon monoxide—no known patient(s)	595	0.07
Carbon monoxide alarm use	377	0.05
Chem / bioterrorism / weapons (suspected or confirmed)	16	0.00
Clarification of media reports of environmental contamination	25	0.00
Clarification of substances involved in a HAZMAT incident—no known victim(s)	115	0.01
General questions about contamination of air and / or soil	347	0.04
HAZMAT planning	122	0.02
Lead—no known patient(s)	383	0.05
Mercury thermometer cleanup	1,541	0.19
Mercury (excluding thermometers) cleanup	3,184	0.39
Notification of a HAZMAT incident—no known patient(s)	596	0.07
Pesticide application by a professional pest control operator	639	0.08
Pesticides (other)	2,395	0.30
Potential toxicity of chemicals in the environment	1,148	0.14
Radiation	51	0.01
Safe disposal of chemicals	1,240	0.15
Water purity/contamination	600	0.07
Other environmental	3,963	0.49
**Subtotal**	**18,782**	**2.33**
**Medical information**		
Dental questions	114	0.01
Diagnostic or treatment recommendations for diseases or conditions—non-toxicology	7,401	0.92
Disease prevention	484	0.06
Explanation of disease states	845	0.10
General first-aid	1,051	0.13
Interpretation of non-toxicology laboratory reports	118	0.01
Medical terminology questions	62	0.01
Rabies—no known patient(s)	261	0.03
Sunburn management	51	0.01
Other medical	48,516	6.02
**Subtotal**	**58,903**	**7.30**
**Occupational information**		
Occupational treatment / first-aid guidelines—no known patient(s)	36	0.00
Information on chemicals in the workplace	104	0.01
MSDS interpretation	64	0.01
Occupational MSDS requests	724	0.09
Routine toxicity monitoring	32	0.00
Safe handling of workplace chemicals	90	0.01
Other occupational	206	0.03
**Subtotal**	**1,256**	**0.16**
**Poison information**		
Analytical toxicology	751	0.09
Carcinogenicity	65	0.01
Food poisoning—no known patient(s)	2,043	0.25
Food preparation/handling practices	6,154	0.76
General toxicity	23,212	2.88
Mutagenicity	41	0.01
Plant toxicity	2,431	0.30
Recalls of non-drug products (including food)	250	0.03
Safe use of household products	3,756	0.47
Toxicology information for legal use / litigation	154	0.02
Other poison	17,475	2.17
**Subtotal**	**56,332**	**6.99**
**Prevention/Safety/Education**		
Confirmation of poison center number	13,569	1.68
General (non-poison) injury prevention requests	519	0.06
Media requests	299	0.04
Poison prevention material requests	8,426	1.04
Poison prevention week date inquiries	34	0.00
Professional education presentation requests	263	0.03
Public education presentation requests	380	0.05
Other prevention	759	0.09
**Subtotal**	**24,249**	**3.01**
**Teratogenicity information**		
Teratogenicity	1,563	0.19
**Subtotal**	**1,563**	**0.19**
**Other information**		
Other	43,830	5.44
**Subtotal**	**43,830**	**5.44**
**Substance Abuse**		
Drug screen information	4,265	0.53
Effects of illicit substances—no known patient(s)	306	0.04
New trend information	281	0.03
Withdrawal from illicit substances—no known patient(s)	181	0.02
Other substance abuse	750	0.09
**Subtotal**	**5,783**	**0.72**
**Administrative**		
Expert witness requests	34	0.00
Faculty activities	60	0.01
Funding	20	0.00
Personnel issues	243	0.03
Poison center record request	143	0.02
Product replacement/malfunction (issues intended for the manufacturer)	2,534	0.31
Scheduling of poison center rotations	93	0.01
Other administration	22,751	2.82
**Subtotal**	**25,878**	**3.21**
**Caller Referred**		
Immediate referral—animal poison center or veterinarian	16,172	2.01
Immediate referral—drug identification	5,102	0.63
Immediate referral—drug information	200	0.02
Immediate referral—health department	7,232	0.90
Immediate referral—medical advice line	610	0.08
Immediate referral—pediatric triage service	238	0.03
Immediate referral—pesticide hotline	364	0.05
Immediate referral—pharmacy	682	0.08
Immediate referral—poison center	2,952	0.37
Immediate referral—private physician	2,609	0.32
Immediate referral—psychiatric crisis line	118	0.01
Immediate referral—teratology information program	102	0.01
Other call referral	11,301	1.40
**Subtotal**	**47,682**	**5.91**
**Total**	**806,347**	**100.00**

**Figure 1.**
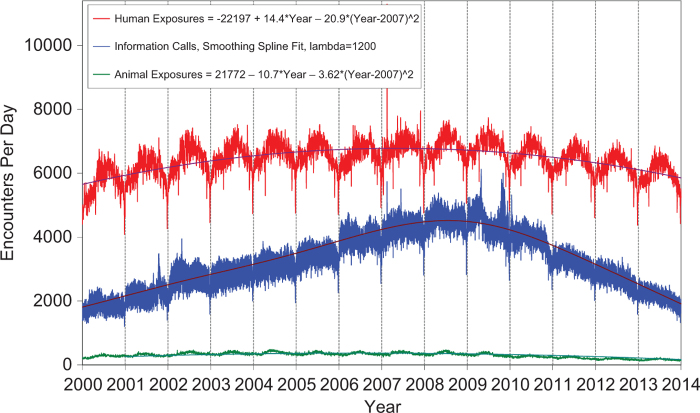
**Human Exposure Calls, Information Calls and Animal Exposure Calls by Day since January 1, 2000.** Both linear and second-order (quadratic) terms were statistically significant for least-squares second-order regressions of Human Exposures and Animal Exposures. Smoothing spline fit for Information calls has lambda = 1200, R-square = 0.832 (colour version of this figure can be found in the online version at www.informahealthcare.com/ctx).

Figure 1 shows the human exposures, information calls and animal exposures by day since 1 January 2001. Second-order (quadratic) least squares regression of these data shows a statistically significant departure from linearity (declining rate of calls since mid-2007) for human exposure calls. Information calls are best described by a smoothing spline fit, and animal exposure calls have likewise been declining since mid-2005.

A hallmark of PC case management is the use of follow-up calls to monitor case progress and medical outcome. US PCs made 2,515,811 follow-up calls in 2013. Follow-up calls were made in 46.1% of human exposure cases. One follow-up call was made in 22.0% of human exposure cases, and multiple follow-up calls (range, 2–121) were placed in 24.1% of cases.

Figure 3 shows a graphic summary and analyses of Health Care Facility (HCF) exposure and HCF information calls. HCF exposure calls slightly departed from linearity but continued to increase at a steady rate, while the rate of HCF information calls has been declining since early 2005. This increasing use of the PCs for the more serious exposures (HCF calls) is important in the face of the decline in exposure and information calls. The 2 May 2006 exposure data spike on the figure was the result of 602 children in a Midwest school reporting a noxious odor which caused anxiety, but resolved without sequelae.

**Figure 2.**
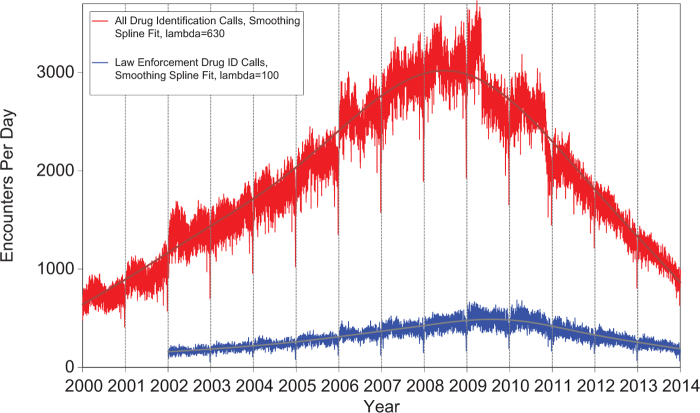
**All Drug Identification and Law Enforcement Drug Identification Calls by day since January 1, 2000.** Smoothing Spline Fits were better than second-order regressions, R-square = 0.933 for All Drug Identification Calls, R-square = 0.780 for Law Enforcement Drug ID Calls (colour version of this figure can be found in the online version at www.informahealthcare.com/ctx).

**Figure 3.**
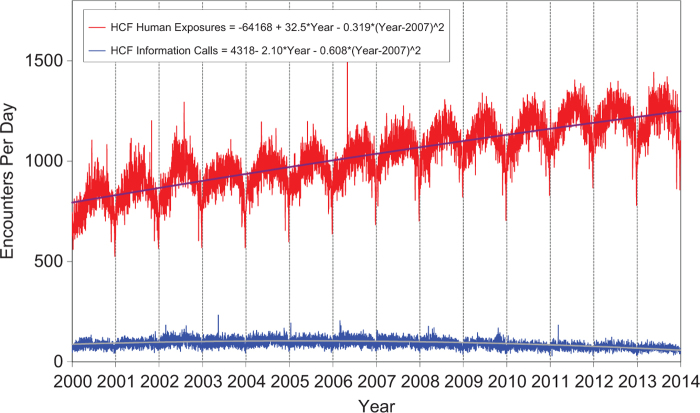
**Health Care Facility (HCF) Exposure Calls and HCF Information Calls by day since January 1, 2000.** Regression lines show least-squares second-order regressions for HCF Exposure and HCF Information Calls. All terms shown were statistically significant for each of the two regressions (colour version of this figure can be found in the online version at www.informahealthcare.com/ctx).

Tables 22A (Nonpharmaceuticals) and 22B (Pharmaceuticals) provide summary demographic data on patient age, reason for exposure, medical outcome, and use of a health care facility for all 2,188,013 human exposure cases, presented by substance categories. The Pharmaceuticals category includes both licit and illicit drugs.

Column 1: Name of the major, minor generic categories and their associated generic codes.

Column 2: Number of Case Mentions (All Exposures) in grey shading, displays the number of times the specific generic code was reported in all human exposure cases. If a human exposure case has multiple instances of a specific generic code, it is counted only once.

Column 3: Single Substance Exposures; this column was previously named “No. of Single Exposures” and was renamed in the 2009 report for clarity. This column displays the number of human exposure cases that identified only one substance (one case, one substance).

The succeeding columns (Age, Reason, Treatment Site, And Outcome) show selected detail from these single-substance exposure cases. Death cases include both cases that have the outcomes of Death or Death (indirect report).These death cases are not limited by the relative contribution to fatality.

Tables 22A and 22B restrict the breakdown columns to single-substance cases. Prior to 2007, when multisubstance exposures were included, a relatively innocuous substance could be mentioned in a death column when, for example, the death was attributed to an antidepressant, opioid, or cyanide. This subtlety was not always appreciated by the user of this table. The restriction of the breakdowns to single-substance exposures should increase precision and reduce misrepresentation of the results in this unique by-substance table. Single-substance cases reflect the majority (89.1%) of all exposures. In contrast, only 44.2% of fatalities are single substance exposures (Table 5).

Tables 22A and 22B tabulate 2,575,837 substance exposures, of which 1,950,455 were single-substance exposures, including1,013,229 (52.0%) nonpharmaceuticals and 937,226 (48.0%) pharmaceuticals. In 19.6% of single- substance exposures that involved pharmaceutical substances, the reason for exposure was intentional, compared with only 3.6% that involved a nonpharmaceutical substance. Correspondingly, treatment in a health care facility was provided in a higher percentage of exposures that involved pharmaceutical substances (29.8%) compared with that of nonpharmaceutical substances (15.9%). Exposures to pharmaceuticals also had more severe outcomes. Of single-substance exposure-related fatal cases, 708 (70.7%) were pharmaceuticals compared with 293 (29.3%) nonpharmaceutical.

### Age and Gender Distributions

The age and gender distribution of human exposures is outlined in Table 3. Children younger than 3 years were involved in 35.5% of exposures and children younger than 6 years accounted for approximately half of all human exposures (48.0%). Male predominance was found among cases involving children younger than 13 years, but this gender distribution was reversed in teenagers and adults, with females comprising the majority of reported exposures.

### Caller Site and Exposure Site

As shown in Table 2, of the 2,188,013 human exposures reported, 71.8% of calls originated from a residence (own or other) but 93.5% actually occurred at a residence (own or other). Another 20.3% of calls were made from a HCF. Beyond residences, exposures occurred in the workplace in 1.6% of cases, schools (1.3%), health care facilities (0.3%), and restaurants or food services (0.2%).

**Table 2.** Site of Call and Site of Exposure, Human Exposure Cases.

	**Site of caller**		**Site of exposure**	
**Site**	**N**	**%**	**N**	**%**
Residence				
Own	1,539,136	70.34	1,994,810	91.17
Other	32,014	1.46	50,310	2.30
Workplace	24,320	1.11	35,563	1.63
Health care facility	443,318	20.26	6,083	0.28
School	9,691	0.44	27,961	1.28
Restaurant/food service	436	0.02	4,766	0.22
Public area	6,707	0.31	20,482	0.94
Other	125,752	5.75	24,827	1.13
Unknown	6,639	0.30	23,211	1.06

**Table 3A.** Age and Gender Distribution of Human Exposures.

	**Male**		**Female**		**Unknown gender**		**Total**		**Cumulative total**	
**Age (y)**	**N**	**% of age group total**	**N**	**% of age group total**	**N**	**% of age group total**	**N**	**% of total exposures**	**N**	**%**
**Children (< 20)**										
< 1	58,132	51.83	53,698	47.88	329	0.29	112,159	5.13	112,159	5.13
1	172,707	52.03	158,747	47.82	508	0.15	331,962	15.17	444,121	20.30
2	174,346	52.40	157,886	47.45	498	0.15	332,730	15.21	776,851	35.50
3	81,745	54.55	67,776	45.23	319	0.21	149,840	6.85	926,691	42.35
4	42,045	55.87	33,019	43.88	190	0.25	75,254	3.44	1,001,945	45.79
5	25,725	56.58	19,585	43.07	159	0.35	45,469	2.08	1,047,414	47.87
Unknown ≤ 5	953	46.24	809	39.25	299	14.51	2,061	0.09	1,049,475	47.96
Child 6–12	78,140	57.82	55,802	41.29	1,203	0.89	135,145	6.18	1,184,620	54.14
Teen 13–19	63,767	41.64	88,527	57.81	843	0.55	153,137	7.00	1,337,757	61.14
Unknown Child	1,685	41.05	1,385	33.74	1,035	25.21	4,105	0.19	1,341,862	61.33
**Subtotal**	**699,245**	**52.11**	**637,234**	**47.49**	**5,383**	**0.40**	**1,341,862**	**61.33**	**1,341,862**	**61.33**
**Adults (≥ 20)**										
20–29	87,238	46.45	100,402	53.46	173	0.09	187,813	8.58	1,529,675	69.91
30–39	63,400	43.11	83,524	56.80	130	0.09	147,054	6.72	1,676,729	76.63
40–49	52,726	41.51	74,213	58.42	89	0.07	127,028	5.81	1,803,757	82.44
50–59	47,450	40.19	70,556	59.76	68	0.06	118,074	5.40	1,921,831	87.83
60–69	30,050	37.99	49,011	61.96	39	0.05	79,100	3.62	2,000,931	91.45
70–79	17,030	35.90	30,382	64.04	27	0.06	47,439	2.17	2,048,370	93.62
80–89	9,729	34.04	18,834	65.90	15	0.05	28,578	1.31	2,076,948	94.92
≥ 90	1,962	31.41	4,276	68.45	9	0.14	6,247	0.29	2,083,195	95.21
Unknown adult	35,855	38.88	53,889	58.43	2,486	2.70	92,230	4.22	2,175,425	99.42
**Subtotal**	**345,440**	**41.44**	**485,087**	**58.19**	**3,036**	**0.36**	**833,563**	**38.10**	**2,175,425**	**99.42**
**Other**										
Unknown age	4,385	34.83	5,656	44.93	2,547	20.23	12,588	0.58	2,188,013	100.00
**Total**	**1,049,070**	**47.95**	**1,127,977**	**51.55**	**10,966**	**0.50**	**2,188,013**	**100.00**	**2,188,013**	**100.00**

**Table 3B.** Population-Adjusted Exposures by Age Group.

**Age Group**	**Exposures/100k population**	**Number of Exposures^a^**	**Population^b^**
**Children (< 20)**			
< 1	2,813	112,159	3,987,406
1	8,294	331,962	4,002,216
2	8,245	332,730	4,035,525
3	3,711	149,840	4,037,916
4	1,863	75,254	4,040,463
5	1,090	45,469	4,170,700
Child 6–12	464	135,145	29,101,221
Teen 13–19	511	153,137	29,940,491
**Subgroup**	**1,603**	**1,335,696**	**83,315,938**
**Adults (≥ 20)**			
20–29	418	187,813	44,929,989
30–39	355	147,054	41,386,428
40–49	298	127,028	42,583,166
50–59	267	118,074	44,263,274
60–69	238	79,100	33,169,197
70–79	256	47,439	18,561,592
80–89	295	28,578	9,695,645
90+	276	6,247	2,264,634
**Subgroup**	**313**	**741,333**	**236,853,925**
**Overall Total**	**683**	**2,188,013**	**320,169,863**

^a^Number of exposures excludes UNKNOWN ages from the individual age categories, but includes them in the Subtotals and overall total (see Table 3A)

^b^AAPCC Total as of 1 July 2013 320,169,863 (see Table 1A).(3,4,5)

### Exposures in Pregnancy

Exposure during pregnancy occurred in 7,384 women (0.3% of all human exposures). Of those with known pregnancy duration (*n* = 6,830), 31.5% occurred in the first trimester, 37.0% in the second trimester, and 31.5% in the third trimester. Most (73.9%) were unintentional exposures and 19.6% were intentional exposures. There was one death of a pregnant woman in 2013.

### Chronicity

Most human exposures, 1,922,316 (87.9%), were acute cases (single, repeated, or continuous exposure occurring over 8 hours or less) compared with 1,328 acute cases of 2,477 fatalities (53.6%). Chronic exposures (continuous or repeated exposures occurring over > 8 hours) comprised 2.1% (46,900) of all human exposures. Acute-on-chronic exposures (single exposure that was preceded by a continuous, repeated, or intermittent exposure occurring over a period of > 8 hours) numbered 188,899 (8.6%).

### Reason for Exposure

The reason for most human exposures was unintentional (79.9%) with unintentional general (54.2%), therapeutic error (12.5%), and unintentional misuse (5.6%) of all exposures (Table 6A).

**Table 4.** Distribution of Age^a^ and Gender for Fatalities^b.^

**Age (y)**	**Male**	**Female**	**Unknown**	**Total (%)**	**Cumulative total (%)**
< 1 year	4	0	0	4 (0.3%)	4 (0.3%)
1 year	7	5	0	12 (1.0%)	16 (1.3%)
2 years	2	1	0	3 (0.3%)	19 (1.6%)
3 years	3	2	0	5 (0.4%)	24 (2.0%)
4 years	2	1	0	3 (0.3%)	27 (2.2%)
5 years	1	1	0	2 (0.2%)	29 (2.4%)
Child 6–12 years	3	3	0	6 (0.5%)	35 (2.9%)
Teen 13–19 years	37	26	1	64 (5.3%)	99 (8.1%)
20–29 years	103	88	0	191 (15.7%)	290 (23.8%)
30–39 years	93	101	0	194 (15.9%)	484 (39.7%)
40–49 years	98	109	0	207 (17.0%)	691 (56.7%)
50–59 years	111	115	0	226 (18.6%)	917 (75.3%)
60–69 years	72	66	0	138 (11.3%)	1,055 (86.6%)
70–79 years	35	41	0	76 (6.2%)	1,131 (92.9%)
80–89 years	23	45	0	68 (5.6%)	1,199 (98.4%)
> = 90 years	5	5	0	10 (0.8%)	1,209 (99.3%)
Unknown adult	2	3	0	5 (0.4%)	1,214 (99.7%)
Unknown age	0	2	2	4 (0.3%)	1,218 (100.0%)
**Total**	**601**	**614**	**3**	**1,218 (100.0%)**	**1,218 (100.0%)**

^a^Age includes cases with both actual and estimated ages as shown in Table 21. ^b^Includes cases with relative contribution to fatality of 1—undoubtedly responsible, 2—probably responsible, or 3—contributory. This excludes reports with outcome of Death INDIRECT.

**Table 5.** Number of Substances Involved in Human Exposure Cases.

**No. of Substances**	**Human exposures**		**Fatal exposures**^a^	
1	1,950,455	89.14	538	44.17
2	149,026	6.81	295	24.22
3	48,980	2.24	152	12.48
4	20,504	0.94	105	8.62
5	9,116	0.42	62	5.09
6	4,436	0.20	25	2.05
7	2,262	0.10	18	1.48
8	1,265	0.06	5	0.41
> = 9	1,969	0.09	18	1.48
**Total**	2,188,013	100.00	1,218	100.00

^a^Includes cases with relative contribution to fatality of 1–undoubtedly responsible, 2—probably responsible, or 3—contributory. This excludes reports with outcome of Death INDIRECT.

**Table 6A.** Reason for Human Exposure Cases.

**Reason**	**N**	**% Human exposures**
**Unintentional**		
Unintentional—General	1,185,997	54.2
Unintentional—Therapeutic error	272,623	12.5
Unintentional—Misuse	123,229	5.6
Unintentional—Environmental	58,365	2.7
Unintentional—Bite/sting	56,378	2.6
Unintentional—Occupational	25,886	1.2
Unintentional—Food poisoning	21,334	1.0
Unintentional—Unknown	3,724	0.2
**Subtotal**	**1,747,536**	**79.9**
**Intentional**		
Intentional—Suspected suicide	230,080	10.5
Intentional—Misuse	55,740	2.5
Intentional—Abuse	48,976	2.2
Intentional—Unknown	20,151	0.9
**Subtotal**	**354,947**	**16.2**
**Adverse Reaction**		
Adverse reaction—Drug	38,198	1.7
Adverse reaction—Other	10,637	0.5
Adverse reaction—Food	5,146	0.2
**Subtotal**	**53,981**	**2.5**
**Unknown**		
Unknown reason	15,670	0.7
**Subtotal**	**15,670**	**0.7**
**Other**		
Other—Malicious	7,261	0.3
Other—Contamination/tampering	7,046	0.3
Other—Withdrawal	1,572	0.1
**Subtotal**	**15,879**	**0.7**
**Total**	**2,188,013**	**100.0**

### Scenarios

Of the total 289,699 therapeutic errors, the most common scenarios for all ages included: inadvertent double dosing (28.2%), wrong medication taken or given (16.2%), other incorrect dose (13.6%), doses given/taken too close together (10.3%), and inadvertent exposure to someone else's medication (8.0%). The types of therapeutic errors observed are different for each age group and are summarized in Table 6B.

**Table 6B.** Scenarios for Therapeutic Errors^a^ by Age^b^.

**Scenario**	**N**	**< = 5 y (Row %)**	**6–12 y (Row %)**	**13–19 y (Row %)**	**> = 20 y (Row %)**	**Unknown child (Row %)**	**Unknown adult (Row %)**	**Unknown age (Row %)**
Inadvertently took/given medication twice	81,591	17.03	12.88	5.83	58.07	0.07	5.92	0.20
Wrong medication taken/given	46,802	15.75	12.57	6.35	59.29	0.05	5.74	0.25
Other incorrect dose	39,264	32.17	11.98	6.49	45.36	0.12	3.63	0.24
Medication doses given/taken too close together	29,735	17.33	9.93	6.49	59.24	0.08	6.67	0.27
Inadvertently took/given someone else's medication	23,247	16.53	20.83	7.08	50.89	0.05	4.44	0.17
Other/unknown therapeutic error	16,460	20.44	11.10	6.87	53.71	0.18	7.17	0.54
Incorrect dosing route	15,564	7.83	3.96	3.30	72.98	0.10	11.19	0.64
Confused units of measure	10,391	57.51	18.49	4.05	18.20	0.09	1.54	0.13
Dispensing cup error	5,892	66.06	19.45	3.00	10.61	0.07	0.73	0.08
Health professional/iatrogenic error (pharmacist/nurse/physician)	5,630	26.93	11.37	6.63	48.03	0.16	5.79	1.10
Incorrect formulation or concentration given	5,622	46.16	16.88	4.73	29.06	0.21	2.86	0.09
More than 1 product containing same ingredient	4,913	11.99	15.20	13.70	52.33	0.08	6.37	0.33
Drug interaction	2,003	6.74	7.64	7.74	63.70	0.10	13.38	0.70
10-fold dosing error	1,282	57.41	9.83	3.74	26.60	0.00	2.18	0.23
Incorrect formulation or concentration dispensed	1,163	44.54	16.34	5.07	29.84	0.00	3.87	0.34
Exposure through breast milk	140	93.57	0.00	0.00	2.86	1.43	2.14	0.00

^a^All cases with a scenario category of therapeutic error regardless of reason

^b^Of the human exposure cases reported to U.S. Poison Centers in 2013, 407,832 (18.6%) were coded to 1 or more of 54 scenarios.

### Reason by Age

Intentional exposures accounted for 16.2% of human exposures. Suicidal intent was suspected in 10.5% of cases, intentional misuse in 2.5%, and intentional abuse in 2.2%. Unintentional exposures outnumbered intentional exposures in all age groups with the exception of ages 13–19 years (Table 7). Intentional exposures were more frequently reported than unintentional exposures in patients aged 13–19 years. In contrast, of the 1,218 reported fatalities with RCF 1–3, the major reason reported for children ≤ 5 years was unintentional while most fatalities in adults (> 20 years) were intentional (Table 8).

**Table 7.** Distribution of Reason for Exposure by Age.

	**< = 5 y**		**6–12 y**		**13–19 y**		**> = 20 y**		**Unknown child**		**Unknown adult**		**Unknown age**		**Total**	
**Reason**	**N**	**Row %**	**N**	**Row %**	**N**	**Row %**	**N**	**Row %**	**N**	**Row %**	**N**	**Row %**	**N**	**Row %**	**N**	**%**
Unintentional	1,042,537	62.32	118,754	7.10	60,129	3.59	444,092	26.55	3,617	0.22	71,059	4.25	7,348	0.44	1,747,536	79.87
Intentional	1,303	0.38	11,081	3.23	85,380	24.88	242,112	70.55	199	0.06	11,575	3.37	3,297	0.96	354,947	16.22
Adverse reaction	3,675	7.70	2,873	6.02	3,995	8.37	36,314	76.12	111	0.23	6,164	12.92	849	1.78	53,981	2.47
Unknown	741	5.17	864	6.02	1,848	12.88	10,093	70.37	54	0.38	1,273	8.88	797	5.56	15,670	0.72
Other	1,219	8.97	1,573	11.57	1,785	13.13	8,722	64.15	124	0.91	2,159	15.88	297	2.18	15,879	0.73
**Total**	**1,049,475**	**50.17**	**135,145**	**6.46**	**153,137**	**7.32**	**741,333**	**35.44**	**4,105**	**0.20**	**92,230**	**4.41**	**12,588**	**0.60**	**2,188,013**	**100.00**

**Table 8.** Distribution of Reason for Exposure and Age for Fatalities^a^.

**Reason**	**< = 5 y**	**6 –12 y**	**13–19 y**	**> = 20 y**	**Unknown child**	**Unknown adult**	**Unknown age**	**Total**
**Unintentional**								
Unintentional—General	14	0	1	13	0	0	0	28
Unintentional—Environmental	7	4	1	37	0	1	0	50
Unintentional—Occupational	0	0	0	9	0	0	0	9
Unintentional—Therapeutic error	2	0	1	32	0	0	0	35
Unintentional —Misuse	0	0	0	5	0	0	0	5
Unintentional—Bite/sting	1	0	0	3	0	0	0	4
Unintentional —Food poisoning	0	0	0	1	0	0	0	1
Unintentional —Unknown	0	0	0	3	0	0	0	3
**Subtotal**	24	4	3	103	0	1	0	135
**Intentional**								
Intentional —Suspected suicide	0	1	29	577	0	3	2	612
Intentional—Misuse	0	0	1	46	0	0	0	47
Intentional —Abuse	0	1	26	129	0	0	0	156
Intentional —Unknown	0	0	1	83	0	0	0	84
**Subtotal**	0	2	57	835	0	3	2	899
**Other**								
Other —Malicious	3	0	0	7	0	0	1	11
Other—Withdrawal	0	0	0	2	0	0	0	2
**Subtotal**	3	0	0	9	0	0	1	13
**Adverse reaction**								
Adverse reaction—Drug	0	0	2	42	0	0	0	44
Adverse reaction—Food	0	0	0	1	0	0	0	1
Adverse reaction—Other	0	0	0	1	0	0	0	1
**Subtotal**	0	0	2	44	0	0	0	46
**Unknown**								
Unknown reason	2	0	2	119	0	1	1	125
**Subtotal**	2	0	2	119	0	1	1	125
**Total**	29	6	64	1,110	0	5	4	1,218

^a^Includes cases with relative contribution to fatality of 1—undoubtedly responsible, 2—probably responsible, or 3-contributory. This excludes reports with outcome of Death INDIRECT.

### Route of Exposure

Ingestion was the route of exposure in 83.4% of cases (Table 9), followed in frequency by dermal (7.0%), inhalation/nasal (6.1%), and ocular routes (4.3%). For the 1,218 exposure-related fatalities, ingestion (80.9%), inhalation/nasal (10.2%), unknown (8.9%), and parenteral (5.1%) were the predominant exposure routes. Each exposure case may have more than one route.

**Table 9.** Route of Exposure for Human Exposure Cases.

	**Human exposures**			**Fatal exposures**^a^		
**Route**	**N**	**% of All Routes**	**% of All Cases**	**N**	**% of All Routes**	**% of All Cases**
Ingestion	1,824,913	79.40	83.41	985	75.08	80.87
Dermal	152,028	6.61	6.95	11	0.84	0.90
Inhalation/nasal	134,143	5.84	6.13	124	9.45	10.18
Ocular	93,673	4.08	4.28	1	0.08	0.08
Bite/sting	56,376	2.45	2.58	4	0.30	0.33
Parenteral	18,973	0.83	0.87	62	4.73	5.09
Unknown	11,022	0.48	0.50	108	8.23	8.87
Other	2,611	0.11	0.12	4	0.30	0.33
Otic	1,901	0.08	0.09	0	0.0	0
Aspiration (with ingestion)	1,175	0.05	0.05	13	0.99	1.07
Vaginal	915	0.04	0.04	0	0.0	0
Rectal	736	0.03	0.03	0	0.0	0
**Total Number of Routes^b^**	**2,298,466**	**100.00**	**105.05**	**1,312**	**100.00**	**107.72**

^a^Includes cases with relative contribution to fatality of 1—undoubtedly responsible, 2—probably responsible, or 3—contributory. This excludes reports with outcome of Death INDIRECT.

^b^Each exposure case may have more than one route.

### Clinical Effects

The NPDS database allows for the coding of up to 131 individual clinical effects (signs, symptoms, or laboratory abnormalities) for each case. Each clinical effect can be further defined as related, not related, or unknown if related. Clinical effects were coded in 810,259 (37.0%) cases (17.8% had 1 effect, 9.5% had 2 effects, 5.1% had 3 effects, 2.2% had 4 effects, 1.0% had 5 effects, and 1.4% had > 5 effects coded). Of the clinical effects coded, 77.8% were deemed related to the exposure, 9.9% were considered not related, and 12.3% were coded as unknown if related.

### Case Management Site

The majority of cases reported to PCs were managed in a non-HCF (68.7%), usually at the site of exposure, primarily the patient's own residence (Table 10); 1.5% of cases were referred to a HCF but they refused referral. Treatment in a HCF was rendered in 27.5% of cases.

**Table 10.** Management Site of Human Exposures.

**Site of management**	**N**	**%**
Managed on site, non-health care facility	1,502,483	68.7
Managed in health care facility		
Treated/evaluated and released	286,690	13.1
Admitted to critical care unit	99,117	4.5
Patient lost to follow-up/left AMA	86,725	4.0
Admitted to noncritical care unit	67,114	3.1
Admitted to psychiatric facility	61,996	2.8
**Subtotal (managed in HCF)**	**601,642**	**27.5**
Other	27,929	1.3
Refused referral	33,305	1.5
Unknown	22,654	1.0
**Total**	**2,188,013**	**100.0**

Of the 601,642 cases managed in a HCF, 286,690(47.7%) were treated and released, 99,117(16.5%) were admitted for critical care, and 67,114(11.2%) were admitted to a noncritical unit.

The percentage of patients treated in a HCF varied considerably with age. Only 11.8% of children ≤ 5 years and only 14.7% of children between 6 and 12 years were managed in a HCF compared with 54.1% of teenagers (13–19 years) and 41.7% of adults (age, ≥ 20 years).

### Medical Outcome

Table 11 displays the medical outcome of human exposure cases distributed by age. Older age groups exhibit a greater number of severe medical outcomes. Table 12 compares medical outcome and reason for exposure, and shows a greater frequency of serious outcomes in intentional exposures.

**Table 11.** Medical Outcome of Human Exposure Cases by Patient Age^a.^

	**< = 5 y**		**6–12 y**		**13–19 y**		**> = 20 y**		**Unknown child**		**Unknown adult**		**Unknown age**		**Total**	
**Outcome**	**N**	**%**	**N**	**%**	**N**	**%**	**N**	**%**	**N**	**%**	**N**	**%**	**N**	**%**	**N**	**%**
No effect	245,313	23.37	23,884	17.67	26,679	17.42	92,502	12.48	726	17.69	8,360	9.06	1,244	9.9	398,708	18.22
Minor effect	87,976	8.38	20,104	14.88	40,321	26.33	170,777	23.04	365	8.89	12,134	13.16	1,793	14.2	333,470	15.24
Moderate effect	10,433	0.99	4,066	3.01	23,442	15.31	107,877	14.55	82	2.00	3,030	3.29	352	2.8	149,282	6.82
Major effect	763	0.07	217	0.16	2,238	1.46	17,346	2.34	2	0.05	156	0.17	27	0.2	20,749	0.95
Death	43	0.00	11	0.01	74	0.05	1,396	0.19	0	0.00	17	0.02	11	0.1	1,552	0.07
No follow-up, nontoxic	199,344	18.99	20,030	14.82	7,566	4.94	45,166	6.09	583	14.20	11,657	12.64	880	7.0	285,226	13.04
No follow-up, minimal toxicity	472,491	45.02	60,556	44.81	37,953	24.78	230,608	31.11	1,614	39.32	40,579	44.00	3,544	28.2	847,345	38.73
No follow-up, potentially toxic	18,711	1.78	3,080	2.28	10,934	7.14	44,793	6.04	601	14.64	12,566	13.62	4,389	34.9	95,074	4.35
Unrelated effect	14,393	1.37	3,192	2.36	3,913	2.56	29,986	4.04	132	3.22	3,719	4.03	347	2.8	55,682	2.54
Death, indirect report	8	0.00	5	0.00	17	0.01	882	0.12	0	0.00	12	0.01	1	0.0	925	0.04
**Total**	**1,049,475**	**100.00**	**135,145**	**100.0**	**153,137**	**100.00**	**741,333**	**100.00**	**4,105**	**100.00**	**92,230**	**100.00**	**12,588**	**100.00**	**2,188,013**	**100.00**

^a^Total number of cases where Death was an outcome (1,552 + 925) is greater than the number of fatalities (1,218) judged to be exposure-related (relative contribution to fatality of 1—undoubtedly responsible, 2—probably responsible, or 3—contributory).

**Table 12.** Medical Outcome by Reason for Exposure in Human Exposures^a.^

	**Unintentional**		**Intentional**		**Other**		**Adverse reaction**		**Unknown**		**Total**	
**Outcome**	**N**	**%**	**N**	**%**	**N**	**%**	**N**	**%**	**N**	**%**	**N**	**%**
Death	172	0.01	1,038	0.29	25	0.16	81	0.15	236	1.51	1,552	0.07
Death, indirect report	59	0.00	827	0.23	6	0.04	5	0.01	28	0.18	925	0.04
Major effect	2,563	0.15	16,011	4.51	150	0.94	748	1.39	1,277	8.15	20,749	0.95
Minor effect	215,265	12.32	99,939	28.16	2,973	18.72	12,731	23.58	2,562	16.35	333,470	15.24
Moderate effect	44,276	2.53	92,424	26.04	1,232	7.76	7,638	14.15	3,712	23.69	149,282	6.82
No effect	335,880	19.22	58,387	16.45	1,779	11.20	1,492	2.76	1,170	7.47	398,708	18.22
No follow-up, nontoxic	278,497	15.94	4,421	1.25	1,090	6.86	986	1.83	232	1.48	285,226	13.04
No follow-up, minimal toxicity	787,499	45.06	33,987	9.58	5,750	36.21	18,249	33.81	1,860	11.87	847,345	38.73
No follow-up, potentially toxic	46,211	2.64	40,253	11.34	1,682	10.59	3,801	7.04	3,127	19.96	95,074	4.35
Unrelated effect	37,114	2.12	7,660	2.16	1,192	7.51	8,250	15.28	1,466	9.36	55,682	2.54
**Total**	**1,747,536**	**100.00**	**354,947**	**100.00**	**15,879**	**100.00**	**53,981**	**100.00**	**15,670**	**100.00**	**2,188,013**	**100.00**

^a^Total number of cases where Death was an outcome (1,552 + 925) is greater than the number of fatalities (1,218) judged to be exposure-related (relative contribution to fatality of 1—undoubtedly responsible, 2—probably responsible, or 3—contributory).

The duration of effect is required for all cases which report at least one clinical effect and have a medical outcome of minor, moderate, or major effect (*n* = 503,501; 23.0% of exposures). Table 13 demonstrates an increasing duration of the clinical effects observed with more severe outcomes.

**Table 13.** Duration of Clinical Effects by Medical Outcome.

	**Minor effect**		**Moderate effect**		**Major effect**	
**Duration of effect**	**N**	**%**	**N**	**%**	**N**	**%**
< = 2 hours	110,524	33.14	7,550	5.06	409	1.97
> 2 hours, < = 8 hours	88,918	26.66	29,991	20.09	1,128	5.44
> 8 hours, < = 24 hours	60,828	18.24	52,909	35.44	4,627	22.30
> 24 hours, < = 3 days	22,157	6.64	29,252	19.60	7,020	33.83
> 3 days, < = 1 week	4,075	1.22	7,484	5.01	3,751	18.08
> 1 week, < = 1 month	1,280	0.38	1,736	1.16	1,143	5.51
> 1 month	385	0.12	403	0.27	160	0.77
Anticipated permanent	535	0.16	206	0.14	378	1.82
Unknown	44,768	13.42	19,751	13.23	2,133	10.28
**Total**	**333,470**	**100.00**	**149,282**	**100.00**	**20,749**	**100.00**

### Decontamination Procedures and Specific Antidotes

Tables 14 and 15 outline the use of decontamination procedures, specific physiological antagonists (antidotes), and measures to enhance elimination in the treatment of patients reported in the NPDS database. These should be interpreted as minimum frequencies because of the limitations of telephone data gathering.

**Table 14.** Decontamination and Therapeutic Interventions.

**Therapy**	**N**	**%**
Decontamination Only	1,066,542	48.7
Therapeutic Intervention Only	244,074	11.2
Decontamination and Therapeutic Intervention	152,943	7.0
Not Coded	724,454	33.1
**Total**	**2,188,013**	**100.0**

**Table 15.** Therapy Provided in Human Exposures by Age.

**Therapy**		**< = 5 y**	**6–12 y**	**13–19 y**	**> = 20 y**	**Unknown child**	**Unknown adult**	**Unknown age**	**Total**
**Decontamination**									
	Cathartic	858	186	2,389	6,187	1	74	6	9,701
	Charcoal, multiple doses	73	15	335	926	0	2	0	1,351
	Charcoal, single dose	9,261	1,000	11,491	27,076	8	246	26	49,108
	Dilute/irrigate/wash	515,442	53,440	30,488	189,343	1,072	30,392	2,481	822,658
	Food/snack	135,721	11,917	6,000	30,972	134	4,499	172	189,415
	Fresh air	6,512	4,413	5,134	41,721	591	10,524	968	69,863
	Ipecac	42	10	32	48	0	2	0	134
	Lavage	79	13	558	2,100	0	25	1	2,776
	Other emetic	6,221	557	967	4,693	9	390	42	12,879
	Whole bowel irrigation	81	27	299	1,439	0	7	1	1,854
**Other Therapies**									
	2-PAM	2	1	4	41	0	3	0	51
	Alkalinization	144	75	1,908	8,867	0	43	6	11,043
	Amyl nitrite	0	0	1	7	0	0	0	8
	Antiarrhythmic	12	8	181	1,247	0	5	1	1,454
	Antibiotics	1,878	867	1,191	12,725	12	637	72	17,382
	Anticonvulsants^a^	58	22	138	890	0	3	0	1,111
	Antiemetics	1,237	520	5,177	12,277	3	131	13	19,358
	Antihistamines	2,221	1,469	1,782	9,850	14	1,023	76	16,435
	Antihypertensives	23	16	137	2,412	0	13	1	2,602
	Antivenin (fab fragment)	216	183	220	1,426	0	13	6	2,064
	Antivenin/antitoxin^b^	28	28	36	251	0	2	0	345
	Atropine	117	22	107	1,261	0	13	0	1,520
	BAL	7	1	1	17	0	0	0	26
	Benzodiazepines	1,037	495	5,636	25,967	1	198	26	33,360
	Bronchodilators	518	254	378	4,394	8	254	15	5,821
	Calcium	8,570	592	312	2,422	1	89	14	12,000
	Cardioversion	1	0	20	276	0	1	0	298
	CPR	53	7	94	1,044	1	8	4	1,211
	Deferoxamine	5	3	23	28	0	0	0	59
	ECMO	5	0	9	15	0	0	0	29
	EDTA	20	4	2	11	0	1	0	38
	Ethanol	0	0	4	38	0	1	0	43
	Extracorp. procedure (other)	0	0	3	28	0	0	0	31
	Fab fragments	22	30	22	667	0	4	1	746
	Fluids, IV	6,845	2,260	27,689	116,128	12	797	92	153,823
	Flumazenil	106	12	147	1,412	0	11	0	1,688
	Folate	12	1	32	1,033	0	4	0	1,082
	Fomepizole	97	16	90	1,590	0	9	2	1,804
	Glucagon	31	3	101	1,869	0	7	0	2,011
	Glucose, > 5%	385	34	274	3,289	0	25	5	4,012
	Hemodialysis	5	7	111	2,290	0	10	1	2,424
	Hemoperfusion	2	0	3	49	0	0	0	54
	Hydroxocobalamin	6	5	4	67	0	0	2	84
	Hyperbaric oxygen	29	21	29	306	0	10	9	404
	Insulin	13	8	111	1,829	0	3	0	1,964
	Intubation	534	114	1,672	18,481	0	121	26	20,948
	Methylene blue	18	4	10	114	0	2	1	149
	NAC, IV	216	155	4,023	14,237	0	78	12	18,721
	NAC, PO	51	39	1,005	3,104	1	20	4	4,224
	Nalmefene	0	0	4	16	0	0	0	20
	Naloxone	1,021	162	1,556	16,632	1	130	16	19,518
	Neuromuscular blocker	58	8	157	1,205	0	3	0	1,431
	Octreotide	85	5	40	292	0	1	0	423
	Other	39,246	8,616	13,157	81,394	147	4,248	1,060	147,868
	Oxygen	1,575	731	3,593	41,812	17	501	91	48,320
	Pacemaker	2	1	3	202	0	1	0	209
	Penicillamine	1	0	0	3	0	1	0	5
	Physostigmine	10	7	65	188	0	1	0	271
	Phytonadione	16	4	56	717	0	3	1	797
	Pyridoxine	5	3	37	308	0	0	0	353
	Sedation (other)	337	84	1,582	14,546	0	74	12	16,635
	Sodium nitrite	0	0	3	25	0	0	0	28
	Sodium thiosulfate	1	1	2	32	0	0	0	36
	Steroids	708	391	492	4,534	17	376	29	6,547
	Succimer	78	11	8	52	0	2	0	151
	Transplantation	0	0	4	13	0	0	0	17
	Vasopressors	74	29	364	5,291	0	25	1	5,784
	Ventilator	482	104	1,558	17,392	0	109	24	19,669

^a^Excludes benzodiazepines.

^b^Excludes Fab fragments.

Ipecac-induced emesis for poisoning continues to decline as shown in Tables 16A and 16B. Ipecac was administered in only 42 (0.0%) of pediatric exposures in 2013. The continued decrease in ipecac syrup use over the last 2 decades is likely a result of ipecac use guidelines issued in 1997 by the American Academy of Clinical Toxicology and the European Association of Poisons Centres and Clinical Toxicologists and updated in 2004.(6,7) In a separate report, the American Academy of Pediatrics not only concluded that ipecac should no longer be used routinely as a home treatment strategy, but also recommended disposal of home ipecac stocks.(8) A decline was also observed since the early 1990s for reported use of activated charcoal. While not as dramatic as the decline in use of ipecac, reported use of activated charcoal decreased from 3.7% of pediatric cases in 1993 to just 0.9% in 2013.

**Table 16A.** Decontamination Trends (1985–2013).

**Year**	**Human exposures**	**Ipecac administered (% of all exposures)**	**Activated charcoal administered (% of all exposures)**	**Exposures involving children ≤ 5 y (% of all exposures)**	**Ipecac administered (% of child exposures)**	**Activated charcoal administered (% of child exposures)**
1985	886,389	132,947 (14.999)	41,063 (4.6)	568,691 (64.2)	94,919 (16.6908)	14,718 (2.59)
1986	1,095,228	145,516 (13.286)	56,481 (5.2)	690,137 (63.0)	99,688 (14.4447)	18,191 (2.64)
1987	1,164,648	117,840 (10.118)	60,310 (5.2)	730,228 (62.7)	83,443 (11.427)	18,507 (2.53)
1988	1,364,113	114,654 (8.4050)	88,876 (6.5)	843,106 (61.8)	80,749 (9.5776)	26,118 (3.10)
1989	1,578,968	110,545 (7.0011)	101,368 (6.4)	963,924 (61.0)	79,192 (8.2156)	30,345 (3.15)
1990	1,646,946	98,986 (6.0103)	108,341 (6.6)	999,751 (60.7)	73,469 (7.3487)	31,579 (3.16)
1991	1,836,364	94,877 (5.1666)	129,092 (7.0)	1,099,179 (59.9)	73,069 (6.6476)	36,177 (3.29)
1992	1,862,796	79,493 (4.2674)	135,625 (7.3)	1,094,256 (58.7)	63,486 (5.8018)	38,937 (3.56)
1993	1,747,147	65,078 (3.7248)	127,893 (7.3)	978,560 (56.0)	50,834 (5.1948)	35,791 (3.66)
1994	1,926,992	51,356 (2.6651)	138,247 (7.2)	1,042,651 (54.1)	41,489 (3.9792)	35,670 (3.42)
1995	2,023,089	47,359 (2.3409)	155,880 (7.7)	1,070,472 (52.9)	38,372 (3.5846)	38,095 (3.56)
1996	2,155,952	39,376 (1.8264)	157,331 (7.3)	1,137,263 (52.7)	32,622 (2.8685)	37,986 (3.34)
1997	2,192,088	32,098 (1.4643)	156,213 (7.1)	1,150,931 (52.5)	26,536 (2.3056)	35,856 (3.12)
1998	2,241,082	26,653 (1.1893)	152,134 (6.8)	1,180,989 (52.7)	22,247 (1.8838)	34,302 (2.90)
1999	2,201,156	21,942 (0.9968)	145,853 (6.6)	1,154,799 (52.5)	18,326 (1.5869)	33,812 (2.93)
2000	2,168,248	18,177 (0.8383)	145,911 (6.7)	1,142,796 (52.7)	15,239 (1.3335)	31,554 (2.76)
2001	2,267,979	16,058 (0.7080)	149,442 (6.6)	1,169,478 (51.6)	13,389 (1.1449)	30,367 (2.60)
2002	2,380,028	13,555 (0.5695)	149,527 (6.3)	1,227,381 (51.6)	11,163 (0.9095)	30,340 (2.47)
2003	2,395,582	9,284 (0.3875)	140,412 (5.9)	1,245,584 (52.0)	7,310 (0.5869)	28,888 (2.32)
2004	2,438,643	4,701 (0.1928)	135,969 (5.6)	1,250,536 (51.3)	3,366 (0.2692)	28,335 (2.27)
2005	2,424,180	3,027 (0.1249)	123,263 (5.1)	1,233,695 (50.9)	1,999 (0.1620)	26,338 (2.13)
2006	2,403,539	2,176 (0.0905)	111,351 (4.6)	1,223,815 (50.9)	1,337 (0.1092)	23,843 (1.95)
2007	2,482,041	1,740 (0.0701)	106,010 (4.3)	1,271,595 (51.2)	1,052 (0.0827)	22,829 (1.80)
2008	2,491,049	1,205 (0.0484)	97,297 (3.9)	1,292,754 (51.9)	641 (0.0496)	21,286 (1.65)
2009	2,479,355	658 (0.0265)	84,805 (3.4)	1,290,784 (52.1)	330 (0.0256)	19,168 (1.48)
2010	2,384,825	360 (0.0151)	74,431 (3.1)	1,207,575 (50.6)	163 (0.0135)	16,581 (1.37)
2011	2,334,004	262 (0.0112)	66,770 (2.9)	1,144,729 (49.1)	98 (0.0086)	13,930 (1.22)
2012	2,275,141	193 (0.0085)	57,888 (2.5)	1,102,307 (48.5)	83 (0.0075)	11,284 (1.02)
2013	2,188,013	134 (0.0061)	50,459 (2.3)	1,049,475 (48.0)	42 (0.0040)	9,334 (0.89)

**Table 16B.** Decontamination Trends: Total Human and Pediatric Exposures < = 5 Years^a.^

	**Human exposures**		**Exposures children < = 5 y**	
**Therapy**	**N**	**%**	**N**	**%**
Activated charcoal administered	50,459	2.31	9,334	0.89
Cathartic	9,701	0.44	858	0.08
Ipecac administered	134	0.01	42	0.00
Lavage	2,776	0.13	79	0.01
Other emetic	12,879	0.59	6,221	0.59
Whole bowel irrigation	1,854	0.08	81	0.01
**Total**	**77,803**	**3.56**	**16,615**	**1.58**

^a^Human exposures = 2,188,013; Pediatric exposures = 1,049,475

### Top Substances in Human Exposures

Table 17A presents the most common 25 substance categories, listed by frequency of human exposure for cases with more serious outcomes (moderate, severe, and death). This ranking provides an indication where prevention efforts might be focused, as well as the types of serious exposures PCs regularly manage. It is relevant to know whether exposures to these substances are increasing or decreasing.

**Table 17A.** Substance Categories Most Frequently Involved in Human Exposures (Top 25).

**Substance (Major Generic Category)**	**All substances**	**%**^a^	**Single substance exposures**	**%**^b^
Analgesics	298,633	11.50	193,037	9.90
Cosmetics/personal care products	199,838	7.70	192,940	9.89
Cleaning substances (household)	196,183	7.55	175,594	9.00
Sedative/hypnotics/antipsychotics	153,398	5.91	57,901	2.97
Antidepressants	109,110	4.20	45,123	2.31
Foreign bodies/toys/miscellaneous	103,737	3.99	100,632	5.16
Cardiovascular drugs	101,544	3.91	46,406	2.38
Antihistamines	99,176	3.82	70,682	3.62
Topical preparations	89,287	3.44	87,278	4.47
Pesticides	85,033	3.27	79,405	4.07
Alcohols	70,258	2.71	24,176	1.24
Vitamins	66,206	2.55	56,914	2.92
Cold and cough preparations	65,053	2.51	46,581	2.39
Bites and envenomations	61,857	2.38	61,143	3.13
Stimulants and street drugs	58,514	2.25	33,278	1.71
Antimicrobials	58,514	2.25	48,259	2.47
Hormones and hormone antagonists	56,957	2.19	38,556	1.98
Anticonvulsants	53,102	2.04	21,957	1.13
Gastrointestinal preparations	47,698	1.84	36,180	1.85
Plants	46,376	1.79	43,947	2.25
Dietary supplements/herbals/homeopathic	38,955	1.50	31,254	1.60
Chemicals	38,873	1.50	32,959	1.69
Fumes/gases/vapors	33,973	1.31	31,244	1.60
Hydrocarbons	33,081	1.27	31,031	1.59
Electrolytes and minerals	30,498	1.17	25,089	1.29

^a^Percentages are based on the total number of substances reported in all exposures (N = 2,596,915)

^b^Percentages are based on the total number of single substance exposures (N = 1,950,455)

To better understand these relationships, we examined exposures with more serious outcomes per year over the last 13 years for the change over time for each of the 68 major generic categories via least-square linear regression. The serious outcome exposure calls per year over this period were increasing for 39 and decreasing for 29, respectively, of the 68 categories. The change over time for the 13 yearly values was statistically significant (p < 0.05) for 45 of the 68 categories. Table 17B shows the 25 categories which were increasing most rapidly. Statistical significance of the linear regressions can be verified by noting the 95% confidence interval on the rate of increase excluding 0 for all, but 3 of the 25 categories. Figure 5 shows the linear regressions for the top 4 increasing categories in Table 17B.

**Table 17B.** Substance Categories with the Greatest Rate of More Serious Exposure Increase (Top 25).

**Substance (major generic category)**	**Increase in more serious exposures per year^a^**		**More serious exposures in 2013**
	**Mean**	**95% CI**^b^	
Sedative/hypnotics/antipsychotics	2,559	[2,189, 2,923]	48,482
Analgesics	2,214	[1,953, 2,467]	46,227
Antidepressants	1,164	[1,010, 1,309]	33,924
Cardiovascular drugs	995	[935, 1,048]	19,136
Alcohols	944	[856, 1,031]	21,184
Stimulants and street drugs	650	[269, 1,032]	19,649
Anticonvulsants	608	[560, 656]	13,850
Muscle relaxants	516	[455, 576]	9,310
Antihistamines	493	[418, 567]	12,455
Cold and cough preparations	297	[220, 375]	8,485
Unknown drug	289	[241, 336]	6,123
Hormones and hormone antagonists	255	[236, 273]	5,818
Miscellaneous drugs	112	[73, 151]	2,118
Gastrointestinal preparations	73	[60, 87]	2,585
Diuretics	60	[48, 71]	1,389
Anticoagulants	53	[45, 62]	1,094
Other/unknown nondrug substances	51	[16, 85]	1,125
Vitamins	43	[35, 51]	952
Electrolytes and minerals	42	[33, 50]	965
Anticholinergic drugs	41	[30, 52]	1,117
Antimicrobials	25	[-5, 55]	2,573
Automotive/aircraft/boat products	17	[2, 32]	1,125
Swimming pool/aquarium	11	[-3, 25]	626
Essential oils	11	[9, 12]	227
Cosmetics/personal care products	8	[-3, 20]	2,472

^a^More Serious exposures have medical outcomes of moderate, major or death.

^b^Increase and confidence intervals are based on least-squares linear regression of the number of more serious exposures per year for 2000–2013.

Tables 17C and 17D present exposure results for children and adults, respectively, and show the differences between substance categories involved in pediatric and adult exposures.

**Table 17C.** Substance Categories Most Frequently Involved in Pediatric (≤ 5 years) Exposures (Top 25)^a.^

**Substance (major generic category)**	**All substances**	**%**^b^	**Single substance exposures**	**%**^c^
Cosmetics/personal care products	151,154	13.82	148,040	14.52
Cleaning substances (household)	113,872	10.41	109,548	10.75
Analgesics	106,639	9.75	97,388	9.55
Foreign bodies/toys/miscellaneous	75,184	6.88	73,366	7.20
Topical preparations	66,893	6.12	65,756	6.45
Vitamins	47,816	4.37	43,355	4.25
Antihistamines	45,250	4.14	40,983	4.02
Pesticides	35,254	3.22	34,246	3.36
Plants	29,346	2.68	28,296	2.78
Gastrointestinal preparations	28,481	2.60	25,883	2.54
Antimicrobials	27,928	2.55	26,294	2.58
Cold and cough preparations	25,708	2.35	23,647	2.32
Dietary supplements/herbals/homeopathic	24,638	2.25	22,550	2.21
Cardiovascular drugs	23,124	2.11	14,645	1.44
Arts/crafts/office supplies	20,736	1.90	20,126	1.97
Hormones and hormone antagonists	20,522	1.88	15,869	1.56
Electrolytes and minerals	20,071	1.84	18,293	1.79
Deodorizers	17,555	1.61	17,354	1.70
Other/unknown nondrug substances	13,261	1.21	12,627	1.24
Sedative/hypnotics/antipsychotics	12,676	1.16	9,844	0.97
Antidepressants	11,526	1.05	8,343	0.82
Alcohols	11,026	1.01	10,756	1.06
Information Calls	9,984	0.91	9,389	0.92
Hydrocarbons	9,947	0.91	9,622	0.94
Asthma therapies	9,923	0.91	9,112	0.89

^a^Includes all children with actual or estimated ages ≤ 5 years old. Results do not include “Unknown Child” or “Unknown Age”.

^b^Percentages are based on the total number of substances reported in pediatric exposures (N = 1,093,578).

^c^Percentages are based on the total number of single substance pediatric exposures (N = 1,019,297).

**Table 17D.** Substance Categories Most Frequently Involved in Adult (≥ 20 years) Exposures (Top 25)^a.^

**Substance (major generic category)**	**All substances**	**%**^b^	**Single substance exposures**	**%**^c^
Analgesics	138,440	12.18	63,555	9.55
Sedative/hypnotics/antipsychotics	119,784	10.54	38,138	5.73
Antidepressants	74,818	6.58	25,534	3.84
Cardiovascular drugs	67,325	5.92	25,359	3.81
Cleaning substances (household)	66,408	5.84	52,395	7.87
Alcohols	52,430	4.61	10,422	1.57
Pesticides	42,055	3.70	38,022	5.71
Bites and envenomations	41,400	3.64	40,966	6.15
Anticonvulsants	38,709	3.41	13,606	2.04
Antihistamines	33,625	2.96	16,578	2.49
Cosmetics/personal care products	32,010	2.82	29,374	4.41
Hormones and hormone antagonists	31,223	2.75	19,038	2.86
Stimulants and street drugs	30,928	2.72	14,375	2.16
Fumes/gases/vapors	24,349	2.14	22,270	3.35
Chemicals	23,430	2.06	19,023	2.86
Antimicrobials	22,409	1.97	16,034	2.41
Cold and cough preparations	20,828	1.83	11,232	1.69
Muscle relaxants	20,351	1.79	7,117	1.07
Hydrocarbons	18,735	1.65	17,266	2.59
Topical preparations	17,288	1.52	16,645	2.50
Gastrointestinal preparations	15,005	1.32	7,599	1.14
Foreign Bodies/toys/miscellaneous	13,582	1.19	12,632	1.90
Miscellaneous drugs	12,173	1.07	6,095	0.92
Information calls	11,844	1.04	10,466	1.57
Other/unknown nondrug substances	11,514	1.01	10,092	1.52

^a^Includes all adults with actual or estimated ages ≥ 20 years old. Results also include “Unknown Adult” but do not include “Unknown Age”.

^b^Percentages are based on the total number of substances reported in adult exposures (N = 1,136,662).

^c^Percentages are based on the total number of single substance adult exposures (N = 665,623).

Table 17E reports the 25 categories of substances most frequently involved in pediatric (≤ 5 years) fatalities in 2013.

**Table 17E.** Substance Categories Most Frequently Involved in Pediatric (≤ 5 years) Deaths^a.^

**Substance (major generic category)**	**All substances**	**%**^b^	**Single substance exposures**	**%**^c^
Fumes/gases/vapors	11	17.46	7	16.28
Analgesics	10	15.87	5	11.63
Unknown drug	7	11.11	6	13.95
Batteries	4	6.35	4	9.30
Alcohols	3	4.76	3	6.98
Antidepressants	3	4.76	1	2.33
Antihistamines	3	4.76	1	2.33
Sedative/hypnotics/antipsychotics	3	4.76	1	2.33
Cleaning substances (household)	2	3.17	2	4.65
Hydrocarbons	2	3.17	2	4.65
Other/unknown nondrug substances	2	3.17	1	2.33
Pesticides	2	3.17	1	2.33
Anesthetics	1	1.59	1	2.33
Antineoplastics	1	1.59	1	2.33
Bites and envenomations	1	1.59	1	2.33
Cold and cough preparations	1	1.59	1	2.33
Deodorizers	1	1.59	1	2.33
Foreign bodies/toys/miscellaneous	1	1.59	0	0.00
Gastrointestinal preparations	1	1.59	1	2.33
Industrial cleaners	1	1.59	1	2.33
Miscellaneous drugs	1	1.59	1	2.33
Muscle relaxants	1	1.59	0	0.00
Stimulants and street drugs	1	1.59	1	2.33
**Total**	**63**	**100.00**	**43**	**100.00**

^a^Includes all children with actual or estimated ages ≤ 5 years old. Results do not include “Unknown Child” or “Unknown Age”. Includes death and death, indirect regardless of RCF.

^b^Percentages are based on the total number of substances reported in pediatric fatalities (N = 63).

^c^Percentages are based on the total number of single substance pediatric fatalities (N = 43).

Table 17F reports the 25 drug ID categories most frequently queried in 2013, highlighting the value of drug ID information to the AAPCC, public health, public safety, and regulatory agencies. Internet-based resources do not afford the caller the option to speak with a health care professional if needed. Proper resources to continue this vital public service are essential, especially since the top 10 substance categories include antibiotics as well as drugs with widespread use and abuse potential such as opioids and benzodiazepines.

**Table 17F.** Substance Categories Most Frequently Identified in Drug Identification Calls (Top 25).

**Substance (major generic category)**	**All substances**	**%**^a^
Analgesics	185,035	40.15
Sedative/hypnotics/antipsychotics	74,303	16.12
Unknown drug	28,811	6.25
Cardiovascular drugs	24,341	5.28
Muscle relaxants	24,057	5.22
Antidepressants	21,905	4.75
Antihistamines	17,835	3.87
Antimicrobials	14,324	3.11
Stimulants and street drugs	12,561	2.73
Anticonvulsants	11,929	2.59
Information Calls	10,934	2.37
Hormones and hormone antagonists	9,285	2.01
Gastrointestinal preparations	8,388	1.82
Diuretics	5,163	1.12
Miscellaneous drugs	3,247	0.70
Cold and cough preparations	2,189	0.47
Anticholinergic drugs	1,383	0.30
Electrolytes and minerals	903	0.20
Vitamins	867	0.19
Anticoagulants	846	0.18
Asthma therapies	719	0.16
Other/unknown nondrug substances	443	0.10
Dietary supplements/herbals/homeopathic	353	0.08
Antineoplastics	198	0.04
Anesthetics	149	0.03

^a^Percentages are based on the total number of substances reported in all drug identification calls (N = 460,850).

Table 17G reports the 25 substance categories most frequently reported in exposures involving pregnant patients.

**Table 17G.** Substance Categories Most Frequently Involved in Pregnant Exposures^a^ (Top 25).

**Substance (major generic category)**	**All substances**	**%**^b^	**Single substance exposures**	**%**^c^
Analgesics	984	11.61	601	9.06
Cleaning substances (household)	841	9.92	637	9.60
Pesticides	602	7.10	542	8.17
Fumes/gases/vapors	542	6.39	504	7.59
Bites and envenomations	523	6.17	519	7.82
Sedative/hypnotics/antipsychotics	356	4.20	176	2.65
Vitamins	275	3.24	216	3.25
Foreign bodies/toys/miscellaneous	274	3.23	261	3.93
Antihistamines	273	3.22	174	2.62
Cosmetics/personal care products	248	2.93	225	3.39
Antidepressants	243	2.87	137	2.06
Antimicrobials	221	2.61	159	2.40
Information Calls	205	2.42	177	2.67
Chemicals	190	2.24	168	2.53
Hydrocarbons	161	1.90	152	2.29
Stimulants and street drugs	156	1.84	87	1.31
Hormones and hormone antagonists	152	1.79	129	1.94
Cold and cough preparations	147	1.73	91	1.37
Alcohols	139	1.64	55	0.83
Gastrointestinal preparations	135	1.59	103	1.55
Other/unknown nondrug substances	132	1.56	119	1.79
Cardiovascular drugs	124	1.46	80	1.21
Infectious and toxin-mediated diseases	121	1.43	119	1.79
Topical preparations	121	1.43	116	1.75
Paints and stripping Agents	118	1.39	107	1.61

^a^Includes all patient classified as pregnant and all female patients with a ‘duration of pregnancy’ greater than 0.

^b^Percentages are based on the total number of substances reported in pregnant exposures (N = 8,477).

^c^Percentages are based on the total number of single substance pregnant exposures (N = 6,637).

### Changes Over Time

Total encounters peaked in 2008 at 4,333,012 calls with 2,491,049 human exposure calls and 1,703,762 information calls. Total encounters decreased 9.3% from 3,373,025 in 2012 to 3,060,122 in 2013. Information calls decreased by 21.4% from 1,025,547 calls in 2012 to 806,347 in 2013, with a 26.8% decrease in drug identification calls and a 8.5 % decrease in HCF information calls. Human exposures decreased by 3.8% from 2,275,141 to 2,188,013 cases.

**Figure 4.**
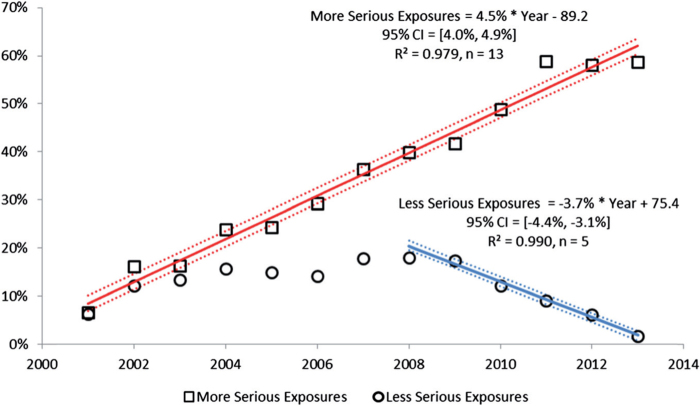
**Change in encounters by outcome from 2000.** The figure shows the percent change from baseline for Human Exposure Calls divided among the 10 Medical Outcomes. The More Serious Exposures (Major, Moderate, and Death) increased. The Less Serious Exposures (no effect, minor effect, not followed (non-toxic), not followed (minimal toxicity possible), unable to follow (potentially toxic), and unrelated effect) decreased after 2008. Solid lines show least-squares linear regressions for the change in More Serious Exposures per year (□) and Less Serious Exposures (○). Broken lines show 95% confidence interval on the regression (colour version of this figure can be found in the online version at www.informahealthcare.com/ctx).

Figure 4 shows the year-to-year change since 2000 as a percentage of year 2000 for human exposure calls broken down into cases with more serious outcomes (death, major effect, and moderate effect) and less serious outcomes [minor effect, no effect, not followed (non-toxic), not followed (minimal toxicity possible), unable to follow (potentially toxic), and unrelated effect]. Since 2000, cases with more serious outcomes have increased by 4.5% [95% CI (4.0%, 4.9%)] per year from 108,148 cases in 2000 to 171,583 cases in 2013. However, cases with less serious outcomes have consistently decreased since 2008 by 3.7% [95% CI (−4.4%, −3.1%)] per year from 2,339,460 in 2008 to 2,015,505 cases in 2013. This decrease in less serious exposures has driven the overall decrease in human exposures since 2008.

Likewise, we see a consistent increase in exposure calls from HCFs (Figure 3) and for the more severe exposures (Figure 4), despite a decrease in calls involving less severe exposures.

### Distribution of Suicides

Table 19A shows the modest variation in the distribution of suicides and pediatric deaths over the past 2 decades as reported to the NPDS national database. Within the last decade, the percentage of exposures determined to be suspected suicides ranged from 30.3%% to 53.9%, and the percentage of pediatric cases has ranged from 1.5% to 3.2%.The relatively large change seen for 2011 and 2012 reflects the large increase in indirect death reports in those years. Analyses of suicides and pediatric deaths for direct and indirect reports are shown in Table 19B.

### Plant Exposures

Table 20 provides the number of times the specific plant was reported to NPDS (*n* = 46,376). The 25 most commonly involved plant species and categories account for 39.7% of all plant exposures reported. The top 3 categories in the table are essentially synonymous for unknown plant and comprise 12.8% (5,955/46,376) of all plant exposures. For several reasons, it was not possible to make a precise identification in these three groups. The top most frequent plant exposures where a positive plant identification was made were the following (descending order): *Phytolacca americana* (L.) (Botanic name), *Spathiphyllum* species (Botanic name), *Cherry* (Species unspecified), *Ilex* species (Botanic name), *Philodendron* (Species unspecified), *Caladium species* (Botanic name of all species of the genus caladium) and *Malus* species (Botanic name)

### Deaths and Exposure-related Fatalities

A list of cases (Table 21) and summary of cases (Tables 4, 5, 8, 9, 18, and 22) are provided for fatal cases for which there exists reasonable confidence that the death was a result of that exposure (exposure-related fatalities). Tables 11, 12, and 19 list all deaths, irrespective of the RCF. Beginning in 2010, cases with outcome of Death, Indirect Report were not further reviewed by the AAPCC fatality review team, and the RCF was determined by the individual PC review team.

**Table:** 

Table	Fatalities included	RCF	N
4	Death only	1,2,3	1,218
5	Death only	1,2,3	1,218
8	Death only	1,2,3	1,218
9	Death only	1,2,3	1,218
11	Death and Death (indirect report)	All	2,477
12	Death and Death (indirect report)	All	2,477
17E	Pediatric Death and Death (indirect report)	All	51
18	Death only	1,2,3	1,218
19A	Death and Death (indirect report)	All	2,477
19B	Death and Death (indirect report)	All	2,477
21	Death and Death (indirect report)	1,2,3	2,113
22	Death and Death (indirect report) – Single-substance deaths only	All	1,001

**Table 18.** Categories Associated with Largest Number of Fatalities (Top 25)^a^.

**Substance (minor generic category)**	**All substances**	**%^b^**	**Single substance exposures**	**%^c^**
Miscellaneous sedative/hypnotics/antipsychotics	363	12.86	19	3.53
Miscellaneous cardiovascular drugs	301	10.67	58	10.78
Opioids	243	8.61	34	6.32
Miscellaneous stimulants and street drugs	210	7.44	44	8.18
Miscellaneous alcohols	174	6.17	12	2.23
Acetaminophen combinations	153	5.42	44	8.18
Acetaminophen alone	145	5.14	58	10.78
Selective serotonin reuptake inhibitors (SSRI)	92	3.26	4	0.74
Miscellaneous fumes/gases/vapors	89	3.15	53	9.85
Miscellaneous antidepressants	77	2.73	6	1.12
Miscellaneous antihistamines	70	2.48	5	0.93
Tricyclic antidepressants (TCA)	64	2.27	12	2.23
Acetylsalicylic acid alone	62	2.20	22	4.09
Miscellaneous muscle relaxants	60	2.13	6	1.12
Miscellaneous anticonvulsants	59	2.09	1	0.19
Miscellaneous unknown drug	52	1.84	12	2.23
Nonsteroidal antiinflammatory drugs	44	1.56	4	0.74
Anticonvulsants: gamma aminobutyric acid and analogs	39	1.38	1	0.19
Oral hypoglycemic	38	1.35	8	1.49
Miscellaneous chemicals	33	1.17	17	3.16
Miscellaneous anticoagulants	31	1.10	8	1.49
Miscellaneous hormones and hormone antagonists	30	1.06	4	0.74
Serotonin norepinephrine reuptake inhibitors (SNRI)	27	0.96	1	0.19
Cannabinoids and analogs	26	0.92	2	0.37
Other miscellaneous drugs	21	0.74	2	0.37

^a^Numbers represent total exposures associated with 1,218 fatalities (with relative contribution to fatality of 1-Undoubtedly responsible, 2-Probably responsible, or 3-Contributory); each fatality may have had exposure to more than one substance.

^b^Percentages are based on the total number of substances reported in fatal exposures (N = 2,822).

^c^Percentages are based on the total number of single substance fatal exposures (N = 538).

**Table 19A.** Comparisons of Death Data (1985–2013)^a.^

	**Total fatalities**		**Suicides**		**Pediatric deaths^b^**	
**Year**	**N**	**% of cases**	**N**	**% of deaths**	**N**	**% of deaths**
1985	328	0.036	174	53.0	20	6.1
1986	406	0.037	223	54.9	15	3.7
1987	398	0.034	227	57.0	22	5.5
1988	544	0.040	296	54.4	30	5.5
1989	590	0.037	323	54.7	24	4.1
1990	553	0.032	320	57.9	21	3.8
1991	764	0.042	408	53.4	44	5.8
1992	705	0.038	395	56.0	29	4.1
1993	626	0.036	338	54.0	27	4.3
1994	766	0.040	410	53.5	26	3.4
1995	724	0.036	405	55.9	20	2.8
1996	726	0.034	358	49.3	29	4.0
1997	786	0.036	418	53.2	25	3.2
1998	775	0.035	421	54.3	16	2.1
1999	873	0.040	472	54.1	24	2.7
2000	921	0.042	477	51.8	20	2.2
2001	1,085	0.048	553	51.0	27	2.5
2002	1,170	0.049	635	54.3	27	2.3
2003	1,109	0.046	592	53.4	35	3.2
2004	1,190	0.049	642	53.9	27	2.3
2005	1,438	0.059	674	46.9	32	2.2
2006	1,515	0.063	705	46.5	39	2.6
2007	1,597	0.064	737	46.1	47	2.9
2008	1,756	0.070	797	45.4	39	2.2
2009	1,544	0.062	779	50.5	37	2.4
2010	1,730	0.072	779	45.0	55	3.2
2011	2,765	0.118	865	31.3	42	1.5
2012	2,937	0.129	890	30.3	46	1.6
2013	2,477	0.113	785	31.7	51	2.1

^a^Human exposures with medical outcome of death or death, indirect regardless of RCF.

^b^Includes all children with actual or estimated ages ≤ 5 years old. Results do not include “Unknown Child” or “Unknown Age”. Includes death and death, indirect regardless of RCF.

**Table 19B.** Comparisons of Direct and Indirect Death Data (2000–2013)^a.^

	**All deaths**			**Suicides**					**Pediatric deaths**				
**Year**	**Total**	**Direct**	**Indirect**	**Total**	**% of deaths**	**Direct**	**% of direct**	**Indirect**	**Total**	**% of deaths**	**Direct**	**% of direct**	**Indirect**
2000	864	845	19	448	51.85	443	52.43	5	18	2.08	18	2.13	0
2001	1,066	952	114	542	50.84	503	52.84	39	26	2.44	24	2.52	2
2002	850	739	111	455	53.53	436	59.00	19	24	2.82	15	2.03	9
2003	867	826	41	464	53.52	454	54.96	10	29	3.34	22	2.66	7
2004	955	898	57	516	54.03	501	55.79	15	25	2.62	21	2.34	4
2005	1,423	1,332	91	666	46.80	656	49.25	10	32	2.25	26	1.95	6
2006	1,515	1,415	100	705	46.53	687	48.55	18	39	2.57	32	2.26	7
2007	1,597	1,502	95	737	46.15	712	47.40	25	47	2.94	41	2.73	6
2008	1,756	1,535	221	797	45.39	750	48.86	47	39	2.22	32	2.08	7
2009	1,544	1,452	92	779	50.45	748	51.52	31	37	2.40	31	2.13	6
2010	1,730	1,455	275	779	45.03	732	50.31	47	55	3.18	47	3.23	8
2011	2,765	1,503	1,262	865	31.28	758	50.43	107	42	1.52	31	2.06	11
2012	2,937	1,507	1,430	890	30.30	759	50.36	131	46	1.57	30	1.99	16
2013	2,477	1,552	925	785	31.69	698	44.97	87	51	2.06	43	2.77	8

^a^Human exposures with medical outcome of death or death, indirect regardless of Relative Contribution to Fatality.

**Table 20.** Frequency of Plant Exposures (Top 25)^a.^

	**Botanical name or Category**	**AAPCC Generic Code Name**	**N**
1	Plants-general-unknown	Unknown Toxic Types or Unknown if Toxic	2,347
2	Unknown Botanical Name	Unknown Toxic Types or Unknown if Toxic	2,000
3	BOTANICAL TERMS	Unknown Toxic Types or Unknown if Toxic	1,608
4	*Phytolacca americana* (L.)	Gastrointestinal Irritants (Excluding Oxalate Containing Plants)	1,190
5	*Spathiphyllum* spp.	Oxalates	981
6	Cherry (Species unspecified)	Amygdalin and/or Cyanogenic Glycosides	799
7	Plants-toxicodendrol	Skin Irritants (Excluding Oxalate Containing Plants)	786
8	*Ilex* spp (not otherwise specified)	Gastrointestinal Irritants (Excluding Oxalate Containing Plants)	772
9	Plants-cardiac glycosides	Cardiac Glycosides (Excluding Drugs)	654
10	*Philodendron* spp.	Oxalates	622
11	Plants-pokeweed	Other Toxic Types	602
12	*Caladium* spp.	Oxalates	575
13	*Malus* spp.	Amygdalin and/or Cyanogenic Glycosides	561
14	*Zantedeschia aethiopica*	Oxalates	505
15	Berry (not otherwise specified)	Unknown Toxic Types or Unknown if Toxic	481
16	*Solanum dulcamara*	Solanine	447
17	Mold (not otherwise specified)	Unknown Toxic Types or Unknown if Toxic	439
18	*Solanum nigrum*	Solanine	422
19	*Euphorbia pulcherrima* (Willd.)	Gastrointestinal Irritants (Excluding Oxalate Containing Plants)	420
20	*Narcissus pseudonarcissus* (L.)	Gastrointestinal Irritants (Excluding Oxalate Containing Plants)	410
21	*Epipremnum areum*	Oxalates	396
22	Plants-oxalates	Oxalates	382
23	Unknown Botanical Name	Non-Toxic	338
24	*Taxus canadensis*	Other Toxic Types	333
25	*Nandina domestica* (Thumb)	Amygdalin and/or Cyanogenic Glycosides	326

^a^Number of substances related to a human exposure with a major generic category of plant. Unknown Botanical Name represents substances with a major generic category of Plant and a NULL substance code. Total = 46,376

**Table 21.** Listing of Fatal Nonpharmaceutical and Pharmaceutical Exposures.

**Annual Report ID**	**Age**	**Substances**	**Substance Rank**	**Cause Rank**	**Chronicity**	**Route**	**Reason**	**RCF**	**Analyte**	**Blood Concentration @ Time**
**Non-Pharmaceutical Exposures**										
**Alcohols**										
[1ha]	17 y F				A	Unk	Int-S	1		
		methanol	1	1					methanol	45 mg/dL In Unknown @ 20 h (pe)
2ai	18 y F				U	Ingst	Int-A	2		
		ethanol	1	1						
3ai	20 y M				U	Ingst	Int-A	2		
		ethanol	1	1						
4ai	21 y M				U	Ingst	Int-A	2		
		ethanol	1	1						
5ai	22 y M				U	Ingst+ Par	Int-A	2		
		ethanol	1	1						
		heroin	2	2						
		oxycodone	3	3						
		oxymorphone	4	4						
6ai	24 y M				A	Ingst+ Unk	Int-A	2		
		ethanol (non-beverage)	1	1						
		cocaine	2	2						
7ai	25 y M				U	Ingst	Int-A	2		
		ethanol	1	1						
8ai	26 y M				A	Ingst	Int-A	2		
		ethanol (non-beverage)	1	1						
		diphenhydramine	2	2						
		doxylamine	3	3						
9ai	26 y M				A	Ingst	Int-A	2		
		ethanol (non-beverage)	1	1						
		oxycodone	2	2						
		doxylamine	3	3						
10ai	26 y M				A	Ingst	Int-A	2		
		ethanol	1	1						
11ai	26 y F				U	Ingst+ Unk	Int-S	2		
		ethanol	1	1						
		methamphetamine	2	2						
12ai	26 y M				U	Ingst	Int-S	2		
		ethanol	1	1						
13ai	26 y M				U	Ingst	Unk	2		
		ethanol	1	1						
14p	26 y F				A	Ingst	Int-A	2		
		ethanol	1	1					ethanol	106 mg/dL In Serum @ 30 m (pe)
		escitalopram	2	2						
		methocarbamol	3	3						
		oxycodone	4	4						
		lorazepam	5	5						
15ai	27 y M				U	Ingst	Int-A	2		
		ethanol	1	1						
16ai	28 y F				U	Ingst	Int-A	2		
		ethanol	1	1						
17ai	28 y M				A	Ingst	Int-A	2		
		ethanol	1	1						
18ai	29 y M				A	Ingst	Int-A	2		
		ethanol	1	1						
		diazepam	2	2						
		fluoxetine	3	3						
19ai	29 y F				A	Ingst	Int-A	2		
		ethanol	1	1						
20ai	30 y M				U	Ingst+ Aspir	Int-A	2		
		alcohol, unknown	1	1						
		zolpidem	2	2						
		diazepam	3	3						
21ai	30 y M				U	Ingst	Int-A	2		
		ethanol	1	1						
22ai	30 y M				A	Ingst	Int-S	2		
		methanol	1	1						
		clonazepam	2	2						
		diphenhydramine	3	3						
		bupropion	4	4						
23	30 y F				A	Unk	Unk	2		
		methanol	1	1						
24	30 y M				A	Ingst	Int-S	1		
		methanol	1	1					methanol	253 mg/dL In Blood (unspecified) @ Unknown
25ai	31 y M				U	Ingst	Int-A	2		
		ethanol	1	1						
26ai	31 y M				U	Ingst	Int-A	2		
		ethanol	1	1						
27ha	31 y M				U	Ingst	Oth-W	3		
		isopropanol	1	1					ethanol	12 mg/dL In Urine (quantitative only) @ Unknown
28ai	32 y M				U	Ingst	Int-S	2		
		ethanol	1	1						
		methamphetamine	2	2						
29ai	33 y M				A	Ingst	Int-A	2		
		ethanol	1	1						
30ai	34 y M				U	Ingst	Int-A	2		
		ethanol	1	1						
31h	35 y F				C	Ingst	Unk	2		
		ethanol	1	1						
32ai	35 y F				U	Ingst	Int-A	2		
		ethanol	1	1						
		zolpidem	2	2						
33ai	35 y M				A	Ingst	Int-A	2		
		ethanol (non-beverage)	1	1						
		citalopram	2	2						
		diphenhydramine	3	3						
		buprenorphine	4	4						
		clonazepam	5	5						
34ai	35 y M				U	Ingst	Int-S	2		
		ethanol	1	1						
35ai	35 y M				A	Ingst	Int-A	2		
		ethanol	1	1						
36ai	36 y M				U	Ingst	Int-S	2		
		ethanol	1	1						
37ai	36 y F				U	Ingst	Int-A	2		
		ethanol	1	1						
38ai	37 y M				C	Ingst	Int-A	2		
		ethanol	1	1						
		diphenhydramine	2	2						
		doxylamine	3	3						
		acetone	4	4						
39ai	37 y M				U	Ingst	Int-A	2		
		ethanol	1	1						
		acetaminophen/hydrocodone	2	2						
		alprazolam	3	3						
40pha	37 y M				U	Ingst+ Unk	Int-U	1		
		ethanol	1	1						
		opioid	2	2						
41	37 y M				A	Ingst	Int-S	1		
		ethanol	1	1					ethanol	11 mg/dL In Blood (unspecified) @ Unknown
		acetaminophen	2	2					acetaminophen	13 mcg/mL In Blood (unspecified) @ 22 h (pe)
		acetaminophen	2	2					acetaminophen	47 mcg/mL In Blood (unspecified) @ Unknown
42ai	38 y M				A	Ingst	Int-A	2		
		ethanol	1	1						
43ai	38 y F				A	Ingst+ Unk	Int-A	2		
		ethanol	1	1						
		cocaine	2	2						
		hydrocodone	3	3						
		cyclobenzaprine	4	4						
		promethazine	5	5						
		doxylamine	6	6						
		acetaminophen	7	7						
44ai	38 y M				U	Ingst	Int-S	2		
		ethanol	1	1						
45ai	38 y M				U	Ingst	Oth-M	2		
		ethanol	1	1						
46ai	38 y M				A	Ingst	Int-A	2		
		ethanol	1	1						
47ai	39 y M				U	Ingst	Int-A	2		
		ethanol	1	1						
48	39 y M				C	Ingst	Int-A	3		
		ethanol	1	1						
		acetaminophen/hydrocodone	2	2						
		amitriptyline	3	3						
		atenolol	4	4						
		levetiracetam	5	5						
49ai	40 y M				A	Ingst+ Unk	Int-A	2		
		ethanol (non-beverage)	1	1						
		heroin	2	2						
		benzodiazepine	3	3						
50ai	40 y M				A	Ingst	Int-A	2		
		ethanol	1	1						
		diazepam	2	2						
51ai	40 y M				U	Ingst	Int-A	2		
		ethanol	1	1						
		acetaminophen/hydrocodone	2	2						
52ai	40 y M				U	Ingst	Int-A	2		
		ethanol	1	1						
53ai	41 y M				A	Ingst	Int-A	2		
		ethanol	1	1						
54ai	41 y M				A	Ingst	Int-A	2		
		ethanol	1	1						
55ai	41 y M				U	Ingst	Int-A	2		
		ethanol	1	1						
56ai	41 y M				U	Ingst	Int-A	2		
		ethanol	1	1						
57	41 y M				C	Ingst	Int-A	3		
		ethanol	1	1						
58pha	42 y M				C	Unk	Int-A	3		
		ethanol	1	1						
59ai	42 y M				A	Ingst	Int-A	2		
		ethanol	1	1						
60ai	42 y M				U	Ingst	Int-A	2		
		ethanol	1	1						
61ai	42 y M				A	Ingst	Int-A	2		
		ethanol	1	1						
62ai	42 y M				U	Ingst	Int-A	2		
		ethanol	1	1						
63	42 y M				A	Ingst	Int-S	1		
		methanol	1	1						
64ai	43 y M				U	Ingst	Int-S	2		
		ethanol	1	1						
65ai	43 y M				U	Ingst	Int-A	2		
		ethanol	1	1						
66ai	44 y F				A	Ingst	Int-A	2		
		ethanol	1	1						
67ai	44 y F				A	Ingst	Unt-G	2		
		ethanol	1	1						
		citalopram	2	2						
		diphenhydramine	3	3						
68ai	44 y M				A	Ingst	Int-A	2		
		ethanol	1	1						
69p	44 y F				U	Ingst	Int-U	3		
		ethanol	1	1					ethanol	157 mg/dL In Blood (unspecified) @ Unknown
		methanol	2	2						
70pha	44 y F				A	Unk	Int-A	1		
		ethanol	1	1					ethanol	0.24 mg/dL In Serum @ 1 h (pe)
		heroin	2	2					morphine (free)	0.088 mg/L In Serum @ 1 h (pe)
		drug, unknown	3	3						
71a	45 y M				C	Ingst	Unk	3		
		ethanol	1	1						
		dextromethorphan	2	2					dextromethorphan	72 ng/mL In Serum @ Unknown
72ai	46 y M				C	Ingst	Int-A	2		
		ethanol	1	1						
73ai	46 y M				A	Ingst	Int-A	2		
		ethanol	1	1						
74ai	46 y F				A	Ingst	Int-A	2		
		ethanol	1	1						
75h	47 y M				A	Ingst	Int-S	1		
		methanol	1	1					methanol	269 mg/dL In Whole Blood @ Unknown
76ai	47 y M				U	Ingst	Int-S	2		
		ethanol	1	1						
		acetaminophen/hydrocodone	2	2						
		oxycodone	3	3						
		alprazolam	4	4						
77ai	48 y M				A	Ingst	Int-A	2		
		ethanol	1	1						
78	48 y M				A	Ingst	Int-A	1		
		methanol	1	1					methanol	300 mg/dL In Serum @ Unknown
79ai	48 y F				A	Ingst	Int-A	2		
		ethanol	1	1						
80h	48 y M				U	Ingst	Int-M	3		
		ethanol	1	1						
81ai	48 y M				A	Ingst	Int-A	2		
		ethanol (non-beverage)	1	1						
		citalopram	2	2						
		dextromethorphan	3	3						
		doxylamine	4	4						
		diphenhydramine	5	5						
82ai	49 y M				A	Ingst	Int-A	2		
		ethanol	1	1						
83ai	49 y M				A	Ingst	Int-A	2		
		ethanol	1	1						
84h	49 y M				U	Ingst	Unk	3		
		ethanol	1	1					ethanol	15 mg/dL In Blood (unspecified) @ 1 h (pe)
		amitriptyline	2	2						
		hydrochlorothiazide/metoprolol	3	3						
		paroxetine	4	4						
		lisinopril	5	5						
		disulfiram	6	6						
		salicylate	7	7						
		insulin	8	8						
85ai	50 y M				A	Ingst	Int-A	2		
		ethanol	1	1						
86ai	50 y M				A	Ingst	Int-A	2		
		ethanol	1	1						
87ai	50 y M				A	Ingst	Int-A	2		
		ethanol (non-beverage)	1	1						
		lamotrigine	2	2						
		amlodipine	3	3						
		diphenhydramine	4	4						
88	51 y M				A	Ingst	Int-A	2		
		ethanol	1	1						
		acetaminophen	2	2					acetaminophen	18.9 mg/L In Serum @ 0.5 m (pe)
89ai	51 y M				A	Ingst	Int-A	2		
		ethanol	1	1						
90ai	52 y F				A	Ingst	Int-A	2		
		ethanol	1	1						
		methadone	2	2						
91ai	52 y M				U	Ingst	Int-A	2		
		ethanol	1	1						
92h	52 y M				U	Ingst	Int-S	3		
		ethanol	1	1					ethanol	407 mg/dL In Serum @ Unknown
		methadone	2	2						
		acetone	3	3					acetone	4.2 mg/dL In Serum @ Unknown
		methanol	4	4					methanol	3.1 mg/dL In Serum @ Unknown
		isopropanol	5	5					isopropanol	4.3 mg/dL In Plasma @ Unknown
93ai	53 y M				A	Ingst	Int-A	2		
		ethanol	1	1						
94h	53 y F				C	Ingst	Oth-W	3		
		ethanol	1	1						
95ai	53 y M				A	Ingst	Int-A	2		
		ethanol (non-beverage)	1	1						
		diphenhydramine	2	2						
		doxylamine	3	3						
		dextromethorphan	4	4						
96h	53 y M				A	Ingst	Int-U	1		
		methanol	1	1					methanol	380 mg/dL In Blood (unspecified) @ Unknown
		methanol	1	1					methanol	47 mg/dL In Blood (unspecified) @ Unknown
97	54 y M				A	Ingst	Int-S	3		
		ethanol	1	1					ethanol	400 mg/dL In Serum @ Unknown
		ethylene glycol (antifreeze)	2	2						
98ai	54 y M				U	Ingst	Int-A	2		
		ethanol	1	1						
99ai	54 y F				U	Ingst	Int-A	2		
		ethanol	1	1						
100ai	54 y M				A	Ingst	Int-A	2		
		ethanol	1	1						
		cyclobenzaprine	2	2						
101h	54 y M				A	Ingst	Int-S	3		
		ethanol	1	1						
		laundry detergent	2	2						
102ai	55 y M				A	Ingst	Int-A	2		
		ethanol (non-beverage)	1	1						
		verapamil	2	2						
		acetaminophen	3	3						
103ai	55 y M				U	Ingst	Int-A	2		
		ethanol	1	1						
104ai	55 y M				A	Ingst	Unt-G	2		
		ethanol	1	1						
105ai	55 y F				A	Ingst	Int-A	2		
		ethanol	1	1						
		morphine	2	2						
		diazepam	3	3						
		citalopram	4	4						
106ai	55 y M				U	Ingst	Int-A	2		
		ethanol	1	1						
107ai	55 y M				U	Ingst	Int-A	2		
		ethanol	1	1						
108	56 y M				U	Ingst	Unk	3		
		ethanol	1	1						
		isopropanol	2	2						
109ai	56 y M				A	Ingst	Int-A	2		
		ethanol	1	1						
110p	56 y M				A	Ingst	Int-A	2		
		ethanol	1	1					ethanol	50 mg/dL In Blood (unspecified) @ 1 h (pe)
		oxycodone	2	2					acetaminophen	10 mcg/mL In Blood (unspecified) @ 1 h (pe)
111ai	56 y M				A	Ingst	Int-A	2		
		ethanol (non-beverage)	1	1						
		oxycodone	2	2						
		citalopram	3	3						
		metoprolol	4	4						
112ai	56 y F				A	Ingst	Int-A	2		
		ethanol	1	1						
		trazodone	2	2						
		fluoxetine	3	3						
113ai	56 y M				A	Ingst	Int-A	2		
		ethanol	1	1						
114ai	56 y M				A	Ingst	Int-A	2		
		ethanol (non-beverage)	1	1						
		amitriptyline	2	2						
115	56 y M				U	Ingst	Unk	2		
		methanol	1	1						
		ethanol	2	2					ethanol	172 mg/dL In Blood (unspecified) @ Unknown
116ai	57 y M				A	Ingst	Int-A	2		
		ethanol (non-beverage)	1	1						
		diphenhydramine	2	2						
		oxycodone	3	3						
117ai	57 y M				U	Ingst	Int-A	2		
		ethanol	1	1						
		carbon monoxide	2	2						
		smoke	3	3						
118ai	58 y M				A	Ingst	Int-A	2		
		ethanol	1	1						
		chlordiazepoxide	2	2						
119ai	58 y M				A	Ingst	Int-A	2		
		ethanol	1	1						
120ai	58 y F				A	Ingst	Int-A	2		
		ethanol	1	1						
		acetaminophen	2	2						
		diphenhydramine	3	3						
121ph	58 y M				U	Ingst	Int-U	3		
		ethanol	1	1					ethanol	369 mg/dL In Serum @ Unknown
		metformin	2	2						
122pa	59 y F				U	Ingst	Int-U	2		
		ethanol	1	1					ethanol	236 mg/dL In Blood (unspecified) @ Unknown
		temazepam	2	2					temazepam	0.69 mg/L In Plasma @ Unknown
		clonazepam	3	3					clonazepam	13 ng/mL In Blood (unspecified) @ Unknown
		risperidone	4	4					risperidone	12 ng/mL In Plasma @ Unknown
123ai	60 y F				A	Ingst	Int-A	2		
		ethanol (non-beverage)	1	1						
		tramadol	2	2						
124	60 y M				U	Ingst+ Aspir	Int-S	3		
		ethanol (non-beverage)	1	1						
		acetaminophen	2	2						
125ai	62 y M				A	Ingst	Int-A	2		
		ethanol	1	1						
126ai	62 y M				A	Ingst	Int-A	2		
		ethanol	1	1						
127ai	63 y M				A	Ingst	Int-A	2		
		ethanol	1	1						
128ai	64 y M				A	Ingst	Int-A	2		
		ethanol (non-beverage)	1	1						
		diazepam	2	2						
		trazodone	3	3						
129ai	65 y M				C	Ingst	Int-A	2		
		ethanol	1	1						
130ai	67 y M				C	Ingst	Int-A	2		
		ethanol	1	1						
131ai	67 y F				A	Ingst	Int-A	2		
		ethanol	1	1						
132ai	69 y M				C	Ingst	Int-A	2		
		ethanol	1	1						
133ai	69 y F				U	Ingst	Int-A	2		
		ethanol	1	1						
134ai	75 y M				U	Ingst	Int-A	2		
		ethanol	1	1						
135ai	75 y M				A	Ingst	Int-A	2		
		ethanol	1	1						
136ph	82 y F				A	Ingst	Int-S	2		
		isopropanol	1	1						
See Also case 137, 162, 163, 166, 192, 232, 233, 240, 251, 253, 265, 270, 272, 275, 276, 281, 285, 287, 294, 297, 301, 315, 342, 390, 406, 407, 426, 438, 445, 447, 460, 469, 497, 509, 514, 527, 535, 558, 559, 560, 570, 573, 580, 584, 595, 626, 629, 631, 641, 652, 668, 674, 678, 683, 684, 685, 687, 691, 698, 702, 711, 715, 731, 744, 745, 746, 747, 749, 752, 755, 759, 761, 762, 772, 778, 785, 794, 804, 814, 816, 818, 821, 825, 829, 830, 836, 844, 854, 858, 859, 861, 867, 870, 871, 874, 878, 881, 888, 889, 890, 892, 895, 906, 907, 912, 913, 917, 918, 920, 931, 936, 940, 954, 959, 975, 981, 989, 994, 995, 1001, 1009, 1011, 1012, 1013, 1015, 1028, 1051, 1052, 1055, 1056, 1061, 1082, 1092, 1104, 1108, 1130, 1146, 1153, 1155, 1164, 1166, 1169, 1179, 1184, 1187, 1192, 1198, 1207, 1216, 1217, 1226, 1229, 1232, 1234, 1237, 1240, 1246, 1286, 1291, 1293, 1296, 1297, 1300, 1308, 1316, 1319, 1323, 1331, 1335, 1341, 1347, 1349, 1351, 1359, 1368, 1376, 1382, 1383, 1385, 1394, 1397, 1402, 1404, 1406, 1410, 1415, 1427, 1434, 1439, 1483, 1494, 1497, 1503, 1513, 1516, 1520, 1539, 1548, 1549, 1554, 1566, 1572, 1575, 1579, 1580, 1587, 1589, 1596, 1609, 1613, 1621, 1632, 1640, 1641, 1642, 1661, 1675, 1680, 1684, 1701, 1719, 1721, 1723, 1728, 1734, 1735, 1737, 1747, 1762, 1764, 1789, 1791, 1792, 1795, 1800, 1805, 1806, 1808, 1810, 1815, 1817, 1819, 1827, 1848, 1849, 1852, 1854, 1858, 1865, 1870, 1883, 1892, 1895, 1897, 1898, 1901, 1903, 1904, 1908, 1910, 1912, 1919, 1923, 1925, 1926, 1932, 1935, 1937, 1939, 1943, 1944, 1945, 1953, 1954, 1956, 1958, 1962, 1968, 1971, 1973, 1979, 1984, 1986, 1987, 1988, 1991, 1992, 1993, 1998, 2003, 2008, 2011, 2012, 2013, 2015, 2018, 2025, 2029, 2030, 2032, 2033, 2036, 2037, 2038, 2040, 2044, 2047, 2052, 2056, 2065, 2066, 2067, 2068, 2070, 2093, 2098, 2106										
**Automotive/Aircraft/Boat Products**										
137pi	21 y M				A	Ingst+ Par	Int-A	3		
		ethylene glycol (antifreeze)	1	1						
		ethanol	2	2					ethanol	421 mg/dL In Blood (unspecified) @ Unknown
138p	25 y M				A	Ingst	Int-S	1		
		ethylene glycol (antifreeze)	1	1					ethylene glycol	27 mg/dL In Serum @ Unknown
139i	30 y F				A	Ingst	Int-S	2		
		ethylene glycol/diethylene glycol	1	1						
140h	30 y M				A	Ingst	Int-S	1		
		ethylene glycol (antifreeze)	1	1					ethylene glycol	85 mg/dL In Unknown @ Unknown
141ph	33 y M				A	Ingst	Int-S	1		
		ethylene glycol (antifreeze)	1	1					ethylene glycol	194.8 mg/dL In Blood (unspecified) @ Unknown
142h	42 y M				A/C	Ingst	Int-S	1		
		ethylene glycol (antifreeze)	1	1						
		lithium	2	2						
		lamotrigine	3	3						
		ziprasidone	4	4						
		levothyroxine	5	5						
143h	46 y M				A	Ingst	Unk	2		
		ethylene glycol (antifreeze)	1	1						
144h	58 y M				A	Ingst	Int-S	1		
		ethylene glycol (antifreeze)	1	1						
145	61 y F				A	Ingst	Int-S	1		
		methanol	1	1					methanol	144 mg/dL In Blood (unspecified) @ Unknown
146	61 y M				A	Ingst	Int-S	1		
		ethylene glycol (antifreeze)	1	1						
147h	62 y M				A	Ingst	Int-S	1		
		ethylene glycol (antifreeze)	1	1						
		hypochlorite	2	2						
		cleaner (household)	3	3						
		ethanol (non-beverage)	4	4						
[148a]	66 y M				A	Oth	Int-S	1		
		ethylene glycol (antifreeze)	1	1					ethylene glycol	1200 mg/dL In Whole Blood @ 6 h (pe)
149h	20 + y M				A	Ingst	Int-S	2		
		brake fluid	1	1						
See Also case 80, 196										
**Batteries**										
150i	2 y M				A	Ingst	Unt-G	1		
		disc battery	1	1						
151	4 y M				A	Ingst	Unt-G	2		
		battery	1	1						
152	4 y F				A	Ingst	Unt-G	2		
		disc battery, lithium	1	1						
[153]	16 m M				A	Ingst	Unt-G	1		
		disc battery	1	1						
See Also case 1484										
**Bites and Envenomations**										
[154h]	3 y M				A	B-S	Unt-B	1		
		sting (scorpion)	1	1						
[155p]	53 y M				A	B-S	Unt-B	1		
		envenomation (crotalid)	1	1						
156p	61 y M				A	Unk	Unt-O	1		
		sting (hymenoptera)	1	1						
		substance (non-drug), unknown	2	2						
		pyrethroids	3	3						
		insecticide (neonicotinoid)	4	4						
		pyrethroids	5	5						
		pyrethroids	6	6						
		pyrethroids	7	7						
157h	62 y M				A	B-S	Unt-B	3		
		sting (hymenoptera)	1	1						
158ph	80 y M				A	B-S	Unt-B	3		
		envenomation (crotalid)	1	1						
See Also case 1897										
**Chemicals**										
159p	18 y M				A	Ingst	Int-S	1		
		cyanide	1	1						
160pa	19 y M				A	Ingst	Int-S	1		
		cyanide	1	1					cyanide	10 mcg/mL In Blood (unspecified) @ Autopsy
[161ha]	19 y M				A	Unk	Int-S	1		
		cyanide	1	1					cyanide	1.3 mg/L In Unknown @ Unknown
		cyanide	1	1					cyanide	10 mcg/mL In Unknown @ Unknown
162	22 y M				A	Ingst	Unt-O	2		
		hydrochloric acid	1	1						
		ethanol	2	2						
		methamphetamine	3	3						
		marijuana	4	4						
163ai	22 y M				U	Ingst	Int-A	2		
		vinyldene chloride	1	1						
		ethanol	2	2						
		chlorpheniramine	3	3						
		dextromethorphan	4	4						
		sertraline	5	5						
164	22 y M				A	Ingst	Int-A	2		
		lysergic acid diethylamide (LSD)	1	1						
165ph	23 y M				A	Derm	Unt-O	3		
		ammonia	1	1						
166ph	23 y M				A	Ingst	Int-S	1		
		cyanide	1	1					cyanide	112 ng/mL In Blood (unspecified) @ 18 h (pe)
		ethanol	2	2					ethanol	340 mg/dL In Serum @ Unknown
167p	27 y M				A	Ingst	Int-S	1		
		cyanide	1	1						
168p	35 y M				A	Ingst	Int-S	1		
		cyanide	1	1						
169ha	36 y M				A	Ingst+ Derm	Int-S	1		
		hydrochloric acid	1	1						
170h	36 y M				A	Ingst	Int-S	2		
		ethylene glycol (antifreeze)	1	1					ethylene glycol	0 Other (see abst) In Plasma @ Unknown
[171h]	45 y M				A	Inhal+ Oc	Unt-O	3		
		ammonia	1	1						
172h	46 y M				A	Ingst	Int-S	1		
		ethylene glycol (antifreeze)	1	1					ethylene glycol	178 mg/dL In Serum @ 1 h (pe)
173	47 y M				A	Ingst	Int-S	2		
		ethylene glycol (antifreeze)	1	1						
		diazepam	2	2						
174phi	48 y M				A	Ingst	Unk	1		
		cyanide	1	1						
175a	54 y F				U	Ingst	Int-S	1		
		drug, unknown *	2	1						
		ethylene glycol (antifreeze) *	1	1						
176a	57 y M				A	Ingst	Int-S	1		
		ethylene glycol (antifreeze)	1	1					ethylene glycol	24 mcg/dL In Serum @ 30 m (pe)
177	61 y M				A	Ingst	Int-A	1		
		ethylene glycol (antifreeze)	1	1						
178h	61 y F				A/C	Ingst+ Aspir	Int-S	3		
		lithium	1	1					lithium	4.3 mmol/L In Blood (unspecified) @ 4 h (pe)
		lithium	1	1					lithium	5 mmol/L In Blood (unspecified) @ 10 h (pe)
		lithium	1	1					lithium	5.9 mmol/L In Blood (unspecified) @ 61 h (pe)
		lithium	1	1					lithium	6.5 mmol/L In Blood (unspecified) @ 37 h (pe)
		lithium	1	1					lithium	6.9 mmol/L In Blood (unspecified) @ 27 h (pe)
		lithium	1	1					lithium	7.4 mmol/L In Blood (unspecified) @ 17 h (pe)
		clonazepam	2	2						
179	63 y M				A	Ingst	Int-S	2		
		ethylene glycol (antifreeze)	1	1						
		drug, unknown	2	2						
180h	63 y M				A	Oth	AR-O	3		
		cobalt	1	1						
		chromium	2	2						
181	64 y M				A	Ingst	Int-M	1		
		chemical, unknown	1	1						
182	65 y M				A	Ingst	Int-S	1		
		ethylene glycol (antifreeze)	1	1						
183h	66 y M				U	Ingst	Int-U	2		
		corrosive (alkali)	1	1						
		acetaminophen	2	2					acetaminophen	21 mcg/mL In Blood (unspecified) @ 2 d (pe)
184	68 y M				A	Ingst	Int-S	1		
		ethylene glycol (antifreeze)	1	1					ethylene glycol	108 mcg/mL In Serum @ Unknown
[185ha]	73 y M				A	Ingst	Int-S	1		
		cyanide	1	1						
[186]	78 y M				A	Par	Unt-T	1		
		Potassium aluminum sulfate	1	1						
187ai	80 y M				A	Inhal	Unt-E	2		
		methylene chloride	1	1						
		citalopram	2	2						
188h	86 y M				A	Ingst	Int-S	1		
		hydrochloric acid	1	1						
189pi	Unknown adult (> = 20 yrs) U				A	Inhal	Unt-G	2		
		cyanide	1	1						
See Also case 38, 92, 97, 208, 241, 267, 271, 281, 292, 525, 1802, 1924										
**Cleaning Substances (Household)**										
190p	22 y F				A	Ingst	Int-S	2		
		hypochlorite	1	1						
		clonazepam	2	2						
		metoprolol	3	3						
191p	29 y M				A/C	Unk	Unk	3		
		toilet bowl cleaner	1	1						
		bupropion	2	2					hydroxybupropion	1700 ng/mL In Blood (unspecified) @ Autopsy
		bupropion	2	2					bupropion	470 ng/mL In Blood (unspecified) @ Autopsy
		fluoxetine	3	3					norfluoxetine	560 ng/mL In Blood (unspecified) @ Autopsy
		fluoxetine	3	3					fluoxetine	870 ng/mL In Blood (unspecified) @ Autopsy
192h	49 y M				A	Ingst	Int-S	2		
		hydrofluoric acid	1	1						
		ethanol	2	2						
193pha	49 y M				A	Ingst	Unt-G	2		
		disinfectant (isopropanol/pine oil)	1	1						
		morphine	2	2					morphine	0.09 mg/L In Blood (unspecified) @ Autopsy
194h	50 y M				A	Ingst	Unk	3		
		cleaner (anionic/nonionic)	1	1						
		disinfectant (phenol)	2	2						
195	52 y M				A	Ingst	Int-S	1		
		drain cleaner (sulfuric acid)	1	1						
196	52 y M				A	Ingst	Int-S	1		
		enzyme detergents	1	1						
		ethylene glycol (antifreeze)	2	2						
197h	56 y M				A	Ingst	Int-S	2		
		hydrofluoric acid	1	1						
198ha	61 y F				U	Ingst	Int-S	2		
		cleaner (household)	1	1						
[199ph]	63 y M				A	Par	Unt-T	2		
		hypochlorite	1	1						
200	65 y F				A	Ingst	Int-S	3		
		cleaner (anionic/nonionic)	1	1						
201p	71 y F				A	Inhal	Unt-E	3		
		drain cleaner (alkali)	1	1						
		chlorine gas	2	2						
202h	81 y F				A	Ingst	Unt-G	2		
		drain cleaner (alkali)	1	1						
203a	86 y M				A	Ingst	Unt-G	1		
		drain cleaner (alkali)	1	1						
204h	87 y F				A	Ingst	Int-S	1		
		hypochlorite	1	1						
205	90 y M				A	Ingst	Unt-G	2		
		chlorhexidine	1	1						
[206ha]	7 m M				A	Ingst	Unt-G	1		
		laundry detergent (pod)	1	1						
207	40 + y M				A	Inhal	Int-A	2		
		cleaner (household)	1	1						
208p	Unknown age U				A	Inhal	Int-S	2		
		hydrogen sulfide *	1	1						
		toilet bowl cleaner (acid) *	2	1						
		sulfur	3	2						
See Also case 101, 147, 366										
**Foreign Bodies/Toys/Miscellaneous**										
[209pha]	19 m F				A	Ingst	Unk	1		
		magnets	1	1						
		carbaryl	2	2						
										
**Fumes/Gases/Vapors**										
210pa	1 y F				A	Inhal	Unt-E	1		
		smoke	1	1					carboxyhemoglobin	60 % In Blood (unspecified) @ Autopsy
211pa	2 y M				A	Inhal	Unt-E	1		
		smoke	1	1					carboxyhemoglobin	60 % In Blood (unspecified) @ Autopsy
212pa	3 y F				A	Inhal	Unt-E	1		
		smoke	1	1					carboxyhemoglobin	54 % In Blood (unspecified) @ Autopsy
213ai	3 y F				A	Inhal	Unt-E	2		
		smoke	1	1						
		carbon monoxide	2	2						
214pa	3 y M				A	Inhal	Unt-E	1		
		smoke	1	1					carboxyhemoglobin	23 % In Blood (unspecified) @ Autopsy
215ai	4 y F				A	Inhal	Unt-E	2		
		smoke	1	1						
		carbon monoxide	2	2						
216pa	4 y M				A	Inhal	Unt-E	1		
		smoke	1	1					carboxyhemoglobin	60 % In Blood (unspecified) @ Autopsy
217pa	5 y M				A	Inhal	Unt-E	1		
		smoke	1	1					carboxyhemoglobin	60 % In Blood (unspecified) @ Autopsy
218ai	6 y F				A	Inhal	Unt-E	2		
		smoke	1	1						
		carbon monoxide	2	2						
219pa	6 y F				A	Inhal	Unt-E	1		
		smoke	1	1					carboxyhemoglobin	60 % In Blood (unspecified) @ Autopsy
220ai	8 y F				A	Inhal	Unt-E	2		
		smoke	1	1						
		carbon monoxide	2	2						
221pi	8 y M				A	Inhal	Unt-E	1		
		smoke	1	1						
222pi	9 y F				A	Inhal	Unt-E	1		
		smoke	1	1						
223ai	10 y F				A	Inhal	Unt-E	2		
		smoke	1	1						
		carbon monoxide	2	2						
[224pa]	11 y M				A	Inhal	Unt-E	1		
		carbon monoxide	1	1					carboxyhemoglobin	50 % In Blood (unspecified) @ Autopsy
225pa	11 y M				A	Inhal	Unt-E	1		
		smoke	1	1					carboxyhemoglobin	60 % In Blood (unspecified) @ Autopsy
226p	12 y M				A	Inhal	Unt-E	1		
		carbon monoxide	1	1						
227pha	16 y F				A	Inhal	Unt-E	1		
		carbon monoxide	1	1					carboxyhemoglobin	48.7 % In Blood (unspecified) @ 30 m (pe)
		smoke	2	2						
228ai	16 y M				A	Ingst+ Inhal	Int-S	2		
		carbon monoxide	1	1						
		citalopram	2	2						
229pha	20 y F				A	Inhal	Unt-E	1		
		smoke	1	1					carboxyhemoglobin	60 % In Blood (unspecified) @ Unknown
		carbon monoxide	2	2						
230pha	21 y M				A	Inhal	Unt-E	1		
		smoke	1	1					carboxyhemoglobin	58 % In Blood (unspecified) @ Unknown
		carbon monoxide	2	2						
231p	21 y M				A	Inhal	Int-S	1		
		helium	1	1						
232ai	22 y F				A	Ingst+ Inhal	Int-S	2		
		carbon monoxide	1	1						
		diphenhydramine	2	2						
		ethanol	3	3						
233pa	23 y M				A	Inhal	Unt-E	1		
		smoke	1	1					carboxyhemoglobin	60 % In Blood (unspecified) @ Autopsy
		ethanol	2	2					ethanol	180 mg/dL In Blood (unspecified) @ Autopsy
234pi	23 y M				A	Inhal	Unt-E	1		
		carbon monoxide	1	1						
235	24 y F				A	Ingst	Unt-E	1		
		carbon monoxide	1	1					carboxyhemoglobin	50 % In Whole Blood @ Unknown
		carbon monoxide	2	2						
		ketamine	3	3						
		methamphetamine	4	4						
236ai	24 y F				U	Inhal	Int-S	2		
		hydrogen sulfide	1	1						
237p	24 y M				A	Inhal	Unt-O	1		
		hydrogen sulfide	1	1						
238ai	25 y M				A	Inhal	Unt-O	2		
		smoke	1	1						
		carbon monoxide	2	2						
239pa	26 y M				A	Ingst	Int-S	1		
		hydrogen sulfide	1	1					thiosulfate	160 mcg/mL In Plasma @ 10 m (pe)
		glyphosate	2	2						
240ph	26 y M				A	Ingst+ Inhal	Unk	1		
		carbon monoxide	1	1					methemoglobin	11 % In Blood (unspecified) @ Unknown
		carbon monoxide	1	1					carboxyhemoglobin	34.2 % In Blood (unspecified) @ Unknown
		smoke	2	2						
		ethanol	3	3						
		marijuana	4	4						
241p	27 y F				A	Inhal	Unt-E	1		
		carbon monoxide *	1	1						
		cyanide *	2	1						
242ph	27 y M				A	Inhal	Unt-G	3		
		carbon monoxide	1	1						
243	28 y F				A	Ingst+ Inhal	Int-S	1		
		carbon monoxide	1	1					carboxyhemoglobin	20.6 % In Whole Blood @ Unknown
		acetaminophen/hydrocodone	2	2						
		alprazolam	3	3						
244phai	28 y F				A	Inhal	Unt-E	1		
		carbon monoxide	1	1					carboxyhemoglobin	45 % In Whole Blood @ Unknown
		smoke	2	2						
		caffeine	3	3					caffeine	1 Other (see abst) In Blood (unspecified) @ Autopsy
		caffeine	3	3					caffeine	1 Other (see abst) In Urine (quantitative only) @ Autopsy
		cotinine	4	4						
		sertraline	5	5					sertraline	0.13 mcg/mL In Blood (unspecified) @ Autopsy
		sertraline	5	5					norsertraline	1 Other (see abst) In Blood (unspecified) @ Autopsy
		sertraline	5	5					sertraline	1 Other (see abst) In Urine (quantitative only) @ Autopsy
		lidocaine	6	6						
		amitriptyline	7	7					amitriptyline	1 Other (see abst) In Urine (quantitative only) @ Autopsy
		metoprolol	8	8					metoprolol	1 Other (see abst) In Urine (quantitative only) @ Autopsy
245pi	30 y M				A	Inhal	Unt-E	1		
		carbon monoxide	1	1						
246pi	30 y M				A	Inhal	Unt-E	1		
		carbon monoxide	1	1						
247ai	32 y F				A	Inhal	Oth-M	2		
		smoke	1	1						
		carbon monoxide	2	2						
248pha	33 y M				A	Inhal	Unk	1		
		hydrogen sulfide	1	1					thiosulfate	6.1 mg/L In Plasma @ Autopsy
249p	34 y M				A	Inhal	Unt-E	1		
		carbon monoxide	1	1						
		smoke	2	2						
250p	34 y F				A	Inhal	Int-S	1		
		smoke	1	1						
251ai	35 y M				A	Ingst+ Inhal	Unt-E	2		
		smoke	1	1						
		carbon monoxide	2	2						
		ethanol (non-beverage)	3	3						
		diphenhydramine	4	4						
252pi	35 y M				A	Inhal	Unt-E	1		
		carbon monoxide	1	1						
253ai	37 y M				A	Ingst+ Inhal	Unt-E	2		
		smoke	1	1						
		carbon monoxide	2	2						
		oxycodone	3	3						
		alprazolam	4	4						
		fluoxetine	5	5						
		hydrocodone	6	6						
		acetaminophen	7	7						
		ethanol	8	8						
254pi	37 y F				A	Inhal	Int-S	2		
		carbon monoxide	1	1						
255ph	37 y M				A	Inhal	Unt-E	1		
		carbon monoxide	1	1					carboxyhemoglobin	53 % In Blood (unspecified) @ 5 m (pe)
256ph	40 y M				A	Inhal	Int-S	2		
		carbon monoxide	1	1						
257ai	41 y M				A	Ingst+ Inhal+ Derm	Unt-E	2		
		carbon monoxide	1	1						
		fentanyl (transdermal)	2	2						
		diphenhydramine	3	3						
		oxycodone	4	4						
		acetaminophen	5	5						
258p	43 y F				A	Ingst+ Inhal	Int-S	1		
		hydrogen sulfide	1	1						
259p	44 y M				A	Inhal	Unt-E	3		
		smoke	1	1						
260pi	44 y M				A	Inhal	Unt-E	1		
		carbon monoxide	1	1						
261p	45 y M				A	Inhal	Unt-E	1		
		carbon monoxide	1	1						
262p	45 y F				A	Inhal	Unt-E	1		
		smoke	1	1					carboxyhemoglobin	28 mg/dL In Blood (unspecified) @ Unknown
263pi	47 y F				A	Inhal	Unt-E	1		
		carbon monoxide	1	1						
264ai	47 y M				A	Inhal	Unt-E	2		
		carbon monoxide	1	1						
265p	47 y M				A	Ingst+ Inhal	Int-S	1		
		helium	1	1						
		ethanol	2	2						
266	47 y M				A	Inhal+ Derm	Unt-O	3		
		ethylene	1	1						
267hai	47 y M				A	Ingst+ Inhal	Int-S	1		
		carbon monoxide	1	1					carboxyhemoglobin	0 Other (see abst) In Whole Blood @ 24 h (pe)
		amitriptyline	2	2					amitriptyline	0 Other (see abst) In Whole Blood @ Unknown
		amitriptyline	2	2					nortriptyline	0 Other (see abst) In Whole Blood @ Unknown
		ethylene glycol (antifreeze)	3	3					ethylene glycol	11 mg/dL In Whole Blood @ Unknown
		cocaine	4	4					cocaine	0 Other (see abst) In Whole Blood @ Unknown
		cocaine	4	4					benzoylecognine	1 Other (see abst) In Whole Blood @ Unknown
		marijuana	5	5						
268ai	48 y M				A	Inhal+ Unk	Oth-M	2		
		smoke	1	1						
		carbon monoxide	2	2						
		cocaine	3	3						
		diphenhydramine	4	4						
269	49 y M				A	Inhal	Unt-E	1		
		carbon monoxide	1	1						
		smoke	2	2						
270ai	49 y M				A	Ingst+ Inhal	Int-S	2		
		carbon monoxide	1	1						
		diphenhydramine	2	2						
		ethanol	3	3						
271a	49 y M				U	Inhal	Unt-E	1		
		smoke	1	1					carboxyhemoglobin	60 % In Blood (unspecified) @ Unknown
		smoke	1	1					carboxyhemoglobin	8.3 % In Blood (unspecified) @ Unknown
		cyanide	2	2						
272ai	49 y M				A	Ingst+ Inhal	Unt-E	2		
		smoke	1	1						
		ethanol	2	2						
273ai	49 y M				A	Ingst+ Inhal	Oth-M	2		
		smoke	1	1						
		carbon monoxide	2	2						
		tramadol	3	3						
		cocaine	4	4						
274pi	50 y M				A	Inhal	Unt-E	1		
		carbon monoxide	1	1						
275ai	50 y F				A	Ingst+ Inhal+ Unk	Unt-E	2		
		smoke	1	1						
		carbon monoxide	2	2						
		cocaine	3	3						
		morphine	4	4						
		ethanol	5	5						
276ai	51 y M				A	Ingst+ Inhal	Unt-E	2		
		smoke	1	1						
		carbon monoxide	2	2						
		ethanol	3	3						
277ai	51 y M				A	Inhal	Int-S	2		
		carbon monoxide	1	1						
278pha	51 y M				A	Inhal	Unt-O	1		
		carbon monoxide	1	1					carboxyhemoglobin	60 % In Blood (unspecified) @ Autopsy
		freon	2	2						
279pa	51 y M				A	Inhal	Int-S	1		
		smoke	1	1					carboxyhemoglobin	60 % In Blood (unspecified) @ Autopsy
280ai	52 y M				A	Ingst+ Inhal	Unt-E	2		
		carbon monoxide	1	1						
		sertraline	2	2						
		tramadol	3	3						
		trazodone	4	4						
		diphenhydramine	5	5						
		promethazine	6	6						
281ph	52 y F				A	Ingst+ Inhal	Unt-E	2		
		smoke	1	1					carboxyhemoglobin	0.2 % In Blood (unspecified) @ 13 h (pe)
		smoke	1	1					carboxyhemoglobin	34.9 % In Blood (unspecified) @ 15 m (pe)
		smoke	1	1					carboxyhemoglobin	4 % In Blood (unspecified) @ 3 h (pe)
		ethanol	2	2					ethanol	319 mg/dL In Blood (unspecified) @ 15 m (pe)
		cyanide	3	3						
282ai	53 y M				A	Ingst+ Inhal	Int-S	2		
		carbon monoxide	1	1						
		clonazepam	2	2						
		fluoxetine	3	3						
		diphenhydramine	4	4						
		doxylamine	5	5						
[283pha]	53 y M				A	Inhal	Unt-G	1		
		hydrogen sulfide	1	1						
284	54 y M				A	Inhal	Unt-E	1		
		carbon monoxide	1	1						
285ai	55 y M				A	Ingst+ Inhal	Unt-E	2		
		smoke	1	1						
		carbon monoxide	2	2						
		ethanol (non-beverage)	3	3						
		diphenhydramine	4	4						
286ai	56 y M				A	Ingst+ Inhal	Unt-E	2		
		carbon monoxide	1	1						
		diphenhydramine	2	2						
287ai	56 y F				A	Ingst+ Inhal	Unt-E	2		
		smoke	1	1						
		carbon monoxide	2	2						
		diphenhydramine	3	3						
		ethanol	4	4						
288ph	56 y M				A	Inhal	Unt-E	3		
		carbon monoxide	1	1						
289pa	57 y F				A	Inhal	Oth-M	1		
		smoke	1	1					carboxyhemoglobin	60 % In Blood (unspecified) @ Autopsy
290ai	57 y F				A	Ingst+ Inhal	Unt-E	2		
		smoke	1	1						
		carbon monoxide	2	2						
		sertraline	3	3						
291pa	57 y M				A	Inhal	Unt-E	1		
		smoke	1	1					carboxyhemoglobin	60 % In Blood (unspecified) @ Autopsy
292ph	58 y F				A	Inhal	Unt-E	1		
		carbon monoxide	1	1						
		cyanide	2	2						
293p	58 y M				A	Inhal	Unt-O	1		
		hydrogen sulfide	1	1						
294ai	59 y F				A	Ingst+ Inhal	Unt-E	2		
		smoke	1	1						
		ethanol (non-beverage)	2	2						
		quinine	3	3						
295p	59 y F				A	Inhal	Unt-E	2		
		smoke	1	1						
296ai	59 y F				U	Inhal	Int-S	2		
		helium	1	1						
297ai	59 y M				A	Ingst+ Inhal	Unt-E	2		
		smoke	1	1						
		carbon monoxide	2	2						
		ethanol	3	3						
298ai	59 y F				A	Inhal	Unt-E	2		
		smoke	1	1						
		carbon monoxide	2	2						
299ai	60 y M				A	Inhal	Unt-E	2		
		carbon monoxide	1	1						
300ph	60 y M				C	Inhal	Unt-E	3		
		carbon monoxide	1	1					carboxyhemoglobin	13.9 % In Blood (unspecified) @ 15 m (pe)
301p	63 y M				A	Ingst+ Inhal	Int-S	2		
		carbon monoxide	1	1					carboxyhemoglobin	10 % In Blood (unspecified) @ Unknown
		doxepin	2	2						
		citalopram	3	3						
		buspirone	4	4						
		gabapentin	5	5						
		ethanol	6	6					ethanol	353 mg/dL In Blood (unspecified) @ Unknown
		salicylate	7	7					salicylate	3 mg/dL In Serum @ Unknown
302pa	63 y F				A	Inhal	Unt-E	1		
		smoke	1	1					carboxyhemoglobin	60 % In Blood (unspecified) @ Autopsy
303a	63 y M				A	Ingst+ Inhal	Int-S	1		
		carbon monoxide	1	1					carboxyhemoglobin	42 % In Blood (unspecified) @ 1 h (pe)
304h	64 y M				A	Inhal	Int-S	1		
		chloramine gas	1	1						
305ai	66 y F				A	Inhal	Unt-E	2		
		smoke	1	1						
		carbon monoxide	2	2						
306ai	66 y M				A	Ingst+ Inhal	Unt-E	2		
		smoke	1	1						
		carbon monoxide	2	2						
		trazodone	3	3						
307ai	67 y M				A	Ingst+ Inhal	Int-S	2		
		helium	1	1						
		zolpidem	2	2						
308	68 y M				A	Inhal	Unt-E	1		
		carbon monoxide	1	1						
309p	68 y F				A	Inhal	Oth-M	2		
		smoke	1	1						
310ai	70 y F				A	Inhal	Unt-E	2		
		smoke	1	1						
		carbon dioxide	2	2						
		acetaminophen	3	3						
311ai	70 y M				A	Ingst+ Inhal	Unt-E	2		
		smoke	1	1						
		carbon monoxide	2	2						
		diltiazem	3	3						
		bupropion	4	4						
312ai	70 y M				A	Ingst+ Inhal	Unt-E	2		
		smoke	1	1						
		carbon monoxide	2	2						
		trazodone	3	3						
		zolpidem	4	4						
		fluoxetine	5	5						
313	71 y F				A	Inhal	Unt-E	1		
		carbon monoxide	1	1					carboxyhemoglobin	4.9 % In Blood (unspecified) @ Unknown
314pha	71 y M				A	Inhal	Unt-E	2		
		carbon monoxide	1	1						
315pa	72 y M				A	Ingst+ Inhal	Unt-E	1		
		smoke	1	1					carboxyhemoglobin	60 % In Blood (unspecified) @ Autopsy
		ethanol	2	2					ethanol	40 mg/dL In Blood (unspecified) @ Autopsy
[316pa]	72 y F				A	Inhal	Unt-E	1		
		carbon monoxide	1	1					carboxyhemoglobin	60 % In Blood (unspecified) @ Autopsy
317	73 y F				A	Inhal	Unt-E	1		
		smoke	1	1						
[318pa]	73 y M				A	Inhal	Unt-E	1		
		carbon monoxide	1	1					carboxyhemoglobin	60 % In Blood (unspecified) @ Autopsy
319pha	74 y F				A	Inhal	Unt-E	1		
		smoke	1	1					carboxyhemoglobin	12.7 mmol/L In Blood (unspecified) @ 5.5 h (pe)
		smoke	1	1					carboxyhemoglobin	19.6 mmol/L In Blood (unspecified) @ 1 h (pe)
		smoke	1	1					carboxyhemoglobin	63 mmol/L In Blood (unspecified) @ 0.5 h (pe)
		smoke	1	1					carboxyhemoglobin	9.3 mmol/L In Blood (unspecified) @ 1.75 h (pe)
320pa	74 y F				A	Inhal	Unt-E	1		
		smoke	1	1					carboxyhemoglobin	60 % In Blood (unspecified) @ Autopsy
321ai	75 y M				A	Ingst+ Inhal	Unt-E	2		
		carbon monoxide	1	1						
		verapamil	2	2						
322	76 y M				A	Inhal	Int-S	1		
		carbon monoxide	1	1					carboxyhemoglobin	33 % In Blood (unspecified) @ 1 h (pe)
323pa	78 y M				A	Inhal	Oth-M	1		
		smoke	1	1					carboxyhemoglobin	26 % In Blood (unspecified) @ Autopsy
324p	81 y M				A	Inhal+ Derm	Unt-E	1		
		carbon monoxide	1	1					carboxyhemoglobin	39.7 % In Blood (unspecified) @ 5 m (pe)
		smoke	2	2						
325ha	83 y F				A	Inhal	Unt-E	1		
		smoke	1	1					carboxyhemoglobin	12.7 % In Blood (unspecified) @ 5 m (pe)
		hyperthermia	2	2						
326ph	83 y F				A	Inhal	Unt-E	3		
		carbon monoxide	1	1						
327	86 y F				C	Ingst+ Inhal+ Derm	Unk	2		
		natural gas	1	1						
		substance (non-drug), unknown	2	2						
328ph	88 y M				A	Inhal	Unt-E	1		
		carbon monoxide	1	1					carboxyhemoglobin	63 % In Blood (unspecified) @ Unknown
329ai	89 y M				A	Inhal	Unt-E	2		
		smoke	1	1						
		carbon monoxide	2	2						
330ph	94 y F				A	Inhal	Unt-E	1		
		smoke	1	1						
		carbon monoxide	2	2					carboxyhemoglobin	40 % In Blood (unspecified) @ 1 h (pe)
331p	99 y F				A	Inhal	Unt-E	2		
		smoke	1	1						
332ph	18 m M				A	Inhal	Unt-E	1		
		smoke	1	1					carboxyhemoglobin	3.1 % In Serum @ Unknown
333pi	30 + y F				A	Inhal	Int-S	1		
		hydrogen sulfide	1	1						
334	60 + y M				A	Inhal	Unt-E	1		
		smoke	1	1						
335ph	80 + y F				A	Inhal	Int-S	1		
		carbon monoxide	1	1					carboxyhemoglobin	52 % In Whole Blood @ 30 m (pe)
336p	Unknown adult (> = 20 yrs) M				A	Inhal	Int-S	1		
		hydrogen sulfide	1	1						
337pi	Unknown adult (> = 20 yrs) M				A	Inhal	Int-S	1		
		hydrogen sulfide	1	1						
338pi	Unknown adult (> = 20 yrs) F				A/C	Inhal	Unt-E	1		
		carbon monoxide	1	1						
339p	Unknown adult (> = 20 yrs) M				A	Inhal	Unt-E	1		
		hydrogen sulfide	1	1						
340pa	Unknown age F				A	Inhal+ Unk	Unk	1		
		carbon monoxide	1	1						
		Food (pork)	2	2						
See Also case 117, 201, 208, 1238, 1589										
**Heavy Metals**										
341h	24 y M				A	Ingst	Int-S	2		
		potassium chromate	1	1						
[342h]	73 y M				U	Ingst	Int-A	1		
		lead	1	1						
		ethanol	2	2						
343h	83 y F				A	Ingst	Oth-M	3		
		arsenic *	2	1						
		carvedilol *	1	1						
See Also case 180, 1887										
**Hydrocarbons**										
344p	12 y F				A	Inhal	Int-A	2		
		freon	1	1						
345p	18 y F				A	Inhal	Int-A	3		
		freon	1	1						
346ph	21 y F				A	Inhal+ Unk	Int-A	3		
		freon	1	1						
		methamphetamine	2	2						
347pa	22 y M				A	Ingst+ Inhal	Int-A	1		
		freon	1	1					1,1-difluoroethane	91 mg/L In Blood (unspecified) @ Autopsy
		alprazolam	2	2					alprazolam	0.03 mg/L In Blood (unspecified) @ Autopsy
		oxycodone	3	3					oxycodone	0.043 mg/L In Blood (unspecified) @ Autopsy
		dextromethorphan	4	4					dextromethorphan	0.74 mg/L In Blood (unspecified) @ Autopsy
		dextromethorphan	4	4					dextromethorphan	10 mg/kg In Liver @ Autopsy
		diphenhydramine	5	5					diphenhydramine	0.39 mg/L In Blood (unspecified) @ Autopsy
		diphenhydramine	5	5					diphenhydramine	6.2 mg/kg In Liver @ Autopsy
348p	22 y F				A	Inhal	Int-A	2		
		freon	1	1						
349p	27 y M				A	Ingst+ Inhal	Int-U	1		
		freon	1	1						
		opioid	2	2						
350ai	28 y F				U	Inhal	Int-A	2		
		freon	1	1						
351ai	28 y M				U	Inhal	Int-A	2		
		freon	1	1						
352	31 y F				A	Inhal	Int-A	1		
		freon	1	1						
353ph	32 y F				U	Inhal	Int-A	2		
		freon	1	1						
354p	33 y F				A	Inhal	Int-A	2		
		freon	1	1						
[355ha]	33 y M				C	Inhal	Int-A	1		
		freon	1	1						
356p	33 y F				A	Inhal	Int-A	1		
		freon	1	1						
357pa	34 y F				A	Inhal	Int-A	1		
		freon	1	1						
		alprazolam	2	2					alprazolam	0.033 mg/L In Blood (unspecified) @ Autopsy
		dextromethorphan	3	3					dextromethorphan	0.06 mg/L In Blood (unspecified) @ Autopsy
		diphenhydramine	4	4					diphenhydramine	0.08 mg/L In Blood (unspecified) @ Autopsy
		doxylamine	5	5					doxylamine	0.1 mg/L In Blood (unspecified) @ Autopsy
		fluoxetine	6	6					fluoxetine	0.09 mg/L In Blood (unspecified) @ Autopsy
358ai	34 y F				A	Ingst+ Inhal	Int-A	2		
		freon	1	1						
		doxylamine	2	2						
		fluoxetine	3	3						
		diphenhydramine	4	4						
		alprazolam	5	5						
		dextromethorphan	6	6						
		methadone	7	7						
		hydrocodone	8	8						
		trazodone	9	9						
		acetaminophen	10	10						
359pa	36 y F				A	Inhal	Int-U	1		
		freon	1	1					1,1-difluoroethane	21 mcg/mL In Blood (unspecified) @ Unknown
360ph	40 y M				A	Inhal	Int-A	2		
		freon	1	1						
361pa	40 y M				A	Inhal	Int-A	1		
		freon	1	1						
362ai	42 y F				A	Ingst+ Inhal	Int-A	2		
		freon	1	1						
		oxycodone	2	2						
		trazodone	3	3						
		diphenhydramine	4	4						
363ai	52 y F				U	Inhal	Int-A	2		
		toluene	1	1						
364pai	55 y F				U	Inhal	Int-A	2		
		toluene	1	1						
365a	56 y M				A	Ingst+ Aspir+ Derm	Int-S	3		
		mineral spirits	1	1						
366	58 y M				A	Ingst+ Aspir	Unt-G	2		
		lamp oil	1	1						
		Glacleaner (household)	2	2						
[367ph]	15 m M				A	Ingst	Unt-G	2		
		lamp oil	1	1						
[368]	17 m M				A	Ingst+ Aspir	Unt-G	1		
		gasoline	1	1						
See Also case 278, 533										
**Industrial Cleaners**										
[369]	2 y M				A	Ingst	Unt-G	1		
		hydrofluoric acid	1	1						
370	88 y M				A	Ingst	Int-S	1		
		cleaner (acid)	1	1						
										
**Infectious and Toxin-Mediated Diseases**										
371pa	57 y M				U	Ingst	Unt-F	1		
		Salmonella (food borne)	1	1						
		Staphylococcus (food borne)	2	2						
										
**Other/Unknown Nondrug Substances**										
372pa	18 y F				A/C	Ingst	Int-S	3		
		substance (non-drug), unknown	1	1					oxycodone (total)	63 ng/mL In Blood (unspecified) @ Autopsy
		substance (non-drug), unknown	1	1					methanol	9.5 mg/dL In Blood (unspecified) @ Autopsy
373p	26 y M				A	Ingst	Int-S	2		
		nondrug, unknown	1	1						
See Also case 156, 325, 327, 493, 1290, 1402, 1996										
**Paints and Stripping Agents**										
374ph	50 y M				A	Inhal	Unt-O	2		
		methylene chloride	1	1					carboxyhemoglobin	5.8 % In Blood (unspecified) @ 1 h (pe)
375h	58 y M				A/C	Ingst	Int-U	2		
		varnishes and lacquers	1	1						
		antipsychotic (atypical)	2	2						
		mirtazapine	3	3						
See Also case 2104										
**Pesticides**										
376	19 y M				A	Ingst	Int-S	1		
		aldicarb	1	1						
[377h]	19 y M				A	Ingst	AR-D	1		
		dinitrophenol	1	1						
378h	25 y M				A	Ingst	Int-U	1		
		phosphine	1	1						
379ha	25 y M				A	Ingst	Int-S	1		
		paraquat	1	1						
[380a]	28 y M				A/C	Ingst	Int-S	2		
		dinitrophenol	1	1						
		diphenhydramine	2	2					diphenhydramine	0.058 mcg/mL In Blood (unspecified) @ Unknown
381h	32 y M				A	Ingst	Int-S	1		
		brodifacoum	1	1						
		salicylate	2	2					salicylate	50.4 mg/dL In Blood (unspecified) @ Unknown
		acetaminophen	3	3					acetaminophen	10 mcg/mL In Blood (unspecified) @ Unknown
382	34 y M				A	Ingst	Int-S	1		
		brodifacoum	1	1						
383i	35 y M				A	Ingst	Int-S	1		
		paraquat	1	1						
[384ph]	37 y M				A	Ingst	Unt-G	1		
		DEET (insect repellent)	1	1						
385h	38 y M				A	Ingst	Int-S	1		
		2,4-dichlorophenoxyacetic acid (2,4-D)	1	1						
386	45 y M				A	Ingst	Oth-M	1		
		paraquat	1	1						
387p	48 y M				A	Ingst	Int-S	1		
		organophosphate	1	1						
		malathion	2	2						
388	48 y M				A	Ingst	Int-S	2		
		diquat	1	1						
[389ha]	49 y M				A	Ingst	Int-S	1		
		malathion	1	1						
390	50 y F				A	Ingst	Int-S	1		
		zinc phosphide	1	1						
		ethanol	2	2						
391ha	50 y M				A	Ingst	Int-S	2		
		glyphosate	1	1						
392p	51 y M				A	Ingst	Int-S	1		
		methomyl	1	1						
393p	53 y M				A	Ingst	Unk	2		
		borate	1	1						
		oxycodone	2	2						
		opioid	3	3						
		benzodiazepine	4	4						
		acetaminophen	5	5						
394	60 y M				A	Ingst	Int-S	3		
		glyphosate	1	1						
[395ha]	66 y M				A	Ingst	Unt-M	1		
		paraquat	1	1						
[396ph]	69 y M				A/C	Ingst	Int-S	2		
		carbaryl	1	1						
[397]	70 y F				A	Ingst	Unt-M	1		
		paraquat	1	1						
398h	75 y M				A	Ingst	Int-S	1		
		organophosphate	1	1						
See Also case 156, 209, 239, 1394										
**Plants**										
399p	28 y F				A	Ingst	Int-M	2		
		Pinus genus	1	1						
[400ph]	36 y M				U	Ingst	Int-A	2		
		Mitragyna	1	1						
		paroxetine	2	2						
		lamotrigine	3	3						
[401h]	74 y M				A	Ingst	Int-S	2		
		cardiac glycoside	1	1					digoxin	1.9 ng/mL In Blood (unspecified) @ 17 h (pe)
		cardiac glycoside	1	1					digoxin	2.01 ng/mL In Blood (unspecified) @ 9 h (pe)
		cardiac glycoside	1	1					digoxin	3.23 ng/mL In Blood (unspecified) @ 6 h (pe)
See Also case 1031										
**Weapons of Mass Destruction**										
402p	22 y M				A	Ingst	Int-U	3		
		non-powder, unknown	1	1						
		diphenhydramine	2	2					diphenhydramine	1100 ng/mL In Blood (unspecified) @ Autopsy
See Also case 149, 1997										
**Pharmaceutical Exposures**										
**Analgesics**										
403ai	4 y F				A	Ingst	Unt-G	2		
		hydromorphone	1	1						
		lorazepam	2	2						
		diphenhydramine	3	3						
		acetaminophen	4	4						
[404pa]	5 y F				A	Ingst	Unt-G	1		
		buprenorphine/naloxone (sublingual)	1	1					buprenorphine	2.5 ng/mL In Blood (unspecified) @ Autopsy
405ph	12 y F				A	Ingst	Int-S	1		
		methadone	1	1						
406pa	13 y M				A	Ingst	Int-A	1		
		methadone	1	1					methadone	0.36 mcg/mL In Blood (unspecified) @ Autopsy
		acetaminophen/hydrocodone	2	2						
		ethanol	3	3					ethanol	52.8 mg/dL In Blood (unspecified) @ 10 m (pe)
407pa	14 y M				A	Ingst	Unt-T	1		
		buprenorphine	1	1					buprenorphine	240 Other (see abst) In Liver @ Autopsy
		buprenorphine	1	1					buprenorphine	4.3 ng/mL In Blood (unspecified) @ Autopsy
		alprazolam	2	2					alprazolam	0.03 mg/L In Blood (unspecified) @ Autopsy
		gabapentin	3	3					gabapentin	34 mg/L In Blood (unspecified) @ Autopsy
		ethanol	4	4						
		amphetamine	5	5					amphetamine	0.12 mg/L In Blood (unspecified) @ Autopsy
408pa	15 y M				A	Unk	Int-A	1		
		methadone	1	1					methadone	0.28 mcg/mL In Blood (unspecified) @ Autopsy
		acetaminophen/hydrocodone	2	2						
409	15 y F				A	Ingst	Int-S	1		
		acetaminophen	1	1						
410a	16 y F				A	Ingst	Int-S	1		
		acetaminophen	1	1					acetaminophen	180 mcg/mL In Serum @ 10 h (pe)
411pa	16 y M				A	Ingst	Unk	2		
		morphine	1	1					morphine (free)	0.08 mcg/mL In Whole Blood @ Autopsy
412ph	17 y M				A	Ingst	Int-S	2		
		oxycodone	1	1						
		codeine	2	2						
		dextromethorphan	3	3						
		chlorpheniramine	4	4						
413ph	17 y M				A	Ingst	Int-S	1		
		oxycodone	1	1					oxycodone	1340 ng/mL In Urine (quantitative only) @ 1.5 h (pe)
		oxycodone	1	1					oxycodone	200 ng/mL In Serum @ 1.5 h (pe)
		oxycodone	1	1					oxymorphone	850 ng/mL In Urine (quantitative only) @ 1.5 h (pe)
414ai	17 y M				U	Ingst	Int-A	2		
		oxycodone	1	1						
415ai	17 y M				A	Unk	Int-A	2		
		fentanyl	1	1						
416ha	17 y F				A	Ingst	Int-S	1		
		methadone	1	1						
		cyclic antidepressant, unknown	2	2						
417h	17 y F				A/C	Ingst	Int-S	2		
		acetaminophen	1	1						
		acetaminophen/hydrocodone	2	2						
		salicylate	3	3						
418	18 y M				A	Ingst	Int-S	1		
		salicylate	1	1						
		diphenhydramine	2	2						
419ph	18 y M				A	Ingst	Int-A	2		
		acetaminophen/hydrocodone	1	1					acetaminophen	36 mcg/mL In Blood (unspecified) @ 1 h (pe)
		cyclobenzaprine	2	2						
		alprazolam	3	3						
420pha	18 y F				U	Unk	Int-A	1		
		oxymorphone	1	1					oxymorphone	19 ng/mL In Blood (unspecified) @ Unknown
		alprazolam	2	2					alprazolam	95.1 ng/mL In Blood (unspecified) @ Unknown
		lorazepam	3	3					alpha-oh-alprazolam	94 ng/mL In Blood (unspecified) @ Unknown
		marijuana	4	4					carboxy-thc	9.3 ng/mL In Blood (unspecified) @ Unknown
421ha	19 y M				A	Unk	Unt-G	3		
		opioid	1	1						
		buprenorphine/naloxone	2	2						
422h	19 y M				A	Ingst	Int-S	2		
		acetaminophen/diphenhydramine	1	1						
		salicylate	2	2						
		salicylate	3	3						
423p	19 y F				A	Ingst	Int-S	2		
		acetaminophen/hydrocodone	1	1						
		zolpidem	2	2						
		quetiapine	3	3						
		hydroxyzine	4	4						
		warfarin	5	5						
		nabumetone	6	6						
		benzonatate	7	7						
		acetaminophen	8	8					acetaminophen	268 mcg/mL In Serum @ Unknown
		naproxen	9	9						
		ibuprofen	10	10						
424ai	20 y F				U	Ingst+ Par	Unk	2		
		oxycodone	1	1						
		oxymorphone	2	2						
		hydromorphone	3	3						
425	20 y M				U	Unk	Int-U	2		
		oxycodone	1	1						
		acetaminophen/hydrocodone	2	2						
		carisoprodol	3	3						
426p	20 y M				A	Ingst+ Aspir	Unk	1		
		tramadol	1	1						
		muscle relaxant, unknown	2	2						
		ibuprofen	3	3						
		ethanol	4	4						
		acetaminophen/dextromethorphan/doxalamine	5	5						
427ai	20 y M				U	Ingst	Int-A	2		
		acetaminophen/hydrocodone	1	1						
428ai	20 y F				A	Ingst	Int-U	2		
		hydrocodone	1	1						
		clonazepam	2	2						
		quetiapine	3	3						
		oxycodone	4	4						
		lamotrigine	5	5						
		topiramate	6	6						
		acetaminophen	7	7						
429	20 y M				A	Ingst	Int-S	1		
		salicylate	1	1					salicylate	123 mg/dL In Blood (unspecified) @ 4 h (pe)
430pa	20 y M				C	Ingst	Int-U	3		
		oxycodone	1	1					oxycodone	740 ng/mL In Blood (unspecified) @ Autopsy
		diazepam	2	2					diazepam	180 ng/mL In Blood (unspecified) @ Autopsy
		diazepam	2	2					nordiazepam	220 ng/mL In Blood (unspecified) @ Autopsy
431	20 y F				A	Ingst	Int-S	2		
		opioid	1	1						
		benzodiazepine	2	2						
		acetaminophen	3	3					acetaminophen	13 mcg/mL In Blood (unspecified) @ 6 h (pe)
432ph	20 y F				U	Ingst	Int-A	2		
		oxycodone	1	1						
433pha	20 y M				U	Ingst	Int-U	2		
		acetaminophen/hydrocodone	1	1					acetaminophen	15 mcg/mL In Serum @ Unknown
		acetaminophen/hydrocodone	1	1					acetaminophen	32 mcg/mL In Blood (unspecified) @ Autopsy
		acetaminophen/hydrocodone	1	1					hydromorphone	36 ng/mL In Blood (unspecified) @ Autopsy
		acetaminophen/hydrocodone	1	1					dihydrocodeine	58 ng/mL In Blood (unspecified) @ Autopsy
		acetaminophen/hydrocodone	1	1					hydrocodone	670 ng/mL In Blood (unspecified) @ Autopsy
		alprazolam	2	2					alprazolam	130 ng/mL In Blood (unspecified) @ Autopsy
		alprazolam	2	2					alpha-oh-alprazolam	29 ng/mL In Blood (unspecified) @ Autopsy
		carisoprodol	3	3					meprobamate	7.5 ng/mL In Blood (unspecified) @ Autopsy
		lamotrigine	4	4					lamotrigine	0.94 mcg/mL In Blood (unspecified) @ Autopsy
		lamotrigine	4	4					salicylate	5.8 mg/dL In Blood (unspecified) @ Unknown
434a	20 y M				A	Ingst	Int-S	2		
		diclofenac	1	1						
		meclizine	2	2						
		baclofen	3	3						
435p	20 y F				A	Ingst	Int-A	2		
		oxymorphone	1	1						
436ai	21 y F				U	Ingst	Int-A	2		
		methadone	1	1						
437pa	21 y F				A	Unk	Unk	1		
		morphine	1	1						
		oxycodone	2	2						
		cocaine	3	3						
		amphetamine	4	4						
		citalopram	5	5						
		dextromethorphan	6	6						
		promethazine	7	7						
		fluoxetine	8	8						
		lidocaine	9	9						
		benzodiazepine	10	10						
		marijuana	11	11						
438	21 y F				A	Ingst	Int-S	2		
		acetaminophen/propoxyphene	1	1					acetaminophen	225 mcg/mL In Serum @ 1 h (pe)
		ethanol	2	2						
439pa	21 y M				C	Ingst+ Unk	Int-M	1		
		acetaminophen/hydrocodone	1	1						
440ai	21 y F				A	Ingst	Int-A	2		
		methadone	1	1						
		diphenhydramine	2	2						
		hydroxyzine	3	3						
441ai	22 y M				U	Unk	Int-A	2		
		morphine	1	1						
		diazepam	2	2						
		cyclic antidepressant, unknown	3	3						
		citalopram	4	4						
442ai	22 y M				U	Unk	Int-A	2		
		fentanyl	1	1						
443ai	22 y M				U	Unk	Int-A	2		
		morphine	1	1						
444p	22 y M				C	Ingst	Unk	2		
		methadone	1	1						
		alprazolam	2	2						
445ai	23 y F				A	Ingst	Int-A	2		
		methadone	1	1						
		ethanol (non- beverage)	2	2						
		diphenhydramine	3	3						
		fluoxetine	4	4						
446ai	23 y M				U	Ingst+ Unk	Int-A	2		
		codeine	1	1						
		tramadol	2	2						
		diazepam	3	3						
		metoprolol	4	4						
		promethazine	5	5						
		cyclobenzaprine	6	6						
447pa	23 y M				A	Ingst+ Inhal	Int-A	2		
		methadone	1	1						
		ethanol	2	2						
		marijuana	3	3						
448ha	23 y F				A	Ingst	Unk	1		
		acetaminophen/ hydrocodone	1	1					acetaminophen	82 mg/L In Serum @ 6 h (pe)
449ai	23 y M				A	Unk	Int-A	2		
		methadone	1	1						
		clonazepam	2	2						
450p	23 y M				U	Unk	Int-A	2		
		oxycodone	1	1					oxycodone	0.1 mg/L In Blood (unspecified) @ Unknown
451ph	23 y M				A	Ingst	Unk	2		
		methadone	1	1						
		drug, unknown	2	2						
452ai	23 y F				U	Ingst+ Unk	Int-A	2		
		morphine	1	1						
		amitriptyline	2	2						
		sertraline	3	3						
		cyclobenzaprine	4	4						
		trazodone	5	5						
		hydroxychloroquine	6	6						
453ai	23 y M				U	Ingst+ Unk	Int-A	2		
		fentanyl	1	1						
		alprazolam	2	2						
454p	23 y M				A	Ingst+ Par	Int-A	2		
		fentanyl (transdermal)	1	1						
		drug, unknown	2	2						
455pa	23 y M				A	Par	Int-A	2		
		opioid	1	1						
456	23 y F				A/C	Ingst	Int-S	2		
		acetaminophen/hydrocodone	1	1					acetaminophen	303 mcg/mL In Blood (unspecified) @ Unknown
457p	23 y M				U	Unk	Int-U	2		
		opioid	1	1						
		amphetamine	2	2						
458ai	24 y F				A	Ingst	Int-A	2		
		morphine	1	1						
		tramadol	2	2						
		trazodone	3	3						
		chlorpromazine	4	4						
		benztropine	5	5						
459ai	24 y M				U	Ingst	Int-A	2		
		methadone	1	1						
		opioid	2	2						
		benzodiazepine	3	3						
460ai	24 y M				U	Ingst+ Unk	Int-A	2		
		morphine	1	1						
		ethanol	2	2						
461ai	24 y F				U	Ingst	Int-A	2		
		methadone	1	1						
		alprazolam	2	2						
462ai	24 y M				U	Ingst+ Unk	Int-A	2		
		methadone	1	1						
		alprazolam	2	2						
		morphine	3	3						
463	24 y M				A	Ingst	Int-S	1		
		salicylate	1	1					salicylate	80 mg/dL In Serum @ 1 h (pe)
464ha	24 y M				U	Ingst	Int-S	1		
		fentanyl (transdermal)	1	1						
465pa	24 y M				A	Unk	Unk	2		
		oxycodone	1	1						
		cocaine	2	2						
		buprenorphine/naloxone (film)	3	3						
466ai	25 y M				A	Ingst	Int-A	2		
		methadone	1	1						
		oxycodone	2	2						
		hydrocodone	3	3						
		diazepam	4	4						
467h	25 y F				A	Ingst	Int-S	1		
		acetaminophen	1	1						
		dextromethorphan/guaifenesin	2	2						
468ai	25 y M				A	Ingst	Int-A	2		
		propoxyphene	1	1						
		flunitrazepam	2	2						
		acetaminophen	3	3						
469ai	25 y M				U	Ingst	Int-A	2		
		methadone	1	1						
		ethanol	2	2						
470ai	25 y F				A	Ingst	Int-A	2		
		morphine	1	1						
		diphenhydramine	2	2						
		acetaminophen	3	3						
471ai	25 y M				A	Ingst	Int-A	2		
		methadone	1	1						
		carbamazepine	2	2						
472ai	25 y F				A	Ingst	Int-A	2		
		methadone	1	1						
473ai	25 y M				A	Ingst	Int-A	2		
		oxycodone	1	1						
		clonazepam	2	2						
		chlorpheniramine	3	3						
		dextromethorphan	4	4						
		acetaminophen	5	5						
474ai	25 y M				U	Ingst	Int-A	2		
		acetaminophen/hydrocodone	1	1						
		alprazolam	2	2						
		oxycodone	3	3						
475ai	25 y M				A	Ingst	Int-A	2		
		oxycodone	1	1						
		citalopram	2	2						
476h	25 y M				U	Ingst	Int-A	1		
		acetaminophen/hydrocodone	1	1					acetaminophen	23 mcg/mL In Blood (unspecified) @ 12 h (pe)
		acetaminophen/hydrocodone	1	1					acetaminophen	90 mcg/mL In Blood (unspecified) @ 1 h (pe)
477p	25 y M				A	Ingst	Int-A	2		
		opioid	1	1						
		benzodiazepine	2	2						
478ph	25 y F				A	Ingst+ Par	Int-A	2		
		oxycodone	1	1						
		morphine	2	2						
479pa	26 y F				U	Ingst	Unk	2		
		oxycodone	1	1					oxycodone	320 ng/mL In Whole Blood @ Autopsy
		cyclobenzaprine	2	2					cyclobenzaprine	370 ng/mL In Whole Blood @ Autopsy
		skeletal muscle relaxant	3	3						
480ai	26 y F				A	Ingst	Int-A	2		
		oxycodone	1	1						
		diphenhydramine	2	2						
		quetiapine	3	3						
481h	26 y F				A	Ingst	Int-S	2		
		acetaminophen	1	1					acetaminophen	60 mcg/mL In Blood (unspecified) @ Unknown
482	26 y F				A	Ingst	Int-S	2		
		acetaminophen	1	1						
483p	26 y F				A	Ingst	Int-S	1		
		acetaminophen/hydrocodone	1	1					acetaminophen	46 mcg/mL In Serum @ 1 h (pe)
		alprazolam	2	2						
484h	26 y F				A/C	Ingst	Int-S	2		
		acetaminophen	1	1						
		hydroxyzine	2	2						
		simethicone	3	3						
485	26 y M				U	Ingst	Int-S	1		
		salicylate	1	1					salicylate	106 mg/dL In Blood (unspecified) @ 3 h (pe)
		salicylate	1	1					salicylate	67 mg/dL In Blood (unspecified) @ 1 h (pe)
486a	27 y M				A	Ingst	Int-U	1		
		oxycodone	1	1					oxymorphone	0.012 mg/L In Blood (unspecified) @ Unknown
		oxycodone	1	1					oxycodone	0.47 mg/L In Blood (unspecified) @ Unknown
		clonazepam	2	2					7-aminoclonazepam	0.022 mg/L In Blood (unspecified) @ Unknown
		alpha blocker	3	3						
		trazodone	4	4						
		alprazolam	5	5						
487pa	27 y F				A	Ingst	Int-S	1		
		fentanyl	1	1						
		phenobarbital	2	2						
488	27 y F				U	Ingst	Int-U	2		
		acetaminophen	1	1					acetaminophen	15.1 mcg/mL In Whole Blood @ Unknown
489ai	27 y M				A	Unk	Int-A	2		
		fentanyl	1	1						
490ai	27 y M				A	Ingst	Int-A	2		
		methadone	1	1						
		clonazepam	2	2						
		citalopram	3	3						
491ai	27 y M				U	Ingst	Int-A	2		
		methadone	1	1						
		diazepam	2	2						
492ai	27 y M				U	Ingst	Int-A	2		
		acetaminophen/hydrocodone	1	1						
		hydromorphone	2	2						
		alprazolam	3	3						
		temazepam	4	4						
493p	27 y M				A/C	Ingst+ Inhal	Int-A	2		
		morphine	1	1						
		embalming fluid	2	2						
		cocaine	3	3						
494ai	27 y M				A	Unk	Int-A	2		
		methadone	1	1						
		citalopram	2	2						
[495h]	27 y F				U	Ingst	Int-S	1		
		acetaminophen	1	1					acetaminophen	123 mcg/mL In Serum @ 3 d (pe)
496p	28 y F				A	Ingst	Int-S	1		
		hydromorphone	1	1						
		alprazolam	2	2						
		cyclobenzaprine	3	3						
		zolpidem	4	4						
497ai	28 y M				A	Ingst+ Unk	Int-A	2		
		methadone	1	1						
		cocaine	2	2						
		hydrocodone	3	3						
		alprazolam	4	4						
		diphenhydramine	5	5						
		ethanol	6	6						
498ai	28 y F				U	Ingst	Int-A	2		
		methadone	1	1						
		alprazolam	2	2						
499ai	28 y M				A	Unk	Int-A	2		
		methadone	1	1						
		venlafaxine	2	2						
		oxycodone	3	3						
		amphetamine	4	4						
500	28 y F				A	Ingst	Int-S	2		
		opioid	1	1						
		benzodiazepine	2	2						
		amphetamine	3	3						
		oxycodone	4	4						
501	28 y F				A	Ingst	Int-U	2		
		acetaminophen	1	1						
502ai	28 y F				A	Ingst	Int-A	2		
		tramadol	1	1						
		trazodone	2	2						
503ai	28 y M				A	Ingst	Int-A	2		
		methadone	1	1						
504ai	28 y F				U	Ingst	Int-S	2		
		acetaminophen	1	1						
505ai	28 y F				U	Ingst	Int-A	2		
		oxycodone	1	1						
506	28 y M				A	Ingst	Int-S	1		
		salicylate	1	1					salicylate	123 mg/dL In Blood (unspecified) @ Unknown
507	28 y F				A	Ingst	Int-U	1		
		salicylate	1	1					salicylate	62.4 mg/dL In Serum @ Unknown
		salicylate	1	1					salicylate	65 mg/dL In Serum @ Unknown
		salicylate	1	1					salicylate	86 mg/dL In Serum @ Unknown
508	28 y F				A	Ingst	Int-S	2		
		morphine *	1	1					morphine	0.11 mg/L In Blood (unspecified) @ Unknown
		sumatriptan *	2	1						
		lorazepam	3	2						
		zolpidem	4	3						
509ai	29 y F				A	Ingst	Int-A	2		
		oxycodone	1	1						
		alprazolam	2	2						
		hydrocodone	3	3						
		diphenhydramine	4	4						
		acetaminophen	5	5						
		ethanol	6	6						
510ai	29 y M				A	Ingst	Unt-G	2		
		tramadol	1	1						
		cyclobenzaprine	2	2						
511ai	29 y F				A	Ingst	Int-A	2		
		methadone	1	1						
		alprazolam	2	2						
		tramadol	3	3						
		citalopram	4	4						
512ai	29 y F				U	Ingst+ Aspir+ Unk	Int-A	2		
		morphine	1	1						
		acetaminophen/hydrocodone	2	2						
		alprazolam	3	3						
513	29 y F				A	Ingst	Int-S	2		
		acetaminophen/oxycodone	1	1					acetaminophen	23 mcg/mL In Blood (unspecified) @ Unknown
		lorazepam	2	2						
514ph	29 y F				A/C	Ingst	Int-U	1		
		tramadol	1	1						
		ethanol	2	2						
515a	29 y F				U	Ingst	Unk	1		
		methadone	1	1					methadone	0.24 mg/kg In Blood (unspecified) @ Autopsy
		methadone	1	1					methadone	2.3 mg/kg In Liver @ Autopsy
		oxycodone	2	2					oxycodone	0.28 mg/L In Blood (unspecified) @ Autopsy
516ai	29 y F				A	Ingst	Int-U	2		
		buprenorphine	1	1						
		mirtazapine	2	2						
		naloxone	3	3						
517h	29 y M				C	Ingst	Unk	2		
		acetaminophen/hydrocodone	1	1					acetaminophen	230 mcg/mL In Blood (unspecified) @ Unknown
518pa	29 y M				A	Unk	Int-A	1		
		fentanyl (transdermal)	1	1					fentanyl	110 Other (see abst) In Liver @ Autopsy
		fentanyl (transdermal)	1	1					fentanyl	27 ng/mL In Blood (unspecified) @ Autopsy
		clonazepam	2	2					7-aminoclonazepam	0.042 mg/L In Blood (unspecified) @ Autopsy
		alprazolam	3	3					alprazolam	0.064 mg/L In Blood (unspecified) @ Autopsy
		amphetamine/dextroamphetamine	4	4					amphetamine	0.19 mg/L In Blood (unspecified) @ Autopsy
		citalopram	5	5					citalopram	0.51 mg/L In Blood (unspecified) @ Autopsy
		citalopram	5	5					citalopram	3.9 mg/kg In Liver @ Autopsy
519	29 y M				U	Ingst	Int-U	2		
		acetaminophen/butalbital	1	1						
520ai	30 y M				A	Ingst	Int-A	2		
		methadone	1	1						
		oxycodone	2	2						
		alprazolam	3	3						
		clonazepam	4	4						
		diphenhydramine	5	5						
		doxylamine	6	6						
521a	30 y M				U	Ingst	Unk	2		
		acetaminophen	1	1					acetaminophen	20 mcg/mL In Blood (unspecified) @ Unknown
		metformin	2	2						
522h	30 y F				C	Ingst	Int-M	2		
		acetaminophen	1	1						
523ha	30 y M				A/C	Ingst	Int-S	1		
		acetaminophen/caffeine/salicylate	1	1					salicylate	106.6 mg/dL In Serum @ Unknown
		acetaminophen/caffeine/salicylate	1	1					salicylate	71 mg/dL In Serum @ Unknown
524ai	30 y F				U	Ingst	Int-A	2		
		methadone	1	1						
525pa	30 y M				A	Ingst+ Inhal	Int-U	1		
		methadone	1	1					methadone	77 ng/mL In Blood (unspecified) @ Autopsy
		citalopram	2	2					escitalopram	220 ng/mL In Blood (unspecified) @ Autopsy
		limonene	3	3						
526ai	30 y F				U	Ingst+ Unk	Int-A	2		
		morphine	1	1						
		acetaminophen/hydrocodone	2	2						
		oxycodone	3	3						
		diazepam	4	4						
527ph	30 y M				A	Ingst	Int-S	2		
		acetaminophen/hydrocodone	1	1						
		benzodiazepine	2	2						
		ethanol	3	3						
		marijuana	4	4						
528pha	30 y F				A/C	Ingst+ Unk	Int-U	1		
		morphine	1	1					morphine (free)	44 ng/mL In Blood (unspecified) @ Autopsy
		citalopram	2	2						
		trazodone	3	3						
		chlorpromazine	4	4						
		quetiapine	5	5						
529ha	30 y F				C	Inhal	Int-U	2		
		acetaminophen	1	1					acetaminophen	18.3 mcg/mL In Blood (unspecified) @ 7 h (pe)
		acetaminophen	1	1					acetaminophen	4.9 mcg/mL In Blood (unspecified) @ 20 h (pe)
530h	31 y M				C	Ingst	Int-A	3		
		acetaminophen/oxycodone	1	1					acetaminophen	12 mcg/mL In Blood (unspecified) @ Unknown
531ai	31 y M				A	Unk	Int-U	2		
		morphine	1	1						
		codeine	2	2						
532ai	31 y F				U	Ingst	Int-A	2		
		fentanyl	1	1						
		midazolam	2	2						
533ai	31 y F				U	Ingst+ Inhal	Int-A	2		
		methadone	1	1						
		freon	2	2						
534ai	31 y F				U	Par	Int-A	2		
		fentanyl	1	1						
535ai	31 y M				A	Ingst	Int-A	2		
		oxycodone	1	1						
		ethanol	2	2						
536ai	31 y M				A	Ingst	Int-A	2		
		methadone	1	1						
		lorazepam	2	2						
		alprazolam	3	3						
		clonazepam	4	4						
		amphetamine	5	5						
537ai	31 y F				U	Ingst	Int-A	2		
		acetaminophen/hydrocodone	1	1						
		oxycodone	2	2						
538ai	31 y M				A	Ingst	Int-U	2		
		hydrocodone	1	1						
		oxymorphone	2	2						
		chlorpheniramine	3	3						
		hydroxyzine	4	4						
539ai	31 y M				U	Ingst	Int-A	2		
		methadone	1	1						
540p	31 y F				A/C	Ingst+ Derm	Int-U	2		
		fentanyl	1	1						
541ai	31 y M				U	Ingst	Int-A	2		
		acetaminophen/hydrocodone	1	1						
		skeletal muscle relaxant	2	2						
542a	31 y F				C	Ingst	Int-M	1		
		acetaminophen	1	1					acetaminophen	74 mcg/mL In Serum @ 1 h (pe)
543a	31 y F				U	Ingst	Int-S	1		
		acetaminophen	1	1						
		buprenorphine	2	2						
544h	31 y F				A/C	Ingst	Int-S	1		
		acetaminophen	1	1					acetaminophen	394.7 mcg/mL In Blood (unspecified) @ Unknown
		aripiprazole	2	2						
		alpha blocker	3	3						
		cyclobenzaprine	4	4						
545a	31 y M				C	Ingst	Int-M	1		
		acetaminophen	1	1					acetaminophen	28 mcg/mL In Serum @ 1 h (pe)
546ha	32 y M				U	Unk	Unk	2		
		opioid	1	1						
		methamphetamine	2	2						
		drug, unknown	3	3						
		cyclobenzaprine	4	4					cyclobenzaprine	74 ng/mL In Blood (unspecified) @ Autopsy
		doxylamine	5	5					doxylamine	114 ng/mL In Blood (unspecified) @ Autopsy
		methadone	6	6					methadone	211 ng/mL In Blood (unspecified) @ Autopsy
		fentanyl	7	7					fentanyl	3.7 pg/mL In Blood (unspecified) @ Autopsy
547ai	32 y M				A	Ingst	Int-A	2		
		methadone	1	1						
548ai	32 y F				U	Ingst+ Unk	Int-A	2		
		morphine	1	1						
		oxycodone	2	2						
		diazepam	3	3						
		skeletal muscle relaxant	4	4						
549ai	32 y F				A	Ingst	Int-A	2		
		hydromorphone	1	1						
		citalopram	2	2						
		quetiapine	3	3						
550ai	32 y F				U	Ingst	Int-A	2		
		acetaminophen/hydrocodone	1	1						
		oxycodone	2	2						
		diazepam	3	3						
		oxymorphone	4	4						
551ai	32 y F				A	Ingst+ Derm	Int-A	2		
		fentanyl	1	1						
		doxylamine	2	2						
		carisoprodol	3	3						
		acetaminophen	4	4						
552ai	32 y M				A	Unk	Int-A	2		
		oxycodone	1	1						
		sertraline	2	2						
553ai	32 y M				A	Ingst	Int-A	2		
		oxycodone	1	1						
		alprazolam	2	2						
554pa	32 y M				U	Unk	Unk	1		
		acetaminophen/oxycodone	1	1					oxycodone	0.17 mcg/mL In Blood (unspecified) @ Autopsy
		zolpidem	2	2					zolpidem	0.029 mcg/mL In Blood (unspecified) @ Autopsy
		olanzapine	3	3						
555ai	32 y M				A	Unk	Int-A	2		
		methadone	1	1						
		quetiapine	2	2						
		diphenhydramine	3	3						
		chlorpheniramine	4	4						
		fluoxetine	5	5						
		bupropion	6	6						
556ai	32 y M				U	Ingst	Int-A	2		
		acetaminophen/hydrocodone	1	1						
		alprazolam	2	2						
557ph	32 y M				A	Ingst	Int-S	1		
		acetaminophen/hydrocodone	1	1					acetaminophen	197.3 mcg/mL In Blood (unspecified) @ 1 h (pe)
		acetaminophen/butalbital/caffeine	2	2						
		tizanidine	3	3						
		trazodone	4	4						
		zolpidem	5	5						
		nabumetone	6	6						
558a	33 y F				U	Ingst	Int-U	1		
		acetaminophen/diphenhydramine	1	1					acetaminophen	15 mcg/mL In Serum @ Unknown
		naltrexone	2	2						
		ethanol	3	3						
		salicylate	4	4					salicylate	7 mg/dL In Serum @ Unknown
559	33 y F				A	Ingst	Int-S	2		
		acetaminophen	1	1					acetaminophen	74 mg/L In Serum @ Unknown
		fentanyl (transdermal)	2	2						
		salicylate	3	3						
		ethanol	4	4						
560ai	33 y F				U	Ingst	Int-A	2		
		oxycodone	1	1						
		ethanol	2	2						
561ai	33 y F				U	Ingst	Int-S	2		
		methadone	1	1						
		dextromethorphan	2	2						
		fluoxetine	3	3						
562ai	33 y F				A	Ingst	Int-A	2		
		methadone	1	1						
563pha	33 y F				A	Ingst	Unk	2		
		acetaminophen/hydrocodone	1	1					hydrocodone	67 ng/mL In Serum @ Unknown
		meprobamate	2	2					carisoprodol (n-isopropyl meprobamate)	13.3 mg/L In Blood (unspecified) @ Unknown
		meprobamate	2	2					carisoprodol	3.22 mg/L In Blood (unspecified) @ Unknown
564ai	33 y M				A	Unk	Int-U	2		
		morphine	1	1						
565ai	33 y F				A	Ingst	Int-U	2		
		methadone	1	1						
		meprobamate	2	2						
		diphenhydramine	3	3						
		promethazine	4	4						
		hydroxyzine	5	5						
		pseudoephedrine	6	6						
		dextromethorphan	7	7						
		phenylpropanolamine	8	8						
		acetaminophen	9	9						
566	33 y M				A	Ingst	Int-S	1		
		salicylate	1	1					salicylate	133 mg/dL In Blood (unspecified) @ Unknown
567h	33 y M				C	Ingst	Int-M	2		
		acetaminophen	1	1					acetaminophen	71 mg/L In Serum @ Unknown
568	33 y F				A	Ingst	Int-S	2		
		acetaminophen	1	1						
		metformin	2	2						
		zolpidem	3	3						
		aripiprazole	4	4						
		cyclobenzaprine	5	5						
		pregabalin	6	6						
		clonazepam	7	7						
		lisinopril	8	8						
		acetaminophen/hydrocodone	9	9						
		promethazine	10	10						
		promethazine	11	11						
569	33 y F				C	Ingst	Int-A	2		
		acetaminophen/hydrocodone	1	1						
570	33 y M				U	Ingst	Int-S	3		
		acetaminophen	1	1					acetaminophen	121 mcg/mL In Blood (unspecified) @ Unknown
		ethanol	2	2						
571h	34 y F				A	Ingst	Int-S	1		
		ibuprofen	1	1					ibuprofen	833 mcg/mL In Blood (unspecified) @ Unknown
		acetaminophen/dextromethorphan/doxylamine/pseudoephedrine	2	2						
		acetaminophen/phenylephrine	3	3					acetaminophen	15 mcg/mL In Blood (unspecified) @ Unknown
		acetaminophen/phenylephrine	3	3					acetaminophen	81 mcg/mL In Blood (unspecified) @ Unknown
		diphenhydramine	4	4						
		asenapine	5	5						
572ai	34 y M				A	Unk	Int-A	2		
		methadone	1	1						
		benzodiazepine	2	2						
		lidocaine	3	3						
573ai	34 y M				U	Ingst	Int-A	2		
		propoxyphene	1	1						
		ethanol	2	2						
		diazepam	3	3						
574ai	34 y M				A	Ingst	Int-A	2		
		oxycodone	1	1						
		morphine	2	2						
		alprazolam	3	3						
575ai	34 y M				A	Ingst	Int-A	2		
		morphine	1	1						
		alprazolam	2	2						
		trazodone	3	3						
576ai	34 y M				A	Ingst	Int-A	2		
		methadone	1	1						
		tramadol	2	2						
		oxycodone	3	3						
		diazepam	4	4						
		clonazepam	5	5						
		meprobamate	6	6						
		cyclobenzaprine	7	7						
		diphenhydramine	8	8						
		acetaminophen	9	9						
577ai	34 y F				U	Ingst	Int-A	2		
		acetaminophen/hydrocodone	1	1						
		hydromorphone	2	2						
		butalbital	3	3						
578	34 y F				C	Ingst	Int-M	3		
		acetaminophen/hydrocodone	1	1					acetaminophen	71 mg/L In Serum @ Unknown
579pha	34 y M				A	Ingst	Int-S	1		
		oxycodone	1	1						
		skeletal muscle relaxant	2	2						
		lorazepam	3	3						
		amphetamine (hallucinogenic), alpha-PPP	4	4						
		meprobamate	5	5						
580ai	34 y M				A	Ingst	Int-A	2		
		methadone	1	1						
		ethanol	2	2						
581	34 y F				A	Ingst+ Unk	Int-S	1		
		tramadol	1	1						
		acetaminophen	2	2						
582a	34 y F				C	Unk	Unk	2		
		acetaminophen/hydrocodone	1	1					acetaminophen	47 mcg/mL In Blood (unspecified) @ Autopsy
		acetaminophen/hydrocodone	1	1					morphine (free)	93 mcg/L In Blood (unspecified) @ Autopsy
		acetaminophen/hydrocodone	1	1					hydrocodone (free)	95 mcg/L In Blood (unspecified) @ Autopsy
		lorazepam	2	2					lorazepam	64 mcg/L In Blood (unspecified) @ Autopsy
		benzodiazepine	3	3					7-aminoclonazepam	11 mcg/L In Blood (unspecified) @ Autopsy
		dextromethorphan	4	4					dextromethorphan	18 mcg/L In Blood (unspecified) @ Autopsy
583h	34 y F				A/C	Ingst	Int-M	1		
		acetaminophen/oxycodone	1	1					acetaminophen	21 mg/L In Serum @ 25 h (pe)
		acetaminophen/oxycodone	1	1					acetaminophen	32.4 mg/L In Serum @ 30 m (pe)
584pa	34 y M				A	Ingst	Int-A	1		
		oxycodone	1	1						
		cocaine	2	2						
		amphetamine	3	3						
		acetaminophen	4	4						
		alprazolam	5	5						
		ethanol (non-beverage)	6	6						
585	35 y F				C	Ingst	Unt-T	3		
		acetaminophen	1	1					acetaminophen	119 mcg/mL In Blood (unspecified) @ Unknown
586p	35 y M				U	Ingst	Unk	3		
		tramadol	1	1					tramadol	4947 ng/mL In Blood (unspecified) @ Autopsy
		tramadol	1	1					n-demethyl tramadol	927 ng/mL In Blood (unspecified) @ Autopsy
587ha	35 y M				A	Ingst	Int-S	1		
		salicylate	1	1						
		diphenhydramine	2	2						
		amphetamine	3	3					diphenhydramine	2500 ng/mL In Blood (unspecified) @ Autopsy
		amphetamine	3	3					phentermine	340 ng/mL In Blood (unspecified) @ Autopsy
		amphetamine	3	3					salicylate	49 mg/dL In Blood (unspecified) @ Autopsy
588ai	35 y M				U	Ingst	Int-A	2		
		acetaminophen/hydrocodone	1	1						
		alprazolam	2	2						
589ai	35 y M				A	Ingst	Int-A	2		
		methadone	1	1						
		doxepin	2	2						
		alprazolam	3	3						
		diphenhydramine	4	4						
590ai	35 y M				A	Ingst	Int-A	2		
		methadone	1	1						
		cocaine	2	2						
		citalopram	3	3						
		alprazolam	4	4						
		clonazepam	5	5						
		doxylamine	6	6						
591ai	35 y M				A	Ingst+ Unk	Int-A	2		
		oxycodone	1	1						
		hydromorphone	2	2						
		cocaine	3	3						
		tramadol	4	4						
		alprazolam	5	5						
592ai	35 y M				U	Ingst+ Unk	Int-A	2		
		oxycodone	1	1						
		methamphetamine	2	2						
		alprazolam	3	3						
593ai	35 y F-Pregnant				A	Ingst	Int-A	2		
		methadone	1	1						
		tramadol	2	2						
594ai	35 y F				U	Ingst	Int-A	2		
		acetaminophen/hydrocodone	1	1						
		oxycodone	2	2						
595ha	35 y F				A	Ingst	Int-S	3		
		ibuprofen	1	1						
		ethanol	2	2					ethanol	63 mg/dL In Serum @ Unknown
596ai	35 y F				A	Ingst+ Unk	Int-A	2		
		methadone	1	1						
		diazepam	2	2						
		diphenhydramine	3	3						
597ai	35 y F				A	Ingst+ Unk	Int-A	2		
		oxycodone	1	1						
		hydrocodone	2	2						
		cocaine	3	3						
		alprazolam	4	4						
		fluoxetine	5	5						
		acetaminophen	6	6						
598p	35 y M				A/C	Ingst	Int-S	2		
		tramadol	1	1						
		diazepam	2	2						
		methadone	3	3						
599	35 y M				U	Ingst	Int-S	1		
		acetaminophen	1	1					acetaminophen	188 mcg/mL In Blood (unspecified) @ 60 h (pe)
600ha	35 y M				A/C	Ingst	Int-M	1		
		acetaminophen	1	1					acetaminophen	15.6 mcg/mL In Blood (unspecified) @ Unknown
		acetaminophen	1	1					acetaminophen	28.5 mcg/mL In Blood (unspecified) @ Unknown
601	35 y M				A	Ingst	Int-S	2		
		acetaminophen	1	1						
		salicylate	2	2					acetaminophen	23 ng/mL In Serum @ Unknown
		salicylate	2	2					acetaminophen	32 ng/mL In Serum @ Unknown
		salicylate	2	2					salicylate	34 mg/dL In Serum @ Unknown
		phencyclidine	3	3						
602ha	36 y F				A	Ingst	Int-S	1		
		acetaminophen	1	1					acetaminophen	57 mcg/mL In Blood (unspecified) @ Autopsy
		clonazepam	2	2					clonazepam	77 ng/mL In Blood (unspecified) @ Autopsy
		zolpidem	3	3					zolpidem	110 ng/mL In Blood (unspecified) @ Autopsy
		butalbital	4	4						
		cyclic antidepressant, unknown	5	5						
		skeletal muscle relaxant	6	6						
		meprobamate	7	7						
		fluoxetine	8	8						
		topiramate	9	9						
603	36 y M				C	Ingst	Int-M	2		
		acetaminophen	1	1						
604h	36 y M				C	Ingst	Int-M	2		
		acetaminophen	1	1						
605	36 y M				A	Ingst	Int-S	3		
		acetaminophen	1	1					acetaminophen	107.1 mcg/mL In Serum @ Unknown
		acetaminophen	1	1					acetaminophen	87.8 mcg/mL In Serum @ Unknown
606pha	36 y F				A	Ingst	Int-U	1		
		acetaminophen	1	1					acetaminophen	100 mcg/mL In Plasma @ Unknown
		hydrocodone	2	2					hydrocodone	88 ng/mL In Blood (unspecified) @ Unknown
		carisoprodol	3	3					carisoprodol	1 mg/L In Blood (unspecified) @ Unknown
		carisoprodol	3	3					carisoprodol (n-isopropyl meprobamate)	27 mg/L In Blood (unspecified) @ Unknown
[607h]	36 y M				A	Ingst	Int-S	1		
		salicylate	1	1					salicylate	108 mg/dL In Blood (unspecified) @ 9 h (pe)
		salicylate	1	1					salicylate	86 mg/dL In Blood (unspecified) @ 3 h (pe)
		salicylate	1	1					salicylate	94 mg/dL In Blood (unspecified) @ 6 h (pe)
608ha	37 y M				A	Ingst	Int-S	1		
		salicylate	1	1					salicylate	601 mg/L In Blood (unspecified) @ Unknown
		salicylate	1	1					salicylate	77.5 mg/dL In Serum @ Unknown
609ha	37 y F				C	Ingst	Unt-T	1		
		acetaminophen	1	1					acetaminophen	76 mcg/mL In Blood (unspecified) @ Unknown
610pha	37 y F				A	Par+ Unk	Int-A	2		
		opioid	1	1					morphine	118 ng/mL In Blood (unspecified) @ Autopsy
611ai	37 y F				U	Ingst	Int-A	2		
		methadone	1	1						
612p	37 y F				A	Ingst+ Derm	Int-S	2		
		fentanyl (transdermal)	1	1						
		quetiapine	2	2						
		trazodone	3	3						
		ziprasidone	4	4						
		loratadine	5	5						
613ph	37 y M				A/C	Ingst	Int-U	2		
		methadone	1	1						
614ph	37 y F				A	Ingst	Int-S	2		
		acetaminophen/oxycodone	1	1						
		hydrocodone/ibuprofen	2	2						
		acetaminophen/hydrocodone	3	3						
		morphine	4	4						
615	37 y M				U	Ingst	Unk	2		
		acetaminophen/oxycodone	1	1					oxycodone	0.095 mg/L In Blood (unspecified) @ Autopsy
		acetaminophen/oxycodone	1	1					acetaminophen	23 mcg/mL In Blood (unspecified) @ Unknown
		acetaminophen/oxycodone	1	1					acetaminophen	41 mcg/mL In Blood (unspecified) @ Unknown
		drug, unknown	2	2					zolpidem	0.287 mg/L In Blood (unspecified) @ Autopsy
		benzodiazepine	3	3						
616	38 y M				U	Ingst+ Aspir	Int-U	1		
		oxycodone	1	1						
617pha	38 y M				U	Ingst	Unk	2		
		methadone	1	1					methadone	0.16 mg/L In Blood (unspecified) @ 10 m (pe)
		benzodiazepine	2	2					alprazolam	0.04 mg/L In Blood (unspecified) @ 10 m (pe)
618ai	38 y F				U	Ingst	Int-A	2		
		acetaminophen/hydrocodone	1	1						
		tramadol	2	2						
		nortriptyline	3	3						
		cyclobenzaprine	4	4						
		alprazolam	5	5						
619ai	38 y M				U	Par	Int-A	2		
		morphine	1	1						
		methamphetamine	2	2						
620p	38 y F				U	Unk	Int-S	2		
		tramadol	1	1						
		duloxetine	2	2						
		clonazepam	3	3						
		cyclobenzaprine	4	4						
		gabapentin	5	5						
		ibuprofen	6	6						
		diphenhydramine/ibuprofen	7	7						
621ai	38 y M				U	Ingst+ Unk	Int-A	2		
		morphine	1	1						
		quetiapine	2	2						
622ai	38 y M				U	Ingst	Int-A	2		
		oxycodone	1	1						
		oxymorphone	2	2						
623h	38 y F				U	Ingst	Int-S	2		
		acetaminophen	1	1					acetaminophen	526 mcg/mL In Serum @ Unknown
624pa	39 y F				A/C	Ingst	Int-S	1		
		acetaminophen/hydrocodone	1	1					hydrocodone	0.12 mg/L In Serum @ 4 h (pe)
		acetaminophen/hydrocodone	1	1					acetaminophen	7.3 mg/L In Serum @ 2 h (pe)
625pha	39 y F				A	Ingst	Int-S	2		
		tramadol	1	1						
		hydroxyzine	2	2						
		promethazine	3	3						
		duloxetine	4	4						
		topiramate	5	5						
		cyclobenzaprine	6	6						
		gabapentin	7	7						
		pregabalin	8	8						
		guaifenesin	9	9						
		diuretics, potassium sparing	10	10						
626a	39 y F				A	Ingst	Int-S	1		
		salicylate	1	1					salicylate	101.8 mg/dL In Blood (unspecified) @ Unknown
		temazepam	2	2					temazepam	420 ng/mL In Blood (unspecified) @ Unknown
		ethanol	3	3						
		oxazepam	4	4					oxazepam	70 ng/mL In Blood (unspecified) @ Unknown
627ai	39 y M				U	Ingst	Int-A	2		
		acetaminophen/hydrocodone	1	1						
		alprazolam	2	2						
628ai	39 y F				U	Ingst	Int-A	2		
		oxycodone	1	1						
		acetaminophen/hydrocodone	2	2						
		alprazolam	3	3						
		diphenhydramine	4	4						
		cyclobenzaprine	5	5						
629ai	39 y F				U	Ingst	Int-A	2		
		acetaminophen/hydrocodone	1	1						
		ethanol	2	2						
		alprazolam	3	3						
630ai	39 y F				U	Unk	Int-A	2		
		fentanyl	1	1						
631ai	39 y M				A	Ingst	Int-A	2		
		oxymorphone	1	1						
		diphenhydramine	2	2						
		ethanol	3	3						
632ai	39 y M				A	Ingst	Int-A	2		
		methadone	1	1						
		metoprolol	2	2						
633ai	39 y M				U	Ingst+ Unk	Int-A	2		
		oxycodone	1	1						
		hydromorphone	2	2						
634ai	39 y F				U	Ingst	Int-A	2		
		oxycodone	1	1						
		butalbital	2	2						
635ai	39 y F				A	Ingst	Int-A	2		
		oxycodone	1	1						
		metaxalone	2	2						
		quetiapine	3	3						
		cyclobenzaprine	4	4						
		sertraline	5	5						
		diphenhydramine	6	6						
636ai	39 y F				U	Ingst	Int-A	2		
		acetaminophen/hydrocodone	1	1						
		oxycodone	2	2						
637ai	39 y F				U	Unk	Int-A	2		
		fentanyl	1	1						
		morphine	2	2						
		phentermine	3	3						
		imipramine	4	4						
		diazepam	5	5						
638	39 y F				C	Unk	Unk	2		
		acetaminophen/hydrocodone	1	1						
		valproic acid	2	2						
		levetiracetam	3	3						
		promethazine	4	4						
		hydroxyzine	5	5						
		zolpidem	6	6						
639ai	40 y M				A	Ingst	Int-A	2		
		morphine	1	1						
		oxycodone	2	2						
		hydrocodone	3	3						
		clonazepam	4	4						
		citalopram	5	5						
		acetaminophen	6	6						
640ha	40 y F				C	Ingst	Int-A	1		
		acetaminophen/opioid	1	1					hydrocodone	0.08 mg/L In Blood (unspecified) @ 10 m (pe)
		acetaminophen/opioid	1	1					acetaminophen	5.8 mg/L In Blood (unspecified) @ 10 m (pe)
		carisoprodol	2	2					meprobamate	36 mg/L In Blood (unspecified) @ 10 m (pe)
		carisoprodol	2	2					carisoprodol	9.8 mg/L In Blood (unspecified) @ 10 m (pe)
641ai	40 y M				U	Ingst	Int-A	2		
		methadone	1	1						
		ethanol	2	2						
642ai	40 y F				U	Ingst	Int-A	2		
		methadone	1	1						
		diazepam	2	2						
		alprazolam	3	3						
643ai	40 y F				U	Ingst	Int-A	2		
		acetaminophen/hydrocodone	1	1						
644ai	40 y M				A	Ingst+ Unk	Int-A	2		
		hydromorphone	1	1						
		cocaine	2	2						
		fluoxetine	3	3						
645	40 y M				A	Ingst	Int-S	1		
		salicylate	1	1					salicylate	100 mg/dL In Serum @ Unknown
646	40 y M				A	Ingst	Int-S	2		
		salicylate	1	1					salicylate	100 mg/dL In Blood (unspecified) @ Unknown
647ai	40 y M				U	Ingst+ Par+ Unk	Int-A	2		
		morphine	1	1						
		hydromorphone	2	2						
		amitriptyline	3	3						
		diphenhydramine	4	4						
648	40 y F				U	Ingst	Int-S	2		
		acetaminophen/hydrocodone	1	1					acetaminophen	20.1 mcg/mL In Blood (unspecified) @ Unknown
		acetaminophen/hydrocodone	1	1					acetaminophen	48.9 mcg/mL In Blood (unspecified) @ 8 h (pe)
		acetaminophen/hydrocodone	1	1					acetaminophen	63.4 mcg/mL In Blood (unspecified) @ 1 h (pe)
		skeletal muscle relaxant	2	2						
		zolpidem	3	3						
		carbamazepine	4	4					carbamazepine	4.4 mg/L In Blood (unspecified) @ Unknown
		phenytoin	5	5					phenytoin	5.3 mcg/mL In Blood (unspecified) @ Unknown
		phenytoin	5	5					phenytoin	5.9 mcg/mL In Blood (unspecified) @ Unknown
		gabapentin	6	6						
		estrogens, conjugated	7	7						
649pa	40 y F				A	Ingst	Int-S	1		
		morphine	1	1					morphine	2.48 mg/L In Blood (unspecified) @ 1 h (pe)
		alprazolam	2	2					lorazepam	113 ng/mL In Blood (unspecified) @ 1 h (pe)
		alprazolam	2	2					diazepam	20 ng/mL In Blood (unspecified) @ 1 h (pe)
		alprazolam	2	2					alprazolam	9 ng/mL In Blood (unspecified) @ 1 h (pe)
		citalopram	3	3						
		lamotrigine	4	4						
650ai	40 y M				A	Par	Int-A	2		
		fentanyl	1	1						
		heroin	2	2						
		diphenhydramine	3	3						
651ph	40 y F				U	Ingst	Int-S	1		
		acetaminophen/diphenhydramine	1	1					acetaminophen	139 mcg/mL In Serum @ 0.1 h (pe)
652ai	40 y M				A	Ingst+ Unk	Int-A	2		
		opioid	1	1						
		ethanol	2	2						
653ai	41 y F				U	Ingst	Int-A	2		
		acetaminophen/hydrocodone	1	1						
		alprazolam	2	2						
654ai	41 y F				A	Ingst	Int-A	2		
		hydrocodone	1	1						
		amphetamine	2	2						
		olanzapine	3	3						
		alprazolam	4	4						
		tramadol	5	5						
		hydroxyzine	6	6						
		lidocaine	7	7						
		acetaminophen	8	8						
655ai	41 y F				A	Ingst+ Unk	Int-A	2		
		methadone	1	1						
		cocaine	2	2						
		diphenhydramine	3	3						
		acetaminophen	4	4						
656i	41 y M				A	Ingst	Int-S	1		
		acetaminophen	1	1						
		drug, unknown	2	2						
657h	41 y M				U	Ingst	Unk	3		
		ibuprofen	1	1						
		acetaminophen	2	2					acetaminophen	12.8 mcg/mL In Serum @ 1 d (pe)
		acetaminophen	2	2					acetaminophen	34.7 mcg/mL In Serum @ 0 d (pe)
658ai	41 y F				U	Ingst	Int-A	2		
		acetaminophen/hydrocodone	1	1						
659ai	41 y F				U	Ingst	Int-A	2		
		oxycodone	1	1						
		alprazolam	2	2						
660ai	41 y F				A	Ingst	Int-A	2		
		oxycodone	1	1						
		tramadol	2	2						
661	41 y M				A/C	Ingst	Int-S	1		
		acetaminophen/diphenhydramine	1	1					acetaminophen	33.8 mcg/mL In Blood (unspecified) @ Unknown
		acetaminophen/diphenhydramine	1	1					acetaminophen	62 mcg/mL In Blood (unspecified) @ Unknown
		warfarin	2	2						
		olanzapine	3	3						
		lisinopril	4	4						
		escitalopram	5	5						
		prednisone	6	6						
662ai	41 y M				U	Ingst	Int-A	2		
		oxycodone	1	1						
		oxymorphone	2	2						
		alprazolam	3	3						
663ai	42 y F				A	Ingst+ Unk	Int-U	2		
		methadone	1	1						
		hydroxyzine	2	2						
		cyclobenzaprine	3	3						
		cocaine	4	4						
664pa	42 y M				U	Ingst	Int-S	1		
		methadone	1	1					methadone	0.27 mg/L In Blood (unspecified) @ 1 h (pe)
		clonazepam	2	2						
665pa	42 y F				U	Ingst	Unk	2		
		oxycodone	1	1					trazodone	0.14 mcg/mL In Blood (unspecified) @ Autopsy
		oxycodone	1	1					oxycodone	0.43 mcg/mL In Blood (unspecified) @ Autopsy
666h	42 y M				A	Ingst	Int-M	1		
		acetaminophen	1	1					acetaminophen	27 mcg/mL In Blood (unspecified) @ 2 d (pe)
		acetaminophen	1	1					acetaminophen	93 mg/dL In Blood (unspecified) @ Unknown
667ai	42 y M				A	Inhal	Int-A	2		
		oxycodone	1	1						
		alprazolam	2	2						
668ai	42 y M				U	Ingst	Int-A	2		
		hydromorphone	1	1						
		ethanol	2	2						
669ai	42 y M				U	Ingst	Int-A	2		
		oxycodone	1	1						
		cyclobenzaprine	2	2						
		venlafaxine	3	3						
670ai	42 y M				U	Ingst	Int-A	2		
		oxycodone	1	1						
		cyclobenzaprine	2	2						
		venlafaxine	3	3						
671p	42 y M				A/C	Ingst	Int-S	2		
		acetaminophen/hydrocodone	1	1						
		alprazolam	2	2						
		zolpidem	3	3						
672ai	42 y M				U	Ingst	Int-A	2		
		oxycodone	1	1						
		methadone	2	2						
		diazepam	3	3						
673ai	42 y M				A	Ingst	Int-U	2		
		oxycodone	1	1						
		benzodiazepine	2	2						
674ai	42 y F				U	Ingst	Int-A	2		
		oxycodone	1	1						
		codeine	2	2						
		citalopram	3	3						
		cyclobenzaprine	4	4						
		ethanol	5	5						
675ai	42 y F				A	Ingst+ Inhal	Int-A	2		
		oxycodone	1	1						
		methadone	2	2						
		fluoxetine	3	3						
		amitriptyline	4	4						
		cyclobenzaprine	5	5						
		promethazine	6	6						
		quetiapine	7	7						
676ai	42 y M				A	Ingst	Int-A	2		
		hydromorphone	1	1						
		morphine	2	2						
677	42 y M				A	Unk	Int-U	2		
		methadone	1	1						
		opioid	2	2						
		benzodiazepine	3	3						
		cocaine	4	4						
678ai	42 y M				A	Ingst	Int-A	2		
		methadone	1	1						
		ethanol (non-beverage)	2	2						
		sertraline	3	3						
679ai	42 y M				A	Ingst+ Unk	Int-A	2		
		oxycodone	1	1						
		cocaine	2	2						
		metoprolol	3	3						
		alprazolam	4	4						
		diphenhydramine	5	5						
680ph	42 y F				A/C	Ingst	Int-S	1		
		acetaminophen/hydrocodone	1	1					acetaminophen	132.4 mcg/mL In Blood (unspecified) @ Unknown
		diazepam	2	2						
681h	42 y F				A	Ingst	Int-S	1		
		acetaminophen	1	1						
		salicylate	2	2						
		lorazepam	3	3						
682ai	42 y F				A	Ingst	Int-A	2		
		morphine	1	1						
		oxycodone	2	2						
		clomipramine	3	3						
		promethazine	4	4						
		amphetamine	5	5						
		sertraline	6	6						
683ph	42 y M				A/C	Ingst+ Par	Int-A	1		
		opioid	1	1						
		ethanol	2	2						
684h	42 y M				A	Ingst	Int-S	1		
		acetaminophen	1	1					acetaminophen	377 mcg/mL In Unknown @ Unknown
		ethanol	2	2					ethanol	27 mg/dL In Blood (unspecified) @ Unknown
685ph	42 y F				A	Ingst	Int-U	3		
		opioid	1	1						
		benzodiazepine	2	2						
		chloral hydrate	3	3						
		amitriptyline	4	4						
		amphetamine/dextroamphetamine	5	5						
		citalopram	6	6						
		carisoprodol	7	7						
		amphetamine	8	8						
		ethanol	9	9					ethanol	13 mg/dL In Blood (unspecified) @ Unknown
686ai	43 y F				A	Unk	Int-A	2		
		methadone	1	1						
		alprazolam	2	2						
687ai	43 y F				A	Ingst	Int-A	2		
		oxycodone	1	1						
		chlordiazepoxide	2	2						
		citalopram	3	3						
		diphenhydramine	4	4						
		metoprolol	5	5						
		ethanol	6	6						
688	43 y F				A	Ingst	Int-S	1		
		acetaminophen	1	1					acetaminophen	175 mcg/mL In Blood (unspecified) @ Unknown
689ai	43 y M				A	Ingst+ Unk	Int-A	2		
		fentanyl	1	1						
		chlordiazepoxide	2	2						
		doxylamine	3	3						
		acetaminophen	4	4						
690pha	43 y F				A	Ingst	Int-S	1		
		tramadol	1	1					tramadol	6.2 mcg/mL In Blood (unspecified) @ Unknown
		diphenhydramine	2	2					diphenhydramine	1 mcg/mL In Blood (unspecified) @ Unknown
691ai	43 y M				A	Ingst+ Derm	Int-A	2		
		fentanyl	1	1						
		methadone	2	2						
		oxycodone	3	3						
		ethanol	4	4						
692ai	43 y F				U	Ingst	Int-A	2		
		oxymorphone	1	1						
		citalopram	2	2						
		cyclobenzaprine	3	3						
693ai	43 y M				U	Ingst	Int-A	2		
		acetaminophen/hydrocodone	1	1						
		skeletal muscle relaxant	2	2						
		diazepam	3	3						
694ai	43 y F				U	Ingst+ Unk	Int-A	2		
		morphine	1	1						
		alprazolam	2	2						
695ai	43 y M				U	Ingst+ Unk	Int-A	2		
		acetaminophen/hydrocodone	1	1						
		methamphetamine	2	2						
696ha	43 y F				C	Ingst	Unk	3		
		acetaminophen	1	1						
697ai	43 y M				U	Ingst	Int-A	2		
		oxycodone	1	1						
		alprazolam	2	2						
698ai	43 y M				U	Ingst	Int-A	2		
		acetaminophen/hydrocodone	1	1						
		oxymorphone	2	2						
		ethanol	3	3						
699ai	43 y F				A	Ingst	Int-U	2		
		methadone	1	1						
		hydroxyzine	2	2						
		clonazepam	3	3						
		bupropion	4	4						
		benztropine	5	5						
		amphetamine	6	6						
700p	43 y M				A	Ingst	Int-S	2		
		acetaminophen/oxycodone	1	1					acetaminophen	43 mcg/mL In Blood (unspecified) @ Unknown
701ph	43 y M				U	Par	Int-A	2		
		oxycodone (extended release)	1	1						
702	43 y F				A	Ingst	Int-S	2		
		salicylate	1	1					salicylate	58.7 mg/dL In Blood (unspecified) @ Unknown
		cocaine	2	2						
		acetaminophen	3	3					acetaminophen	108 mcg/mL In Blood (unspecified) @ Unknown
		ethanol	4	4					ethanol	165 mg/dL In Blood (unspecified) @ Unknown
		diphenhydramine	5	5						
		dextromethorphan	6	6						
		pseudoephedrine	7	7						
703h	44 y M				A	Ingst	Int-S	3		
		acetaminophen/oxycodone	1	1					acetaminophen	3 mcg/mL In Blood (unspecified) @ Unknown
704ai	44 y M				U	Ingst	Unk	2		
		opioid	1	1						
		benzodiazepine	2	2						
705ai	44 y F				U	Unk	Int-A	2		
		morphine	1	1						
		chlorpromazine	2	2						
706ai	44 y F				A	Ingst	Int-A	2		
		hydromorphone	1	1						
		dextromethorphan	2	2						
		doxepin	3	3						
		clonazepam	4	4						
		methylphenidate	5	5						
		tramadol	6	6						
		diphenhydramine	7	7						
		acetaminophen	8	8						
707a	44 y F				U	Ingst	Int-S	1		
		acetaminophen	1	1					acetaminophen	13 mcg/mL In Blood (unspecified) @ 5 d (pe)
		acetaminophen	1	1					acetaminophen	442 mcg/mL In Blood (unspecified) @ 1 h (pe)
708p	44 y F				A/C	Ingst	Int-S	2		
		tramadol	1	1						
		clonazepam	2	2						
709a	44 y M				U	Ingst	Int-S	1		
		acetaminophen/diphenhydramine	1	1					acetaminophen	168 mcg/mL In Blood (unspecified) @ 19 h (pe)
710ha	44 y F				A/C	Ingst	Int-S	1		
		acetaminophen *	1	1						
		quetiapine *	2	1						
711h	44 y F				C	Ingst	Unk	2		
		acetaminophen/oxycodone	1	1					acetaminophen	17 mcg/mL In Blood (unspecified) @ Unknown
		pregabalin	2	2						
		tizanidine	3	3						
		ondansetron	4	4						
		sertraline	5	5						
		metaxalone	6	6						
		ethanol	7	7					ethanol	44 mg/dL In Blood (unspecified) @ 6 h (pe)
712ph	44 y F				U	Ingst	Int-S	1		
		acetaminophen/diphenhydramine	1	1					acetaminophen	357 mcg/mL In Blood (unspecified) @ Unknown
713p	45 y F				U	Ingst	Int-S	2		
		acetaminophen/hydrocodone	1	1						
		hydromorphone	2	2						
		diazepam	3	3						
		drug, unknown	4	4						
714	45 y M				A	Ingst	Unk	1		
		salicylate	1	1					salicylate	98 mg/dL In Blood (unspecified) @ Unknown
		acetaminophen	2	2					acetaminophen	84 mcg/mL In Blood (unspecified) @ Unknown
		drug, unknown	3	3						
		valproic acid	4	4					valproic acid	12 mcg/mL In Blood (unspecified) @ Unknown
715ai	45 y M				A	Ingst	Int-A	2		
		morphine	1	1						
		phenothiazine	2	2						
		thioridazine	3	3						
		oxycodone	4	4						
		diphenhydramine	5	5						
		ethanol	6	6						
716ai	45 y M				U	Ingst	Int-A	2		
		oxycodone	1	1						
717ai	45 y F				U	Ingst	Int-A	2		
		opioid	1	1						
718ai	45 y F				U	Unk	Int-A	2		
		morphine	1	1						
719h	45 y F				A	Ingst	Int-S	1		
		acetaminophen/diphenhydramine	1	1						
720p	45 y F				A	Ingst	Int-S	2		
		opioid	1	1						
721ha	45 y F				U	Ingst	Int-U	1		
		acetaminophen	1	1					acetaminophen	12.5 mcg/mL In Blood (unspecified) @ Unknown
722ai	45 y M				U	Ingst	Int-A	2		
		morphine	1	1						
		alprazolam	2	2						
723ai	45 y M				A	Ingst+ Inhal	Int-A	2		
		oxycodone	1	1						
		oxymorphone	2	2						
		diltiazem	3	3						
		metoprolol	4	4						
724ai	45 y F				U	Derm	Int-A	2		
		fentanyl	1	1						
725ai	45 y F				U	Ingst+ Unk	Int-A	2		
		morphine	1	1						
		alprazolam	2	2						
726ha	45 y F				C	Ingst	Int-M	1		
		acetaminophen	1	1						
		skeletal muscle relaxant	2	2						
727h	45 y M				A/C	Ingst	Int-S	1		
		acetaminophen	1	1					acetaminophen	279 mcg/mL In Blood (unspecified) @ 21 h (pe)
		acetaminophen	1	1					acetaminophen	297 mcg/mL In Blood (unspecified) @ 25 h (pe)
		acetaminophen	1	1					acetaminophen	309 mcg/mL In Blood (unspecified) @ 30 h (pe)
		acetaminophen	1	1					acetaminophen	312 mcg/mL In Blood (unspecified) @ 14 h (pe)
		acetaminophen	1	1					acetaminophen	321 mcg/mL In Blood (unspecified) @ 5 h (pe)
		acetaminophen	1	1					acetaminophen	348 mcg/mL In Blood (unspecified) @ 53 h (pe)
		acetaminophen	1	1					acetaminophen	371 mcg/mL In Blood (unspecified) @ 30 m (pe)
		acetaminophen	1	1					acetaminophen	437 mcg/mL In Blood (unspecified) @ 45 h (pe)
		acetaminophen	1	1					acetaminophen	480 mcg/mL In Blood (unspecified) @ 40 h (pe)
		valproic acid	2	2						
		desvenlafaxine	3	3						
728ai	46 y F				A	Ingst	Int-S	2		
		oxycodone	1	1						
		trazodone	2	2						
		clonazepam	3	3						
		cyclobenzaprine	4	4						
		fentanyl	5	5						
		hydrocodone	6	6						
		acetaminophen	7	7						
729ha	46 y F				U	Unk	Unk	3		
		acetaminophen	1	1					acetaminophen	12 mcg/mL In Blood (unspecified) @ Unknown
		acetaminophen	1	1					acetaminophen	6000 mcg/mL In Blood (unspecified) @ Unknown
		methadone	2	2					methadone	0.147 mg/L In Blood (unspecified) @ Unknown
		methadone	2	2					methadone	0.242 mg/L In Blood (unspecified) @ Autopsy
		hydrocodone	3	3					hydrocodone	0.035 mg/L In Blood (unspecified) @ Autopsy
		codeine	4	4					codeine	0.155 mg/L In Blood (unspecified) @ Unknown
		codeine	4	4					codeine	0.166 mg/L In Blood (unspecified) @ Autopsy
730ai	46 y M				U	Ingst+ Unk	Int-A	2		
		oxycodone	1	1						
		oxymorphone	2	2						
		alprazolam	3	3						
731ai	46 y M				U	Ingst	Int-A	2		
		methadone	1	1						
		ethanol	2	2						
		diazepam	3	3						
732ai	46 y F				U	Ingst	Int-A	2		
		methadone	1	1						
733	46 y M				A	Ingst	Int-S	2		
		acetaminophen	1	1					acetaminophen	14.7 mcg/mL In Blood (unspecified) @ 1 h (pe)
		salicylate	2	2					salicylate	41.7 mg/dL In Blood (unspecified) @ 1 h (pe)
		salicylate	2	2					salicylate	90 mg/dL In Blood (unspecified) @ 11 h (pe)
734ai	46 y F				U	Ingst	Int-A	2		
		acetaminophen/hydrocodone	1	1						
		oxycodone	2	2						
		alprazolam	3	3						
735ai	46 y F				U	Ingst	Int-A	2		
		acetaminophen/hydrocodone	1	1						
		alprazolam	2	2						
736ph	46 y F				A	Ingst	Int-S	3		
		methadone	1	1						
		clonidine	2	2						
737	46 y F				A	Ingst	Int-S	1		
		salicylate	1	1					salicylate	75 mg/dL In Serum @ Unknown
738h	46 y M				C	Ingst	Int-M	1		
		acetaminophen	1	1					acetaminophen	16.7 mcg/mL In Blood (unspecified) @ Unknown
739p	46 y M				U	Ingst	Int-S	2		
		colchicine	1	1						
740	46 y F				U	Ingst	Unk	2		
		acetaminophen/hydrocodone	1	1					acetaminophen	106 mcg/mL In Blood (unspecified) @ Unknown
		carisoprodol	2	2						
741	46 y F				A/C	Ingst+ Inhal	Int-U	3		
		acetaminophen/hydrocodone	1	1						
742h	46 y F				U	Ingst	Int-M	1		
		acetaminophen	1	1					acetaminophen	22 mcg/mL In Serum @ 0.5 h (pe)
743h	47 y F				A	Ingst	Unt-G	1		
		salicylate	1	1					salicylate	108 mg/dL In Blood (unspecified) @ 29 h (pe)
		salicylate	1	1					salicylate	57 mg/dL In Blood (unspecified) @ 21 h (pe)
		salicylate	1	1					salicylate	63 mg/dL In Blood (unspecified) @ 0 h (pe)
		salicylate	1	1					salicylate	64 mg/dL In Blood (unspecified) @ 2 h (pe)
		salicylate	1	1					salicylate	70 mg/dL In Blood (unspecified) @ 9.5 h (pe)
		salicylate	1	1					salicylate	81 mg/dL In Blood (unspecified) @ 12 m (pe)
744ha	47 y M				A	Ingst	Int-S	1		
		acetaminophen	1	1					acetaminophen	54.1 mg/L In Serum @ Unknown
		acetaminophen	1	1					acetaminophen	77 mg/dL In Serum @ Unknown
		ethanol	2	2					ethanol	0.08 mg/L In Blood (unspecified) @ Unknown
		ethanol	2	2					ethanol	97 mg/dL In Serum @ Unknown
745ai	47 y M				A	Par+ Unk	Int-A	2		
		hydromorphone	1	1						
		oxycodone	2	2						
		benzodiazepine	3	3						
		marijuana	4	4						
		ethanol	5	5						
746ai	47 y F				A	Unk	Int-U	2		
		morphine	1	1						
		ethanol	2	2						
747ai	47 y M				A	Ingst+ Inhal	Int-A	2		
		oxycodone	1	1						
		alprazolam	2	2						
		sertraline	3	3						
		ethanol (non-beverage)	4	4						
748ai	47 y F				A	Ingst	Int-A	2		
		oxycodone	1	1						
		carisoprodol	2	2						
		diazepam	3	3						
		quetiapine	4	4						
		metoprolol	5	5						
749ai	47 y M				A	Ingst	Int-A	2		
		morphine	1	1						
		quetiapine	2	2						
		trazodone	3	3						
		diltiazem	4	4						
		citalopram	5	5						
		ethanol	6	6						
750pa	47 y F				U	Ingst	Unk	1		
		oxycodone	1	1					oxymorphone	0.019 mg/L In Blood (unspecified) @ Unknown
		oxycodone	1	1					oxymorphone	0.02 mg/L In Blood (unspecified) @ Unknown
		oxycodone	1	1					oxycodone	0.096 mg/L In Blood (unspecified) @ Unknown
		oxycodone	1	1					oxycodone	0.1 mg/L In Blood (unspecified) @ Unknown
		benzodiazepine	2	2						
		marijuana	3	3						
751ai	47 y F				U	Ingst	Int-A	2		
		oxymorphone	1	1						
		acetaminophen/hydrocodone	2	2						
752	47 y F				A/C	Ingst	Int-U	1		
		acetaminophen	1	1					acetaminophen	85 mcg/mL In Blood (unspecified) @ 1 h (pe)
		ethanol	2	2						
753pha	47 y F				A	Ingst	Int-S	3		
		oxycodone	1	1						
		oxycodone (extended release)	2	2						
		methamphetamine	3	3						
754pha	47 y F				A	Ingst	Int-U	3		
		opioid	1	1					morphine	0.398 mg/L In Unknown @ Unknown
755ph	47 y M				A	Ingst	Int-S	2		
		buprenorphine/naloxone (sublingual)	1	1						
		alprazolam	2	2						
		amitriptyline	3	3						
		ethanol	4	4					ethanol	132 mg/dL In Blood (unspecified) @ Unknown
756	47 y F				A	Ingst	Unk	2		
		acetaminophen	1	1						
757a	47 y F				C	Ingst	Int-M	1		
		acetaminophen	1	1						
758h	47 y F				U	Ingst	Int-U	1		
		acetaminophen	1	1					acetaminophen	37.9 mcg/mL In Blood (unspecified) @ Unknown
759pa	47 y F				A	Ingst	Int-S	2		
		acetaminophen/hydrocodone	1	1					hydrocodone	1.1 mcg/mL In Whole Blood @ Autopsy
		ethanol	2	2					ethanol	0.14 % (wt/Vol) In Whole Blood @ Autopsy
		ethanol	2	2					ethanol	0.15 % (wt/Vol) In Vitreous @ Autopsy
		quetiapine	3	3						
		diphenhydramine	4	4						
		dextromethorphan	5	5						
		fluoxetine	6	6					norfluoxetine	3.5 mcg/mL In Whole Blood @ Autopsy
		fluoxetine	6	6					fluoxetine	8.3 mcg/mL In Whole Blood @ Autopsy
760h	48 y M				U	Ingst	Unk	3		
		acetaminophen/diphenhydramine	1	1						
		ibuprofen	2	2						
761ph	48 y M				A	Ingst	Int-S	2		
		oxycodone	1	1						
		ethanol	2	2					ethanol	221 mg/dL In Serum @ Unknown
762ai	48 y M				A	Ingst	Int-A	2		
		methadone	1	1						
		olanzapine	2	2						
		ethanol	3	3						
763ai	48 y F				U	Ingst	Int-A	2		
		oxycodone	1	1						
		alprazolam	2	2						
764	48 y F				U	Ingst	Unk	3		
		acetaminophen	1	1						
765pa	48 y F				A/C	Ingst+ Inhal	Int-U	2		
		oxycodone	1	1					oxycodone	0.43 mcg/mL In Blood (unspecified) @ Autopsy
		cyclobenzaprine	2	2						
		metoprolol	3	3						
		clonidine	4	4						
		nitroglycerin	5	5						
		lisinopril	6	6						
		escitalopram	7	7						
		ibuprofen	8	8						
		diclofenac	9	9						
		azithromycin	10	10						
		cephalexin	11	11						
		doxycycline	12	12						
		methylprednisolone	13	13						
766ai	48 y F				A	Ingst	Int-A	2		
		oxycodone	1	1						
		zolpidem	2	2						
		bupropion	3	3						
767ai	48 y F				U	Ingst	Int-A	2		
		tramadol	1	1						
		citalopram	2	2						
		trazodone	3	3						
		hydroxyzine	4	4						
		diphenhydramine	5	5						
		cyclobenzaprine	6	6						
768h	48 y F				U	Ingst	Int-M	2		
		acetaminophen/hydrocodone	1	1					acetaminophen	59 mcg/mL In Blood (unspecified) @ Unknown
		metformin	2	2						
769ai	48 y M				U	Ingst+ Unk	Int-A	2		
		morphine	1	1						
		oxycodone	2	2						
770ai	48 y F				A	Unk	Int-A	2		
		methadone	1	1						
		morphine	2	2						
		cocaine	3	3						
		fluoxetine	4	4						
		phenytoin	5	5						
771ai	48 y F				U	Ingst	Int-A	2		
		acetaminophen/hydrocodone	1	1						
		tramadol	2	2						
		antidepressant	3	3						
772ai	48 y M				A	Ingst+ Unk	Int-U	2		
		morphine	1	1						
		ethanol	2	2						
773	48 y F				U	Unk	Unk	3		
		acetaminophen/hydrocodone	1	1						
		acetaminophen/oxycodone	2	2						
774h	48 y F				A/C	Ingst	Int-S	1		
		acetaminophen	1	1					acetaminophen	150 mcg/mL In Blood (unspecified) @ 20 h (pe)
		acetaminophen	1	1					acetaminophen	60 mcg/mL In Blood (unspecified) @ 60 h (pe)
		acetaminophen	1	1					acetaminophen	72.5 mcg/mL In Blood (unspecified) @ 36 h (pe)
		carbamazepine (extended release)	2	2						
		rosuvastatin	3	3						
		clonazepam	4	4						
		mirtazapine	5	5						
		benztropine	6	6						
		levothyroxine	7	7						
		nicotine	8	8						
775ai	48 y F				U	Ingst	Int-A	2		
		fentanyl (transdermal)	1	1						
		morphine	2	2						
		oxycodone	3	3						
		diazepam	4	4						
776	48 y F				A	Ingst	Unk	3		
		acetaminophen/diphenhydramine	1	1					acetaminophen	75 mcg/mL In Blood (unspecified) @ 16 h (pe)
777ai	48 y M				U	Ingst	Int-A	2		
		acetaminophen/hydrocodone	1	1						
		oxycodone	2	2						
		alprazolam	3	3						
		quetiapine	4	4						
778ha	48 y F				A	Ingst	Int-S	1		
		acetaminophen/diphenhydramine	1	1					acetaminophen	179 mg/L In Serum @ 21 h (pe)
		acetaminophen/diphenhydramine	1	1					acetaminophen	456 mg/L In Serum @ 5 h (pe)
		acetaminophen/diphenhydramine	1	1					acetaminophen	53 mg/L In Serum @ 38 h (pe)
		ethanol	2	2					ethanol	332 mg/dL In Serum @ Unknown
779ph	48 y F				A	Ingst	Int-S	2		
		acetaminophen/hydrocodone	1	1					acetaminophen	190.2 mcg/mL In Blood (unspecified) @ Unknown
780pha	48 y F				A/C	Ingst	Int-S	1		
		opioid	1	1					morphine	160 ng/mL In Blood (unspecified) @ Autopsy
		opioid	1	1					tramadol	300 ng/mL In Blood (unspecified) @ Autopsy
		opioid	1	1					o-demethyl tramadol	68 ng/mL In Blood (unspecified) @ Autopsy
		cocaine	2	2					benzoylecognine	740 ng/mL In Blood (unspecified) @ Autopsy
		quetiapine	3	3						
781h	48 y F				A/C	Ingst	Int-S	2		
		acetaminophen/hydrocodone	1	1					acetaminophen	329 mcg/mL In Blood (unspecified) @ Unknown
		amitriptyline	2	2						
782	48 y F				A	Ingst	Unt-T	1		
		acetaminophen	1	1						
783a	48 y F				U	Ingst	Int-S	1		
		acetaminophen/hydrocodone	1	1					acetaminophen	0 mg/mL In Blood (unspecified) @ Unknown
		acetaminophen/hydrocodone	1	1					acetaminophen	3.8 mg/L In Blood (unspecified) @ Unknown
		lorazepam	2	2						
		modafinil	3	3						
		pregabalin	4	4						
		phenazopyridine	5	5						
784ai	49 y F				A	Unk	Int-A	2		
		fentanyl	1	1						
		diazepam	2	2						
		diphenhydramine	3	3						
		cocaine	4	4						
		methadone	5	5						
		oxycodone	6	6						
		hydrocodone	7	7						
		morphine	8	8						
		metoclopramide	9	9						
		acetaminophen	10	10						
785ai	49 y M				A	Ingst	Int-A	2		
		oxycodone	1	1						
		ethanol (non-beverage)	2	2						
		diazepam	3	3						
		fluoxetine	4	4						
786ph	49 y M				A/C	Ingst	Int-S	2		
		methadone	1	1						
		hydrocodone	2	2						
		lorazepam	3	3						
787ai	49 y F				U	Ingst+ Unk	Int-A	2		
		droperidol/fentanyl	1	1						
		oxycodone	2	2						
		amitriptyline	3	3						
		alprazolam	4	4						
		diazepam	5	5						
		chlordiazepoxide	6	6						
788ai	49 y M				U	Ingst+ Unk	Int-A	2		
		methadone	1	1						
		cocaine	2	2						
789ai	49 y M				A	Ingst+ Unk	Int-U	2		
		morphine	1	1						
		venlafaxine	2	2						
		diphenhydramine	3	3						
		metoprolol	4	4						
790ai	49 y F				U	Ingst	Int-A	2		
		oxycodone	1	1						
791ai	49 y M				A	Par+ Oth	Int-A	2		
		fentanyl	1	1						
		cocaine	2	2						
		zolpidem	3	3						
		paroxetine	4	4						
792ai	49 y F				U	Ingst	Int-A	2		
		oxycodone	1	1						
		skeletal muscle relaxant	2	2						
793ai	49 y M				A	Ingst	Int-A	2		
		oxycodone	1	1						
		alprazolam	2	2						
794pa	49 y M				A	Ingst	Unk	1		
		opioid	1	1						
		cocaine	2	2						
		ethanol	3	3						
		benzodiazepine	4	4						
795ai	49 y M				A	Par	Int-A	2		
		fentanyl	1	1						
		heroin	2	2						
		levetiracetam	3	3						
		quinine	4	4						
796ph	49 y F				A/C	Unk	Unk	2		
		oxycodone (extended release)	1	1						
		alprazolam	2	2						
797ph	49 y F				A	Ingst	Int-S	2		
		hydromorphone	1	1						
798	50 y F				A	Ingst	Int-S	2		
		acetaminophen/oxycodone	1	1						
		benzodiazepine	2	2						
		quetiapine	3	3						
		pantoprazole	4	4						
		buspirone	5	5						
		ropinirole	6	6						
799	50 y F				C	Ingst	Int-S	1		
		acetaminophen	1	1					acetaminophen	116 mcg/mL In Blood (unspecified) @ Unknown
800ai	50 y M				A	Ingst	Int-U	2		
		morphine	1	1						
		diazepam	2	2						
		fluoxetine	3	3						
		venlafaxine	4	4						
801ai	50 y F				A	Ingst	Int-A	2		
		methadone	1	1						
		diazepam	2	2						
		doxylamine	3	3						
		dextromethorphan	4	4						
		morphine	5	5						
		acetaminophen	6	6						
		citalopram	7	7						
802pha	50 y F				U	Ingst	Int-S	1		
		morphine	1	1					morphine	129 ng/mL In Blood (unspecified) @ Autopsy
		acetaminophen/hydrocodone	2	2					hydrocodone	46 ng/mL In Blood (unspecified) @ Autopsy
		carisoprodol	3	3					carisoprodol	9.8 Other (see abst) In Blood (unspecified) @ Autopsy
		clonazepam	4	4					7-aminoclonazepam	126 ng/mL In Blood (unspecified) @ Autopsy
803ai	50 y M				U	Ingst+ Unk	Int-A	2		
		morphine	1	1						
		tramadol	2	2						
		diazepam	3	3						
		diphenhydramine	4	4						
		sertraline	5	5						
804ai	50 y F				A	Ingst	Int-A	2		
		methadone	1	1						
		ethanol (non-beverage)	2	2						
		diphenhydramine	3	3						
805ai	50 y F				U	Ingst	Int-S	2		
		acetaminophen/hydrocodone	1	1						
		oxycodone	2	2						
		alprazolam	3	3						
806ai	50 y F				A	Ingst	Int-A	2		
		methadone	1	1						
		morphine	2	2						
		oxycodone	3	3						
		tramadol	4	4						
		alprazolam	5	5						
		promethazine	6	6						
		diphenhydramine	7	7						
807ai	50 y F				U	Ingst	Int-A	2		
		oxycodone	1	1						
808ai	50 y M				A	Unk	Int-A	2		
		methadone	1	1						
		heroin	2	2						
		cocaine	3	3						
		tramadol	4	4						
		metoprolol	5	5						
		quinine	6	6						
809ai	50 y F				A	Ingst	Int-A	2		
		methadone	1	1						
		cocaine	2	2						
		oxycodone	3	3						
810	50 y F				A	Ingst	Int-A	1		
		acetaminophen	1	1						
811ai	50 y F				A	Ingst	Int-A	2		
		methadone	1	1						
		alprazolam	2	2						
		doxepin	3	3						
812ai	50 y M				A	Ingst	Int-S	2		
		hydromorphone	1	1						
		diazepam	2	2						
		amitriptyline	3	3						
		fluoxetine	4	4						
813ai	51 y F				A	Ingst	Int-A	2		
		methadone	1	1						
		alprazolam	2	2						
814ai	51 y M				U	Ingst	Int-A	2		
		acetaminophen/hydrocodone	1	1						
		alprazolam	2	2						
		ethanol	3	3						
815	51 y F				A	Ingst	Int-U	1		
		acetaminophen/hydrocodone	1	1					acetaminophen	60 mcg/mL In Serum @ Unknown
816h	51 y F				C	Ingst	Int-M	1		
		acetaminophen/hydrocodone	1	1						
		ethanol	2	2						
817ai	51 y F				A	Ingst	Int-A	2		
		morphine	1	1						
		metoclopramide	2	2						
		anesthetic, local	3	3						
		lidocaine	4	4						
818ai	51 y F				U	Ingst	Int-A	2		
		oxycodone	1	1						
		ethanol	2	2						
819ai	51 y F				U	Ingst+ Unk	Int-A	2		
		meperidine	1	1						
		oxycodone	2	2						
820a	51 y M				U	Ingst	Int-S	1		
		acetaminophen	1	1					acetaminophen	539.6 mg/L In Serum @ 30 m (pe)
821ai	51 y M				U	Ingst	Int-A	2		
		oxycodone	1	1						
		ethanol	2	2						
822ai	51 y M				U	Ingst	Int-A	2		
		oxycodone	1	1						
823ai	51 y M				U	Ingst	Int-A	2		
		oxycodone	1	1						
		alprazolam	2	2						
824ai	51 y M				U	Ingst	Int-A	2		
		oxycodone	1	1						
		tramadol	2	2						
		diazepam	3	3						
825ai	51 y M				A	Ingst+ Unk	Int-A	2		
		methadone	1	1						
		cocaine	2	2						
		ethanol	3	3						
826ai	51 y F				A	Ingst	Int-A	2		
		methadone	1	1						
		oxycodone	2	2						
		amitriptyline	3	3						
		cyclobenzaprine	4	4						
		diphenhydramine	5	5						
		tramadol	6	6						
827ai	51 y F				U	Ingst	Int-A	2		
		methadone	1	1						
		acetaminophen/hydrocodone	2	2						
		promethazine	3	3						
		cyclobenzaprine	4	4						
828ai	51 y F				U	Ingst+ Unk	Int-A	2		
		droperidol/fentanyl	1	1						
		methamphetamine	2	2						
		acetaminophen/hydrocodone	3	3						
		diazepam	4	4						
		alprazolam	5	5						
829ai	51 y M				U	Ingst	Int-A	2		
		oxycodone	1	1						
		ethanol	2	2						
830ai	51 y M				A	Ingst	Int-U	2		
		methadone	1	1						
		alprazolam	2	2						
		citalopram	3	3						
		ethanol	4	4						
831p	51 y F				U	Ingst	Int-S	3		
		acetaminophen	1	1					acetaminophen	74.6 mcg/mL In Blood (unspecified) @ Unknown
832	51 y M				U	Ingst	Int-S	2		
		acetaminophen/hydrocodone	1	1						
		ibuprofen	2	2						
		bupropion	3	3						
		hydrochlorothiazide	4	4						
833p	51 y F				A/C	Ingst+ Unk	Int-U	2		
		oxycodone	1	1						
		methamphetamine	2	2						
		alprazolam	3	3						
		fentanyl (transdermal)	4	4						
		lamotrigine	5	5						
834h	51 y F				A/C	Ingst	Int-M	1		
		salicylate	1	1					salicylate	104.1 mg/dL In Serum @ 5 h (pe)
		salicylate	1	1					salicylate	111.1 mg/dL In Serum @ 1 h (pe)
		salicylate	1	1					salicylate	68.8 mg/dL In Serum @ 22 h (pe)
		salicylate	1	1					salicylate	95.4 mg/dL In Serum @ 9 h (pe)
835p	51 y F				A/C	Ingst	Int-S	2		
		methadone	1	1						
836	51 y M				A	Ingst	Int-U	2		
		acetaminophen	1	1					acetaminophen	14.1 mcg/mL In Blood (unspecified) @ 1 d (pe)
		acetaminophen	1	1					acetaminophen	20.6 mcg/mL In Blood (unspecified) @ 0 d (pe)
		acetaminophen	1	1					acetaminophen	29 mcg/mL In Blood (unspecified) @ Unknown
		metoprolol	2	2						
		acetaminophen/hydrocodone	3	3						
		ethanol	4	4						
837	52 y F				C	Ingst	Unk	2		
		acetaminophen	1	1					acetaminophen	60.8 mcg/mL In Blood (unspecified) @ 7 h (pe)
838	52 y F				A	Ingst	Int-S	1		
		morphine (extended release)	1	1						
		diazepam	2	2						
		zolpidem (extended release)	3	3						
839ai	52 y F				A	Unk	Int-A	2		
		fentanyl	1	1						
840pa	52 y M				A	Ingst	Int-S	3		
		acetaminophen *	2	1						
		benzodiazepine *	1	1						
841ai	52 y F				U	Ingst	Int-A	2		
		acetaminophen/hydrocodone	1	1						
		hydromorphone	2	2						
		skeletal muscle relaxant	3	3						
842h	52 y M				A	Ingst	Int-S	1		
		acetaminophen/hydrocodone	1	1					acetaminophen	172 mcg/mL In Blood (unspecified) @ Unknown
843ai	52 y F				U	Ingst	Int-A	2		
		methadone	1	1						
844ai	52 y F				A	Ingst	Int-A	2		
		hydromorphone	1	1						
		diazepam	2	2						
		ethanol	3	3						
845ai	52 y M				A	Ingst	Int-A	2		
		methadone	1	1						
		promethazine	2	2						
846ai	52 y F				A	Ingst	Int-A	2		
		methadone	1	1						
		amitriptyline	2	2						
		promethazine	3	3						
847	52 y F				A	Ingst	Int-U	1		
		acetaminophen	1	1						
		opioid	2	2						
		barbiturate	3	3						
848ai	52 y M				U	Ingst	Int-A	2		
		opioid	1	1						
		benzodiazepine	2	2						
849ai	52 y F				U	Ingst	Int-A	2		
		tramadol	1	1						
		amitriptyline	2	2						
850ai	52 y F				U	Ingst	Int-A	2		
		acetaminophen/hydrocodone	1	1						
		cyclobenzaprine	2	2						
851ai	52 y M				U	Ingst	Int-A	2		
		oxycodone	1	1						
852p	52 y F				U	Unk	Int-A	1		
		methadone	1	1						
		alprazolam	2	2						
853ai	52 y M				A	Par	Int-A	2		
		oxymorphone	1	1						
854	52 y F				A	Ingst+ Derm	Unk	1		
		acetaminophen/hydrocodone	1	1					acetaminophen	120 mcg/mL In Blood (unspecified) @ 5 h (pe)
		acetaminophen/hydrocodone	1	1					acetaminophen	127 mcg/mL In Blood (unspecified) @ 3.5 d (pe)
		acetaminophen/hydrocodone	1	1					acetaminophen	233 mcg/mL In Blood (unspecified) @ 3 d (pe)
		acetaminophen/hydrocodone	1	1					acetaminophen	57.3 mcg/mL In Blood (unspecified) @ 1 d (pe)
		acetaminophen/hydrocodone	1	1					acetaminophen	92.4 mcg/mL In Blood (unspecified) @ 2.5 h (pe)
		fentanyl	2	2						
		clonazepam	3	3						
		gabapentin	4	4						
		venlafaxine	5	5						
		ethanol	6	6						
855ai	52 y F				U	Ingst	Int-A	2		
		acetaminophen/hydrocodone	1	1						
		alprazolam	2	2						
		quetiapine	3	3						
		skeletal muscle relaxant	4	4						
856ai	52 y F				U	Ingst	Int-A	2		
		codeine	1	1						
857ai	52 y F				U	Ingst	Int-A	2		
		oxycodone	1	1						
		alprazolam	2	2						
		skeletal muscle relaxant	3	3						
858	52 y M				A/C	Ingst	Int-S	1		
		acetaminophen	1	1						
		ethanol	2	2						
859h	52 y M				A/C	Ingst	Int-S	2		
		acetaminophen	1	1					acetaminophen	252 mcg/mL In Blood (unspecified) @ 15 m (pe)
		ethanol	2	2						
860ha	52 y M				A/C	Ingst	Int-S	1		
		morphine (extended release)	1	1					morphine (free)	180 ng/mL In Blood (unspecified) @ Unknown
861	52 y M				A	Ingst	Int-S	1		
		oxycodone	1	1						
		ethanol	2	2						
862ai	53 y M				A	Ingst	Unt-G	2		
		morphine	1	1						
		tramadol	2	2						
		diazepam	3	3						
		dextromethorphan	4	4						
863ai	53 y F				A	Unk	Int-A	2		
		morphine	1	1						
		cyclobenzaprine	2	2						
		levetiracetam	3	3						
864ai	53 y M				A	Unk	Int-A	2		
		methadone	1	1						
		heroin	2	2						
		alprazolam	3	3						
		promethazine	4	4						
		codeine	5	5						
865ai	53 y M				A	Ingst	Int-A	2		
		morphine	1	1						
		diazepam	2	2						
		trazodone	3	3						
		bupropion	4	4						
		dextromethorphan	5	5						
		mirtazapine	6	6						
		codeine	7	7						
866h	53 y F				A/C	Ingst	Int-S	3		
		acetaminophen	1	1					acetaminophen	479.2 mcg/mL In Serum @ Unknown
		acetaminophen	1	1					salicylate	95.3 mg/dL In Serum @ Unknown
		salicylate	2	2						
		benzodiazepine	3	3						
867ai	53 y F				A	Unk	Int-A	2		
		methadone	1	1						
		cocaine	2	2						
		promethazine	3	3						
		ethanol	4	4						
868ai	53 y F				U	Ingst+ Unk	Int-A	2		
		fentanyl	1	1						
		methadone	2	2						
		alprazolam	3	3						
869ai	53 y F				U	Ingst+ Unk	Int-A	2		
		morphine	1	1						
870ai	53 y M				U	Unk	Int-A	2		
		morphine	1	1						
		ethanol	2	2						
871ai	53 y M				U	Ingst	Int-S	2		
		tramadol	1	1						
		amitriptyline	2	2						
		ethanol	3	3						
872ai	53 y M				U	Ingst	Int-A	2		
		acetaminophen/hydrocodone	1	1						
873h	53 y F				A	Ingst	Int-S	1		
		acetaminophen	1	1					acetaminophen	32 mcg/mL In Blood (unspecified) @ Unknown
874pha	53 y F				U	Ingst	Int-S	1		
		acetaminophen	1	1					acetaminophen	200 mg/mL In Blood (unspecified) @ Unknown
		acetaminophen	1	1					acetaminophen	400 mcg/mL In Blood (unspecified) @ Unknown
		benzodiazepine	2	2						
		ethanol	3	3						
875ph	54 y F				A/C	Ingst	Int-S	1		
		acetaminophen/hydrocodone	1	1					acetaminophen	294 mcg/mL In Blood (unspecified) @ 12 h (pe)
		acetaminophen/hydrocodone	1	1					acetaminophen	408 mcg/mL In Blood (unspecified) @ Unknown
		Hydromorphone	2	2						
		alprazolam	3	3						
		dexlansoprazole	4	4						
876ai	54 y F				U	Ingst	Int-A	2		
		acetaminophen/hydrocodone	1	1						
		oxycodone	2	2						
		citalopram	3	3						
		cyclobenzaprine	4	4						
		nortriptyline	5	5						
877ai	54 y F				A	Ingst+ Unk	Int-A	2		
		morphine	1	1						
		cocaine	2	2						
		carisoprodol	3	3						
		diphenhydramine	4	4						
878ai	54 y M				U	Ingst	Int-A	2		
		oxycodone	1	1						
		alprazolam	2	2						
		ethanol	3	3						
879ai	54 y F				U	Unk	Int-A	2		
		morphine	1	1						
		skeletal muscle relaxant	2	2						
		promethazine	3	3						
		trazodone	4	4						
		venlafaxine	5	5						
880ai	54 y M				U	Ingst	Int-A	2		
		oxycodone	1	1						
		alprazolam	2	2						
		skeletal muscle relaxant	3	3						
881ai	54 y M				U	Ingst	Int-A	2		
		oxymorphone	1	1						
		ethanol	2	2						
882ai	54 y F				U	Ingst+ Unk	Int-A	2		
		morphine	1	1						
		phentermine	2	2						
		diazepam	3	3						
		alprazolam	4	4						
		methadone	5	5						
883ai	54 y F				U	Ingst	Int-A	2		
		oxycodone	1	1						
		temazepam	2	2						
884ai	54 y F				U	Ingst	Int-A	2		
		tramadol	1	1						
		citalopram	2	2						
		fluoxetine	3	3						
		cyclobenzaprine	4	4						
		quetiapine	5	5						
885p	54 y M				A	Oth	Int-A	3		
		opioid	1	1						
886p	54 y M				U	Ingst	Int-S	2		
		oxycodone	1	1						
887ai	54 y F				U	Ingst+ Unk	Int-A	2		
		acetaminophen/hydrocodone	1	1						
		diphenhydramine	2	2						
		morphine	3	3						
888ai	54 y M				A	Ingst	Int-S	2		
		methadone	1	1						
		temazepam	2	2						
		diphenhydramine	3	3						
		ethanol	4	4						
889ai	54 y F				A	Ingst	Int-A	2		
		morphine	1	1						
		cyclobenzaprine	2	2						
		ethanol	3	3						
890ai	54 y F				A	Ingst	Int-U	2		
		hydrocodone	1	1						
		acetaminophen	2	2						
		diphenhydramine	3	3						
		ethanol	4	4						
891ha	54 y F				U	Ingst+ Unk	Int-U	2		
		morphine	1	1					morphine	0.028 mg/L In Blood (unspecified) @ Unknown
		morphine	1	1					morphine	0.347 mg/L In Whole Blood @ Autopsy
		acetaminophen/hydrocodone	2	2						
		benzodiazepine	3	3					midazolam	130 ng/mL In Whole Blood @ Autopsy
		benzodiazepine	3	3					7-aminoclonazepam	16 ng/mL In Whole Blood @ Autopsy
892ai	54 y F				A	Ingst	Int-A	2		
		methadone	1	1						
		amitriptyline	2	2						
		diphenhydramine	3	3						
		ethanol	4	4						
893a	54 y F				A	Ingst	Int-S	2		
		acetaminophen	1	1					acetaminophen	155 mcg/mL In Blood (unspecified) @ Unknown
894ha	54 y F				U	Ingst	Unk	2		
		acetaminophen/oxycodone	1	1					acetaminophen	184 mcg/mL In Blood (unspecified) @ Unknown
895ph	54 y F				A/C	Ingst	Int-S	2		
		acetaminophen/hydrocodone	1	1						
		ethanol	2	2						
		imipramine	3	3					imipramine	0.16 mg/L In Blood (unspecified) @ Autopsy
		imipramine	3	3					desipramine	0.26 mg/L In Blood (unspecified) @ Autopsy
		oxycodone	4	4					oxycodone	0.059 mg/L In Blood (unspecified) @ Unknown
		oxycodone	4	4					oxycodone	0.062 mg/L In Blood (unspecified) @ Unknown
896	54 y M				C	Ingst	Int-M	1		
		acetaminophen/diphenhydramine	1	1						
		caffeine/salicylamide/salicylate	2	2						
897	55 y M				A	Ingst	Int-S	1		
		salicylate	1	1					salicylate	84 mg/dL In Serum @ Unknown
		olanzapine	2	2						
		naproxen	3	3						
898ai	55 y M				U	Ingst	Int-S	2		
		acetaminophen/hydrocodone	1	1						
		bupropion	2	2						
		carbamazepine	3	3						
		amitriptyline	4	4						
899ai	55 y F				U	Ingst+ Unk	Int-A	2		
		oxycodone	1	1						
		alprazolam	2	2						
		morphine	3	3						
		citalopram	4	4						
		diazepam	5	5						
900h	55 y F				U	Ingst	Int-S	2		
		acetaminophen	1	1					acetaminophen	27.1 mg/L In Blood (unspecified) @ Unknown
		acetaminophen	1	1					acetaminophen	31.5 mg/L In Blood (unspecified) @ Autopsy
		acetaminophen	1	1					acetaminophen	342 mg/kg In Gastric (stomach content) @ Autopsy
901ai	55 y M				U	Ingst	Int-A	2		
		oxycodone	1	1						
		oxymorphone	2	2						
		diazepam	3	3						
902ai	55 y M				U	Ingst	Int-A	2		
		oxycodone	1	1						
		oxymorphone	2	2						
903ai	55 y F				A	Unk	Int-A	2		
		fentanyl	1	1						
		oxycodone	2	2						
		trazodone	3	3						
		bupropion	4	4						
		metoprolol	5	5						
904ai	55 y M				A	Unk	Int-A	2		
		morphine	1	1						
905ai	55 y F				U	Ingst+ Unk	Int-A	2		
		fentanyl	1	1						
		diazepam	2	2						
		acetaminophen/hydrocodone	3	3						
		phentermine	4	4						
		promethazine	5	5						
906pha	55 y F				U	Ingst	Int-U	1		
		oxycodone	1	1						
		acetaminophen	2	2					acetaminophen	28.9 mcg/mL In Blood (unspecified) @ Unknown
		ethanol	3	3					ethanol	270 mg/mL In Blood (unspecified) @ Unknown
907ai	55 y M				A	Unk	Int-A	2		
		morphine	1	1						
		cocaine	2	2						
		ethanol (non-beverage)	3	3						
908ai	55 y M				A	Ingst	Int-A	2		
		fentanyl	1	1						
		methadone	2	2						
		diltiazem	3	3						
		citalopram	4	4						
909ha	55 y F				A	Ingst	Int-S	1		
		acetaminophen/hydrocodone	1	1					acetaminophen	10 mg/L In Blood (unspecified) @ Unknown
		acetaminophen/hydrocodone	1	1					hydrocodone	54 Other (see abst) In Blood (unspecified) @ Unknown
		carisoprodol	2	2					carisoprodol	19 mg/L In Blood (unspecified) @ Unknown
		carisoprodol	2	2					meprobamate	25 mg/L In Blood (unspecified) @ Unknown
910ai	55 y M				A	Ingst	Int-A	2		
		methadone	1	1						
		oxycodone	2	2						
		cocaine	3	3						
		alprazolam	4	4						
		clonazepam	5	5						
		meprobamate	6	6						
		diphenhydramine	7	7						
911ha	55 y F				A	Ingst	Int-S	1		
		acetaminophen	1	1					acetaminophen	198 mcg/mL In Blood (unspecified) @ 13 h (pe)
		acetaminophen	1	1					acetaminophen	300 mcg/mL In Blood (unspecified) @ Unknown
		acetaminophen	1	1					acetaminophen	31 mcg/mL In Blood (unspecified) @ 26 h (pe)
912ai	55 y F				A	Ingst	Int-A	2		
		oxycodone	1	1						
		quetiapine	2	2						
		trazodone	3	3						
		citalopram	4	4						
		cyclobenzaprine	5	5						
		methylphenidate	6	6						
		orphenadrine	7	7						
		ethanol	8	8						
913h	55 y F				A	Ingst	Int-M	3		
		oxycodone	1	1						
		ethanol	2	2					ethanol	179 mg/dL In Blood (unspecified) @ Unknown
914h	56 y F				A	Ingst	Unk	1		
		salicylate	1	1					salicylate	170 mg/dL In Blood (unspecified) @ Unknown
915	56 y F				A	Ingst	Int-S	2		
		acetaminophen	1	1						
		salicylate	2	2					salicylate	12.6 mg/dL In Serum @ Unknown
		drug, unknown	3	3						
916	56 y F				A	Ingst	Int-S	2		
		acetaminophen/hydrocodone	1	1					acetaminophen	255 mcg/mL In Blood (unspecified) @ Unknown
		trazodone	2	2						
		temazepam	3	3						
917	56 y M				A	Ingst	Int-U	1		
		acetaminophen	1	1						
		ethanol	2	2						
918ai	56 y F				U	Ingst+ Unk	Int-A	2		
		morphine	1	1						
		ethanol	2	2						
919ai	56 y M				A	Inhal+ Oth	Int-A	2		
		morphine	1	1						
		cocaine	2	2						
		tramadol	3	3						
		doxepin	4	4						
		codeine	5	5						
920pha	56 y M				A	Ingst	Int-S	1		
		oxycodone	1	1						
		ethanol	2	2					ethanol	40 mg/dL In Plasma @ Unknown
		trazodone	3	3						
		venlafaxine (extended release)	4	4						
		droperidol/fentanyl	5	5						
921ai	56 y F				U	Ingst	Int-A	2		
		oxycodone	1	1						
		diazepam	2	2						
		skeletal muscle relaxant	3	3						
922a	56 y M				A	Ingst	Int-S	2		
		acetaminophen/hydrocodone	1	1						
923phi	56 y F				A	Ingst	AR-D	2		
		fentanyl (transdermal)	1	1						
		acetaminophen/hydrocodone	2	2						
		clonazepam	3	3						
		quetiapine	4	4						
		tizanidine	5	5						
		promethazine	6	6						
		esomeprazole	7	7						
		atorvastatin	8	8						
924ai	56 y F				A	Ingst	Int-S	2		
		oxycodone	1	1						
		hydrocodone	2	2						
		metaxalone	3	3						
		acetaminophen	4	4						
925	56 y M				C	Ingst	Int-S	1		
		salicylate	1	1					salicylate	107 mg/dL In Blood (unspecified) @ 1 m (pe)
926	56 y F				U	Ingst	Int-S	2		
		acetaminophen	1	1					acetaminophen	111 mcg/mL In Serum @ Unknown
		clonazepam	2	2						
927p	56 y M				A	Ingst	Int-U	1		
		acetaminophen/oxycodone	1	1					acetaminophen	26 mg/L In Plasma @ Unknown
928ai	57 y F				A	Ingst+ Unk	Int-A	2		
		oxymorphone	1	1						
		cocaine	2	2						
		sertraline	3	3						
		paroxetine	4	4						
		tramadol	5	5						
929ai	57 y F				U	Ingst	Int-S	2		
		salicylate	1	1						
		alprazolam	2	2						
		butalbital	3	3						
930ai	57 y F				U	Ingst	Int-A	2		
		oxycodone	1	1						
		cyclobenzaprine	2	2						
		mirtazapine	3	3						
		sertraline	4	4						
		promethazine	5	5						
931ai	57 y M				U	Ingst	Int-A	2		
		methadone	1	1						
		ethanol	2	2						
932ai	57 y F				A	Ingst	Int-A	2		
		methadone	1	1						
		morphine	2	2						
		olanzapine	3	3						
		fluoxetine	4	4						
		oxycodone	5	5						
933ai	57 y F				U	Ingst	Int-S	2		
		codeine	1	1						
		acetaminophen/hydrocodone	2	2						
		fluoxetine	3	3						
		alprazolam	4	4						
		tramadol	5	5						
		cyclobenzaprine	6	6						
		zolpidem	7	7						
934	57 y F				A/C	Ingst	Int-S	2		
		ibuprofen	1	1						
935ai	57 y F				A	Ingst	Int-A	2		
		morphine	1	1						
		oxycodone	2	2						
936ai	57 y F				U	Ingst+ Unk	Int-A	2		
		morphine	1	1						
		hydromorphone	2	2						
		temazepam	3	3						
		ethanol	4	4						
937	57 y F				A	Ingst	Int-S	1		
		salicylate	1	1					salicylate	88.9 mg/dL In Blood (unspecified) @ Unknown
938ai	57 y F				U	Ingst	Int-A	2		
		methadone	1	1						
		diazepam	2	2						
939	57 y F				A	Ingst	Int-M	2		
		acetaminophen	1	1						
940ai	57 y M				U	Ingst	Int-A	2		
		oxycodone	1	1						
		ethanol	2	2						
		diazepam	3	3						
941	57 y M				U	Ingst	Int-U	3		
		tramadol	1	1						
942	57 y M				A/C	Ingst	Int-S	2		
		acetaminophen/butalbital/caffeine	1	1						
943p	58 y M				U	Ingst	Int-U	2		
		methadone	1	1						
944	58 y F				A/C	Ingst	Int-S	2		
		opioid	1	1						
		trazodone	2	2						
		thyroid preparation	3	3						
945ha	58 y F				A	Ingst	Int-S	1		
		acetaminophen	1	1					acetaminophen	41.5 mg/L In Blood (unspecified) @ Unknown
		acetaminophen	1	1					acetaminophen	452 mg/mL In Serum @ Unknown
946ai	58 y F				A	Ingst	Int-A	2		
		methadone	1	1						
		oxycodone	2	2						
		alprazolam	3	3						
		promethazine	4	4						
		fluoxetine	5	5						
947ai	58 y M				U	Ingst	Int-A	2		
		acetaminophen/hydrocodone	1	1						
948pa	58 y F				A	Ingst	Int-S	1		
		methadone	1	1					methadone	1600 ng/mL In Blood (unspecified) @ Autopsy
		methadone	1	1					eddp (2-ethylidene-1,5-dimethyl-3,3-diphenyl pyrrolidine)	340 ng/mL In Blood (unspecified) @ Autopsy
		morphine	2	2					morphine (free)	690 ng/mL In Blood (unspecified) @ Autopsy
		fluoxetine	3	3					norfluoxetine	1000 ng/mL In Blood (unspecified) @ Autopsy
		fluoxetine	3	3					fluoxetine	1100 ng/mL In Blood (unspecified) @ Autopsy
949ai	58 y F				U	Unk	Int-A	2		
		morphine	1	1						
950ai	58 y F				U	Ingst+ Unk	Unk	2		
		morphine	1	1						
		alprazolam	2	2						
951ai	58 y M				U	Ingst	Int-A	2		
		methadone	1	1						
		acetaminophen/hydrocodone	2	2						
		alprazolam	3	3						
		tramadol	4	4						
952h	58 y M				A	Ingst	Unt-U	3		
		acetaminophen	1	1						
953	58 y M				A/C	Ingst	Int-S	3		
		methadone	1	1						
954ai	58 y M				A	Unk	Int-A	2		
		methadone	1	1						
		clonazepam	2	2						
		promethazine	3	3						
		pheniramine	4	4						
		ethanol	5	5						
955h	58 y F				A	Ingst	Int-S	1		
		acetaminophen	1	1					acetaminophen	34.5 mcg/mL In Blood (unspecified) @ Unknown
		amphetamine	2	2						
		cyclic antidepressant, unknown	3	3						
		oxycodone	4	4						
956ai	58 y M				U	Ingst	Int-A	2		
		acetaminophen/hydrocodone	1	1						
		oxycodone	2	2						
		hydromorphone	3	3						
957ai	58 y M				U	Ingst	Int-A	2		
		oxycodone	1	1						
958	58 y F				A	Ingst	Int-S	3		
		acetaminophen/hydrocodone	1	1					acetaminophen	399 mcg/mL In Blood (unspecified) @ 1 h (pe)
		morphine	2	2						
959ai	58 y M				A	Ingst+ Unk	Int-A	2		
		fentanyl	1	1						
		sertraline	2	2						
		ethanol	3	3						
960ai	58 y F				U	Ingst+ Unk	Int-A	2		
		fentanyl	1	1						
		fluoxetine	2	2						
961a	58 y M				A	Ingst	Int-S	2		
		salicylate	1	1						
		amphetamine	2	2						
962ha	58 y F				U	Ingst	Unk	2		
		acetaminophen	1	1					acetaminophen	30.5 mcg/mL In Blood (unspecified) @ Unknown
		acetaminophen	1	1					acetaminophen	31 mg/mL In Blood (unspecified) @ Autopsy
963	58 y F				A/C	Ingst	Unk	2		
		acetaminophen	1	1						
964h	59 y M				U	Ingst	Unk	3		
		salicylate	1	1					salicylate	28 mg/dL In Serum @ 8 h (pe)
		salicylate	1	1					salicylate	31 mg/dL In Serum @ 5 h (pe)
		salicylate	1	1					salicylate	45 mg/dL In Serum @ 5 m (pe)
965ai	59 y F				A	Ingst	Int-U	2		
		tramadol	1	1						
		diphenhydramine	2	2						
966	59 y F				A	Ingst	Unk	1		
		acetaminophen	1	1					acetaminophen	300 mcg/mL In Serum @ Unknown
967ha	59 y M				A/C	Ingst	Unk	1		
		oxycodone	1	1						
		morphine	2	2					morphine	0.036 mg/L In Blood (unspecified) @ Unknown
		nitroglycerin	3	3						
968ai	59 y F				U	Par	Int-A	2		
		hydromorphone	1	1						
969ai	59 y F				U	Ingst	Int-A	2		
		acetaminophen/hydrocodone	1	1						
		diazepam	2	2						
970	59 y M				A	Ingst	Unk	2		
		acetaminophen/hydrocodone	1	1						
971ai	59 y M				U	Ingst	Int-A	2		
		acetaminophen/hydrocodone	1	1						
972ai	59 y F				A	Ingst	Int-A	2		
		oxycodone	1	1						
		diazepam	2	2						
		alprazolam	3	3						
		amitriptyline	4	4						
		paroxetine	5	5						
973	59 y M				C	Ingst	Int-M	2		
		acetaminophen/hydrocodone	1	1						
974a	59 y F				A	Ingst	Int-A	1		
		methadone	1	1					methadone	0.4 mg/L In Blood (unspecified) @ Unknown
		cocaine	2	2						
		promethazine	3	3					promethazine	0.05 mg/L In Blood (unspecified) @ Autopsy
975ai	59 y M				A	Ingst+ Unk	Int-A	2		
		methadone	1	1						
		diazepam	2	2						
		ethanol	3	3						
976h	59 y F				C	Ingst	Int-M	2		
		acetaminophen	1	1					acetaminophen	53 mcg/mL In Serum @ Unknown
		ibuprofen	2	2						
977	59 y M				A	Ingst	Int-M	3		
		acetaminophen	1	1						
978ai	60 y F				A	Ingst	Int-A	2		
		methadone	1	1						
		diphenhydramine	2	2						
979pha	60 y M				U	Ingst	Int-S	2		
		acetaminophen/hydrocodone	1	1					dihydrocodeine/hydrocodol (free)	12 ng/mL In Blood (unspecified) @ Autopsy
		acetaminophen/hydrocodone	1	1					oxycodone (free)	14 ng/mL In Blood (unspecified) @ Autopsy
		acetaminophen/hydrocodone	1	1					hydrocodone (free)	260 ng/mL In Blood (unspecified) @ Autopsy
		acetaminophen/hydrocodone	1	1					acetaminophen	54 mcg/mL In Unknown @ Unknown
		acetaminophen/hydrocodone	1	1					acetaminophen	80 mcg/mL In Blood (unspecified) @ Autopsy
		angiotensin converting enzyme inhibitor	2	2						
		benzodiazepine	3	3					7-aminoclonazepam	180 ng/mL In Blood (unspecified) @ Autopsy
		benzodiazepine	3	3					alprazolam	54 ng/mL In Blood (unspecified) @ Autopsy
		benzodiazepine	3	3					clonazepam	94 ng/mL In Blood (unspecified) @ Autopsy
		carisoprodol	4	4					carisoprodol	14 mcg/mL In Blood (unspecified) @ Autopsy
		carisoprodol	4	4					meprobamate	19 mcg/mL In Blood (unspecified) @ Autopsy
		trazodone	5	5					trazodone	1.5 mcg/mL In Blood (unspecified) @ Autopsy
980pa	60 y M				A	Ingst+ Aspir	Int-S	2		
		acetaminophen/hydrocodone	1	1					hydrocodone (free)	69 mcg/mL In Serum @ Unknown
981ai	60 y F				U	Ingst	Int-A	2		
		methadone	1	1						
		ethanol	2	2						
982	60 y M				U	Ingst	Int-U	3		
		acetaminophen	1	1					acetaminophen	77 mcg/mL In Blood (unspecified) @ Unknown
983h	60 y M				A/C	Ingst	Int-S	2		
		tramadol	1	1						
		tizanidine	2	2						
		oxazepam	3	3						
		clonazepam	4	4						
		ibuprofen	5	5						
984a	60 y F				A/C	Ingst	Int-M	3		
		acetaminophen	1	1					acetaminophen	32.1 mg/mL In Blood (unspecified) @ Unknown
985h	60 y F				U	Ingst	Unk	1		
		acetaminophen *	2	1					acetaminophen	128 mcg/mL In Blood (unspecified) @ 26 h (pe)
		acetaminophen *	2	1					acetaminophen	254 mcg/mL In Blood (unspecified) @ 3 h (pe)
		acetaminophen *	2	1					acetaminophen	453 mcg/mL In Blood (unspecified) @ 0 h (pe)
		acetaminophen *	2	1					acetaminophen	454 mcg/mL In Blood (unspecified) @ 65 h (pe)
		acetaminophen *	2	1					acetaminophen	497 mcg/mL In Blood (unspecified) @ 45 h (pe)
		acetaminophen *	2	1					acetaminophen	504 mcg/mL In Blood (unspecified) @ 41 h (pe)
		acetaminophen/diphenhydramine *	1	1						
986ai	61 y M				A	Ingst	Int-U	2		
		tramadol	1	1						
		methadone	2	2						
		promethazine	3	3						
987ai	61 y F				U	Ingst	Int-A	2		
		acetaminophen/hydrocodone	1	1						
		temazepam	2	2						
988pa	61 y F				A/C	Ingst	Int-S	1		
		acetaminophen/oxycodone	1	1					acetaminophen	2 mcg/mL In Unknown @ Unknown
		acetaminophen/oxycodone	1	1					dihydrocodeine/hydrocodol (free)	47 ng/mL In Blood (unspecified) @ Autopsy
		acetaminophen/oxycodone	1	1					hydrocodone (free)	66 ng/mL In Blood (unspecified) @ Autopsy
		methamphetamine	2	2					methamphetamine	5.7 ng/mL In Blood (unspecified) @ Autopsy
		diazepam	3	3					diazepam	360 ng/mL In Blood (unspecified) @ Autopsy
		diazepam	3	3					nordiazepam	390 ng/mL In Blood (unspecified) @ Autopsy
		temazepam	4	4					temazepam	42 ng/mL In Blood (unspecified) @ Autopsy
		oxazepam	5	5					oxazepam	23 ng/mL In Blood (unspecified) @ Autopsy
989	61 y M				U	Ingst	Int-S	1		
		acetaminophen/hydrocodone	1	1					acetaminophen	722 mcg/mL In Serum @ Unknown
		acetaminophen	2	2					salicylate	17 mg/dL In Serum @ Unknown
		acetaminophen/caffeine/salicylate	3	3						
		ethanol	4	4						
990h	61 y F				U	Ingst	Int-S	1		
		acetaminophen	1	1					acetaminophen	109 mcg/mL In Serum @ Unknown
		diphenhydramine	2	2						
991ai	61 y F				U	Ingst	Int-A	2		
		acetaminophen/hydrocodone	1	1						
		citalopram	2	2						
		cyclobenzaprine	3	3						
992ai	61 y F				A	Ingst	Int-S	2		
		hydrocodone	1	1						
		mirtazapine	2	2						
		acetaminophen	3	3						
993ai	62 y F				A	Ingst	Unk	2		
		methadone	1	1						
		oxycodone	2	2						
		amitriptyline	3	3						
		oxcarbazepine	4	4						
		paroxetine	5	5						
		mirtazapine	6	6						
		metoprolol	7	7						
994a	62 y M				A	Ingst	Int-S	1		
		acetaminophen	1	1						
		ethanol	2	2					ethanol	100 mg/dL In Blood (unspecified) @ 0.5 h (pe)
		ethanol	2	2					acetaminophen	494 mcg/mL In Blood (unspecified) @ 0.5 h (pe)
995ai	62 y M				A	Ingst	Int-S	2		
		oxycodone	1	1						
		codeine	2	2						
		acetaminophen	3	3						
		ethanol	4	4						
996	62 y M				A	Ingst	Int-S	2		
		acetaminophen/hydrocodone	1	1					acetaminophen	86 ng/mL In Blood (unspecified) @ Unknown
		benzodiazepine	2	2						
997ai	62 y M				U	Ingst+ Unk	Int-A	2		
		acetaminophen/hydrocodone	1	1						
		morphine	2	2						
998ai	62 y M				U	Ingst	Int-A	2		
		methadone	1	1						
		alprazolam	2	2						
999h	62 y F				C	Ingst	Int-A	2		
		acetaminophen/hydrocodone	1	1						
1000ai	62 y F				A	Ingst	Int-A	2		
		oxycodone	1	1						
		alprazolam	2	2						
		sertraline	3	3						
1001h	62 y F				A	Ingst	Int-S	3		
		acetaminophen/hydrocodone	1	1					acetaminophen	252 mcg/mL In Serum @ Unknown
		clonazepam	2	2						
		ethanol	3	3					ethanol	0.22 g/dL In Serum @ Unknown
1002h	63 y F				A/C	Ingst	Int-M	2		
		acetaminophen/hydrocodone	1	1					acetaminophen	11 mcg/mL In Serum @ Unknown
1003pha	63 y M				U	Ingst	Unk	1		
		acetaminophen/opioid	1	1					acetaminophen	43 mg/L In Serum @ Unknown
1004ph	63 y F				A/C	Ingst	Int-U	2		
		acetaminophen/hydrocodone	1	1						
1005pha	63 y F				U	Ingst+ Unk	Int-U	1		
		acetaminophen	1	1					acetaminophen	276 mg/L In Blood (unspecified) @ Unknown
		opioid	2	2					hydrocodone	0.4 mg/L In Blood (unspecified) @ Unknown
		opioid	2	2					hydrocodone (free)	0.48 mg/L In Blood (unspecified) @ Unknown
		benzodiazepine	3	3					midazolam	0.02 mg/L In Blood (unspecified) @ Unknown
1006ai	63 y M				A	Unk	Int-A	2		
		methadone	1	1						
		sertraline	2	2						
1007ai	63 y M				A	Unk	Int-A	2		
		methadone	1	1						
		phencyclidine	2	2						
1008ai	63 y F				U	Ingst	Unk	2		
		acetaminophen/hydrocodone	1	1						
1009ai	63 y M				U	Ingst	Int-A	2		
		methadone	1	1						
		ethanol	2	2						
1010ph	63 y F				A/C	Ingst	Int-S	2		
		acetaminophen/butalbital/caffeine	1	1					acetaminophen	190 mcg/mL In Blood (unspecified) @ Unknown
		melatonin	2	2						
1011	63 y F				C	Ingst	Unt-M	1		
		acetaminophen	1	1						
		ethanol	2	2						
1012ha	64 y F				A	Ingst	Int-S	2		
		acetaminophen	1	1					acetaminophen	20 mcg/mL In Blood (unspecified) @ 1 h (pe)
		ethanol	2	2						
1013	64 y M				U	Ingst	Unk	1		
		acetaminophen	1	1					acetaminophen	59 mcg/mL In Blood (unspecified) @ 1 h (pe)
		ethanol	2	2						
1014ai	64 y M				U	Ingst	Int-A	2		
		oxycodone	1	1						
1015h	64 y F				A/C	Ingst	Int-M	2		
		acetaminophen	1	1					acetaminophen	21 mcg/mL In Serum @ 10 h (pe)
		acetaminophen	1	1					acetaminophen	37.8 mcg/mL In Serum @ 5 m (pe)
		acetaminophen/hydrocodone *	2	2						
		carisoprodol *	3	2						
		salicylate *	4	2					salicylate	13 mg/dL In Serum @ 6 h (pe)
		salicylate *	4	2					salicylate	18.7 mg/dL In Serum @ 5 m (pe)
		ethanol	5	4					ethanol	24 mg/dL In Serum @ 5 m (pe)
1016	64 y F				C	Ingst	Int-M	2		
		acetaminophen/hydrocodone	1	1						
		acetaminophen	2	2					acetaminophen	161 mcg/mL In Blood (unspecified) @ Unknown
1017ai	65 y M				U	Unk	Unk	2		
		morphine	1	1						
1018	65 y M				C	Ingst	Int-M	3		
		salicylates in combination	1	1						
1019ai	65 y M				A	Ingst	Int-A	2		
		oxycodone	1	1						
		trazodone	2	2						
		venlafaxine	3	3						
		diphenhydramine	4	4						
1020	65 y F				A	Ingst	Int-S	2		
		acetaminophen/oxycodone	1	1					acetaminophen	293 mcg/mL In Blood (unspecified) @ Unknown
		acetaminophen/hydrocodone	2	2						
		morphine (extended release)	3	3						
		hydrocodone/ibuprofen	4	4						
1021h	65 y M				A/C	Ingst	Unt-T	1		
		colchicine	1	1						
1022	65 y F				A/C	Ingst	Unt-M	1		
		acetaminophen/hydrocodone	1	1					acetaminophen	115 mcg/mL In Blood (unspecified) @ Unknown
1023ph	65 y F				A/C	Ingst	Unk	2		
		oxycodone (extended release)	1	1						
		benzodiazepine	2	2						
1024h	66 y F				A/C	Ingst	Unt-T	2		
		acetaminophen	1	1					acetaminophen	70 mcg/mL In Blood (unspecified) @ Unknown
1025ai	66 y M				U	Ingst	Int-A	2		
		acetaminophen/hydrocodone	1	1						
		skeletal muscle relaxant	2	2						
		diazepam	3	3						
1026a	66 y F				A	Ingst	Int-S	1		
		acetaminophen/hydrocodone	1	1					hydrocodone	0.35 mg/L In Plasma @ Unknown
		acetaminophen/hydrocodone	1	1					acetaminophen	12 mg/L In Plasma @ Unknown
		diphenhydramine	2	2					diphenhydramine	2.1 mg/L In Plasma @ Unknown
		alprazolam	3	3					alprazolam	0.02 mg/L In Plasma @ Unknown
		fluoxetine	4	4					fluoxetine	0.31 mg/L In Plasma @ Unknown
		fluoxetine	4	4					norfluoxetine	0.49 mg/L In Plasma @ Unknown
1027pha	67 y M				A	Ingst	Int-S	1		
		acetaminophen/opioid	1	1					hydrocodone	100 ng/mL In Blood (unspecified) @ 10 h (pe)
		acetaminophen/opioid	1	1					codeine	120 ng/mL In Blood (unspecified) @ 10 h (pe)
		acetaminophen/opioid	1	1					acetaminophen	127 mg/L In Blood (unspecified) @ 10 h (pe)
		acetaminophen/opioid	1	1					morphine	29 ng/mL In Blood (unspecified) @ 10 h (pe)
1028h	67 y F				U	Ingst	Unk	1		
		acetaminophen	1	1					acetaminophen	163 mcg/mL In Blood (unspecified) @ Unknown
		ethanol	2	2					ethanol	12 mg/dL In Blood (unspecified) @ Unknown
1029	67 y F				U	Unk	Int-S	1		
		salicylate	1	1					salicylate	110 mg/dL In Blood (unspecified) @ Unknown
		drug, unknown	2	2						
1030ai	68 y F				U	Derm	Int-A	2		
		droperidol/fentanyl	1	1						
1031	68 y F				U	Ingst	Int-S	1		
		salicylate	1	1					salicylate	118 mg/dL In Serum @ Unknown
		codeine/terpin hydrate	2	2						
		dextromethorphan/salicylate	3	3						
		antibiotic, macrolide	4	4						
		cephalexin	5	5						
		lysozyme	6	6						
		antihistamine/decongestant	7	7						
		tetrahydropalmatine	8	8						
		eprazinone	9	9						
		analgesics, unknown	10	10						
		codeine	11	11						
		diphenhydramine	12	12						
		codeine	13	13						
		analgesics, unknown	14	14						
		piracetam	15	15						
		N-acetylcsysteine	16	16						
		cefixime	17	17						
		ampicillin	18	18						
1032h	68 y F				A	Ingst	Unk	3		
		salicylate	1	1					salicylate	25 mg/dL In Serum @ 3.25 h (pe)
		salicylate	1	1					salicylate	40 mg/dL In Serum @ 0.25 h (pe)
1033hi	68 y M				A	Ingst	Int-S	1		
		salicylate	1	1					salicylate	110 mg/dL In Serum @ 2.5 h (pe)
1034	68 y F				U	Ingst	Unt-T	3		
		acetaminophen/hydrocodone	1	1					acetaminophen	210 mcg/mL In Blood (unspecified) @ 6 h (pe)
		acetaminophen/hydrocodone	1	1					acetaminophen	257 mcg/mL In Blood (unspecified) @ 10 m (pe)
		acetaminophen/hydrocodone	1	1					acetaminophen	287 mcg/mL In Blood (unspecified) @ 15 h (pe)
		acetaminophen	2	2						
1035	70 y F				C	Ingst	Unt-M	2		
		acetaminophen	1	1						
1036	70 y F				U	Ingst	Unk	1		
		salicykates in combination	1	1						
1037ha	70 y F				A/C	Ingst	Int-S	2		
		salicylate	1	1					salicylate	27.3 mg/dL In Blood (unspecified) @ 16 h (pe)
		salicylate	1	1					salicylate	56 mg/dL In Blood (unspecified) @ 12 h (pe)
		salicylate	1	1					salicylate	72 mg/dL In Blood (unspecified) @ 1 h (pe)
1038p	70 y M				A	Ingst	Int-S	2		
		tramadol	1	1						
		hydroxyzine	2	2						
1039a	71 y F				U	Ingst	Int-S	3		
		acetaminophen/hydrocodone	1	1					acetaminophen	48 mcg/mL In Blood (unspecified) @ 1 h (pe)
1040ph	71 y M				A	Ingst	Int-S	1		
		methadone	1	1						
		citalolpram	2	2						
		bupropion	3	3						
		acetaminophen/hydrocodone	4	4					acetaminophen	133 mcg/mL In Blood (unspecified) @ Unknown
1041ai	72 y M				U	Ingst	Int-A	2		
		acetaminophen/hydrocodone	1	1						
1042ha	72 y F				U	Ingst	Int-S	1		
		oxycodone	1	1					oxycodone	0.474 mg/L In Blood (unspecified) @ Unknown
1043	72 y M				C	Ingst	Unk	2		
		acetaminophen	1	1						
1044ai	73 y F				A	Ingst	Int-S	2		
		hydrocodone	1	1						
		citalopram	2	2						
		acetaminophen	3	3						
1045ai	73 y F				U	Ingst	Int-A	2		
		acetaminophen/hydrocodone	1	1						
		hydromorphone	2	2						
1046h	73 y F				C	Ingst	Int-U	2		
		acetaminophen/oxycodone	1	1						
		acetaminophen	2	2					acetaminophen	4 mcg/mL In Blood (unspecified) @ Unknown
		salicylate	3	3					salicylate	6.5 mg/dL In Blood (unspecified) @ Unknown
		ibuprofen	4	4						
1047	73 y M				A/C	Ingst	Int-S	2		
		acetaminophen	1	1					acetaminophen	103 mg/L In Serum @ Unknown
		antihypertensive	2	2						
		trazodone	3	3						
1048	73 y F				C	Ingst	Int-U	3		
		acetaminophen	1	1					acetaminophen	3.38 mcg/mL In Blood (unspecified) @ Unknown
		acetaminophen/oxycodone	2	2						
		warfarin	3	3						
1049h	73 y F				U	Ingst	Int-S	1		
		acetaminophen/hydrocodone	1	1						
1050ai	74 y M				A	Ingst	Int-A	2		
		methadone	1	1						
		oxycodone	2	2						
		tramadol	3	3						
1051ai	75 y F				U	Ingst	Int-A	2		
		tramadol	1	1						
		ethanol	2	2						
1052ph	75 y F				A/C	Ingst	Int-S	3		
		tramadol	1	1						
		ethanol	2	2						
1053	77 y F				C	Ingst	Int-M	1		
		acetaminophen/hydrocodone	1	1						
		acetaminophen/caffeine/salicylate	2	2						
		acetaminophen	3	3						
1054h	77 y M				C	Ingst	Unt-T	3		
		oxycodone	1	1						
1055h	77 y F				U	Ingst	Int-S	1		
		acetaminophen/hydrocodone	1	1					acetaminophen	428 mcg/mL In Blood (unspecified) @ Unknown
		ethanol	2	2					ethanol	114 mg/dL In Blood (unspecified) @ Unknown
1056	77 y F				A/C	Ingst	Int-S	2		
		acetaminophen/hydrocodone	1	1					acetaminophen	76.5 mcg/mL In Blood (unspecified) @ Unknown
		ethanol	2	2					ethanol	2 mg/dL In Blood (unspecified) @ Unknown
		primidone	3	3					phenobarbital	1 mcg/mL In Blood (unspecified) @ Unknown
[1057ha]	78 y M				C	Ingst	Unt-T	2		
		colchicine	1	1					colchicine	4 ng/mL In Blood (unspecified) @ 60 m (pe)
1058ai	78 y F				U	Ingst	Int-A	2		
		acetaminophen/hydrocodone	1	1						
1059ph	78 y F				U	Unk	Unk	2		
		fentanyl	1	1						
		hydromorphone	2	2						
		fentanyl (transdermal)	3	3						
1060a	78 y F				A	Ingst	Int-S	3		
		salicylate	1	1						
1061	78 y M				U	Ingst	Unk	3		
		acetaminophen	1	1					acetaminophen	13 mcg/mL In Blood (unspecified) @ Unknown
		ethanol	2	2						
1062a	78 y F				A/C	Ingst	Int-S	2		
		acetaminophen/butalbital/caffeine	1	1					butalbital	33 mcg/mL In Blood (unspecified) @ Unknown
		acetaminophen/butalbital/caffeine	1	1					acetaminophen	67 mcg/mL In Blood (unspecified) @ Unknown
1063	78 y F				A	Ingst	Int-S	2		
		hydromorphone	1	1						
		acetaminophen/hydrocodone	2	2						
1064p	79 y F				A/C	Ingst	Int-S	2		
		acetaminophen/hydrocodone	1	1						
1065	79 y F				A/C	Ingst	Int-S	2		
		oxycodone (extended release)	1	1						
		fentanyl	2	2						
		acetaminophen	3	3						
1066	80 y F				A/C	Ingst	Int-M	1		
		acetaminophen	1	1						
1067	81 y F				A	Ingst	Int-U	1		
		acetaminophen/hydrocodone	1	1					acetaminophen	74 mcg/mL In Serum @ Unknown
1068	81 y F				C	Ingst	Int-S	2		
		acetaminophen/hydrocodone	1	1					acetaminophen	143 mcg/mL In Serum @ Unknown
		acetaminophen/hydrocodone	1	1					acetaminophen	44 mcg/mL In Serum @ Unknown
		acetaminophen/hydrocodone	1	1					acetaminophen	77.3 mcg/mL In Serum @ Unknown
1069	81 y F				A	Ingst	Int-S	1		
		salicylate	1	1					salicylate	121 mg/dL In Blood (unspecified) @ Unknown
1070	82 y M				A/C	Ingst	Int-S	3		
		acetaminophen/codeine	1	1					acetaminophen	55 mg/L In Serum @ 1 h (pe)
1071p	83 y F				A/C	Ingst	Int-U	3		
		morphine	1	1					morphine (free)	350 ng/mL In Blood (unspecified) @ Autopsy
		citalopram	2	2					citalopram	740 ng/mL In Blood (unspecified) @ Autopsy
		lorazepam	3	3					lorazepam	18 ng/mL In Blood (unspecified) @ Autopsy
		clonazepam	4	4					7-aminoclonazepam	32 ng/mL In Blood (unspecified) @ Autopsy
1072	84 y F				U	Ingst	Unk	2		
		acetaminophen	1	1					acetaminophen	106 mcg/mL In Blood (unspecified) @ 24 h (pe)
1073ha	84 y F				A	Ingst	Int-S	1		
		salicylate	1	1					salicylate	1000 mcg/mL In Blood (unspecified) @ Autopsy
		salicylate	1	1					salicylate	84 mg/dL In Blood (unspecified) @ Unknown
		doxepin	2	2					doxepin	1000 ng/mL In Blood (unspecified) @ Autopsy
		doxepin	2	2					desmethyldoxepin	320 ng/mL In Blood (unspecified) @ Autopsy
		ibuprofen	3	3						
1074	84 y F				A	Ingst	Int-S	1		
		acetaminophen	1	1					acetaminophen	133.9 mcg/mL In Serum @ Unknown
		acetaminophen	1	1					acetaminophen	232.9 mcg/mL In Serum @ Unknown
		salicylate	2	2					salicylate	39.4 mg/dL In Serum @ Unknown
		salicylate	2	2					salicylate	61 mg/dL In Serum @ Unknown
1075	85 y F				A	Ingst	Int-S	3		
		acetaminophen	1	1						
1076	86 y F				U	Ingst	Int-S	3		
		acetaminophen	1	1					acetaminophen	428 mcg/mL In Serum @ Unknown
		acetaminophen/hydrocodone	2	2						
1077h	86 y F				C	Ingst	Unt-G	1		
		acetaminophen	1	1					acetaminophen	227 mcg/mL In Blood (unspecified) @ Unknown
		acetaminophen	1	1					acetaminophen	317 mcg/mL In Blood (unspecified) @ Unknown
1078h	87 y M				A/C	Ingst	Unt-T	3		
		morphine (extended release)	1	1						
1079h	87 y F				A	Ingst	Unk	3		
		acetaminophen/hydrocodone	1	1					acetaminophen	248.8 mcg/mL In Blood (unspecified) @ 1 h (pe)
1080h	87 y F				A/C	Ingst	Int-S	3		
		tramadol	1	1						
1081h	88 y F				A/C	Ingst	Unt-T	1		
		acetaminophen	1	1						
1082	91 y F				U	Ingst	Int-S	1		
		acetaminophen/hydrocodone	1	1					acetaminophen	806 mcg/mL In Serum @ Unknown
		ethanol	2	2						
1083	94 y M				A	Ingst	Int-S	2		
		acetaminophen/hydrocodone	1	1						
1084a	94 y M				A	Ingst	Int-U	2		
		acetaminophen	1	1						
		opioid	2	2						
[1085a]	11 m M				A	Ingst	Unt-G	1		
		salicylate	1	1					salicylate	850 mg/L In Blood (unspecified) @ 7 h (pe)
1086pa	18 m F				U	Ingst	Unk	1		
		hydrocodone	1	1					hydrocodone (free)	240 ng/mL In Blood (unspecified) @ Autopsy
		hydrocodone	1	1					dihydrocodeine/hydrocodol (free)	31 ng/mL In Blood (unspecified) @ Autopsy
		hydrocodone	1	1					dextromethorphan	8.8 ng/mL In Blood (unspecified) @ Autopsy
		acetaminophen	2	2					acetaminophen	22 mcg/mL In Blood (unspecified) @ Autopsy
		alprazolam	3	3					alprazolam	18 ng/mL In Blood (unspecified) @ Autopsy
		dihydrocodeine	4	4					dihydrocodeine/hydrocodol (free)	31 ng/mL In Blood (unspecified) @ Autopsy
1087pa	19 m M				A	Ingst	Oth-M	1		
		methadone	1	1					methadone	0.8 mg/L In Blood (unspecified) @ Autopsy
[1088]	19 m F				A	Ingst	Unt-G	1		
		methadone	1	1					eddp (2-ethylidene-1,5-dimethyl-3,3-diphenyl pyrrolidine)	13 ng/mL In Blood (unspecified) @ 1 d (pe)
		methadone	1	1					methadone	248 ng/mL In Blood (unspecified) @ 1 d (pe)
1089p	23 m M				A	Ingst	Oth-M	2		
		hydromorphone	1	1						
1090p	30 + y F				A	Ingst	Int-S	2		
		methadone	1	1						
1091p	40 + y F				A	Ingst	Int-U	3		
		methadone	1	1					eddp (2-ethylidene-1,5-dimethyl-3,3-diphenyl pyrrolidine)	2256 ng/mL In Urine (quantitative only) @ Autopsy
		methadone	1	1					methadone	3457 ng/mL In Urine (quantitative only) @ Autopsy
		alprazolam	2	2						
1092pha	Unknown adult (> = 20 yrs) F				U	Unk	Unk	2		
		morphine	1	1					morphine	170 ng/mL In Blood (unspecified) @ Autopsy
		morphine	1	1					6-monoacetylmorphine	41 ng/mL In Urine (quantitative only) @ Autopsy
		methamphetamine	2	2					methamphetamine	1100 ng/mL In Blood (unspecified) @ Autopsy
		hydroxyzine	3	3						
		ethanol	4	4					ethanol	199 mg/dL In Blood (unspecified) @ Autopsy
1093pi	Unknown adult (> = 20 yrs) F				A	Ingst	Int-A	2		
		fentanyl (transdermal)	1	1						
See Also case 5, 9, 14, 33, 39, 40, 41, 43, 48, 51, 76, 84, 88, 90, 92, 102, 105, 110, 111, 116, 120, 123, 124, 153, 183, 193, 243, 253, 257, 273, 275, 280, 301, 310, 347, 349, 358, 362, 381, 393, 1105, 1108, 1112, 1117, 1125, 1128, 1129, 1147, 1151, 1154, 1159, 1162, 1166, 1177, 1178, 1180, 1185, 1189, 1196, 1202, 1204, 1206, 1207, 1214, 1217, 1223, 1227, 1241, 1248, 1249, 1250, 1251, 1252, 1256, 1257, 1262, 1263, 1264, 1266, 1269, 1270, 1271, 1275, 1277, 1278, 1281, 1282, 1291, 1292, 1294, 1295, 1298, 1300, 1323, 1325, 1327, 1334, 1340, 1345, 1346, 1350, 1351, 1355, 1357, 1366, 1382, 1387, 1395, 1403, 1410, 1422, 1424, 1425, 1426, 1434, 1436, 1437, 1439, 1445, 1464, 1471, 1484, 1486, 1494, 1498, 1500, 1506, 1510, 1514, 1517, 1531, 1537, 1539, 1540, 1544, 1546, 1547, 1548, 1549, 1550, 1553, 1555, 1563, 1567, 1577, 1578, 1580, 1584, 1586, 1587, 1588, 1590, 1592, 1594, 1597, 1600, 1601, 1607, 1609, 1610, 1612, 1617, 1618, 1620, 1623, 1627, 1629, 1634, 1635, 1647, 1665, 1672, 1684, 1685, 1690, 1697, 1701, 1706, 1709, 1710, 1711, 1714, 1715, 1716, 1717, 1722, 1723, 1725, 1726, 1727, 1729, 1732, 1734, 1739, 1740, 1744, 1745, 1750, 1751, 1753, 1754, 1755, 1756, 1758, 1762, 1763, 1765, 1766, 1770, 1775, 1776, 1779, 1784, 1787, 1788, 1791, 1793, 1797, 1798, 1799, 1801, 1804, 1806, 1807, 1809, 1810, 1811, 1812, 1818, 1820, 1822, 1825, 1830, 1831, 1834, 1838, 1840, 1841, 1843, 1844, 1845, 1846, 1850, 1851, 1856, 1859, 1863, 1865, 1869, 1871, 1875, 1878, 1879, 1881, 1882, 1892, 1893, 1894, 1898, 1900, 1902, 1909, 1913, 1915, 1917, 1918, 1920, 1923, 1925, 1926, 1927, 1928, 1930, 1933, 1934, 1935, 1941, 1942, 1943, 1944, 1947, 1949, 1954, 1957, 1958, 1960, 1964, 1972, 1973, 1976, 1978, 1985, 1989, 1990, 1991, 2002, 2007, 2015, 2019, 2021, 2023, 2030, 2034, 2036, 2037, 2039, 2041, 2049, 2050, 2051, 2059, 2061, 2064, 2066, 2069, 2070, 2072, 2076, 2091, 2096, 2103, 2106										
**Anesthetics**										
1094ai	24 y M				U	Inhal	Unk	2		
		nitrous oxide	1	1						
1095p	25 y M				A	Ingst	Int-U	1		
		sevoflurane	1	1						
[1096pa]	37 y M				A	Inhal	Int-S	1		
		sevoflurane	1	1					phenytoin	12 mcg/mL In Blood (unspecified) @ Autopsy
1097p	40 y F				U	Ingst	Int-M	1		
		lidocaine	1	1						
1098ai	60 y M				A	Inhal	Int-A	2		
		isoflurane	1	1						
1099	66 y F				A	Par	AR-D	1		
		lidocaine	1	1						
[1100]	77 y F				A	Par	Unt-T	1		
		lidocaine	1	1						
1101a	83 y F				A	Par	Unt-T	1		
		lidocaine	1	1						
[1102pha]	13 m F				A	Ingst	Unt-G	1		
		lidocaine	1	1						
See Also case 235, 244, 437, 572, 654, 817, 1744, 1752, 1775, 1990, 2083										
**Anticholinergic Drugs**										
1103	32 y M				A/C	Ingst	Int-S	1		
		benztropine	1	1						
1104ai	35 y M				U	Ingst	Unk	2		
		benztropine	1	1						
		fluoxetine	2	2						
		ethanol	3	3						
1105a	50 y F				A	Ingst	Int-S	2		
		benztropine	1	1					benztropine mesylate	0.44 mg/L In Plasma @ Unknown
		opioid	2	2						
See Also case 458, 699, 774, 1204, 1650, 1788										
**Anticoagulants**										
1106	59 y F				U	Ingst	AR-D	2		
		rivaroxaban	1	1						
1107h	61 y M				A/C	Ingst	AR-D	3		
		rivaroxaban	1	1						
1108	63 y M				U	Ingst	Int-S	1		
		warfarin	1	1						
		ethanol	2	2					ethanol	192 mg/dL In Blood (unspecified) @ Unknown
		salicylate	3	3						
[1109h]	66 y M				C	Ingst	AR-D	3		
		rivaroxaban	1	1						
1110h	70 y M				C	Ingst	AR-D	3		
		dabigatran	1	1						
[1111]	73 y M				A/C	Ingst	Unt-T	3		
		enoxaparin	1	1						
1112h	78 y F				A	Ingst	Int-S	2		
		warfarin	1	1						
		metoprolol	2	2						
		tramadol	3	3						
		primidone	4	4						
		ciprofloxacin	5	5						
		docusate	6	6						
		furosemide	7	7						
		celecoxib	8	8						
		amoxicillin	9	9						
		ondansetron	10	10						
		esomeprazole	11	11						
1113	82 y M				C	Ingst	AR-D	2		
		rivaroxaban	1	1						
1114h	85 y F				C	Ingst	AR-D	1		
		dabigatran	1	1						
1115	90 y M				A	Ingst	AR-D	3		
		dabigatran	1	1						
See Also case 423, 661, 1048, 1140, 1345, 1346, 1387, 1394, 1447, 1449, 1452, 1453, 1466, 1471, 1474, 1477, 1482, 1612, 1619, 1660										
**Anticonvulsants**										
1116	28 y M				A	Ingst	Int-S	1		
		lamotrigine	1	1						
		venlafaxine	2	2						
1117p	33 y F				A	Ingst	Int-S	2		
		gabapentin	1	1						
		tramadol	2	2						
		venlafaxine	3	3						
1118ph	35 y M				A	Ingst	Int-S	2		
		phenytoin	1	1						
		venlafaxine	2	2						
		clonidine	3	3						
		lamotrigine	4	4						
1119	35 y M				A/C	Ingst	Int-S	2		
		lamotrigine	1	1						
		amphetamine/dextroamphetamine	2	2						
1120	37 y F				A/C	Ingst	Int-U	2		
		gabapentin	1	1						
		trazodone	2	2						
1121ph	41 y M				A/C	Ingst	Int-S	1		
		lamotrigine	1	1						
		cardiac glycoside	2	2						
		fluoxetine	3	3						
1122h	41 y F				U	Ingst	Int-S	2		
		valproic acid	1	1					valproic acid	21 mcg/mL In Serum @ 17 h (pe)
		quetiapine	2	2						
		sertraline	3	3						
1123p	42 y F				U	Ingst	Int-S	3		
		gabapentin	1	1						
1124p	44 y F				A/C	Ingst	Int-S	2		
		gabapentin	1	1						
		clonazepam	2	2						
1125a	45 y F				A/C	Ingst	Int-S	1		
		carbamazepine	1	1					carbamazepine	30.9 Other (see abst) In Blood (unspecified) @ 1 h (pe)
		carbamazepine	1	1					carbamazepine	48.6 Other (see abst) In Blood (unspecified) @ 9 h (pe)
		bupropion	2	2						
		tramadol	3	3						
		olanzapine	4	4						
		lorazepam	5	5						
1126ai	46 y F				A	Ingst	Int-S	2		
		lamotrigine	1	1						
		paroxetine	2	2						
1127ai	52 y F				A	Ingst	Int-S	2		
		lamotrigine	1	1						
		venlafaxine	2	2						
		trazodone	3	3						
1128h	53 y F				A	Ingst	Int-S	1		
		gabapentin	1	1						
		acetaminophen/butalbital/caffeine	2	2						
		atenolol	3	3						
1129	53 y M				A	Ingst	Int-S	3		
		gabapentin	1	1						
		fluoxetine	2	2						
		naproxen	3	3						
		tetracycline	4	4						
		sertraline	5	5						
		allopurinol	6	6						
1130a	53 y M				A	Ingst	Int-S	2		
		gabapentin	1	1						
		alprazolam	2	2						
		ethanol (non-beverage)	3	3					ethanol	256 mg/dL In Plasma @ Unknown
1131a	54 y M				A/C	Ingst	Int-S	2		
		valproic acid	1	1					valproic acid	125 mcg/mL In Blood (unspecified) @ 1 d (pe)
		valproic acid	1	1					valproic acid	136.4 mcg/mL In Blood (unspecified) @ Autopsy
		duloxetine	2	2						
		quetiapine	3	3						
1132ha	55 y F				A	Ingst	Int-S	1		
		lamotrigine	1	1					lamotrigine	28 mg/L In Blood (unspecified) @ Autopsy
		topiramate	2	2					topiramate	4 mg/L In Blood (unspecified) @ Autopsy
		clonazepam	3	3						
1133h	58 y F				A/C	Ingst	Int-S	2		
		lamotrigine	1	1						
		sertraline	2	2						
		alprazolam	3	3						
1134ai	60 y M				A	Ingst	Int-S	2		
		lamotrigine	1	1						
		metoprolol	2	2						
		chlorpromazine	3	3						
		citalopram	4	4						
1135	61 y F				A/C	Ingst	Int-S	2		
		gabapentin	1	1						
		lisinopril	2	2						
[1136a]	63 y F				A	Ingst	Int-S	1		
		valproic acid	1	1					valproic acid	970 mg/L In Blood (unspecified) @ Unknown
1137	64 y F				C	Ingst	Int-S	2		
		valproic acid (extended release)	1	1						
		quetiapine	2	2						
		benzodiazepine	3	3						
		ziprasidone	4	4						
1138	66 y F				A	Ingst	Int-S	3		
		carbamazepine	1	1					carbamazepine	19 mg/L In Serum @ Unknown
		carbamazepine	1	1					carbamazepine	27 mg/L In Serum @ Unknown
		carbamazepine	1	1					carbamazepine	32 mg/L In Serum @ Unknown
		carbamazepine	1	1					carbamazepine	9.4 mg/L In Serum @ Unknown
1139	67 y M				A	Ingst+ Aspir	Int-S	3		
		lamotrigine	1	1						
		zonisamide	2	2						
		lorazepam	3	3						
		escitalopram	4	4						
		lovastatin	5	5						
1140	79 y M				C	Ingst	AR-D	3		
		phenytoin	1	1						
		warfarin	2	2						
See Also case 48, 87, 142, 301, 400, 407, 428, 433, 471, 568, 602, 620, 625, 638, 648, 649, 711, 714, 727, 770, 774, 783, 795, 833, 854, 863, 898, 993, 1031, 1056, 1112, 1144, 1145, 1155, 1159, 1165, 1170, 1180, 1189, 1194, 1203, 1210, 1228, 1234, 1267, 1270, 1279, 1294, 1295, 1298, 1340, 1342, 1346, 1348, 1352, 1354, 1366, 1369, 1372, 1376, 1382, 1405, 1408, 1416, 1424, 1437, 1444, 1483, 1500, 1510, 1531, 1565, 1570, 1593, 1594, 1597, 1599, 1621, 1627, 1633, 1636, 1638, 1641, 1650, 1656, 1818, 1835, 1841, 1855, 1956, 1978, 1987, 1998, 2015, 2021										
**Antidepressants**										
1141	3 y M				A	Ingst	Unt-G	2		
		amitriptyline	1	1						
		cyclobenzaprine	2	2						
1142	3 y F				A	Ingst	Unt-G	1		
		bupropion	1	1						
1143	17 y F				A	Ingst	Int-S	3		
		sertraline	1	1						
1144p	17 y M				A	Ingst	Int-S	1		
		amitriptyline	1	1						
		gabapentin	2	2						
1145	19 y F				A	Ingst	Int-S	1		
		doxepin	1	1						
		valproic acid	2	2						
		alprazolam	3	3						
		diazepam	4	4						
1146p	19 y F				A	Ingst	Int-S	3		
		venlafaxine	1	1						
		quetiapine	2	2						
		ethanol	3	3					ethanol	218 mg/dL In Blood (unspecified) @ Unknown
1147pa	20 y F				A	Ingst	Int-S	1		
		citalopram	1	1						
		acetaminophen/hydrocodone	2	2						
		zolpidem	3	3						
		clonazepam	4	4						
		diphenhydramine	5	5						
1148h	20 y F				A	Ingst	Int-S	1		
		bupropion	1	1						
		diazepam	2	2						
		amitriptyline	3	3						
		citalopram	4	4						
1149h	20 y F				A/C	Ingst	Int-S	1		
		antidepressant	1	1					bupropion	4.7 mg/L In Blood (unspecified) @ Autopsy
1150	20 y F				A/C	Ingst	Int-S	1		
		bupropion	1	1						
1151a	20 y F				U	Ingst	Int-S	2		
		venlafaxine	1	1						
		metaxalone	2	2						
		acetaminophen/hydrocodone	3	3					acetaminophen	16 mg/L In Whole Blood @ 5 h (pe)
1152a	20 y F				A/C	Ingst	Int-S	1		
		fluvoxamine *	2	1					fluvoxamine	20000 ng/mL In Blood (unspecified) @ Autopsy
		quetiapine *	1	1						
1153ph	20 y F				A/C	Ingst	Int-S	1		
		doxepin	1	1						
		bupropion (extended release)	2	2						
		olanzepine	3	3						
		ethanol	4	4						
		buspirone	5	5						
1154p	22 y M				A	Ingst	Int-S	2		
		bupropion	1	1						
		quetiapine	2	2						
		olanzapine	3	3						
		acetaminophen/hydrocodone	4	4						
		sertraline	5	5						
		clonazepam	6	6						
1155ha	23 y M				A/C	Ingst	Int-M	1		
		doxepin	1	1					nordoxepin	1600 ng/mL In Blood (unspecified) @ Autopsy
		doxepin	1	1					doxepin	540 ng/mL In Blood (unspecified) @ Autopsy
		valproic acid (extended release)	2	2					valproic acid	286 mcg/mL In Serum @ Unknown
		ethanol	3	3					ethanol	239 mg/dL In Serum @ Unknown
		cocaine	4	4						
		alprazolam	5	5						
1156	25 y F				A/C	Ingst	Int-S	1		
		amitriptyline	1	1						
1157	25 y F				A	Ingst	Int-S	1		
		bupropion	1	1						
		alprazolam	2	2						
1158pa	25 y F				U	Ingst	Int-S	2		
		citalopram	1	1					citalopram	4100 mcg/L In Blood (unspecified) @ Unknown
1159	25 y F				A	Ingst	Int-S	1		
		paroxetine	1	1						
		propranolol	2	2						
		salicylate	3	3						
		gabapentin	4	4						
1160p	25 y F				A/C	Ingst	Int-S	2		
		amitriptyline	1	1						
1161	26 y F				A	Ingst	Int-S	3		
		citalopram	1	1						
1162ai	26 y F				A	Ingst	Int-S	2		
		trazodone	1	1						
		sertraline	2	2						
		hydrocodone	3	3						
		alprazolam	4	4						
		acetaminophen	5	5						
1163ai	27 y F				U	Ingst	Int-A	2		
		sertraline	1	1						
		trazodone	2	2						
		diphenhydramine	3	3						
		dextromethorphan	4	4						
1164ha	28 y F				A/C	Ingst+ Unk	Unk	2		
		bupropion	1	1						
		ethanol	2	2						
		cocaine	3	3						
1165p	28 y F				A/C	Ingst	Int-S	2		
		bupropion	1	1						
		escitalopram	2	2						
		gabapentin	3	3						
		alprazolam	4	4						
1166ai	28 y F				U	Ingst	Int-A	2		
		doxepin	1	1						
		methadone	2	2						
		ethanol	3	3						
1167ha	29 y F				A/C	Ingst	Int-S	2		
		venlafaxine	1	1					norvenlafaxine	15.171 mg/L In Blood (unspecified) @ Autopsy
		venlafaxine	1	1					venlafaxine	26.959 mg/L In Blood (unspecified) @ Autopsy
		venlafaxine	1	1					venlafaxine	51.887 mg/L In Blood (unspecified) @ Autopsy
		venlafaxine	1	1					norvenlafaxine	7.119 mg/L In Blood (unspecified) @ Autopsy
		amphetamine/dextroamphetamine	2	2						
		phencyclidine	3	3						
1168ai	29 y M				U	Ingst	Int-S	2		
		doxepin	1	1						
		fluoxetine	2	2						
1169ai	30 y M				U	Ingst	Int-A	2		
		amitriptyline	1	1						
		ethanol	2	2						
1170h	31 y F				A/C	Ingst	Int-M	2		
		citalopram	1	1						
		antihistamine	2	2						
		gabapentin	3	3						
1171ai	31 y F				U	Ingst	Int-A	2		
		amitriptyline	1	1						
1172ai	32 y F				A	Ingst	Int-U	2		
		fluoxetine	1	1						
		propranolol	2	2						
		amitriptyline	3	3						
		dextromethorphan	4	4						
1173	32 y F				A	Ingst	Int-S	2		
		paroxetine	1	1						
		trazodone	2	2						
1174h	32 y M				C	Ingst	Unk	2		
		lithium	1	1					lithium	4.81 mmol/L In Blood (unspecified) @ Unknown
1175i	33 y M				A/C	Ingst	Int-S	3		
		citalopram	1	1					citalopram	2201 mg/mL In Blood (unspecified) @ Unknown
1176pha	33 y F				U	Ingst	Int-S	2		
		amitriptyline	1	1						
1177h	33 y M				A	Ingst	Int-S	2		
		amitriptyline	1	1						
		amitriptyline	2	2						
		amphetamine/dextroamphetamine	3	3						
		quetiapine	4	4						
		meloxicam	5	5						
		amoxicillin	6	6						
		acetaminophen/hydrocodone	7	7					acetaminophen	22 mcg/mL In Blood (unspecified) @ 3 h (pe)
1178ai	34 y F				A	Ingst	Int-S	2		
		bupropion	1	1						
		tizanidine	2	2						
		amitriptyline	3	3						
		oxycodone	4	4						
		alprazolam	5	5						
		acetaminophen	6	6						
1179pa	34 y M				A/C	Ingst	Int-S	1		
		bupropion (extended release)	1	1					hydroxybupropion	180 ng/mL In Blood (unspecified) @ Unknown
		venlafaxine	2	2					venlafaxine	3000 ng/mL In Blood (unspecified) @ Unknown
		ethanol	3	3					ethanol	146 mg/dL In Whole Blood @ Unknown
1180pha	34 y F				A/C	Ingst	Int-S	2		
		amitriptyline	1	1					nortriptyline	35 ng/mL In Blood (unspecified) @ Autopsy
		tramadol	2	2					o-demethyl tramadol	210 ng/mL In Blood (unspecified) @ Autopsy
		tramadol	2	2					tramadol	460 ng/mL In Blood (unspecified) @ Autopsy
		acetaminophen/hydrocodone	3	3						
		ondansetron	4	4						
		linaclotide	5	5						
		diclofenac	6	6						
		lubiprostone	7	7						
		zolpidem	8	8						
		liothyronine	9	9						
		escitalopram	10	10						
		sertraline	11	11						
		gabapentin	12	12					gabapentin	1.1 mcg/mL In Blood (unspecified) @ Autopsy
1181pa	34 y F				A/C	Ingst	Int-U	2		
		citalopram	1	1					citalopram	7300 ng/mL In Blood (unspecified) @ Unknown
		aripiprazole	2	2						
1182	35 y F				A/C	Ingst	Int-S	3		
		lithium	1	1					lithium	0.9 mEq/L In Blood (unspecified) @ 11 h (pe)
		lithium	1	1					lithium	0.9 mEq/L In Blood (unspecified) @ 5 h (pe)
		lithium	1	1					lithium	1.2 mEq/L In Blood (unspecified) @ 6.5 h (pe)
		lithium	1	1					lithium	2.3 mEq/L In Blood (unspecified) @ 3 h (pe)
		quetiapine	2	2						
[1183h]	35 y F				C	Ingst	Int-M	1		
		lithium	1	1					lithium	1.7 mEq/L In Blood (unspecified) @ 24 h (pe)
		lithium	1	1					lithium	4.4 mEq/L In Blood (unspecified) @ 1 h (pe)
1184	35 y M				A	Ingst	Int-S	2		
		bupropion	1	1						
		ethanol	2	2						
1185pa	36 y F				A/C	Ingst	Int-S	1		
		venlafaxine	1	1					venlafaxine	10 mg/L In Blood (unspecified) @ Autopsy
		venlafaxine	1	1					o-desmethylvenlafaxine	13 mg/kg In Liver @ Autopsy
		venlafaxine	1	1					venlafaxine	46 mg/kg In Liver @ Autopsy
		venlafaxine	1	1					o-desmethylvenlafaxine	5.3 mg/L In Blood (unspecified) @ Autopsy
		quetiapine	2	2					quetiapine	100 mg/kg In Liver @ Autopsy
		quetiapine	2	2					quetiapine	6.9 mg/L In Blood (unspecified) @ Autopsy
		amphetamine/dextroamphetamine (extended release)	3	3					amphetamine	0.21 mg/L In Blood (unspecified) @ Autopsy
		diazepam	4	4					oxazepam	0.078 mg/kg In Blood (unspecified) @ Autopsy
		diazepam	4	4					nordiazepam	0.38 mg/kg In Blood (unspecified) @ Autopsy
		diazepam	4	4					diazepam	0.46 mg/kg In Blood (unspecified) @ Autopsy
		tramadol	5	5					tramadol	0.31 mg/L In Blood (unspecified) @ Autopsy
		trazodone	6	6					meta-chlorophenylpiperazine (mcpp)	1.1 mg/L In Blood (unspecified) @ Autopsy
		trazodone	6	6					trazodone	15 mg/kg In Liver @ Autopsy
		trazodone	6	6					trazodone	5.2 mg/L In Blood (unspecified) @ Autopsy
		trazodone	6	6					meta-chlorophenylpiperazine (mcpp)	7.1 mg/kg In Liver @ Autopsy
1186p	37 y F				A/C	Ingst	Int-S	1		
		amitriptyline	1	1						
1187ai	37 y F				A	Ingst+ Inhal	Int-U	2		
		doxepin	1	1						
		cocaine	2	2						
		diphenhydramine	3	3						
		ethanol	4	4						
1188h	37 y F				A/C	Ingst	Int-S	2		
		trazodone	1	1					trazodone	814 ng/mL In Serum @ 33 h (pe)
1189pa	37 y F				A/C	Ingst	Int-S	2		
		venlafaxine	1	1						
		oxycodone	2	2						
		orphenadrine	3	3						
		gabapentin	4	4						
		cetirizine	5	5						
		omeprazole	6	6						
1190p	38 y F				A/C	Ingst	Int-S	2		
		bupropion (extended release)	1	1						
		venlafaxine	2	2						
		pramipexole	3	3						
		zolpidem (extended release)	4	4						
1191a	38 y F				A	Ingst	Int-S	1		
		amitriptyline	1	1						
		beta blocker	2	2						
1192h	38 y M				A/C	Ingst	Int-S	2		
		venlafaxine	1	1						
		ethanol	2	2						
1193p	40 y F				A/C	Ingst	Int-S	2		
		trazodone	1	1						
		drug, unknown	2	2						
1194	41 y F				A	Ingst	Unk	2		
		amitriptyline	1	1						
		quetiapine	2	2						
		benzonatate	3	3						
		duloxetine	4	4						
		gabapentin	5	5						
		tizanidine	6	6						
1195ai	42 y F				U	Ingst	Int-A	2		
		fluoxetine	1	1						
		dextromethorphan	2	2						
		zolpidem	3	3						
1196	42 y F				U	Ingst	Int-S	2		
		amitriptyline	1	1						
		buprenorphine	2	2						
1197	43 y F				A/C	Ingst	Unk	2		
		sertraline	1	1						
		escitalopram	2	2						
		atomoxetine	3	3						
1198ha	43 y M				A/C	Ingst	Int-S	1		
		amitriptyline	1	1					nortriptyline	1600 ng/mL In Blood (unspecified) @ Autopsy
		amitriptyline	1	1					amitriptyline	3400 ng/mL In Blood (unspecified) @ Autopsy
		cocaine	2	2						
		ethanol	3	3					ethanol	163 mg/dL In Blood (unspecified) @ Autopsy
		ethanol	3	3					ethanol	219 mg/dL In Blood (unspecified) @ 20 m (pe)
1199	43 y F				U	Ingst	Int-S	1		
		amitriptyline	1	1						
		clonidine	2	2						
[1200a]	43 y F				A/C	Ingst	Int-S	1		
		bupropion	1	1					bupropion	1.5 mg/L In Blood (unspecified) @ Unknown
		bupropion	1	1					bupropion	14 mg/kg In Liver @ Autopsy
		bupropion	1	1					threobupropion	150 mg/kg In Liver @ Autopsy
		bupropion	1	1					threobupropion	5.6 mg/L In Blood (unspecified) @ Unknown
		diltiazem (extended release)	2	2						
		prednisone	3	3						
1201h	44 y F				A/C	Ingst	Int-S	1		
		amitriptyline	1	1						
		asenapine	2	2						
		clonazepam	3	3						
1202ai	44 y F				U	Ingst+ Unk	Int-A	2		
		fluoxetine	1	1						
		morphine	2	2						
		fentanyl	3	3						
		diphenhydramine	4	4						
		diazepam	5	5						
1203pha	44 y F				U	Ingst	Int-S	1		
		amitriptyline	1	1					amitriptyline	0.68 mg/L In Blood (unspecified) @ Autopsy
		amitriptyline	1	1					nortriptyline	1.9 mg/L In Blood (unspecified) @ Autopsy
		amitriptyline	1	1					amitriptyline	25 mg/kg In Liver @ Autopsy
		amitriptyline	1	1					nortriptyline	86 mg/kg In Liver @ Autopsy
		quetiapine	2	2					quetiapine	1.4 mg/L In Blood (unspecified) @ Autopsy
		quetiapine	2	2					quetiapine	17 mg/kg In Liver @ Autopsy
		diphenhydramine	3	3					diphenhydramine	0.27 mg/L In Blood (unspecified) @ Autopsy
		alprazolam	4	4					alprazolam	0.01 mg/L In Blood (unspecified) @ Autopsy
		gabapentin	5	5					gabapentin	4 mg/L In Blood (unspecified) @ Autopsy
		cocaine	6	6					benzoylecognine	0.074 mg/L In Blood (unspecified) @ Autopsy
		clonidine	7	7						
1204ai	45 y M				U	Ingst	Int-A	2		
		amitriptyline	1	1						
		acetaminophen/hydrocodone	2	2						
		chlorpromazine	3	3						
		dextromethorphan	4	4						
		benztropine	5	5						
1205	45 y F				A/C	Ingst	Int-S	1		
		amitriptyline	1	1						
1206ai	46 y F				A	Ingst	Int-S	2		
		duloxetine	1	1						
		citalopram	2	2						
		tramadol	3	3						
		diphenhydramine	4	4						
1207ai	46 y F				A	Ingst	Int-S	2		
		nortriptyline	1	1						
		oxycodone	2	2						
		oxymorphone	3	3						
		sertraline	4	4						
		alprazolam	5	5						
		acetaminophen	6	6						
		ethanol	7	7						
1208ai	46 y F				U	Ingst	AR-D	2		
		fluoxetine	1	1						
		metoprolol	2	2						
1209	46 y M				A/C	Ingst	Int-S	1		
		venlafaxine	1	1						
1210	46 y F				A/C	Ingst	Int-S	3		
		amitriptyline	1	1						
		topiramate	2	2						
		desvenlafaxine	3	3						
		zolpidem	4	4						
1211h	46 y F				A/C	Ingst	Unk	2		
		cyclic antidepressant, unknown	1	1						
		quetiapine	2	2						
		risperidone	3	3					9-hydroxyrisperidone	196.8 ng/mL In Blood (unspecified) @ Autopsy
		risperidone	3	3					risperidone	253.7 ng/mL In Blood (unspecified) @ Autopsy
1212pa	47 y F				A/C	Ingst	Int-U	2		
		amitriptyline	1	1						
1213p	48 y M				A	Ingst	Int-S	1		
		amitriptyline	1	1						
		benzodiazepine	2	2						
1214ai	48 y F				U	Ingst	Int-S	2		
		bupropion	1	1						
		beta blocker	2	2						
		oxycodone	3	3						
		promethazine	4	4						
1215ph	48 y F				A/C	Ingst	Int-S	1		
		bupropion	1	1						
1216a	48 y F				A	Ingst	Int-S	2		
		bupropion (extended release)	1	1						
		ethanol	2	2						
1217h	48 y F				A	Ingst	Int-S	2		
		lithium	1	1					lithium	5.7 mEq/L In Serum @ Unknown
		bupropion	2	2						
		acetaminophen	3	3					acetaminophen	162 mcg/mL In Serum @ Unknown
		ethanol	4	4					ethanol	191 mg/dL In Serum @ Unknown
1218ha	49 y F				A	Ingst	Int-S	1		
		venlafaxine (extended release)	1	1					venlafaxine	20117 ng/mL In Blood (unspecified) @ Autopsy
		venlafaxine (extended release)	1	1					norvenlafaxine	3608 ng/mL In Blood (unspecified) @ Autopsy
		clonazepam	2	2					7-aminoclonazepam	207 ng/mL In Blood (unspecified) @ Autopsy
		clonazepam	2	2					clonazepam	354 ng/mL In Blood (unspecified) @ Autopsy
1219h	49 y F				A	Ingst	Int-S	2		
		amitriptyline	1	1						
		antidepressant (SSRI)	2	2						
		lorazepam	3	3						
1220ai	50 y M				U	Ingst	Int-A	2		
		amitriptyline	1	1						
1221ai	50 y F				A	Ingst	Int-S	2		
		amitriptyline	1	1						
1222	51 y M				A	Ingst	Int-S	2		
		doxepin	1	1						
1223pa	51 y F				A/C	Ingst	Unk	1		
		nortriptyline	1	1						
		methadone	2	2						
1224ai	52 y M				U	Ingst	Int-A	2		
		trazodone	1	1						
1225ai	52 y F				U	Ingst	Int-A	2		
		antidepressant	1	1						
1226ai	53 y M				A	Ingst+ Unk	Int-S	2		
		venlafaxine	1	1						
		citalopram	2	2						
		diphenhydramine	3	3						
		cocaine	4	4						
		quetiapine	5	5						
		ethanol	6	6						
1227ai	53 y F				U	Ingst+ Unk	Int-A	2		
		citalopram	1	1						
		diphenhydramine	2	2						
		fentanyl	3	3						
		alprazolam	4	4						
		midazolam	5	5						
1228	53 y M				U	Ingst	AR-D	1		
		venlafaxine	1	1					venlafaxine	6340 ng/mL In Blood (unspecified) @ Unknown
		carbamazepine	2	2						
		trazodone	3	3						
		hydroxyzine	4	4						
		angiotensin converting enzyme inhibitor	5	5						
1229ai	54 y M				A	Ingst	Int-S	2		
		sertraline	1	1						
		doxylamine	2	2						
		alprazolam	3	3						
		ethanol	4	4						
1230a	54 y M				U	Ingst	Int-S	1		
		doxepin	1	1					desmethyldoxepin	82 ng/mL In Blood (unspecified) @ Unknown
		doxepin	1	1					doxepin	870 ng/mL In Blood (unspecified) @ Unknown
1231	54 y M				A/C	Ingst	Int-U	2		
		lithium	1	1					lithium	2.8 mEq/L In Blood (unspecified) @ Unknown
1232p	54 y F				A/C	Ingst	Int-S	2		
		bupropion	1	1						
		amphetamine/dextroamphetamine	2	2						
		alprazolam	3	3						
		ethanol	4	4						
1233h	54 y M				C	Ingst	AR-D	3		
		lithium	1	1					lithium	2.3 mmol/L In Serum @ Unknown
		beta blocker	2	2						
1234	55 y M				A	Ingst	Int-S	2		
		doxepin	1	1					nordoxepin	0.34 mg/L In Blood (unspecified) @ Autopsy
		doxepin	1	1					doxepin	5.7 mg/L In Blood (unspecified) @ Autopsy
		ethanol	2	2					ethanol	0.08 g/dL In Blood (unspecified) @ Autopsy
		ethanol	2	2					ethanol	0.11 g/dL In Vitreous @ Autopsy
		lamotrigine	3	3						
1235ai	55 y F				A	Ingst	Int-S	2		
		fluoxetine	1	1						
		dextromethorphan	2	2						
		doxylamine	3	3						
		metoprolol	4	4						
1236ai	55 y M				U	Ingst	Unk	2		
		doxepin	1	1						
1237p	55 y F				A/C	Ingst	Int-S	3		
		trazodone	1	1						
		ethanol	2	2						
1238h	55 y F				A	Ingst+ Inhal	Int-S	2		
		amitriptyline	1	1						
		carbon monoxide	2	2					carboxyhemoglobin	2.9 mg/dL In Blood (unspecified) @ 1 h (pe)
1239ai	56 y M				U	Ingst	Int-A	2		
		antidepressant	1	1						
1240ph	57 y F				A	Ingst	Int-S	2		
		venlafaxine	1	1						
		ethanol	2	2					ethanol	0.01 % In Blood (unspecified) @ Unknown
		benzodiazepine	3	3					nordiazepam	0.185 mg/L In Blood (unspecified) @ Unknown
		benzodiazepine	3	3					diazepam	0.907 mg/L In Blood (unspecified) @ Unknown
		metronidazole	4	4						
		diphenhydramine	5	5					diphenhydramine	4.939 mg/L In Blood (unspecified) @ Unknown
1241	57 y F				U	Ingst+ Unk	Int-S	2		
		cyclic antidepressant, unknown	1	1						
		opioid	2	2						
		benzodiazepine	3	3						
		cocaine	4	4						
1242	57 y F				A/C	Ingst	Int-S	1		
		desipramine	1	1						
		clonazepam	2	2						
1243a	57 y F				A	Ingst	Int-S	2		
		nortriptyline	1	1					nortriptyline	0.484 mg/L In Blood (unspecified) @ Autopsy
1244	58 y M				A	Ingst	Int-S	2		
		amitriptyline	1	1						
1245ai	59 y M				U	Ingst	Int-A	2		
		paroxetine	1	1						
		diazepam	2	2						
1246ai	59 y F				U	Ingst	Int-A	2		
		fluoxetine	1	1						
		ethanol	2	2						
		quetiapine	3	3						
1247	59 y M				A	Ingst	Int-S	2		
		nortriptyline	1	1						
1248ai	61 y M				U	Ingst+ Unk	Unk	2		
		citalopram	1	1						
		morphine	2	2						
		acetaminophen/hydrocodone	3	3						
		zolpidem	4	4						
		diazepam	5	5						
		mirtazapine	6	6						
1249	61 y F				A	Ingst	Int-S	2		
		cyclic antidepressant, unknown	1	1						
		benzodiazepine	2	2						
		opioid	3	3						
		methadone	4	4						
1250ai	62 y M				A	Ingst	Int-U	2		
		paroxetine	1	1						
		theophylline	2	2						
		acetaminophen	3	3						
1251	63 y M				A	Ingst	Int-S	3		
		amitriptyline	1	1						
		clonazepam	2	2						
		acetaminophen/hydrocodone	3	3					acetaminophen	11.5 mcg/mL In Blood (unspecified) @ Unknown
1252ai	64 y M				A	Ingst	Int-S	2		
		bupropion	1	1						
		phentermine	2	2						
		pseudoephedrine	3	3						
		sertraline	4	4						
		hydrocodone	5	5						
		dextromethorphan	6	6						
		fluoxetine	7	7						
		doxylamine	8	8						
		acetaminophen	9	9						
1253hi	64 y M				A/C	Ingst	Int-S	1		
		lithium	1	1					lithium	1.21 mEq/L In Serum @ 2 d (pe)
		lithium	1	1					lithium	2.33 mEq/L In Serum @ 24 h (pe)
		lithium	1	1					lithium	2.5 mEq/L In Serum @ 5 h (pe)
		lithium	1	1					lithium	3.53 mEq/L In Serum @ 36 h (pe)
		lithium	1	1					lithium	5.3 mEq/L In Serum @ 18 h (pe)
1254p	64 y F				A/C	Ingst	Int-S	2		
		bupropion	1	1						
1255	66 y M				A/C	Ingst	Int-S	3		
		citalopram *	1	1					citalopram	0.05 mg/L In Blood (unspecified) @ Unknown
		quetiapine *	2	1					quetiapine	0.44 mg/L In Blood (unspecified) @ Autopsy
		quetiapine *	2	1					quetiapine	3.5 mg/L In Blood (unspecified) @ Unknown
1256ha	66 y F				A/C	Ingst	Int-S	1		
		nortriptyline	1	1					nortriptyline	370 ng/mL In Blood (unspecified) @ Autopsy
		oxycodone	2	2					oxycodone (free)	870 ng/mL In Blood (unspecified) @ Autopsy
		alprazolam	3	3					alprazolam	0.24 mg/L In Blood (unspecified) @ Autopsy
		duloxetine	4	4						
		carbidopa/levodopa	5	5						
1257	67 y F				A/C	Ingst	Int-S	1		
		amitriptyline	1	1						
		oxycodone	2	2						
1258p	67 y F				U	Ingst	Int-S	1		
		amitriptyline	1	1						
1259	67 y M				A/C	Ingst	Int-S	2		
		paroxetine	1	1						
1260	67 y M				U	Ingst	Int-S	2		
		trazodone	1	1						
		sertraline	2	2						
		drug, unknown	3	3						
		drug, unknown	4	4						
1261	68 y F				A/C	Ingst	Int-S	2		
		venlafaxine	1	1						
		clonazepam	2	2						
1262h	68 y M				A/C	Ingst+ Par	Int-S	2		
		trazodone	1	1						
		acetaminophen/hydrocodone	2	2						
		acetaminophen	3	3						
		alprazolam	4	4						
		insulin	5	5						
1263ai	69 y F				U	Ingst	Int-A	2		
		amitriptyline	1	1						
		codeine	2	2						
		citalopram	3	3						
		meclizine	4	4						
		diphenhydramine	5	5						
1264ph	77 y M				A/C	Ingst	Int-S	3		
		amitriptyline	1	1						
		temazepam	2	2						
		hydrochlorothiazide	3	3						
		hydrocodone	4	4						
1265ai	78 y M				A	Ingst	Int-S	2		
		mirtazapine	1	1						
		flurazepam	2	2						
		paroxetine	3	3						
1266	82 y M				A	Ingst	Unk	2		
		desvenlafaxine	1	1						
		oxycodone	2	2						
1267	88 y F				A/C	Ingst	Int-S	1		
		venlafaxine	1	1						
		metoprolol	2	2						
		gabapentin	3	3						
		buspirone	4	4						
		levothyroxine	5	5						
[1268pha]	9 m M				A	Ingst	Oth-M	1		
		amitriptyline	1	1					nortriptyline	1.7 mg/L In Blood (unspecified) @ Autopsy
		amitriptyline	1	1					nortriptyline	28 mg/kg In Liver @ Autopsy
		amitriptyline	1	1					amitriptyline	3.5 mg/L In Blood (unspecified) @ Autopsy
		amitriptyline	1	1					amitriptyline	46 mg/kg In Liver @ Autopsy
		diphenhydramine	2	2					diphenhydramine	1.9 mg/L In Blood (unspecified) @ Autopsy
		diphenhydramine	2	2					diphenhydramine	8.3 mg/kg In Liver @ Autopsy
1269pa	40 + y M				A/C	Ingst	Int-S	2		
		amitriptyline	1	1						
		alprazolam	2	2					alprazolam	1149 ng/mL In Urine (quantitative only) @ Autopsy
		alprazolam	2	2					alprazolam	50.6 ng/mL In Blood (unspecified) @ Autopsy
		alprazolam	2	2					alpha-oh-alprazolam	947 ng/mL In Urine (quantitative only) @ Autopsy
		acetaminophen/hydrocodone	3	3					hydrocodone	1000 ng/mL In Urine (quantitative only) @ Autopsy
		acetaminophen/hydrocodone	3	3					hydrocodone	168 ng/mL In Blood (unspecified) @ Autopsy
		acetaminophen/hydrocodone	3	3					hydromorphone	291 ng/mL In Urine (quantitative only) @ Autopsy
1270ai	Unknown adult (> = 20 yrs) F				U	Ingst	Unt-T	2		
		lithium	1	1						
		venlafaxine	2	2						
		hydrocodone	3	3						
		quetiapine	4	4						
		trazodone	5	5						
		gabapentin	6	6						
		clonazepam	7	7						
		topiramate	8	8						
1271	Unknown age F				A	Ingst	Int-S	1		
		citalopram	1	1						
		trazodone	2	2						
		acyclovir	3	3						
		naproxen	4	4						
See Also case 14, 18, 22, 33, 48, 67, 81, 84, 105, 111, 112, 114, 128, 142, 163, 187, 191, 228, 244, 253, 267, 280, 282, 290, 301, 306, 311, 312, 357, 358, 362, 375, 400, 416, 437, 441, 445, 452, 458, 475, 486, 490, 494, 499, 502, 511, 516, 518, 525, 528, 549, 552, 555, 557, 561, 575, 589, 590, 597, 602, 612, 618, 620, 625, 635, 637, 639, 644, 647, 649, 661, 669, 670, 674, 675, 678, 682, 685, 687, 692, 699, 706, 711, 727, 728, 747, 749, 755, 759, 765, 766, 767, 770, 771, 774, 781, 785, 787, 789, 791, 800, 801, 803, 811, 812, 826, 830, 832, 846, 849, 854, 865, 871, 876, 879, 884, 892, 895, 898, 899, 903, 908, 912, 916, 919, 920, 928, 930, 932, 933, 944, 946, 948, 955, 959, 960, 972, 979, 991, 992, 993, 1000, 1006, 1019, 1026, 1040, 1044, 1047, 1071, 1073, 1104, 1116, 1117, 1118, 1120, 1121, 1122, 1125, 1126, 1127, 1129, 1131, 1133, 1134, 1139, 1277, 1279, 1294, 1295, 1297, 1298, 1303, 1326, 1330, 1340, 1346, 1351, 1353, 1354, 1355, 1358, 1361, 1363, 1366, 1372, 1382, 1385, 1387, 1395, 1397, 1399, 1403, 1404, 1406, 1408, 1416, 1420, 1421, 1424, 1430, 1436, 1437, 1439, 1444, 1466, 1470, 1483, 1494, 1504, 1506, 1510, 1512, 1519, 1527, 1528, 1531, 1568, 1570, 1573, 1576, 1577, 1582, 1586, 1589, 1599, 1600, 1605, 1606, 1607, 1609, 1613, 1621, 1628, 1630, 1631, 1635, 1646, 1650, 1651, 1660, 1661, 1677, 1685, 1697, 1709, 1739, 1742, 1743, 1765, 1769, 1770, 1788, 1792, 1799, 1801, 1808, 1810, 1814, 1818, 1832, 1833, 1835, 1843, 1846, 1847, 1850, 1859, 1867, 1872, 1874, 1875, 1876, 1879, 1883, 1892, 1895, 1899, 1900, 1906, 1917, 1918, 1920, 1925, 1930, 1933, 1942, 1945, 1966, 1967, 1970, 1972, 1975, 1979, 1984, 1991, 1993, 1998, 2007, 2010, 2011, 2015, 2035, 2044, 2049, 2050, 2051, 2059, 2060, 2070, 2110										
**Antihistamines**										
[1272h]	2 y F				A	Ingst	Unt-G	2		
		diphenhydramine	1	1						
1273pha	14 y F				U	Ingst	Int-S	2		
		diphenhydramine	1	1						
		metformin	2	2						
		loratadine	3	3						
		lovastatin	4	4						
1274	18 y M				U	Ingst	Int-S	1		
		diphenhydramine	1	1						
		quetiapine	2	2						
1275h	20 y F				A	Ingst	Int-S	1		
		diphenhydramine	1	1						
		ibuprofen	2	2						
1276ph	21 y M				U	Ingst	Int-S	2		
		diphenhydramine	1	1						
1277a	21 y F				A	Ingst	Int-S	1		
		diphenhydramine	1	1					diphenhydramine	0.5 mg/L In Blood (unspecified) @ Autopsy
		salicylate	2	2					salicylate	10.9 mg/L In Serum @ 30 m (pe)
		cyclobenzaprine	3	3					cyclobenzaprine	0.06 mg/L In Blood (unspecified) @ Autopsy
		citalopram	4	4					citalopram	0.4 mg/L In Blood (unspecified) @ Autopsy
		ibuprofen	5	5						
1278a	23 y M				A	Par	Int-S	1		
		diphenhydramine	1	1					diphenhydramine	316 ng/mL In Blood (unspecified) @ Unknown
		diphenhydramine	1	1					diphenhydramine	372 ng/mL In Blood (unspecified) @ Autopsy
		hydromorphone	2	2					morphine	15 ng/mL In Blood (unspecified) @ Autopsy
		hydromorphone	2	2					hydromorphone	3 ng/mL In Blood (unspecified) @ Autopsy
		hydromorphone	2	2					hydromorphone	5.5 ng/mL In Serum @ Autopsy
		hydromorphone	2	2					morphine	74.4 ng/mL In Blood (unspecified) @ Autopsy
		fentanyl *	3	3					fentanyl	14.4 ng/mL In Blood (unspecified) @ Autopsy
		fluconazole *	4	3						
1279ha	30 y F				A/C	Ingst	Int-S	1		
		diphenhydramine	1	1					diphenhydramine	4919 ng/mL In Serum @ Unknown
		cyclic antidepressant, unknown	2	2					duloxetine	278 ng/mL In Serum @ Unknown
		clonazepam	3	3					7-aminoclonazepam	60.9 ng/mL In Serum @ Unknown
		clonazepam	3	3					7-aminoclonazepam	796 ng/mL In Urine (quantitative only) @ 2 d (pe)
		clonazepam	3	3					clonazepam	83.1 ng/mL In Serum @ Unknown
		anticonvulsant	4	4					gabapentin	19.7 mcg/mL In Serum @ Unknown
		antidepressant (SSRI)	5	5					sertraline	311 ng/mL In Serum @ Unknown
		ziprasidone	6	6						
1280ai	31 y F				A	Ingst	Int-S	2		
		diphenhydramine	1	1						
1281ai	33 y F				U	Ingst	Int-S	2		
		diphenhydramine	1	1						
		cyclobenzaprine	2	2						
		phentermine	3	3						
		codeine	4	4						
		acetaminophen/hydrocodone	5	5						
		butalbital	6	6						
1282	34 y F				A	Ingst	Unk	2		
		diphenhydramine	1	1					diphenhydramine	1.1 mg/L In Whole Blood @ Autopsy
		morphine	2	2					morphine	0.18 mg/L In Whole Blood @ Autopsy
		zolpidem	3	3					zolpidem	0.12 mg/L In Whole Blood @ Autopsy
		acetaminophen/hydrocodone	4	4						
		alprazolam	5	5						
1283h	35 y F				C	Ingst	Int-A	2		
		diphenhydramine	1	1						
		N-acetylcsysteine	2	2						
1284p	36 y F				A/C	Par	Int-A	2		
		diphenhydramine	1	1						
1285pha	37 y F				A	Ingst	Int-S	1		
		diphenhydramine	1	1					diphenhydramine	15490 ng/mL In Blood (unspecified) @ Autopsy
1286ph	38 y F				A	Ingst	Int-S	1		
		diphenhydramine	1	1						
		ethanol	2	2					ethanol	204 mg/dL In Serum @ 10 m (pe)
1287ai	42 y M				U	Ingst	Int-A	2		
		diphenhydramine	1	1						
[1288pha]	43 y F				A	Ingst	Int-S	1		
		diphenhydramine	1	1					diphenhydramine	28 mcg/mL In Whole Blood @ Autopsy
1289ai	45 y M				U	Ingst	Int-A	2		
		diphenhydramine	1	1						
1290pa	45 y M				A	Unk	Int-A	1		
		diphenhydramine	1	1					diphenhydramine	0.4 mg/L In Blood (unspecified) @ Autopsy
		hyperthermia	2	2						
		drug, unknown	3	3						
1291ai	45 y M				U	Ingst	Int-A	2		
		promethazine	1	1						
		acetaminophen/hydrocodone	2	2						
		ethanol	3	3						
		diphenhydramine	4	4						
		zolpidem	5	5						
1292ai	48 y F				U	Ingst	Int-A	2		
		diphenhydramine	1	1						
		tramadol	2	2						
1293ai	49 y M				U	Ingst	Int-A	2		
		diphenhydramine	1	1						
		ethanol	2	2						
1294p	50 y F				A	Ingst	Int-S	1		
		diphenhydramine	1	1						
		bupropion	2	2						
		lamotrigine	3	3						
		lisdexamfetamine	4	4						
		naproxen	5	5						
		alprazolam	6	6						
1295	51 y F				A	Ingst	Int-S	3		
		promethazine	1	1						
		verapamil	2	2						
		escitalopram	3	3						
		acetaminophen/hydrocodone	4	4					acetaminophen	84 mcg/mL In Blood (unspecified) @ 12 h (pe)
		diazepam	5	5						
		topiramate	6	6						
		esomeprazole	7	7						
		diclofenac	8	8						
1296ai	51 y F				A	Ingst	Int-S	2		
		diphenhydramine	1	1						
		ethanol	2	2						
1297ai	53 y M				A	Ingst	Int-S	2		
		diphenhydramine	1	1						
		dextromethorphan	2	2						
		bupropion	3	3						
		ethanol	4	4						
1298a	66 y M				A	Ingst	Int-S	1		
		diphenhydramine	1	1						
		risperidone	2	2						
		salicylate	3	3						
		mirtazapine	4	4						
		valproic acid	5	5						
See Also case 8, 22, 33, 38, 43, 67, 81, 87, 95, 116, 120, 163, 232, 251, 257, 268, 270, 280, 282, 285, 286, 287, 347, 357, 358, 362, 380, 402, 403, 412, 418, 423, 434, 437, 440, 445, 446, 470, 473, 480, 484, 497, 509, 520, 538, 555, 565, 568, 571, 576, 587, 589, 596, 612, 625, 628, 631, 635, 638, 647, 650, 654, 655, 663, 675, 679, 682, 687, 690, 699, 702, 706, 715, 759, 767, 784, 789, 803, 804, 806, 826, 827, 845, 846, 864, 867, 877, 879, 887, 888, 890, 892, 905, 910, 923, 930, 946, 954, 965, 974, 978, 986, 990, 1019, 1026, 1031, 1038, 1092, 1147, 1163, 1187, 1189, 1202, 1203, 1206, 1214, 1226, 1227, 1228, 1240, 1263, 1268, 1336, 1351, 1358, 1382, 1395, 1489, 1490, 1494, 1495, 1504, 1528, 1531, 1551, 1562, 1565, 1573, 1580, 1596, 1609, 1656, 1665, 1685, 1697, 1709, 1716, 1721, 1722, 1727, 1734, 1742, 1751, 1752, 1779, 1788, 1793, 1797, 1801, 1805, 1806, 1807, 1818, 1826, 1830, 1832, 1850, 1865, 1875, 1877, 1878, 1881, 1894, 1903, 1906, 1920, 1933, 1943, 1945, 1946, 1960, 1967, 1972, 1979, 1985, 1990, 1992, 2006, 2019, 2021, 2041, 2049, 2052, 2066, 2074										
**Antimicrobials**										
1299	47 y M				A	Unk	Int-A	2		
		levamisole	1	1						
		cocaine	2	2						
1300	54 y M				A/C	Ingst	Int-A	1		
		levofloxacin	1	1						
		acetaminophen/oxycodone	2	2					acetaminophen	0 mcg/mL In Blood (unspecified) @ Unknown
		ethanol	3	3						
[1301pha]	65 y F				A/C	Ingst	Int-U	2		
		amantadine	1	1						
1302ai	72 y F				U	Ingst	Int-S	2		
		amantadine	1	1						
1303pha	74 y F				A	Ingst	Int-S	1		
		hydroxychloroquine	1	1						
		bupropion	2	2					hydroxybupropion	1500 ng/mL In Blood (unspecified) @ Autopsy
		bupropion	2	2					bupropion	860 ng/mL In Blood (unspecified) @ Autopsy
		zolpidem	3	3					zolpidem	210 ng/mL In Blood (unspecified) @ Autopsy
See Also case 294, 452, 765, 795, 808, 1031, 1112, 1129, 1177, 1240, 1271, 1278, 1594, 1600, 1641, 1690, 1709, 1710, 1743, 1744, 1751, 1752, 1756, 1765, 1779, 1783, 1784, 1788, 1793, 1797, 1801, 1811, 1814, 1818, 1832, 1841, 1850, 1855, 1861, 1866, 1874, 1875, 1879, 1880, 1881, 1885, 1888, 1912, 1920, 1930, 1943, 1957, 1965, 1971, 1973, 1975, 1979, 1981, 1991, 2002, 2007, 2008, 2015, 2018, 2019, 2022, 2039, 2044, 2047, 2048, 2052, 2053, 2065, 2066, 2068, 2070, 2077, 2096										
**Antineoplastics**										
1304h	1 y M				A	Par	Unt-T	1		
		antineoplastic drug	1	1						
1305h	62 y F				C	Unk	Unk	2		
		methotrexate	1	1					methotrexate	0.76 Other (see abst) In Serum @ Unknown
1306	79 y F				C	Ingst	AR-D	3		
		methotrexate	1	1					methotrexate	0.09 mmol/L In Blood (unspecified) @ Unknown
[1307h]	82 y F				A/C	Ingst	Unt-T	2		
		methotrexate	1	1					methotrexate	0.03 mmol/L In Blood (unspecified) @ 4 d (pe)
										
**Asthma Therapies**										
1308a	34 y F				U	Unk	Unk	3		
		theophylline	1	1						
		pseudoephedrine	2	2					ephedrine	5000 ng/mL In Blood (unspecified) @ Autopsy
		pseudoephedrine	2	2					pseudoephedrine	6600 ng/mL In Blood (unspecified) @ Autopsy
		phenylpropanolamine	3	3						
		ethanol	4	4					ethanol	30 mg/dL In Blood (unspecified) @ Autopsy
1309p	36 y M				A	Par	Int-A	1		
		epinephrine	1	1						
1310	61 y F				A/C	Ingst	AR-D	3		
		theophylline	1	1					theophylline	34.6 mg/L In Blood (unspecified) @ Unknown
See Also case 1250, 1403										
**Cardiovascular Drugs**										
1311h	16 y F				A	Ingst	Int-S	1		
		nebivolol	1	1						
		amlodipine	2	2						
		metformin	3	3						
1312p	17 y F				A/C	Ingst	Int-S	1		
		flecainide	1	1						
1313pa	17 y M				A	Ingst	Int-S	1		
		metoprolol	1	1					metoprolol	11.4 mg/L In Blood (unspecified) @ 5 m (pe)
1314ai	19 y F				A	Ingst	Int-S	2		
		verapamil	1	1						
		zolpidem	2	2						
1315a	20 y F				A	Ingst	Int-S	2		
		diltiazem (extended release)	1	1					diltiazem	16.7 mg/L In Blood (unspecified) @ Autopsy
1316a	20 y F				A	Ingst	Int-S	1		
		flecainide	1	1						
		ethanol	2	2					ethanol	158 mg/dL In Whole Blood @ 4 h (pe)
1317a	21 y F				A	Ingst	Int-S	2		
		carvedilol	1	1						
		methamphetamine	2	2						
		buspirone	3	3						
		zolpidem	4	4						
[1318h]	23 y F				C	Par	Unt-T	3		
		nitroprusside	1	1					cyanide	0.128 mg/L In Blood (unspecified) @ 3 d (pe)
		nitroprusside	1	1					cyanide	6.289 mg/L In Blood (unspecified) @ 3 d (pe)
1319ha	23 y F				A	Ingst	Int-S	1		
		flecanide	1	1					flecainide	25 mcg/mL In Blood (unspecified) @ Autopsy
		ethanol	2	2					ethanol	0.14 g/dL In Blood (unspecified) @ Autopsy
1320a	24 y F				A	Ingst	Int-S	1		
		diltiazem	1	1					diltiazem	38000 ng/mL In Blood (unspecified) @ Autopsy
1321p	24 y F				A/C	Ingst	AR-D	2		
		verapamil	1	1						
1322h	24 y F				A/C	Ingst	Int-S	1		
		verapamil	1	1						
		verapamil	2	2						
1323	25 y F				A	Ingst+ Par	Int-S	2		
		amlodipine	1	1						
		heroin	2	2						
		ethanol	3	3					ethanol	180 mg/dL In Blood (unspecified) @ Unknown
		acetaminophen	4	4					acetaminophen	90 mcg/mL In Blood (unspecified) @ Unknown
1324	25 y M				A	Ingst	Int-S	2		
		verapamil	1	1						
1325p	26 y M				U	Ingst	Int-U	2		
		propranolol	1	1						
		acetaminophen	2	2					acetaminophen	12 mcg/mL In Serum @ Unknown
1326	26 y M				A/C	Ingst	Int-S	1		
		beta blocker *	2	1						
		bupropion (extended release) *	1	1						
		benzodiazepine	3	3						
		zolpidem	4	4						
1327	26 y F				A	Ingst+ Par	Int-S	1		
		carvedilol	1	1						
		nebivolol	2	2						
		insulin	3	3						
		guanfacine	4	4						
		salicylate	5	5						
1328i	27 y F				A/C	Ingst	Int-S	1		
		verapamil	1	1						
1329a	27 y F				A	Ingst	Int-M	3		
		clonidine	1	1						
1330h	28 y F				A	Ingst	Int-S	1		
		verapamil	1	1						
		trazodone	2	2						
1331	29 y M				A	Ingst	Int-S	1		
		verapamil	1	1						
		ethanol	2	2						
1332h	29 y F				A	Ingst	Int-S	1		
		amlodipine	1	1						
		metoprolol	2	2						
1333h	30 y M				A	Ingst	Int-S	2		
		propafenone	1	1						
		metoprolol (extended release)	2	2						
1334h	31 y F				A	Ingst	Unk	2		
		verapamil	1	1						
		acetaminophen/hydrocodone	2	2					acetaminophen	25 mcg/mL In Blood (unspecified) @ Unknown
1335pa	33 y F				A	Ingst	Int-S	1		
		propranolol	1	1					propranolol	6600 ng/mL In Blood (unspecified) @ 1 h (pe)
		ethanol	2	2					ethanol	198 mg/dL In Blood (unspecified) @ 1 h (pe)
1336ha	34 y F				U	Ingst	Int-S	1		
		cardiac glycoside	1	1					digoxin	24 ng/mL In Blood (unspecified) @ Autopsy
		diphenhydramine	2	2					diphenhydramine	6.7 mg/L In Blood (unspecified) @ Autopsy
		zolpidem	3	3						
		drug, unknown	4	4						
1337a	34 y M				A/C	Ingst	Unt-T	3		
		verapamil	1	1						
1338a	36 y F				A	Ingst	Int-S	1		
		amlodipine	1	1					amlodipine	780 ng/mL In Blood (unspecified) @ Autopsy
1339ha	36 y F				A/C	Ingst	Int-M	1		
		diltiazem (extended release)	1	1						
1340ha	36 y F				A/C	Ingst	Int-S	1		
		verapamil	1	1					verapamil	10.5 mg/L In Blood (unspecified) @ Autopsy
		escitalopram	2	2					citalopram	0.12 mg/L In Blood (unspecified) @ Autopsy
		codeine	3	3					morphine	0.055 mg/L In Blood (unspecified) @ Autopsy
		codeine	3	3					codeine	0.591 mg/L In Blood (unspecified) @ Autopsy
		topiramate	4	4					topiramate	9.84 mg/L In Blood (unspecified) @ Autopsy
1341a	37 y F				A/C	Ingst	Int-S	2		
		diltiazem	1	1						
		ethanol	2	2					ethanol	150 mg/dL In Blood (unspecified) @ Unknown
1342ph	37 y F				A/C	Ingst	Int-S	2		
		propranolol	1	1						
		pregabalin	2	2						
		amphetamine/dextroamphetamine (extended release)	3	3						
		gabapentin	4	4						
		clonazepam	5	5						
1343	37 y F				A	Ingst	Int-S	1		
		verapamil	1	1						
		quetiapine	2	2						
1344p	38 y M				A	Ingst	Unk	2		
		amlodipine/atorvastatin	1	1						
1345pha	38 y F				A/C	Ingst	Int-S	1		
		metoprolol	1	1					metoprolol	16000 ng/mL In Blood (unspecified) @ Autopsy
		nebivolol	2	2						
		hydromorphone	3	3					hydromorphone	130 ng/mL In Blood (unspecified) @ Autopsy
		oxymorphone	4	4					oxymorphone	130 ng/mL In Blood (unspecified) @ Autopsy
		warfarin	5	5						
1346ha	38 y M				A/C	Ingst	Int-S	1		
		flecainide	1	1					flecainide	15.23 mcg/mL In Blood (unspecified) @ Autopsy
		metformin	2	2						
		celecoxib	3	3						
		bupropion	4	4					bupropion	105 ng/mL In Blood (unspecified) @ Autopsy
		duloxetine	5	5					duloxetine	147 ng/mL In Blood (unspecified) @ Autopsy
		gabapentin	6	6					gabapentin	14.4 mcg/mL In Blood (unspecified) @ Autopsy
		warfarin	7	7						
1347	39 y M				U	Ingst	Unk	2		
		amlodipine	1	1						
		ethanol	2	2						
1348ha	40 y M				A	Ingst	Int-S	1		
		propranolol	1	1					propranolol	160 mg/kg In Liver @ Autopsy
		propranolol	1	1					propranolol	5.7 mg/L In Blood (unspecified) @ Autopsy
		gabapentin	2	2					gabapentin	10 mg/L In Blood (unspecified) @ Autopsy
1349	41 y F				U	Ingst	Unk	2		
		propranolol	1	1						
		drug, unknown	2	2						
		ethanol	3	3					ethanol	211 mg/dL In Serum @ Unknown
1350h	42 y M				A/C	Ingst	Int-S	2		
		carvedilol	1	1						
		nifedipine	2	2						
		alprazolam	3	3						
		acetaminophen/hydrocodone	4	4						
1351pai	42 y M				U	Unk	Unk	1		
		propranolol	1	1						
		methadone	2	2					methadone	169 ng/mL In Urine (quantitative only) @ Autopsy
		methadone	2	2					methadone	399 ng/mL In Blood (unspecified) @ Autopsy
		methadone	2	2					eddp (2-ethylidene-1,5-dimethyl-3,3-diphenyl pyrrolidine)	43.7 ng/mL In Blood (unspecified) @ Autopsy
		buprenorphine/naloxone (sublingual)	3	3					buprenorphine	0 ng/mL In Blood (unspecified) @ Autopsy
		ethanol	4	4						
		lithium	5	5						
		alprazolam	6	6					alpha-oh-alprazolam	1392 ng/mL In Urine (quantitative only) @ Autopsy
		alprazolam	6	6					alprazolam	152 ng/mL In Blood (unspecified) @ Autopsy
		alprazolam	6	6					alprazolam	891 ng/mL In Urine (quantitative only) @ Autopsy
		clonazepam	7	7					7-aminoclonazepam	32.4 ng/mL In Blood (unspecified) @ Autopsy
		diphenhydramine	8	8						
		naproxen	9	9						
		ibuprofen	10	10						
		pravastatin	11	11						
		lactobacillus acidophilus	12	12						
		marijuana	13	13					carboxy-thc	169 ng/mL In Urine (quantitative only) @ Autopsy
		marijuana	13	13					thc (tetrahydrocannabinol)	3.4 ng/mL In Blood (unspecified) @ Autopsy
		marijuana	13	13					delta-9-carboxy-thc	40.8 ng/mL In Blood (unspecified) @ Autopsy
1352	42 y M				A/C	Ingst	Int-S	1		
		carvedilol	1	1						
		diltiazem	2	2						
		phenytoin	3	3						
		pravastatin	4	4						
1353h	43 y M				A/C	Ingst	Int-S	1		
		amlodipine	1	1						
		carvedilol	2	2						
		lisinopril	3	3						
		paroxetine	4	4						
		simvastatin	5	5						
1354	43 y F				C	Ingst	Int-S	1		
		carvedilol	1	1						
		amlodipine	2	2						
		pramipexole	3	3						
		citalopram	4	4						
		risperidone	5	5						
		pravastatin	6	6						
		topiramate	7	7						
		omeprazole	8	8						
		levothyroxine	9	9						
1355ha	44 y M				A/C	Ingst	Int-S	2		
		metoprolol	1	1						
		metformin	2	2						
		acetaminophen/hydrocodone	3	3					acetaminophen	15 mcg/mL In Other @ Unknown
		acetaminophen/oxycodone	4	4					acetaminophen	15 mcg/mL In Unknown @ Unknown
		acetaminophen/oxycodone	4	4					oxycodone	30 ng/mL In Blood (unspecified) @ Unknown
		acetaminophen/oxycodone	4	4					oxycodone	62 ng/mL In Blood (unspecified) @ Autopsy
		thiazolidinedione	5	5						
		diazepam	6	6						
		zolpidem	7	7					zolpidem	70 ng/mL In Blood (unspecified) @ Unknown
		furosemide	8	8						
		citalopram	9	9					citalopram	216 ng/mL In Blood (unspecified) @ Autopsy
		citalopram	9	9					citalopram	74 ng/mL In Blood (unspecified) @ Unknown
1356	44 y F				A	Unk	Unk	2		
		metoprolol (extended release)	1	1						
1357a	44 y M				A/C	Ingst	Int-S	1		
		amlodipine	1	1					amlodipine	0.62 mg/L In Blood (unspecified) @ Unknown
		acetaminophen	2	2						
1358a	44 y F				A	Ingst	Int-S	1		
		diltiazem	1	1						
		citalopram	2	2						
		cetirizine	3	3						
1359	45 y M				A/C	Ingst	Int-S	3		
		beta blocker	1	1						
		isopropanol	2	2						
1360	46 y F				A/C	Ingst	Int-S	1		
		verapamil	1	1						
1361	46 y M				A/C	Ingst	Int-S	1		
		verapamil	1	1						
		lisinopril	2	2						
		metformin	3	3						
		simvastatin	4	4						
		fluoxetine	5	5						
1362a	46 y M				C	Ingst	AR-D	3		
		cardiac glycoside	1	1					digoxin	4.5 mg/mL In Plasma @ Unknown
1363a	47 y F				A/C	Ingst	Int-S	2		
		verapamil	1	1					verapamil	0.74 mcg/mL In Blood (unspecified) @ Unknown
		venlafaxine	2	2						
		amitriptyline	3	3						
		chlorpromazine	4	4						
1364h	47 y M				A/C	Ingst	Int-S	1		
		amlodipine	1	1						
		metoprolol (extended release)	2	2						
1365	47 y F				A/C	Ingst	Int-S	1		
		amlodipine	1	1						
		metoprolol	2	2						
		lisinopril	3	3						
		zolpidem	4	4						
1366ha	47 y F				A/C	Ingst	Int-S	1		
		amlodipine	1	1					amlodipine	1800 mcg/L In Blood (unspecified) @ Autopsy
		tizanidine	2	2						
		doxepin	3	3					doxepin	0.13 mg/L In Blood (unspecified) @ Autopsy
		lithium	4	4						
		fluoxetine	5	5					fluoxetine	0.6 mg/L In Blood (unspecified) @ Autopsy
		fluoxetine	5	5					norfluoxetine	1.4 mg/L In Blood (unspecified) @ Autopsy
		gabapentin	6	6						
		acetaminophen/hydrocodone	7	7						
		flurazepam	8	8						
1367	47 y F				A/C	Ingst	Int-S	1		
		verapamil	1	1						
1368ai	48 y F				A	Ingst	Int-S	2		
		diltiazem	1	1						
		metoprolol	2	2						
		amlodipine	3	3						
		cocaine	4	4						
		ethanol (non-beverage)	5	5						
1369h	48 y M				A/C	Ingst	Int-S	1		
		amlodipine	1	1						
		lamotrigine	2	2						
		lisinopril	3	3						
		risperidone	4	4						
		quetiapine	5	5						
		omeprazole	6	6						
1370	48 y M				A	Ingst	Int-S	1		
		verapamil	1	1						
1371	48 y M				A	Ingst	Int-S	2		
		amlodipine	1	1						
		metoprolol	2	2						
1372i	48 y M				A	Ingst	Int-S	2		
		lisinopril	1	1						
		valproic acid	2	2						
		sertraline	3	3						
1373h	48 y M				A/C	Ingst	Int-S	1		
		amlodipine	1	1						
1374	48 y F				A/C	Ingst	Int-S	1		
		propranolol	1	1						
		lisinopril	2	2						
		alprazolam	3	3						
1375h	49 y F				C	Ingst	Int-S	2		
		verapamil	1	1						
		clozapine	2	2						
1376a	49 y M				A/C	Ingst	Int-S	1		
		verapamil	1	1					verapamil	610 ng/mL In Blood (unspecified) @ 1 h (pe)
		valproic acid (extended release)	2	2					valproic acid	10 mcg/mL In Serum @ Unknown
		ethanol	3	3					ethanol	37 mg/dL In Serum @ Unknown
		amlodipine/valsartan	4	4						
		valproic acid	5	5						
1377ai	49 y F				A	Ingst	Int-S	2		
		verapamil	1	1						
		clonazepam	2	2						
1378h	49 y F				U	Ingst	Unk	3		
		metoprolol	1	1						
1379	50 y F				A/C	Ingst	Int-S	2		
		atenolol	1	1						
1380ha	50 y F				A	Ingst	Int-S	1		
		verapamil	1	1						
		metoprolol	2	2						
		furosemide	3	3						
[1381a]	51 y F				U	Ingst	Int-S	1		
		amlodipine/benazepril	1	1					amlodipine	1300 ng/mL In Blood (unspecified) @ Unknown
1382	52 y M				A/C	Ingst	Int-S	1		
		diltiazem (extended release)	1	1						
		metoprolol	2	2						
		citalopram	3	3						
		gabapentin	4	4						
		mirtazapine	5	5						
		hydroxyzine	6	6						
		ethanol	7	7						
		omeprazole	8	8						
		salicylate	9	9						
1383	52 y M				A/C	Ingst	Int-S	3		
		propranolol	1	1						
		ethanol	2	2						
1384	52 y F				A/C	Ingst	Int-S	1		
		verapamil	1	1						
1385h	53 y F				A/C	Ingst	Int-S	2		
		verapamil	1	1						
		venlafaxine	2	2						
		ethanol	3	3						
1386h	53 y F				A/C	Ingst	Int-S	2		
		beta blocker	1	1						
1387	54 y F				A	Ingst	Int-S	2		
		beta blocker	1	1						
		carvedilol	2	2						
		quetiapine	3	3						
		angiotensin converting enzyme inhibitor	4	4						
		desfenlafaxine	5	5						
		acetaminophen/hydrocodone	6	6						
		salicylate	7	7						
		warfarin	8	8						
		bupropion	9	9						
		simvastatin	10	10						
1388h	54 y F				C	Ingst	Unk	3		
		digoxin	1	1					digoxin	2.4 ng/mL In Blood (unspecified) @ Unknown
1389h	54 y F				A	Ingst	Int-S	1		
		metoprolol	1	1						
1390h	54 y F				A	Ingst	AR-D	2		
		nadolol	1	1						
		sildenafil	2	2						
1391a	55 y M				A/C	Ingst	Int-S	1		
		metoprolol	1	1					metoprolol	39000 ng/mL In Blood (unspecified) @ Autopsy
		antihyperlipidemic	2	2						
1392	55 y F				A/C	Ingst	Int-S	2		
		amlodipine	1	1						
1393h	55 y F				A	Ingst	Int-S	1		
		calcium antagonist	1	1						
1394a	55 y M				U	Ingst	Unk	2		
		carvedilol	1	1						
		metformin	2	2					metformin	100 mcg/mL In Blood (unspecified) @ 1 h (pe)
		pesticide, unknown	3	3						
		ethanol	4	4					ethanol	0.041 g/dL In Blood (unspecified) @ 1 h (pe)
		prasugrel	5	5						
1395a	56 y F				U	Ingst	Int-S	2		
		atenolol	1	1						
		doxepin	2	2						
		promethazine	3	3						
		acetaminophen/hydrocodone	4	4					hydrocodone	6.4 mg/mL In Serum @ Unknown
		lorazepam	5	5					lorazepam	114 ng/mL In Serum @ Unknown
1396	56 y M				A/C	Ingst	Int-S	1		
		diltiazem	1	1						
1397	56 y M				A	Ingst	Int-S	1		
		carvedilol	1	1						
		amlodipine	2	2						
		bupropion (extended release)	3	3						
		doxepin	4	4						
		sertraline	5	5						
		simvastatin	6	6						
		ethanol	7	7						
1398a	56 y M				A/C	Ingst+ Unk	Int-S	1		
		atenolol	1	1						
		amlodipine	2	2						
		hydrochlorothiazide	3	3						
		lisinopril	4	4						
1399h	57 y M				A/C	Ingst	Int-S	1		
		amlodipine	1	1						
		sertraline	2	2						
1400	57 y F				A	Ingst	Int-S	1		
		verapamil	1	1						
		metoprolol	2	2						
		clonazepam	3	3						
1401h	57 y M				C	Ingst	Int-S	3		
		propranolol	1	1						
1402ph	58 y M				A/C	Ingst	Int-S	2		
		isradipine	1	1						
		sildenafil	2	2						
		ethanol *	3	3						
		hurricane related *	4	3						
1403p	58 y M				A/C	Ingst	Int-S	1		
		amlodipine	1	1						
		morphine	2	2						
		hydromorphone	3	3						
		tramadol	4	4						
		theophylline	5	5					theophylline	21.4 mcg/mL In Blood (unspecified) @ 22 h (pe)
		theophylline	5	5					theophylline	37 mcg/mL In Blood (unspecified) @ 3 h (pe)
		theophylline	5	5					theophylline	39.4 mcg/mL In Blood (unspecified) @ 8 h (pe)
		fluphenazine	6	6						
		citalopram	7	7						
		guaifenesin/pseudoephedrine	8	8						
1404ha	58 y M				A/C	Ingst	Int-S	2		
		carvedilol	1	1						
		ethanol	2	2					ethanol	445 mg/dL In Blood (unspecified) @ Autopsy
		trazodone	3	3					trazodone	0.95 mcg/mL In Blood (unspecified) @ Autopsy
		fluoxetine	4	4					fluoxetine	1.6 mcg/mL In Blood (unspecified) @ Autopsy
		zolpidem	5	5					zolpidem	0.51 mcg/mL In Blood (unspecified) @ Autopsy
1405ha	58 y F				A/C	Ingst	Int-S	1		
		verapamil	1	1						
		topiramate	2	2					topiramate	18 mg/L In Blood (unspecified) @ Unknown
1406h	59 y M				A/C	Ingst	Int-S	1		
		diltiazem	1	1						
		ramipril	2	2						
		paroxetine	3	3						
		ethanol	4	4					ethanol	222 mg/dL In Serum @ Unknown
[1407ha]	59 y M				A	Ingst	Int-S	1		
		verapamil	1	1					verapamil	1500 ng/mL In Serum @ Unknown
1408pha	59 y F				A/C	Ingst	Int-U	2		
		propranolol	1	1						
		citalopram	2	2						
		lamotrigine	3	3					lamotrigine	43.9 mcg/mL In Blood (unspecified) @ Unknown
		buspirone	4	4						
1409h	59 y F				U	Ingst	Unt-G	1		
		amlodipine	1	1						
		fluoxetine/olanzapine	2	2						
		clonidine	3	3						
1410h	60 y M				A	Ingst	Int-S	2		
		metoprolol	1	1						
		lisinopril	2	2						
		lorazepam	3	3						
		acetaminophen	4	4					acetaminophen	218 mcg/mL In Blood (unspecified) @ Unknown
		isopropanol	5	5						
		shampoo	6	6						
[1411ha]	60 y M				A/C	Ingst	Int-S	1		
		diltiazem	1	1					diltiazem	8.5 mg/L In Blood (unspecified) @ Unknown
1412	60 y F				A	Ingst	Int-S	1		
		diltiazem	1	1						
		atenolol	2	2						
		zolpidem	3	3						
		hydrochlorothiazide	4	4						
		alprazolam	5	5						
		lorazepam	6	6						
1413p	61 y M				U	Ingst	Int-S	1		
		amlodipine	1	1						
		atenolol	2	2						
		clonazepam	3	3						
1414	62 y M				A	Ingst	Int-S	2		
		diltiazem	1	1						
		metformin	2	2						
1415h	62 y F				A/C	Ingst	Int-S	2		
		verapamil	1	1						
		ethanol	2	2						
1416ha	63 y F				A	Ingst	Int-S	1		
		calcium antagonist	1	1					amlodipine	220 ng/mL In Blood (unspecified) @ Autopsy
		bupropion (extended release)	2	2					bupropion	13 ng/mL In Blood (unspecified) @ Autopsy
		bupropion	3	3					hydroxybupropion	1000 ng/mL In Blood (unspecified) @ Autopsy
		clonazepam	4	4					clonazepam	140 ng/mL In Blood (unspecified) @ Autopsy
		clonazepam	4	4					7-aminoclonazepam	67 ng/mL In Blood (unspecified) @ Autopsy
		lamotrigine	5	5					lamotrigine	21 mcg/mL In Blood (unspecified) @ Autopsy
		antidepressant (SSRI)	6	6					norfluoxetine	240 ng/mL In Blood (unspecified) @ Autopsy
		antidepressant (SSRI)	6	6					fluoxetine	340 ng/mL In Blood (unspecified) @ Autopsy
1417	63 y F				A	Par	AR-D	2		
		metroprolol	1	1						
1418h	63 y F				U	Unk	Unk	2		
		sotalol	1	1						
1419h	64 y M				C	Ingst	AR-D	2		
		flecainide	1	1						
1420	64 y F				A/C	Ingst	Int-S	3		
		amlodipine	1	1						
		bupropion	2	2						
1421ha	65 y F				A/C	Ingst	Int-S	1		
		amlodipine	1	1						
		venlafaxine	2	2					o-desmethylvenlafaxine	1800 ng/mL In Blood (unspecified) @ Unknown
		venlafaxine	2	2					venlafaxine	2300 ng/mL In Blood (unspecified) @ Unknown
		buspirone	3	3						
		zolpidem	4	4					zolpidem	1200 ng/mL In Blood (unspecified) @ Unknown
		lorazepam	5	5					lorazepam	0.5 mg/L In Blood (unspecified) @ Unknown
		temazepam	6	6					temazepam	1.09 mg/L In Blood (unspecified) @ Unknown
1422	65 y F				A	Ingst	Int-S	1		
		calcium antagonist	1	1						
		zolpidem	2	2						
		acetaminophen	3	3					acetaminophen	45 mcg/mL In Serum @ Unknown
1423h	65 y F				C	Ingst	AR-D	3		
		cardiac glycoside	1	1					digoxin	4.6 ng/mL In Blood (unspecified) @ Unknown
1424	66 y M				A	Ingst	Int-S	2		
		verapamil	1	1						
		atenolol	2	2						
		gabapentin	3	3						
		fluoxetine	4	4						
		acetaminophen/tramadol	5	5						
		pantoprazole	6	6						
		prednisone	7	7						
1425	66 y F				A/C	Ingst	Unk	3		
		carvedilol	1	1						
		tapentadol (extended release)	2	2						
		naloxone	3	3						
		lisinopril	4	4						
1426p	67 y F				A/C	Ingst	Int-S	1		
		calcium antagonist	1	1						
		salicylate	2	2					salicylate	12.3 mg/dL In Blood (unspecified) @ Unknown
		alprazolam	3	3						
1427h	67 y M				A/C	Ingst	Int-S	1		
		metoprolol	1	1						
		nifedipine	2	2						
		ethanol	3	3						
1428	68 y F				A	Ingst	Int-S	3		
		diltiazem	1	1						
		metoprolol (extended release)	2	2						
1429	68 y M				A	Ingst	Int-S	2		
		diltiazem (extended release)	1	1						
1430	69 y M				A/C	Ingst	Int-S	2		
		olmesartan	1	1						
		fluoxetine	2	2						
1431ha	69 y M				A	Ingst	Int-S	2		
		amlodipine	1	1						
1432	69 y F				A/C	Ingst	Int-S	1		
		amlodipine	1	1						
1433	69 y F				U	Ingst	Int-S	2		
		propafenone	1	1						
1434h	69 y F				A/C	Ingst	Int-S	1		
		amlodipine	1	1						
		ethanol	2	2					ethanol	240 mg/dL In Blood (unspecified) @ Unknown
		celecoxib	3	3						
1435h	69 y F				C	Ingst	AR-D	2		
		flecainide	1	1						
1436h	69 y F				A/C	Ingst	Int-S	1		
		amlodipine	1	1						
		propafenone	2	2						
		fluoxetine	3	3						
		lisinopril	4	4						
		salicylate	5	5						
		diazepam	6	6						
1437h	69 y M				A/C	Ingst	Int-S	1		
		cardiac glycoside	1	1					digoxin	8.7 ng/mL In Serum @ 4 h (pe)
		clonazepam	2	2						
		trazodone	3	3						
		oxybutynin	4	4						
		quetiapine	5	5						
		lamotrigine	6	6						
		escitalopram	7	7						
		finasteride	8	8						
		salicylate	9	9						
1438	71 y M				A/C	Ingst	Int-S	2		
		metoprolol	1	1						
		quetiapine	2	2						
1439	71 y M				A	Ingst	Int-S	1		
		propranolol	1	1						
		tramadol	2	2						
		fluoxetine	3	3						
		ethanol	4	4						
1440	72 y M				A/C	Ingst	Unt-T	3		
		carvedilol	1	1						
		losartan	2	2						
		lorazepam	3	3						
		simvastatin	4	4						
		pantoprazole	5	5						
1441	73 y M				U	Ingst	AR-D	3		
		cardiac glycoside	1	1					digoxin	4.5 ng/mL In Blood (unspecified) @ 24 h (pe)
1442a	73 y F				A	Ingst	Unt-T	1		
		verapamil	1	1					verapamil	2500 ng/mL In Blood (unspecified) @ Unknown
1443h	73 y F				C	Ingst	AR-D	3		
		digoxin	1	1					digoxin	7.6 ng/mL In Blood (unspecified) @ Unknown
1444h	74 y M				U	Unk	Unk	3		
		atenolol	1	1						
		benzodiazepine	2	2						
		escitalopram	3	3						
		primidone	4	4						
1445a	74 y F				A	Ingst	Int-S	2		
		amlodipine	1	1						
		acetaminophen/hydrocodone	2	2						
1446h	74 y M				C	Par	AR-D	3		
		cardiac glycoside	1	1					digoxin	3.1 ng/mL In Blood (unspecified) @ Unknown
1447h	74 y M				A	Ingst	Unt-G	3		
		verapamil	1	1						
		warfarin	2	2						
		lisinopril	3	3						
1448	74 y M				A/C	Ingst	Int-S	3		
		diltiazem	1	1						
		alprazolam	2	2						
1449pa	75 y M				A	Ingst	Int-S	1		
		metoprolol	1	1					metoprolol	3842 ng/mL In Blood (unspecified) @ Unknown
		flecainide	2	2					flecainide	2.29 mcg/mL In Blood (unspecified) @ Unknown
		rivaroxaban	3	3						
		donepezil	4	4					donepezil	150 ng/mL In Blood (unspecified) @ Unknown
		caffeine	5	5						
1450	75 y F				A/C	Ingst	Unt-T	2		
		metoprolol	1	1						
1451h	77 y F				A	Unk	Unk	3		
		clonidine	1	1						
		metoprolol	2	2						
		nifedipine	3	3						
1452	78 y M				A	Ingst	Unt-T	2		
		cardiac glycoside	1	1						
		warfarin	2	2						
1453	79 y F				A/C	Ingst	Unt-T	3		
		metoprolol (extended release)	1	1						
		amlodipine	2	2						
		losartan	3	3						
		warfarin	4	4						
		furosemide	5	5						
1454h	79 y F				A/C	Ingst	Int-S	3		
		amlodipine	1	1						
		metoprolol	2	2						
1455p	79 y M				A/C	Ingst	Int-S	2		
		metoprolol	1	1						
		alprazolam	2	2						
		sumatriptan	3	3						
1456h	79 y F				U	Ingst	Unk	2		
		calcium antagonist	1	1						
1457	79 y M				A	Ingst	AR-D	3		
		cardiac glycoside	1	1						
1458ph	80 y F				C	Ingst	Unt-T	1		
		cardiac glycoside	1	1					digoxin	1.6 ng/mL In Blood (unspecified) @ 3 d (pe)
		cardiac glycoside	1	1					digoxin	1.9 ng/mL In Blood (unspecified) @ 2 d (pe)
		cardiac glycoside	1	1					digoxin	2 ng/mL In Blood (unspecified) @ 5 h (pe)
		cardiac glycoside	1	1					digoxin	3.1 ng/mL In Blood (unspecified) @ 18 h (pe)
		cardiac glycoside	1	1					digoxin	4.1 ng/mL In Blood (unspecified) @ 0 h (pe)
1459	81 y F				A/C	Ingst	Int-U	2		
		diltiazem	1	1						
		cyclobenzaprine	2	2						
		angiotensin receptor blocker	3	3						
		diazepam	4	4						
1460i	82 y M				C	Ingst+ Par	AR-D	3		
		cardiac glycoside	1	1					digoxin	3.19 ng/mL In Blood (unspecified) @ Unknown
1461	82 y M				A/C	Ingst	Unt-U	3		
		metoprolol	1	1						
		cyclobenzaprine	2	2						
		drug, unknown	3	3						
1462ha	83 y M				A	Ingst	Int-S	1		
		sotalol	1	1						
1463h	83 y M				A	Ingst	AR-D	3		
		cardiac glycoside	1	1					digoxin	3.5 ng/mL In Plasma @ Unknown
1464	83 y F				A	Ingst	Int-S	2		
		beta blocker	1	1						
		acetaminophen/hydrocodone	2	2						
1465	84 y F				C	Ingst+ Par	AR-D	3		
		cardiac glycoside	1	1					digoxin	6.7 ng/mL In Blood (unspecified) @ Unknown
1466	85 y F				C	Ingst	AR-D	3		
		atenolol	1	1						
		lisinopril	2	2						
		amlodipine	3	3						
		diltiazem (extended release)	4	4						
		sertraline	5	5						
		nitroglycerin	6	6						
		furosemide	7	7						
		warfarin	8	8						
		zolpidem	9	9						
		atorvastain	10	10						
		donepezil	11	11						
1467	85 y F				U	Ingst	Unk	3		
		cardiac glycoside	1	1					digoxin	3.3 ng/mL In Blood (unspecified) @ Unknown
1468h	85 y F				A	Ingst	Int-S	1		
		amlodipine	1	1						
1469	86 y M				A/C	Ingst	AR-D	3		
		cardiac glycoside	1	1					digoxin	2.8 ng/mL In Serum @ Unknown
1470h	86 y M				A/C	Ingst	Unt-T	1		
		diltiazem	1	1						
		diltiazem (extended release)	2	2						
		bupropion (extended release)	3	3						
1471	86 y F				C	Ingst	AR-D	2		
		cardiac glycoside	1	1					digoxin	2.8 ng/mL In Serum @ Unknown
		metoprolol	2	2						
		warfarin	3	3						
		ibuprofen	4	4						
1472	86 y F				C	Ingst	Unt-T	3		
		digoxin	1	1					digoxin	4.9 ng/mL In Blood (unspecified) @ Unknown
1473ph	86 y M				U	Ingst	Unk	2		
		amlodipine	1	1						
1474a	87 y F				A/C	Ingst	Int-S	2		
		cardiac glycoside	1	1					digoxin	53 ng/mL In Blood (unspecified) @ 2 m (pe)
		carvedilol	2	2						
		diltiazem	3	3					diltiazem	1700 ng/mL In Blood (unspecified) @ 2 m (pe)
		warfarin	4	4						
1475h	87 y F				C	Ingst+ Par	AR-D	3		
		carvedilol	1	1						
		labetalol	2	2						
1476	88 y M				A/C	Ingst	Int-S	2		
		amlodipine	1	1						
		atenolol	2	2						
1477	88 y F				A/C	Ingst	AR-D	1		
		digoxin	1	1					digoxin	5 ng/mL In Blood (unspecified) @ Unknown
		warfarin	2	2						
		thrombin inhibitor	3	3						
1478	89 y M				A/C	Ingst	Int-S	3		
		diltiazem	1	1						
		tamulosin	2	2						
		clonazepam	3	3						
1479	89 y F				A/C	Ingst	AR-F	3		
		cardiac glycoside	1	1						
1480a	90 y F				A/C	Ingst	Int-S	1		
		diltiazem	1	1					diltiazem	6200 ng/mL In Blood (unspecified) @ Unknown
1481	91 y F				A	Ingst	Unt-T	2		
		nifedipine	1	1						
		beta blocker	2	2						
1482	92 y M				C	Ingst	Int-U	3		
		cardiac glycoside	1	1					digoxin	2.18 ng/mL In Serum @ Unknown
		warfarin	2	2						
1483pha	50 + y M				A/C	Ingst	Int-S	1		
		metoprolol	1	1					metoprolol	62000 mcg/mL In Whole Blood @ Autopsy
		ethanol	2	2					ethanol	201 mg/dL In Whole Blood @ Autopsy
		carbamazepine	3	3					carbamazepine	2.8 mcg/mL In Whole Blood @ Autopsy
		sertraline	4	4					norsertraline	30 ng/mL In Whole Blood @ Autopsy
1484	60 + y F				A/C	Ingst	Int-S	2		
		calcium antagonist	1	1						
		atenolol	2	2						
		nitroglycerin	3	3						
		salicylate	4	4						
		disc battery, lithium	5	5						
		drug, unknown	6	6						
1485pi	Unknown adult (> = 20 yrs) M				A	Ingst	Unt-O	2		
		verapamil	1	1						
See Also case 48, 84, 87, 102, 111, 190, 244, 311, 321, 343, 446, 486, 544, 568, 632, 661, 679, 687, 723, 736, 748, 749, 765, 774, 789, 808, 836, 903, 908, 923, 967, 979, 993, 1047, 1112, 1118, 1121, 1128, 1134, 1135, 1139, 1159, 1172, 1191, 1199, 1200, 1203, 1208, 1214, 1228, 1233, 1235, 1267, 1273, 1295, 1500, 1528, 1534, 1540, 1581, 1606, 1607, 1618, 1641, 1642, 1647, 1648, 1650, 1652, 1653, 1744, 1752, 1758, 1811, 1846, 1876, 1989, 2002, 2004, 2025, 2033, 2049, 2053, 2059, 2076, 2102, 2108										
**Cold and Cough Preparations**										
1486ph	15 y F				A	Ingst+ Unk	Int-A	2		
		chlorpheniramine/dextromethorphan	1	1						
		methadone	2	2						
1487pha	18 y F				A	Ingst	Int-A	1		
		codeine/promethazine	1	1					morphine (free)	240 ng/mL In Serum @ 10 h (pe)
1488ai	18 y F				A	Ingst	Int-A	2		
		dextromethorphan	1	1						
1489a	18 y M				A	Ingst	Int-S	3		
		acetaminophen/dextromethorphan/doxalamine	1	1					acetaminophen	12 mcg/mL In Blood (unspecified) @ Unknown
		diphenhydramine	2	2						
		ethanol (non-beverage)	3	3						
1490ai	25 y M				A	Ingst	Int-U	2		
		dextromethorphan	1	1						
		chlorpheniramine	2	2						
1491	31 y M				A	Ingst	Int-A	2		
		cough and cold preparation	1	1						
		amphetamine (hallucinogenic)	2	2						
1492ha	35 y M				C	Ingst	Unt-T	2		
		acetaminophen/decongestant/salicyate/	1	1					diphenhydramine	0.393 mg/L In Blood (unspecified) @ Unknown
		acetaminophen/decongestant/salicyate/	1	1					salicylate	11 mg/dL In Blood (unspecified) @ Unknown
		acetaminophen/decongestant/salicyate/	1	1					acetaminophen	38 mcg/mL In Blood (unspecified) @ Unknown
1493ai	41 y M				U	Ingst	Int-A	2		
		doxylamine	1	1						
1494ai	42 y F				A	Ingst+ Unk	Int-A	2		
		dextromethorphan	1	1						
		diphenhydramine	2	2						
		doxepin	3	3						
		doxylamine	4	4						
		quetiapine	5	5						
		citalopram	6	6						
		fentanyl	7	7						
		buspirone	8	8						
		acetaminophen	9	9						
		isopropanol	10	10						
1495ai	44 y M				A	Ingst	Int-A	2		
		dextromethorphan	1	1						
		diphenhydramine	2	2						
1496p	45 y F				A	Ingst	AR-D	1		
		cough and cold preparation	1	1						
1497h	47 y M				A/C	Ingst	Int-U	3		
		acetaminophen/dextromethorphan/doxylamine	1	1					acetaminophen	3.5 mcg/mL In Blood (unspecified) @ Unknown
		ethanol	2	2						
		ethanol	3	3						
1498	68 y M				A	Ingst	Int-S	1		
		benzonatate	1	1						
		folic acid	2	2						
		salicylate	3	3					salicylate	40.7 mg/dL In Blood (unspecified) @ Unknown
		salicylate	3	3					salicylate	48.2 mg/dL In Blood (unspecified) @ Unknown
		furosemide	4	4						
1499	82 y M				A/C	Ingst	Unt-T	3		
		codeine/promethazine	1	1						
See Also case 8, 9, 38, 43, 71, 81, 95, 163, 282, 347, 357, 358, 412, 423, 426, 437, 467, 473, 520, 546, 551, 561, 565, 571, 582, 590, 625, 689, 702, 706, 759, 801, 862, 865, 1031, 1163, 1170, 1172, 1194, 1195, 1204, 1229, 1235, 1252, 1263, 1297, 1308, 1403, 1565, 1622, 1656, 1697, 1721, 1757, 1797, 1798, 1801, 1807, 1832, 1875, 1885, 1895, 1913, 1914, 1917, 1923, 1927, 1943, 1945, 1966, 1972, 1990, 2015, 2041, 2051, 2052										
**Diuretics**										
1500h	51 y F				A	Ingst	Int-S	2		
		thiazide	1	1						
		anticonvulsant, unknown	2	2						
		acetaminophen/diphenhydramine	3	3						
		calcium antagonist	4	4						
See Also case 625, 832, 1112, 1264, 1355, 1380, 1398, 1412, 1453, 1466, 1498, 1556, 1648										
**Electrolytes and Minerals**										
[1501h]	33 y M				A	Ingst	Int-A	1		
		sodium bicarbonate	1	1						
1502	77 y F				A	Ingst	Unt-G	2		
		sodium bicarbonate	1	1						
										
**Eye/Ear/Nose/Throat Preparations**										
1503	Unknown age U				A	Ingst	Oth-M	2		
		naphazoline/pheniramine	1	1						
		ethanol	2	2						
										
**Gastrointestinal Preparations**										
1504p	19 y M				A/C	Ingst	AR-D	3		
		glycopyrrolate	1	1						
		fluoxetine	2	2						
		methylphenidate	3	3						
		diphenhydramine	4	4						
1505ha	29 y M				A	Ingst	Int-U	2		
		loperamide	1	1						
1506ha	37 y F				A	Ingst	Int-S	2		
		loperamide	1	1						
		escitalopram	2	2						
		meloxicam	3	3						
See Also case 484, 711, 784, 798, 817, 875, 923, 1031, 1112, 1180, 1189, 1295, 1351, 1354, 1369, 1382, 1424, 1437, 1440, 1531, 1568, 1596, 1600, 1621										
**Hormones and Hormone Antagonists**										
1507	19 y M				A	Ingst	Int-S	1		
		metformin	1	1						
1508pa	19 y F				A	Ingst	Int-S	1		
		metformin	1	1					metformin	57 mcg/mL In Blood (unspecified) @ Autopsy
1509h	27 y M				A	Ingst	Int-S	2		
		metformin	1	1						
1510h	36 y F				A/C	Ingst	Int-S	3		
		metformin	1	1						
		acetaminophen/butalbital/caffeine	2	2						
		topiramate	3	3						
		venlafaxine	4	4						
		clonazepam	5	5						
1511h	37 y F				A/C	Par	Int-S	1		
		insulin	1	1						
1512a	38 y F				A	Ingst	Int-S	1		
		metformin	1	1					metformin	210 mcg/mL In Blood (unspecified) @ Autopsy
		doxepin	2	2					nordoxepin	0.22 mg/L In Blood (unspecified) @ Autopsy
		doxepin	2	2					doxepin	2 mg/L In Blood (unspecified) @ Autopsy
1513a	43 y M				U	Ingst	Int-U	2		
		metformin	1	1						
		ethanol	2	2					ethanol	68 mg/dL In Blood (unspecified) @ Unknown
1514	43 y M				A	Ingst	Int-S	2		
		insulin	1	1						
		salicylate	2	2					salicylate	56 mg/dL In Blood (unspecified) @ 7 h (pe)
		doxylamine	3	3						
1515h	46 y M				A/C	Ingst	Int-S	1		
		metformin	1	1						
1516h	46 y M				A/C	Derm	Int-S	1		
		insulin	1	1						
		insulin	2	2						
		amphetamine/dextroamphetamine	3	3						
1517h	48 y M				C	Ingst	AR-D	2		
		methimazole	1	1						
		acetaminophen	2	2						
1518h	48 y F				C	Ingst	AR-D	1		
		propylthiouracil	1	1						
1519h	50 y M				A/C	Ingst	Int-S	2		
		metformin	1	1						
		lorazepam	2	2						
		fluoxetine	3	3						
		zolpidem	4	4						
1520a	50 y F				C	Ingst	Int-M	2		
		metformin	1	1						
		ethanol	2	2						
1521	51 y M				A/C	Par	Unk	2		
		insulin	1	1						
1522ha	52 y M				A/C	Par	Int-S	1		
		insulin	1	1						
1523	52 y M				A/C	Unk	Int-S	1		
		insulin	1	1						
		glipizide	2	2						
1524i	53 y M				A	Ingst	Int-S	2		
		glyburide	1	1						
		metformin	2	2						
1525ha	54 y F				A	Ingst	Int-S	1		
		metformin	1	1						
1526pha	58 y M				A	Ingst	Int-S	2		
		metformin	1	1						
		glibenclamide	2	2						
1527ha	59 y F				A/C	Ingst	Int-S	1		
		metformin	1	1					metformin	100 mg/L In Blood (unspecified) @ Autopsy
		metformin	1	1					metformin	990 mg/kg In Gastric (stomach content) @ Autopsy
		quetiapine	2	2					quetiapine	100 mg/L In Gastric (stomach content) @ Autopsy
		quetiapine	2	2					quetiapine	9.079 mg/L In Blood (unspecified) @ Autopsy
		trazodone	3	3					trazodone	1.6 mg/L In Blood (unspecified) @ Autopsy
		trazodone	3	3					trazodone	50.1 mg/kg In Gastric (stomach content) @ Autopsy
		sitagliptin	4	4						
1528	61 y F				A/C	Ingst	Int-S	1		
		metformin	1	1						
		glibenclamide	2	2						
		lisinopril	3	3						
		venlafaxine	4	4						
		levothyroxine	5	5						
		famotidine	6	6						
		atorvastatin	7	7						
1529p	63 y M				A	Par	Int-S	2		
		insulin	1	1						
		insulin	2	2						
1530	63 y M				A	Ingst	Int-S	2		
		metformin	1	1						
		temazepam	2	2						
1531	66 y F				A	Ingst	Int-S	2		
		metformin	1	1						
		acetaminophen/oxycodone	2	2						
		lorazepam	3	3						
		tramadol	4	4						
		gabapentin	5	5						
		bupropion	6	6						
		diphenhydramine	7	7						
		ibuprofen	8	8						
		oxybutynin	9	9						
		thyroid preparation	10	10						
		estrogens, conjugated	11	11						
1532	66 y M				C	Ingst	AR-D	2		
		metformin	1	1						
1533h	69 y M				A	Ingst	Int-S	1		
		glyburide	1	1						
		metformin	2	2						
1534	70 y M				A/C	Ingst	Int-S	2		
		metformin	1	1						
		glipizide	2	2						
		angiotensin converting enzyme inhibitor	3	3						
		simvastatin	4	4						
1535	80 y F				A	Ingst	AR-D	2		
		metformin/sitagliptin	1	1						
1536	84 y F				A/C	Ingst	Int-S	2		
		metformin	1	1						
See Also case 84, 121, 142, 521, 568, 648, 661, 765, 768, 774, 944, 1180, 1200, 1262, 1267, 1273, 1311, 1327, 1346, 1354, 1355, 1361, 1394, 1414, 1424, 1599, 1618										
**Miscellaneous Drugs**										
1537ai	42 y F				A	Ingst+ Par	Int-S	2		
		curare and related	1	1						
		tramadol	2	2						
		midazolam	3	3						
1538p	50 y M				A	Par	AR-D	1		
		peginasetide	1	1						
1539pha	54 y M				A/C	Ingst	Int-S	3		
		varenicline	1	1						
		acetaminophen	2	2					acetaminophen	40 mcg/mL In Blood (unspecified) @ Unknown
		ethanol	3	3					ethanol	236 mg/dL In Blood (unspecified) @ Unknown
		hydrocodone	4	4					hydrocodone	0.047 mg/L In Urine (quantitative only) @ Unknown
		hydrocodone	4	4					hydrocodone	0.196 mg/L In Blood (unspecified) @ Unknown
1540p	56 y F				A	Ingst	Int-S	1		
		ropinirole	1	1						
		acetaminophen/hydrocodone	2	2						
		clonidine	3	3						
		diazepam	4	4						
		amlodipine	5	5						
1541ai	81 y F				U	Ingst	Int-A	2		
		memantine	1	1						
		chlordiazepoxide	2	2						
		diazepam	3	3						
1542	7 d M				A	Par	Unt-T	3		
		lipid emulsion	1	1						
See Also case 84, 244, 508, 774, 798, 1031, 1129, 1190, 1197, 1256, 1283, 1354, 1437, 1449, 1455, 1466, 1582, 1618										
**Muscle Relaxants**										
1543ph	22 y M				A	Ingst+ Aspir	Int-S	2		
		baclofen	1	1						
1544ai	27 y M				U	Ingst	Int-A	2		
		skeletal muscle relaxant	1	1						
		acetaminophen/hydrocodone	2	2						
		hydromorphone	3	3						
		temazepam	4	4						
1545	31 y M				A	Ingst	Int-S	2		
		cyclobenzaprine	1	1						
		diazepam	2	2						
[1546pha]	41 y F				U	Ingst	Int-S	1		
		carisoprodol	1	1					carisoprodol	19 mg/L In Blood (unspecified) @ 12 h (pe)
		carisoprodol	1	1					meprobamate	35 mg/L In Blood (unspecified) @ 12 h (pe)
		carisoprodol	1	1					meprobamate	43 mg/L In Blood (unspecified) @ 12 h (pe)
		carisoprodol	1	1					meprobamate	46 mg/kg In Serum @ 12 h (pe)
		carisoprodol	1	1					carisoprodol	6.7 mg/L In Blood (unspecified) @ 12 h (pe)
		meloxicam	2	2						
1547a	41 y F				U	Ingst	Int-S	1		
		cyclobenzaprine	1	1					cyclobenzaprine	590 ng/mL In Blood (unspecified) @ Autopsy
		oxycodone	2	2					oxycodone (free)	680 ng/mL In Blood (unspecified) @ Autopsy
		morphine (extended release)	3	3					morphine (free)	230 ng/mL In Blood (unspecified) @ Autopsy
		doxylamine	4	4					doxylamine	130 ng/mL In Blood (unspecified) @ Autopsy
1548ai	44 y M				U	Ingst	Int-A	2		
		cyclobenzaprine	1	1						
		ethanol	2	2						
		tramadol	3	3						
1549p	44 y F				U	Ingst	Unk	3		
		carisoprodol	1	1						
		acetaminophen	2	2						
		ethanol	3	3						
1550	52 y F				A/C	Ingst	Int-S	2		
		tizanidine	1	1						
		clonazepam	2	2						
		acetaminophen/hydrocodone	3	3					acetaminophen	29 mcg/mL In Serum @ Unknown
1551ai	53 y F				U	Ingst	Int-A	2		
		skeletal muscle relaxant	1	1						
		diphenhydramine	2	2						
		zolpidem	3	3						
1552h	53 y M				A	Ingst	Int-U	2		
		cyclobenzaprine	1	1						
		diazepam	2	2						
1553	54 y F				A	Ingst	Int-S	2		
		cyclobenzaprine	1	1						
		methadone	2	2						
		lorazepam	3	3						
1554ai	55 y F				U	Ingst	Int-A	2		
		cyclobenzaprine	1	1						
		ethanol	2	2						
1555ai	55 y F				U	Ingst	Int-A	2		
		skeletal muscle relaxant	1	1						
		acetaminophen/hydrocodone	2	2						
1556	55 y M				A/C	Ingst	Int-S	2		
		cyclobenzaprine	1	1						
		hydrochlorothiazide	2	2						
1557a	57 y F				A	Ingst	Int-S	3		
		cyclobenzaprine	1	1						
1558h	59 y F				A	Ingst	Int-S	3		
		cyclobenzaprine	1	1						
1559p	60 y M				A	Ingst	Int-S	2		
		skeletal muscle relaxant	1	1						
		lorazepam	2	2						
1560	62 y F				A/C	Ingst	Int-S	2		
		baclofen	1	1						
1561	64 y M				A	Ingst	Int-S	3		
		baclofen	1	1						
1562	65 y M				A/C	Ingst	Unk	2		
		cyclobenzaprine	1	1						
		hydroxyzine	2	2						
		clonazepam	3	3						
1563	75 y F				A	Ingst	Int-S	2		
		carisoprodol	1	1						
		acetaminophen/hydrocodone	2	2					acetaminophen	106 mg/dL In Blood (unspecified) @ Unknown
		acetaminophen/hydrocodone	2	2					acetaminophen	69 mg/dL In Blood (unspecified) @ Unknown
		salicylate	3	3						
1564p	Unknown adult (> = 20 yrs) F				A	Ingst	Int-S	2		
		carisoprodol	1	1						
See also case 14, 43, 100, 419, 425, 426, 433, 434, 446, 452, 479, 496, 510, 541, 544, 546, 548, 551, 557, 568, 576, 579, 602, 606, 618, 620, 625, 628, 635, 640, 648, 663, 669, 670, 674, 675, 685, 692, 693, 711, 726, 728, 740, 748, 765, 767, 792, 802, 826, 827, 841, 850, 855, 857, 863, 876, 877, 879, 880, 884, 889, 909, 912, 921, 923, 924, 930, 933, 979, 983, 991, 1015, 1025, 1141, 1151, 1178, 1189, 1194, 1277, 1281, 1366, 1459, 1461, 1617, 1635, 1847, 1864, 1916, 1924, 1967, 1991, 2002, 2059										
**Sedative/Hypnotics/Antipsychotics**										
1565ha	17 y U				A/C	Ingst	Int-S	2		
		quetiapine	1	1					quetiapine	6970 ng/mL In Whole Blood @ Autopsy
		dextromethorphan	2	2					dextromethorphan	2225 ng/mL In Whole Blood @ Autopsy
		diphenhydramine	3	3					diphenhydramine	421 ng/mL In Whole Blood @ Autopsy
		chlorpheniramine	4	4					chlorpheniramine	266 ng/mL In Whole Blood @ Autopsy
		lamotrigine	5	5						
1566ai	18 y M				U	Ingst+ Inhal	Int-A	2		
		benzodiazepine	1	1						
		marijuana	2	2						
		ethanol	3	3						
1567ph	18 y F				A	Ingst	Int-U	2		
		alprazolam	1	1						
		phencyclidine	2	2						
		opioid	3	3						
		marijuana	4	4						
1568a	19 y M				A	Ingst	Unk	2		
		quetiapine	1	1						
		dicyclomine	2	2						
		trazodone	3	3						
		fluoxetine	4	4					norfluoxetine	0.05 mg/L In Plasma @ Unknown
		fluoxetine	4	4					fluoxetine	0.08 mg/L In Plasma @ Unknown
1569ai	19 y F				U	Ingst	Int-A	2		
		alprazolam	1	1						
		diazepam	2	2						
1570ha	20 y F				U	Ingst	Int-S	1		
		quetiapine	1	1					quetiapine	4419 ng/mL In Blood (unspecified) @ Unknown
		paroxetine	2	2					paroxetine	57.5 ng/mL In Blood (unspecified) @ Unknown
		lamotrigine	3	3					lamotrigine	17 mcg/mL In Blood (unspecified) @ Unknown
1571	20 y M				A/C	Ingst	Int-S	2		
		quetiapine (extended release)	1	1						
1572ai	21 y M				U	Ingst	Int-A	2		
		alprazolam	1	1						
		ethanol	2	2						
1573p	22 y M				A	Ingst	Unk	2		
		olanzapine	1	1						
		hydroxyzine	2	2						
		fluoxetine	3	3						
		zolpidem	4	4						
		flunitrazepam	5	5						
1574ai	23 y M				U	Ingst	Int-A	2		
		benzodiazepine	1	1						
1575ai	23 y F				U	Ingst	Unk	2		
		alprazolam	1	1						
		butalbital	2	2						
		ethanol	3	3						
1576h	24 y M				A/C	Ingst	Int-S	2		
		clonazepam	1	1						
		mirtazapine	2	2						
		cocaine	3	3						
		methamphetamine	4	4						
		marijuana	5	5						
1577	26 y M				U	Ingst	Int-U	2		
		quetiapine	1	1						
		venlafaxine	2	2						
		acetaminophen	3	3					acetaminophen	73 mcg/mL In Serum @ Unknown
		methadone	4	4						
		drug, unknown	5	5						
1578ai	26 y M				A	Ingst	Int-S	2		
		quetiapine	1	1						
		hydrocodone	2	2						
		acetaminophen	3	3						
1579pa	26 y M				A/C	Ingst	Int-A	2		
		alprazolam *	2	1						
		ethanol *	1	1					ethanol	327 mg/dL In Blood (unspecified) @ Autopsy
		ethanol *	1	1					ethanol	455 mg/dL In Vitreous @ Autopsy
1580ai	27 y M				A	Ingst	Int-S	2		
		phenobarbital	1	1						
		oxycodone	2	2						
		quetiapine	3	3						
		hydroxyzine	4	4						
		zolpidem	5	5						
		ethanol	6	6						
1581h	28 y M				A/C	Ingst	Int-S	2		
		risperidone	1	1						
		lisinopril	2	2						
		lorazepam	3	3						
1582ai	28 y F				U	Ingst	Int-A	2		
		clozapine	1	1						
		memantine	2	2						
		citalopram	3	3						
1583ha	28 y M				U	Ingst	Int-S	2		
		quetiapine	1	1					quetiapine	11000 ng/mL In Blood (unspecified) @ Unknown
		diazepam	2	2					oxazepam	0.399 mg/L In Blood (unspecified) @ Unknown
		diazepam	2	2					temazepam	0.42 mg/L In Blood (unspecified) @ Unknown
		diazepam	2	2					diazepam	0.493 mg/L In Blood (unspecified) @ Unknown
		diazepam	2	2					nordiazepam	0.752 mg/L In Blood (unspecified) @ Unknown
1584ai	30 y M				U	Ingst	Int-A	2		
		alprazolam	1	1						
		acetaminophen/hydrocodone	2	2						
		oxycodone	3	3						
1585	30 y F				A	Ingst	Int-S	1		
		quetiapine	1	1						
1586pha	30 y M				A/C	Ingst+ Oth	Int-S	3		
		alprazolam	1	1						
		sertraline	2	2						
		oxycodone	3	3						
1587ai	31 y M				U	Ingst	Int-A	2		
		alprazolam	1	1						
		acetaminophen/hydrocodone	2	2						
		ethanol	3	3						
1588ai	31 y F				A	Ingst	Int-A	2		
		alprazolam	1	1						
		methadone	2	2						
1589	32 y M				A	Ingst+ Inhal	Int-S	2		
		zolpidem (extended release)	1	1					zolpidem	791 ng/mL In Blood (unspecified) @ Autopsy
		ethanol	2	2					ethanol	0.145 % In Blood (unspecified) @ Autopsy
		ethanol	2	2					ethanol	211 mg/dL In Blood (unspecified) @ Unknown
		methamphetamine	3	3					methamphetamine	212 ng/mL In Blood (unspecified) @ Autopsy
		carbon monoxide	4	4						
		escitaopram	5	5					citalopram	119 ng/mL In Blood (unspecified) @ Autopsy
1590ph	33 y M				A	Ingst+ Derm	Int-S	2		
		alprazolam	1	1						
		fentanyl (transdermal)	2	2						
1591h	33 y M				A	Ingst	Int-S	3		
		alprazolam	1	1						
1592pa	35 y M				U	Ingst	Int-U	1		
		alprazolam	1	1					alprazolam	137.6 ng/mL In Whole Blood @ Autopsy
		hydrocodone	2	2					hydrocodone	197 ng/mL In Whole Blood @ Autopsy
1593h	35 y F				A/C	Ingst	Int-S	3		
		quetiapine	1	1						
		gabapentin	2	2						
1594ha	37 y F				A/C	Ingst	Int-S	2		
		diazepam	1	1						
		acetaminophen/hydrocodone	2	2						
		levetiracetam	3	3						
		levofloxacin	4	4						
1595	38 y F				A	Ingst	Int-S	1		
		quetiapine	1	1						
1596ai	38 y M				A	Ingst	Int-S	2		
		pentobarbital	1	1						
		metoclopramide	2	2						
		diphenhydramine	3	3						
		ethanol	4	4						
1597	38 y F				A	Ingst+ Unk	Int-S	2		
		quetiapine	1	1						
		alprazolam	2	2						
		heroin	3	3						
		oxycodone	4	4						
		carbamazepine	5	5						
1598ph	38 y F				A/C	Ingst	Unk	2		
		aripiprazole	1	1						
		alprazolam	2	2						
1599h	39 y F				A/C	Ingst	Int-S	3		
		risperidone	1	1						
		duloxetine	2	2						
		clonazepam	3	3						
		lithium	4	4					lithium	0.2 mEq/L In Blood (unspecified) @ Unknown
		lamotrigine	5	5						
		ziprasidone	6	6						
		medroxyprogesterone	7	7						
1600pha	39 y M				A	Ingst	Int-S	3		
		ziprasidone	1	1						
		amantadine	2	2					amantadine	1 Other (see abst) In Urine (quantitative only) @ 6 d (pe)
		amantadine	2	2					amantadine	1 Other (see abst) In Whole Blood @ 6 d (pe)
		paroxetine	3	3					paroxetine	1 Other (see abst) In Urine (quantitative only) @ 6 d (pe)
		paroxetine	3	3					paroxetine	3.4 mcg/mL In Blood (unspecified) @ 6 d (pe)
		atropine/diphenoxylate	4	4						
		caffeine	5	5					caffeine	1 Other (see abst) In Blood (unspecified) @ 6 d (pe)
		caffeine	5	5					caffeine	1 Other (see abst) In Urine (quantitative only) @ 6 d (pe)
		tramadol	6	6					tramadol	1 Other (see abst) In Urine (quantitative only) @ 6 d (pe)
		hydromorphone	7	7					hydromorphone	42 ng/mL In Blood (unspecified) @ Unknown
1601pha	39 y F				A/C	Ingst	Unt-U	2		
		zolpidem	1	1						
		oxycodone	2	2					oxycodone	122 ng/mL In Blood (unspecified) @ Unknown
1602ai	40 y M				U	Par	Oth-M	2		
		pentobarbital	1	1						
1603h	40 y F				A/C	Ingst	Unk	3		
		alprazolam	1	1						
		drug, unknown	2	2						
1604p	41 y F				A	Ingst	Int-S	2		
		alprazolam	1	1						
1605ai	41 y F				U	Ingst	Int-S	2		
		butalbital	1	1						
		trazodone	2	2						
1606ai	41 y M				U	Ingst	Unk	2		
		clozapine	1	1						
		sertraline	2	2						
		propranolol	3	3						
1607	42 y M				A/C	Ingst	Int-S	3		
		lorazepam	1	1						
		metoprolol	2	2						
		quetiapine	3	3						
		desvenlafaxine	4	4						
		etodolac	5	5						
1608	43 y M				A/C	Ingst	Int-S	2		
		olanzapine	1	1						
1609ai	44 y F				U	Ingst+ Unk	Int-S	2		
		butalbital	1	1						
		lorazepam	2	2						
		diphenhydramine	3	3						
		ethanol	4	4						
		amitriptyline	5	5						
		amphetamine	6	6						
		tramadol	7	7						
1610ai	45 y M				U	Ingst+ Unk	Int-A	2		
		alprazolam	1	1						
		fentanyl	2	2						
		oxycodone	3	3						
		acetaminophen/hydrocodone	4	4						
1611pi	45 y M				U	Unk	Unk	2		
		alprazolam	1	1						
1612	45 y F				A	Ingst	Int-S	3		
		alprazolam *	2	1						
		warfarin *	1	1						
		salicylates in combination	3	3						
1613ai	47 y M				A	Ingst	Int-A	2		
		quetiapine	1	1						
		chlordiazepoxide	2	2						
		zolpidem	3	3						
		trazodone	4	4						
		ethanol	5	5						
1614ai	47 y F				U	Ingst	Int-A	2		
		diazepam	1	1						
1615ai	48 y M				A	Ingst	Int-U	2		
		quetiapine	1	1						
		chlordiazepoxide	2	2						
		caffeine	3	3						
1616	48 y F				A/C	Ingst+ Aspir	Int-S	1		
		fluoxetine/olanzapine	1	1						
1617ai	48 y F				A	Ingst	Int-U	2		
		olanzapine	1	1						
		methadone	2	2						
		cyclobenzaprine	3	3						
		quetiapine	4	4						
1618	49 y F				A	Par	AR-D	3		
		propofol	1	1						
		desmopressin	2	2						
		fentanyl	3	3						
		curare and related	4	4						
		nicardipine	5	5						
		midazolam	6	6						
1619h	50 y F				A/C	Ingst+ Par	Int-S	2		
		paliperidone	1	1						
		dabigatran	2	2						
		quetiapine (extended release)	3	3						
1620ai	50 y F				U	Ingst	Int-A	2		
		alprazolam	1	1						
		oxycodone	2	2						
1621ha	50 y F				A/C	Ingst	Int-S	1		
		zolpidem	1	1						
		clonazepam	2	2						
		ethanol	3	3						
		trazodone	4	4						
		lamotrigine	5	5						
		fluoxetine	6	6						
		buspirone	7	7						
		amphetamine	8	8						
		cocaine	9	9						
		omeprazole	10	10						
1622ai	50 y M				A	Ingst	Int-U	2		
		quetiapine	1	1						
		doxylamine	2	2						
1623ph	51 y M				A/C	Ingst	Int-S	1		
		ziprasidone	1	1						
		methadone	2	2						
		benzodiazepine	3	3						
1624	51 y M				A	Par	AR-D	3		
		haloperidol	1	1						
1625pha	52 y M				A	Ingst	Int-U	3		
		lorazepam	1	1					lorazepam	0.173 mg/L In Blood (unspecified) @ Unknown
1626ai	52 y M				U	Ingst	Int-A	2		
		alprazolam	1	1						
1627ai	52 y F				A	Ingst	Int-S	2		
		alprazolam	1	1						
		acetaminophen	2	2						
		carbamazepine	3	3						
1628ai	53 y F				U	Ingst	Int-A	2		
		alprazolam	1	1						
		duloxetine	2	2						
		quetiapine	3	3						
1629ai	54 y M				U	Ingst	Int-S	2		
		pentobarbital	1	1						
		acetaminophen/hydrocodone	2	2						
1630ha	54 y F				A	Ingst	Int-S	2		
		alprazolam *	2	1					alprazolam	0.14 mg/L In Whole Blood @ Autopsy
		venlafaxine *	1	1					venlafaxine	130 mg/L In Whole Blood @ Autopsy
		chlorpromazine	3	2						
1631ph	55 y F				A/C	Ingst	Int-S	2		
		zolpidem	1	1						
		cocaine	2	2						
		trazodone	3	3						
1632a	55 y F				A	Ingst	Int-S	2		
		clonazepam	1	1						
		alprazolam	2	2					alprazolam	71.7 ng/mL In Blood (unspecified) @ 1 h (pe)
		ethanol	3	3					ethanol	190 mg/dL In Blood (unspecified) @ Autopsy
		ethanol	3	3					ethanol	218 mg/dL In Blood (unspecified) @ 1 h (pe)
1633p	55 y M				A/C	Ingst	Int-S	1		
		zolpidem	1	1						
		pregabalin	2	2						
		drug, unknown	3	3						
1634	55 y M				A/C	Ingst	Int-S	2		
		alprazolam	1	1						
		acetaminophen/hydrocodone	2	2						
1635	55 y F				U	Ingst	Int-S	2		
		alprazolam	1	1						
		tizanidine	2	2						
		sertraline	3	3						
		acetaminophen	4	4						
1636h	56 y M				A	Ingst	Int-S	1		
		phenobarbital	1	1					phenobarbital	300 mcg/mL In Serum @ Unknown
		phenytoin	2	2					phenytoin	3.6 mcg/mL In Serum @ Unknown
		levetiracetam	3	3						
		valproic acid	4	4					valproic acid	62 mcg/mL In Serum @ Unknown
1637p	57 y M				U	Ingst	Int-S	3		
		quetiapine	1	1						
1638ph	57 y F				A/C	Ingst	Unk	2		
		quetiapine	1	1						
		lamotrigine	2	2						
1639pa	57 y F				A	Ingst	AR-D	2		
		triazolam	1	1						
1640pa	58 y F				A	Ingst	Int-S	1		
		alprazolam	1	1					alprazolam	460 ng/mL In Blood (unspecified) @ Unknown
		ethanol	2	2					ethanol	12 mg/dL In Blood (unspecified) @ Unknown
1641h	59 y F				A	Ingst	Int-S	2		
		quetiapine	1	1						
		ethanol	2	2					ethanol	40 mg/dL In Serum @ Unknown
		lisinopril	3	3						
		atazanavir	4	4						
		emtricitabine/tenofovir	5	5						
		ritonavir	6	6						
		gabapentin	7	7						
1642a	59 y F				A	Ingst	Int-S	1		
		quetiapine	1	1					quetiapine	1.5 mg/L In Blood (unspecified) @ Autopsy
		bisoprodol *	2	2						
		ethanol *	3	2					ethanol	0.05 % (wt/Vol) In Blood (unspecified) @ Autopsy
[1643pha]	59 y F				A	Par	Int-S	1		
		pentobarbital/phenytoin	1	1					pentobarbital	4 mcg/mL In Blood (unspecified) @ Unknown
		pentobarbital/phenytoin	1	1					phenytoin	6 mcg/mL In Blood (unspecified) @ Unknown
		pentobarbital/phenytoin	1	1					pentobarbital	74.3 mcg/mL In Blood (unspecified) @ Autopsy
		embutramide/mebezonium/tetracaine	2	2						
1644a	61 y M				A/C	Ingst	Int-S	2		
		phenobarbital	1	1					phenobarbital	37 mg/L In Blood (unspecified) @ Unknown
		alprazolam	2	2						
1645h	61 y F				A	Ingst	Int-S	2		
		alprazolam	1	1						
1646ph	61 y M				A	Ingst	Int-U	2		
		benzodiazepine	1	1						
		antipsychotic (atypical)	2	2						
		benzodiazepine	3	3						
		lithium	4	4						
1647	61 y M				A/C	Ingst	Int-S	2		
		quetiapine	1	1						
		simvastatin	2	2						
		acetaminophen	3	3						
1648h	61 y M				A	Ingst	Int-S	1		
		benzodiazepine	1	1						
		lisinopril	2	2						
		hydrochlorothiazide	3	3						
1649ai	62 y F				U	Ingst	Int-S	2		
		diazepam	1	1						
1650h	62 y M				A	Ingst	Int-S	3		
		temazepam	1	1						
		lorazepam	2	2						
		primidone	3	3						
		lamotrigine	4	4						
		escitalopram	5	5						
		benztropine	6	6						
		antihyperlipidemic	7	7						
1651ph	64 y M				A	Ingst	Int-S	2		
		diazepam	1	1						
		temazepam	2	2						
		trazodone	3	3						
1652pa	68 y M				A/C	Ingst	Oth-M	2		
		risperidone	1	1						
		nitroglycerin	2	2						
1653ai	72 y M				A	Ingst	Int-S	2		
		zolpidem	1	1						
		diltiazem	2	2						
1654pa	74 y M				A	Ingst	Int-S	1		
		temazepam	1	1					temazepam	1512 ng/mL In Blood (unspecified) @ Autopsy
1655h	77 y F				A	Par	Unt-T	3		
		propofol	1	1						
1656pha	78 y F				A	Ingst	Int-S	2		
		benzodiazepine	1	1					nordiazepam	0.29 mg/L In Whole Blood @ Autopsy
		benzodiazepine	1	1					diazepam	0.32 mg/L In Whole Blood @ Autopsy
		temazepam	2	2					temazepam	0.02 mg/L In Whole Blood @ Autopsy
		meclizine	3	3						
		pregabalin	4	4						
		diphenhydramine	5	5						
1657h	80 y M				A	Ingst	Int-S	1		
		temazepam	1	1						
1658ha	81 y M				A	Ingst	Int-S	2		
		diazepam	1	1					nordiazepam	0.15 mg/L In Blood (unspecified) @ Unknown
		diazepam	1	1					diazepam	2.05 mg/L In Blood (unspecified) @ Unknown
1659	81 y F				A	Ingst	Int-S	1		
		lorazepam	1	1						
1660	81 y F				A/C	Ingst	Int-S	2		
		olanzepine	1	1						
		mirtazapine	2	2						
		zolpidem	3	3						
		warfarin	4	4						
1661ai	83 y F				A	Ingst	Int-S	2		
		zolpidem	1	1						
		sertraline	2	2						
		ethanol	3	3						
1662i	83 y F				A	Ingst	Unt-T	2		
		clonazepam	1	1						
1663ha	84 y M				A	Par	Unt-T	1		
		propofol	1	1						
1664	84 y F				A/C	Ingst	Int-S	3		
		lorazepam	1	1						
1665	Unknown adult (> = 20 yrs) F				A	Ingst	Int-S	2		
		clonazepam	1	1						
		diphenhydramine	2	2						
		meclizine	3	3						
		acetaminophen	4	4						
See Also case 14, 18, 20, 22, 32, 33, 39, 49, 50, 76, 105, 118, 122, 128, 142, 173, 178, 190, 243, 253, 282, 301, 307, 312, 347, 357, 358, 375, 393, 403, 407, 419, 420, 423, 428, 430, 431, 433, 437, 441, 444, 446, 449, 453, 458, 459, 461, 462, 466, 468, 473, 474, 477, 480, 483, 486, 487, 490, 491, 492, 496, 497, 498, 500, 508, 509, 511, 512, 513, 518, 520, 526, 527, 528, 532, 536, 544, 548, 549, 550, 553, 554, 555, 556, 557, 563, 565, 568, 571, 572, 573, 574, 575, 576, 577, 579, 582, 584, 588, 589, 590, 591, 592, 596, 597, 598, 602, 612, 615, 617, 618, 620, 621, 626, 627, 628, 629, 634, 635, 637, 638, 639, 642, 648, 649, 653, 654, 659, 661, 662, 664, 667, 671, 672, 673, 675, 677, 679, 680, 681, 685, 686, 687, 689, 693, 694, 697, 699, 704, 705, 706, 708, 710, 713, 715, 722, 725, 728, 730, 731, 734, 735, 745, 747, 748, 749, 750, 755, 759, 762, 763, 766, 774, 775, 777, 780, 783, 784, 785, 786, 787, 791, 793, 794, 796, 798, 800, 801, 802, 803, 805, 806, 811, 812, 813, 814, 823, 824, 828, 830, 833, 838, 840, 844, 847, 848, 852, 854, 855, 857, 862, 864, 865, 866, 868, 874, 875, 878, 880, 882, 883, 884, 888, 891, 897, 899, 901, 905, 910, 912, 916, 921, 923, 926, 929, 932, 933, 936, 938, 940, 946, 950, 951, 954, 969, 972, 975, 979, 983, 987, 988, 996, 998, 1000, 1001, 1005, 1023, 1025, 1026, 1071, 1086, 1091, 1122, 1124, 1125, 1130, 1131, 1132, 1133, 1134, 1137, 1139, 1145, 1146, 1147, 1148, 1152, 1153, 1154, 1155, 1157, 1162, 1165, 1177, 1178, 1180, 1181, 1182, 1185, 1190, 1194, 1195, 1201, 1202, 1203, 1204, 1207, 1210, 1211, 1213, 1218, 1219, 1226, 1227, 1229, 1232, 1240, 1241, 1242, 1245, 1246, 1248, 1249, 1251, 1255, 1256, 1261, 1262, 1264, 1265, 1267, 1269, 1270, 1274, 1279, 1281, 1282, 1291, 1294, 1295, 1298, 1303, 1313, 1314, 1317, 1326, 1336, 1342, 1343, 1350, 1351, 1354, 1355, 1363, 1365, 1366, 1369, 1374, 1375, 1377, 1387, 1395, 1400, 1403, 1404, 1408, 1409, 1410, 1412, 1413, 1416, 1421, 1422, 1426, 1436, 1437, 1438, 1440, 1444, 1448, 1455, 1459, 1466, 1478, 1494, 1510, 1514, 1519, 1527, 1530, 1531, 1537, 1540, 1541, 1544, 1545, 1547, 1550, 1551, 1552, 1553, 1559, 1562, 1706, 1712, 1718, 1725, 1727, 1732, 1736, 1740, 1742, 1744, 1745, 1751, 1756, 1760, 1761, 1765, 1766, 1767, 1770, 1779, 1784, 1792, 1799, 1810, 1811, 1814, 1818, 1830, 1831, 1834, 1838, 1840, 1842, 1843, 1844, 1846, 1855, 1863, 1864, 1869, 1871, 1873, 1900, 1901, 1911, 1918, 1920, 1926, 1941, 1942, 1950, 1952, 1957, 1965, 1967, 1970, 1971, 1973, 1975, 1976, 1989, 1991, 1994, 2007, 2008, 2015, 2019, 2021, 2022, 2032, 2044, 2046, 2047, 2050, 2059, 2074, 2091, 2100, 2110										
**Stimulants and Street Drugs**										
1666pa	14 y M				A	Ingst	Int-A	1		
		amphetamine (hallucinogenic), 2C	1	1						
1667ai	15 y F				U	Unk	Int-S	2		
		methamphetamine	1	1						
1668p	15 y M				A	Ingst	Int-A	2		
		amphetamine (hallucinogenic), 2C-E	1	1						
1669ha	16 y F				A	Ingst	Int-A	2		
		amphetamine	1	1						
1670a	17 y F				U	Unk	Int-A	1		
		amphetamine	1	1					amphetamine	120 ng/mL In Blood (unspecified) @ Unknown
		amphetamine	1	1					methamphetamine	3100 ng/mL In Blood (unspecified) @ Unknown
		methylenedioxymethamphetamine (MDMA) *	2	2					mda (3,4-methylenedioxyamphetamine)	10 ng/mL In Blood (unspecified) @ Unknown
		methylenedioxymethamphetamine (MDMA) *	2	2					mdma (3,4-methylenedioxymethamphetamine)	330 ng/mL In Blood (unspecified) @ Unknown
		THC homolog *	3	2						
[1671pa]	17 y M				A	Ingst	Int-A	1		
		amphetamine (hallucinogenic)	1	1					amphetamine	4100 ng/mL In Urine (quantitative only) @ Unknown
		amphetamine (hallucinogenic)	1	1					amphetamine	64 ng/mL In Blood (unspecified) @ Unknown
1672pa	17 y M				A/C	Par	Int-A	1		
		heroin	1	1						
		morphine	2	2						
1673p	17 y M				A	Inhal	Int-A	2		
		amphetamine (hallucinogenic), 2C-E	1	1						
1674p	17 y M				A	Unk	Int-A	2		
		4-acetoxy-N,N-dimethyltryptamine	1	1						
		marijuana	2	2						
1675ph	17 y M				U	Inhal+ Unk	Int-A	1		
		4-acetoxy-N,N-dimethyltryptamine	1	1						
		ethanol	2	2						
1676	17 y M				A	Ingst	Int-A	1		
		methylenedioxymethamphetamine (MDMA)	1	1						
1677	17 y F				A	Ingst	Int-S	1		
		amphetamine (hallucinogenic), 25i	1	1						
		lithium	2	2						
		cyclic antidepressant, unknown	3	3						
		marijuana	4	4						
1678a	17 y M				A	Ingst	Int-A	1		
		lysergic acid diethylamide (LSD)	1	1						
1679pha	17 y M				A	Inhal	Int-A	2		
		THC homolog, K2	1	1						
1680p	17 y M				A	Inhal	Int-A	3		
		THC homolog	1	1						
		ethanol	2	2					ethanol	33 mg/dL In Blood (unspecified) @ Autopsy
1681pi	18 y M				A	Oth	Int-A	2		
		heroin	1	1						
1682	18 y M				A	Ingst+ Unk	Int-A	2		
		cocaine	1	1						
		methamphetamine	2	2						
		lysergic acid diethylamide (LSD)	3	3						
		marijuana	4	4						
1683p	18 y M				A	Inhal	Int-A	2		
		THC homolog	1	1						
1684ai	19 y F				A	Ingst+ Par	Int-A	2		
		heroin	1	1						
		codeine	2	2						
		ethanol	3	3						
1685ai	19 y M				A	Ingst+ Unk	Int-A	2		
		heroin	1	1						
		hydroxyzine	2	2						
		diphenhydramine	3	3						
		paroxetine	4	4						
		codeine	5	5						
1686ha	19 y F				A	Ingst	Int-M	1		
		methamphetamine	1	1						
[1687a]	19 y F				A	Ingst	Int-A	1		
		methylenedioxymethamphetamine (MDMA)	1	1					midazolam	0.05 mg/L In Blood (unspecified) @ Autopsy
		methylenedioxymethamphetamine (MDMA)	1	1					mdma (3,4-methylenedioxymethamphetamine)	1.01 mg/L In Serum @ Unknown
		methylenedioxymethamphetamine (MDMA)	1	1					mdma (3,4-methylenedioxymethamphetamine)	1.18 mg/L In Vitreous @ Autopsy
		methylenedioxymethamphetamine (MDMA)	1	1					mdma (3,4-methylenedioxymethamphetamine)	1.36 mg/L In Blood (unspecified) @ Autopsy
		methylenedioxymethamphetamine (MDMA)	1	1					mdma (3,4-methylenedioxymethamphetamine)	1.72 mg/L In Blood (unspecified) @ 10 h (pe)
		methylenedioxymethamphetamine (MDMA)	1	1					mdma (3,4-methylenedioxymethamphetamine)	2.37 mg/kg In Liver @ Autopsy
		methylenedioxymethamphetamine (MDMA)	1	1					phenytoin	5.91 mg/L In Blood (unspecified) @ Autopsy
		methylenedioxymethamphetamine (MDMA)	1	1					mdma (3,4-methylenedioxymethamphetamine)	6.55 mg/kg In Brain @ Autopsy
1688pha	19 y M				A	Unk	Int-A	2		
		street drug	1	1						
1689	19 y F				A	Ingst	Int-A	2		
		amphetamine (hallucinogenic)	1	1						
1690ai	20 y F				A	Par	Int-A	2		
		heroin	1	1						
		codeine	2	2						
		quinine	3	3						
[1691a]	20 y M				A	Ingst	Int-A	1		
		amphetamine (hallucinogenic)	1	1					methylone	0.71 mg/L In Blood (unspecified) @ Autopsy
1692pa	20 y M				A	Ingst	Int-M	1		
		methamphetamine	1	1					amphetamine	0.28 mg/L In Blood (unspecified) @ Autopsy
		methamphetamine	1	1					methamphetamine	3.57 mg/L In Blood (unspecified) @ Autopsy
1693p	20 y F				U	Unk	Int-A	2		
		heroin	1	1						
1694	20 y F				U	Ingst	Int-A	1		
		methamphetamine	1	1						
1695	20 y F				A	Ingst	Int-A	1		
		amphetamine (hallucinogenic)	1	1						
1696ph	21 y M				A/C	Unk	Int-A	2		
		heroin	1	1						
1697ai	21 y F				A	Unk	Int-A	2		
		heroin	1	1						
		oxycodone	2	2						
		cocaine	3	3						
		amphetamine	4	4						
		promethazine	5	5						
		dextromethorphan	6	6						
		citalopram	7	7						
		fluoxetine	8	8						
1698pa	21 y F				A	Inhal	Int-A	1		
		amphetamine (hallucinogenic), 25i	1	1						
1699p	21 y F				A	Inhal+ Par	Int-A	2		
		heroin	1	1						
		cocaine	2	2						
1700ai	21 y M				A	Unk	Int-A	2		
		phencyclidine	1	1						
1701p	21 y M				A	Ingst+ Par	Int-A	1		
		heroin	1	1						
		ethanol	2	2					ethanol	107 mg/dL In Serum @ 1 h (pe)
		buprenorphine/naloxone (film)	3	3						
1702ph	21 y F				U	Unk	Int-A	1		
		heroin	1	1						
1703pa	21 y M				A/C	Unk	Int-A	1		
		methylone	1	1					methylone	0.029 mg/L In Blood (unspecified) @ Autopsy
1704ph	21 y M				A	Ingst+ Inhal	Int-M	2		
		heroin	1	1						
		methamphetamine	2	2						
1705ph	21 y M				A	Par	Int-A	2		
		heroin	1	1						
1706a	22 y F				U	Ingst	Unk	2		
		heroin	1	1					6-monoacetylmorphine	8.6 ng/mL In Blood (unspecified) @ Autopsy
		heroin	1	1					morphine	97.5 ng/mL In Blood (unspecified) @ Autopsy
		hydrocodone	2	2					hydrocodone	19.9 ng/mL In Blood (unspecified) @ Autopsy
		alprazolam	3	3					morphine	10000 ng/mL In Urine (quantitative only) @ Autopsy
		alprazolam	3	3					6-monoacetylmorphine	1236 ng/mL In Urine (quantitative only) @ Autopsy
		alprazolam	3	3					alpha-oh-alprazolam	2500 ng/mL In Urine (quantitative only) @ Autopsy
		alprazolam	3	3					hydrocodone	3943 ng/mL In Urine (quantitative only) @ Autopsy
		alprazolam	3	3					codeine	399 ng/mL In Urine (quantitative only) @ Autopsy
		alprazolam	3	3					alprazolam	651 ng/mL In Urine (quantitative only) @ Autopsy
		alprazolam	3	3					hydromorphone	925 ng/mL In Urine (quantitative only) @ Autopsy
1707ai	22 y M				U	Par	Int-A	2		
		heroin	1	1						
1708a	22 y F				A	Par	Oth-M	1		
		methamphetamine	1	1						
1709ai	22 y M				A	Unk	Int-A	2		
		heroin	1	1						
		oxycodone	2	2						
		trazodone	3	3						
		cocaine	4	4						
		bupropion	5	5						
		hydroxyzine	6	6						
		codeine	7	7						
		quinine	8	8						
1710ai	22 y M				A	Par	Int-A	2		
		heroin	1	1						
		quinine	2	2						
		codeine	3	3						
1711ai	22 y M				A	Par	Int-A	2		
		heroin	1	1						
		cocaine	2	2						
		codeine	3	3						
1712ai	22 y M				A	Unk	Int-A	2		
		heroin	1	1						
		diazepam	2	2						
1713p	22 y F				A/C	Par	Int-A	1		
		heroin	1	1						
1714p	22 y M				U	Unk	Int-S	2		
		cocaine	1	1					benzoylecognine	0.09 mg/kg In Brain @ Autopsy
		cocaine	1	1					benzoylecognine	0.11 mg/L In Blood (unspecified) @ Unknown
		cocaine	1	1					benzoylecognine	0.17 mg/L In Blood (unspecified) @ Autopsy
		opioid	2	2					morphine	0.05 mg/kg In Brain @ Autopsy
		opioid	2	2					morphine	0.05 mg/L In Blood (unspecified) @ Autopsy
1715ai	23 y F				A	Unk	Int-A	2		
		heroin	1	1						
		codeine	2	2						
1716ai	23 y M				A	Par	Int-A	2		
		heroin	1	1						
		diphenhydramine	2	2						
		codeine	3	3						
1717ai	23 y M				U	Unk	Int-A	2		
		heroin	1	1						
		codeine	2	2						
1718ai	23 y M				A	Par+ Unk	Int-A	2		
		heroin	1	1						
		alprazolam	2	2						
1719ai	23 y M				A	Ingst+ Unk	Int-A	2		
		heroin	1	1						
		ethanol	2	2						
1720ai	23 y M				U	Unk	Int-A	2		
		methamphetamine	1	1						
1721ai	23 y M				A	Ingst+ Par	Int-A	2		
		heroin	1	1						
		chlorpheniramine	2	2						
		dextromethorphan	3	3						
		ethanol	4	4						
1722ai	23 y F				A	Unk	Int-A	2		
		heroin	1	1						
		hydroxyzine	2	2						
		diphenhydramine	3	3						
		codeine	4	4						
1723ai	23 y M				A	Ingst+ Par	Int-A	2		
		heroin	1	1						
		codeine	2	2						
		ethanol	3	3						
[1724pa]	23 y M				U	Unk	Int-A	1		
		amphetamine (hallucinogenic)	1	1					methylone	0.22 mg/L In Blood (unspecified) @ Autopsy
		amphetamine (hallucinogenic)	1	1					mdma (3,4-methylenedioxymethamphetamine)	2.6 mg/L In Blood (unspecified) @ Autopsy
1725pha	23 y F				A	Ingst+ Inhal+ Par	Int-A	2		
		heroin	1	1						
		alprazolam	2	2						
		buprenorphine/naloxone (film)	3	3						
		fentanyl	4	4						
1726ai	24 y M				A	Unk	Int-A	2		
		heroin	1	1						
		methylone	2	2						
		amphetamine	3	3						
		methamphetamine	4	4						
		codeine	5	5						
1727ai	24 y F				A	Ingst+ Par	Int-A	2		
		heroin	1	1						
		alprazolam	2	2						
		diphenhydramine	3	3						
		acetaminophen	4	4						
1728ai	24 y M				U	Unk	Int-A	2		
		methamphetamine	1	1						
		ethanol	2	2						
1729ai	24 y M				U	Ingst+ Unk	Int-A	2		
		heroin	1	1						
		oxycodone	2	2						
1730ai	24 y M				U	Unk	Int-A	2		
		methamphetamine	1	1						
1731ai	24 y F				A	Par	Int-A	2		
		heroin	1	1						
1732ai	24 y M				A	Unk	Int-A	2		
		heroin	1	1						
		methadone	2	2						
		benzodiazepine	3	3						
1733	24 y F				A	Unk	Int-A	3		
		heroin	1	1						
1734ai	24 y M				A	Ingst+ Unk	Int-A	2		
		heroin	1	1						
		oxycodone	2	2						
		diphenhydramine	3	3						
		acetaminophen	4	4						
		ethanol	5	5						
1735ph	24 y M				A	Ingst	Int-A	1		
		amphetamine (hallucinogenic)	1	1						
		methylenedioxymethamphetamine (MDMA)	2	2						
		ethanol	3	3					ethanol	66 mg/dL In Urine (quantitative only) @ Unknown
1736pa	25 y M				U	Unk	Int-A	1		
		heroin	1	1					morphine	0.027 mg/L In Blood (unspecified) @ Autopsy
		heroin	1	1					codeine	0.26 mg/L In Urine (quantitative only) @ Autopsy
		heroin	1	1					6-monoacetylmorphine	0.57 mg/L In Urine (quantitative only) @ Autopsy
		heroin	1	1					morphine	2.2 mg/L In Urine (quantitative only) @ Autopsy
		clonazepam	2	2					7-aminoclonazepam	0.1 mg/L In Blood (unspecified) @ Autopsy
		alprazolam	3	3					alprazolam	0.026 mg/L In Blood (unspecified) @ Autopsy
		cocaine	4	4					benzoylecognine	0.39 mg/L In Blood (unspecified) @ Autopsy
1737pha	25 y M				A	Ingst+ Par	Int-A	2		
		heroin	1	1					morphine (free)	0.042 mg/L In Blood (unspecified) @ Autopsy
		heroin	1	1					codeine	0.117 mg/L In Urine (quantitative only) @ Autopsy
		heroin	1	1					6-monoacetylmorphine	0.501 mg/L In Urine (quantitative only) @ Autopsy
		heroin	1	1					morphine (free)	1.992 mg/L In Urine (quantitative only) @ Autopsy
		naloxone	2	2						
		ethanol	3	3					ethanol	0.049 mg/L In Blood (unspecified) @ Unknown
1738ai	25 y M				A	Unk	Int-A	2		
		heroin	1	1						
1739ai	25 y M				A	Unk	Int-A	2		
		heroin	1	1						
		bupropion	2	2						
		codeine	3	3						
1740pa	25 y M				A	Ingst	Int-A	1		
		heroin	1	1					morphine (free)	21 ng/mL In Blood (unspecified) @ Unknown
		benzodiazepine	2	2					alprazolam	39 ng/mL In Blood (unspecified) @ Unknown
		methadone	3	3						
1741p	25 y F				U	Par	Int-A	2		
		methamphetamine	1	1						
1742ai	25 y F				A	Unk	Int-A	2		
		heroin	1	1						
		alprazolam	2	2						
		diphenhydramine	3	3						
		citalopram	4	4						
1743ai	25 y F				A	Ingst+ Inhal	Int-A	2		
		heroin	1	1						
		citalopram	2	2						
		quinine	3	3						
1744ai	26 y M				A	Unk	Int-A	2		
		heroin	1	1						
		cocaine	2	2						
		clonazepam	3	3						
		quinine	4	4						
		codeine	5	5						
		diltiazem	6	6						
		lidocaine	7	7						
1745ai	26 y M				A	Unk	Int-A	2		
		heroin	1	1						
		alprazolam	2	2						
		codeine	3	3						
1746ai	26 y F				U	Unk	Int-A	2		
		methamphetamine	1	1						
1747ai	26 y F				A	Ingst+ Par	Int-A	2		
		heroin	1	1						
		ethanol	2	2						
1748ai	26 y M				A	Par	Int-A	2		
		heroin	1	1						
1749ai	26 y F				A	Par	Int-A	2		
		heroin	1	1						
1750ai	26 y M				U	Ingst	Int-A	2		
		phentermine	1	1						
		acetaminophen/hydrocodone	2	2						
		oxycodone	3	3						
1751ai	26 y M				A	Inhal+ Unk	Int-A	2		
		heroin	1	1						
		cocaine	2	2						
		methadone	3	3						
		clonazepam	4	4						
		alprazolam	5	5						
		diphenhydramine	6	6						
		chlorpheniramine	7	7						
		quinine	8	8						
		codeine	9	9						
1752ai	26 y F				A	Unk	Int-A	2		
		heroin	1	1						
		cocaine	2	2						
		diphenhydramine	3	3						
		diltiazem	4	4						
		quinine	5	5						
		lidocaine	6	6						
1753ai	26 y M				A	Par	Int-A	2		
		heroin	1	1						
		codeine	2	2						
1754ai	26 y F				U	Unk	Int-A	2		
		heroin	1	1						
		codeine	2	2						
		acetaminophen/hydrocodone	3	3						
		oxycodone	4	4						
		oxymorphone	5	5						
1755ai	26 y M				U	Par+ Unk	Int-A	2		
		heroin	1	1						
		morphine	2	2						
1756ai	26 y M				A	Ingst+ Par	Int-A	2		
		heroin	1	1						
		alprazolam	2	2						
		quinine	3	3						
		codeine	4	4						
1757ph	26 y M				A	Unk	Int-A	2		
		amphetamine	1	1						
		antitussives-expectorants	2	2						
1758ph	26 y M				A	Ingst+ Unk	Int-A	2		
		gamma-hydroxybutyric acid	1	1						
		vasodilator, unknown	2	2						
		amphetamine *	3	3						
		ibuprofen *	4	3						
		vasodilator, unknown	5	4						
1759p	27 y M				U	Unk	Int-A	2		
		heroin	1	1						
		cocaine	2	2						
1760pa	27 y M				A	Ingst	Int-A	1		
		heroin	1	1					morphine (free)	25 mcg/L In Blood (unspecified) @ Autopsy
		clonazepam	2	2						
		marijuana	3	3						
1761ai	27 y M				A	Par+ Unk	Int-A	2		
		heroin	1	1						
		clonazepam	2	2						
1762ai	27 y M				A	Unk	Int-A	2		
		heroin	1	1						
		cocaine	2	2						
		ethanol (non-beverage)	3	3						
		codeine	4	4						
1763ai	27 y M				A	Par	Int-A	2		
		heroin	1	1						
		codeine	2	2						
1764ai	27 y M				A	Unk	Int-A	2		
		heroin	1	1						
		ethanol	2	2						
1765ai	27 y M				A	Par+ Unk	Int-A	2		
		heroin	1	1						
		hydrocodone	2	2						
		diazepam	3	3						
		trazodone	4	4						
		acetaminophen	5	5						
		clonazepam	6	6						
		quinine	7	7						
		codeine	8	8						
1766pa	27 y M				U	Unk	Int-A	2		
		heroin	1	1					morphine	0.16 mcg/mL In Whole Blood @ Autopsy
		oxycodone	2	2					oxycodone (total)	0.31 mcg/mL In Whole Blood @ Autopsy
		methadone	3	3					methadone	0.076 mcg/mL In Whole Blood @ Autopsy
		clonazepam	4	4						
		zolpidem	5	5					zolpidem	0.15 mcg/mL In Whole Blood @ Autopsy
		drug, unknown	6	6						
1767p	27 y F				A	Ingst+ Par	Int-A	1		
		heroin	1	1						
		quetiapine	2	2						
1768pha	27 y M				A	Ingst	Int-A	1		
		amphetamine (hallucinogenic) *	2	1					amphetamine	0.05 mg/L In Blood (unspecified) @ Unknown
		amphetamine (hallucinogenic) *	2	1					amphetamine	0.18 mg/L In Blood (unspecified) @ Autopsy
		amphetamine (hallucinogenic) *	2	1					methamphetamine	0.49 mg/L In Blood (unspecified) @ Unknown
		amphetamine (hallucinogenic) *	2	1					methamphetamine	0.86 mg/L In Blood (unspecified) @ Autopsy
		methamphetamine *	1	1					amphetamine	0.05 mg/L In Blood (unspecified) @ Unknown
		methamphetamine *	1	1					amphetamine	0.18 mg/L In Blood (unspecified) @ Autopsy
		methamphetamine *	1	1					methamphetamine	0.49 mg/L In Blood (unspecified) @ Unknown
		methamphetamine *	1	1					methamphetamine	0.86 mg/L In Blood (unspecified) @ Autopsy
1769pa	27 y M				A	Par	Int-A	1		
		heroin	1	1					morphine	160 ng/mL In Blood (unspecified) @ Autopsy
		heroin	1	1					6-monoacetylmorphine	450 ng/mL In Urine (quantitative only) @ Autopsy
		paroxetine	2	2						
		trazodone	3	3						
1770ai	27 y M				U	Ingst+ Aspir+ Unk	Int-A	2		
		methamphetamine	1	1						
		tramadol	2	2						
		diazepam	3	3						
		amitriptyline	4	4						
1771	27 y M				U	Unk	Int-A	3		
		cocaine *	1	1						
		drug, unknown *	2	1						
1772	27 y M				A	Unk	Unk	3		
		cocaine	1	1						
1773p	27 y M				A	Par	Int-A	2		
		heroin	1	1						
1774p	27 y M				U	Par	Int-A	2		
		heroin	1	1						
1775a	27 y M				A	Unk	Int-A	1		
		amphetamine (hallucinogenic), 2C-I	1	1						
		ketamine	2	2						
		hydromorphone	3	3						
1776	27 y M				A	Ingst+ Inhal	Int-A	2		
		THC homolog	1	1						
		marijuana	2	2					carboxy-thc	176 ng/mL In Blood (unspecified) @ Unknown
		marijuana	2	2					carboxy-thc	246 ng/mL In Blood (unspecified) @ Unknown
		acetaminophen	3	3						
1777ph	28 y M				A/C	Unk	Int-A	1		
		heroin	1	1						
1778	28 y M				U	Unk	Unk	2		
		methamphetamine	1	1						
1779ai	28 y M				A	Ingst+ Par	Int-A	2		
		heroin	1	1						
		alprazolam	2	2						
		diphenhydramine	3	3						
		methadone	4	4						
		oxycodone	5	5						
		quinine	6	6						
		codeine	7	7						
1780pa	28 y F				A	Ingst+ Par	Int-S	1		
		heroin	1	1						
		drug, unknown	2	2						
1781ai	28 y F				A	Par	Int-A	2		
		heroin	1	1						
1782pa	28 y F				A/C	Par	Int-A	1		
		heroin	1	1					morphine (free)	0.14 mg/L In Blood (unspecified) @ 2 m (pe)
		cocaine	2	2					benzoylecognine	1 mg/L In Blood (unspecified) @ 2 m (pe)
[1783pha]	28 y F				U	Unk	Int-A	2		
		cocaine	1	1					benzoylecognine	3300 ng/mL In Blood (unspecified) @ Autopsy
		levamisole	2	2						
1784ai	28 y M				A	Ingst+ Par	Int-A	2		
		heroin	1	1						
		methadone	2	2						
		oxycodone	3	3						
		alprazolam	4	4						
		acetaminophen	5	5						
		quinine	6	6						
1785i	28 y F				A/C	Unk	Int-A	1		
		amphetamine (hallucinogenic)	1	1						
1786ai	28 y F				U	Unk	Int-A	2		
		methamphetamine	1	1						
1787ai	29 y M				A	Unk	Int-A	2		
		heroin	1	1						
		codeine	2	2						
1788ai	29 y M				A	Unk	Int-A	2		
		heroin	1	1						
		cocaine	2	2						
		sertraline	3	3						
		diphenhydramine	4	4						
		benztropine	5	5						
		hydroxyzine	6	6						
		codeine	7	7						
		quinine	8	8						
1789pha	29 y M				A	Unk	Int-A	1		
		heroin	1	1						
		ethanol	2	2					ethanol	166 mg/dL In Blood (unspecified) @ 10 m (pe)
1790ai	29 y F				A	Unk	Int-A	2		
		heroin	1	1						
1791p	29 y M				U	Ingst+ Inhal	Int-A	2		
		phencyclidine	1	1						
		ethanol	2	2					ethanol	124 mg/dL In Blood (unspecified) @ Unknown
		acetaminophen/oxycodone	3	3						
		marijuana	4	4						
		drug, unknown	5	5						
1792ai	29 y M				A	Ingst+ Par	Int-A	2		
		heroin	1	1						
		sertraline	2	2						
		chlorpromazine	3	3						
		trazodone	4	4						
		ethanol	5	5						
1793ai	29 y M				A	Ingst+ Par	Int-A	2		
		heroin	1	1						
		oxycodone	2	2						
		diphenhydramine	3	3						
		quinine	4	4						
		codeine	5	5						
1794	29 y F				U	Unk	Unk	2		
		methamphetamine	1	1						
1795ai	29 y M				A	Ingst+ Par	Int-A	2		
		heroin	1	1						
		ethanol	2	2						
1796	29 y F				A	Ingst	Int-M	1		
		methamphetamine	1	1						
1797ai	29 y M				A	Ingst+ Par	Int-A	2		
		heroin	1	1						
		diphenhydramine	2	2						
		dextromethorphan	3	3						
		quinine	4	4						
		codeine	5	5						
1798ai	29 y M				A	Unk	Int-A	2		
		heroin	1	1						
		dextromethorphan	2	2						
		codeine	3	3						
1799ai	29 y F				U	Ingst+ Unk	Int-A	2		
		methamphetamine	1	1						
		venlafaxine	2	2						
		acetaminophen/hydrocodone	3	3						
		quetiapine	4	4						
1800pha	29 y F				A/C	Par	Int-A	1		
		heroin	1	1					morphine (free)	220 ng/mL In Whole Blood @ Autopsy
		heroin	1	1					6-monoacetylmorphine	890 ng/mL In Urine (quantitative only) @ Autopsy
		cocaine	2	2						
		ethanol	3	3						
1801ai	30 y M				A	Unk	Int-A	2		
		heroin	1	1						
		methadone	2	2						
		cocaine	3	3						
		hydrocodone	4	4						
		oxycodone	5	5						
		doxylamine	6	6						
		diphenhydramine	7	7						
		citalopram	8	8						
		quinine	9	9						
		acetaminophen	10	10						
1802ph	30 y M				A	Unk	Int-A	3		
		heroin	1	1						
		cyanide	2	2						
1803ai	30 y M				U	Unk	Int-A	2		
		cocaine	1	1						
		methamphetamine	2	2						
		amphetamine	3	3						
1804ai	30 y M				U	Ingst+ Aspir+ Unk	Int-A	2		
		methamphetamine	1	1						
		acetaminophen/hydrocodone	2	2						
1805pai	30 y M				U	Ingst+ Unk	Unt-M	2		
		heroin	1	1					6-monoacetylmorphine	1 Other (see abst) In Urine (quantitative only) @ Autopsy
		heroin	1	1					codeine	1 Other (see abst) In Urine (quantitative only) @ Autopsy
		heroin	1	1					morphine	1 Other (see abst) In Urine (quantitative only) @ Autopsy
		heroin	1	1					morphine	93 ng/mL In Whole Blood @ Autopsy
		ethanol	2	2					ethanol	0.13 % (wt/Vol) In Blood (unspecified) @ Autopsy
		ethanol	2	2					ethanol	0.14 % (wt/Vol) In Vitreous @ Autopsy
		diphenhydramine	3	3					diphenhydramine	1 Other (see abst) In Blood (unspecified) @ Autopsy
		caffeine	4	4					caffeine	1 Other (see abst) In Blood (unspecified) @ Autopsy
		caffeine	4	4					caffeine	1 Other (see abst) In Urine (quantitative only) @ Autopsy
		caffeine	4	4					theobromine	1 Other (see abst) In Urine (quantitative only) @ Autopsy
1806pai	30 y M				U	Ingst+ Unk	Int-A	2		
		heroin	1	1					codeine	1 Other (see abst) In Blood (unspecified) @ Autopsy
		heroin	1	1					morphine	1 Other (see abst) In Urine (quantitative only) @ Autopsy
		heroin	1	1					6-monoacetylmorphine	1 Other (see abst) In Urine (quantitative only) @ Autopsy
		heroin	1	1					codeine	1 Other (see abst) In Urine (quantitative only) @ Autopsy
		heroin	1	1					6-monoacetylmorphine	11 ng/mL In Blood (unspecified) @ Autopsy
		heroin	1	1					morphine	184 ng/mL In Blood (unspecified) @ Autopsy
		ethanol	2	2					ethanol	0.17 % (wt/Vol) In Blood (unspecified) @ Autopsy
		ethanol	2	2					ethanol	0.18 % (wt/Vol) In Vitreous @ Autopsy
		diphenhydramine	3	3					diphenhydramine	1 Other (see abst) In Blood (unspecified) @ Autopsy
		caffeine *	5	4					caffeine	1 Other (see abst) In Blood (unspecified) @ Autopsy
		caffeine *	5	4					caffeine	1 Other (see abst) In Urine (quantitative only) @ Autopsy
		hydrocodone *	4	4					hydrocodone	1 Other (see abst) In Urine (quantitative only) @ Autopsy
		hydrocodone *	4	4					dihydrocodeine	1 Other (see abst) In Urine (quantitative only) @ Autopsy
		hydrocodone *	4	4					hydrocodone	11 ng/mL In Blood (unspecified) @ Autopsy
		nicotine	6	6						
1807ai	30 y M				A	Unk	Int-A	2		
		heroin	1	1						
		diphenhydramine	2	2						
		dextromethorphan	3	3						
		codeine	4	4						
1808ai	30 y F				A	Ingst+ Unk	Int-A	2		
		heroin	1	1						
		bupropion	2	2						
		ethanol	3	3						
1809ai	30 y F				U	Ingst+ Inhal	Int-A	2		
		phencyclidine	1	1						
		acetaminophen/hydrocodone	2	2						
		oxycodone	3	3						
1810ai	31 y F				A	Ingst+ Unk	Int-A	2		
		heroin	1	1						
		oxycodone	2	2						
		cocaine	3	3						
		clonazepam	4	4						
		sertraline	5	5						
		ethanol (non-beverage)	6	6						
1811ai	31 y M				A	Unk	Int-A	2		
		heroin	1	1						
		hydrocodone	2	2						
		alprazolam	3	3						
		codeine	4	4						
		quinine	5	5						
		diltiazem	6	6						
1812ai	31 y M				A	Par	Int-A	2		
		heroin	1	1						
		codeine	2	2						
1813p	31 y M				A	Par	Int-A	1		
		heroin	1	1						
1814ai	31 y M				A	Ingst+ Par	Int-A	2		
		heroin	1	1						
		cocaine	2	2						
		citalopram	3	3						
		alprazolam	4	4						
		quinine	5	5						
1815p	31 y M				A/C	Par	Int-A	1		
		heroin	1	1						
		ethanol	2	2					ethanol	327 mg/dL In Blood (unspecified) @ 30 m (pe)
1816ai	31 y M				U	Par	Int-A	2		
		methamphetamine	1	1						
1817ai	31 y M				A	Ingst+ Unk	Int-A	2		
		heroin	1	1						
		cocaine	2	2						
		ethanol	3	3						
1818ai	31 y M				A	Unk	Int-A	2		
		heroin	1	1						
		methadone	2	2						
		tramadol	3	3						
		clonazepam	4	4						
		lamotrigine	5	5						
		fluoxetine	6	6						
		amphetamine	7	7						
		diphenhydramine	8	8						
		quinine	9	9						
		codeine	10	10						
1819ai	31 y M				A	Ingst+ Par	Int-M	2		
		heroin	1	1						
		ethanol	2	2						
1820ai	31 y M				A	Unk	Int-A	2		
		heroin	1	1						
		cocaine	2	2						
		methylone	3	3						
		codeine	4	4						
1821h	31 y F				U	Par	Unk	3		
		heroin	1	1						
		cocaine	2	2						
1822	31 y F				U	Ingst	Unk	3		
		cocaine	1	1						
		opioid	2	2						
1823ai	32 y M				U	Unk	Oth-M	2		
		methamphetamine	1	1						
		amphetamine	2	2						
1824ai	32 y M				A	Par	Int-A	2		
		heroin	1	1						
1825ai	32 y F				A	Par+ Unk	Int-A	2		
		heroin	1	1						
		methadone	2	2						
		cocaine	3	3						
1826ai	32 y M				A	Par	Int-A	2		
		heroin	1	1						
		diphenhydramine	2	2						
1827ai	32 y M				A	Ingst+ Par	Int-A	2		
		heroin	1	1						
		ethanol	2	2						
1828pa	32 y F				A	Par	Int-S	1		
		heroin	1	1					morphine	77 mcg/L In Blood (unspecified) @ Autopsy
		cocaine	2	2					benzoylecognine	2.4 mg/L In Blood (unspecified) @ Autopsy
1829ai	32 y F				A	Unk	Int-A	2		
		cocaine	1	1						
		heroin	2	2						
1830ai	32 y M				A	Unk	Int-A	2		
		heroin	1	1						
		oxycodone	2	2						
		alprazolam	3	3						
		diphenhydramine	4	4						
		codeine	5	5						
1831ha	32 y F				U	Unk	Unk	1		
		cocaine	1	1					cocaine	0.1 mg/L In Blood (unspecified) @ Unknown
		cocaine	1	1					benzoylecognine	2.92 mg/L In Blood (unspecified) @ Unknown
		methadone	2	2						
		fentanyl	3	3					norfentanyl	3.9 ng/mL In Whole Blood @ Autopsy
		fentanyl	3	3					fentanyl	46 ng/mL In Whole Blood @ Autopsy
		midazolam	4	4					midazolam	220 ng/mL In Whole Blood @ Autopsy
		morphine	5	5					morphine (free)	130 ng/mL In Whole Blood @ Autopsy
1832pa	32 y F				A	Par	Int-A	2		
		cocaine	1	1					benzoylecognine	0.2 mg/L In Blood (unspecified) @ Autopsy
		heroin	2	2					morphine (free)	20 mcg/L In Blood (unspecified) @ Autopsy
		fluoxetine	3	3					fluoxetine	0.6 mg/L In Blood (unspecified) @ Autopsy
		citalopram	4	4					citalopram	0.1 mg/L In Blood (unspecified) @ Autopsy
		dextromethorphan	5	5					dextromethorphan	0.07 mg/L In Blood (unspecified) @ Autopsy
		hydroxyzine	6	6					hydroxyzine	0.06 mg/L In Blood (unspecified) @ Autopsy
		quinine	7	7						
1833	32 y M				U	Ingst	Int-U	2		
		cocaine	1	1						
		amitriptyline	2	2						
1834pha	32 y M				U	Ingst	Int-A	1		
		cocaine	1	1					benzoylecognine	280 ng/mL In Blood (unspecified) @ Autopsy
		benzodiazepine	2	2						
		opioid	3	3						
1835	32 y F				A	Ingst	Int-S	3		
		phencyclidine	1	1						
		trazodone	2	2						
		fluoxetine	3	3						
		carbamazepine	4	4						
[1836ha]	32 y M				A	Ingst	Int-M	1		
		methamphetamine	1	1					amphetamine	24317.5 ng/mL In Urine (quantitative only) @ Autopsy
1837a	32 y M				C	Ingst	Int-M	1		
		methamphetamine	1	1						
1838ai	33 y F				U	Ingst+ Unk	Int-A	2		
		methamphetamine	1	1						
		morphine	2	2						
		diazepam	3	3						
1839	33 y M				U	Unk	Unk	1		
		methamphetamine	1	1						
		marijuana	2	2						
1840ai	33 y M				U	Ingst+ Unk	Int-A	2		
		cocaine	1	1						
		droperidol/fentanyl	2	2						
		diazepam	3	3						
1841ai	33 y M				A	Unk	Int-A	2		
		heroin	1	1						
		carbamazepine	2	2						
		quinine	3	3						
		codeine	4	4						
1842ai	33 y M				A	Unk	Int-A	2		
		heroin	1	1						
		cocaine	2	2						
		3,4-methylenedioxyamphetamine (MDA)	3	3						
		alprazolam	4	4						
1843ai	33 y F				A	Ingst+ Par	Int-A	2		
		heroin	1	1						
		cocaine	2	2						
		citalopram	3	3						
		oxymorphone	4	4						
		alprazolam	5	5						
		codeine	6	6						
1844ai	33 y F				A	Par+ Unk	Int-A	2		
		heroin	1	1						
		methadone	2	2						
		cocaine	3	3						
		alprazolam	4	4						
1845p	33 y M				A	Ingst+ Inhal	Int-A	2		
		cocaine	1	1					benzoylecognine	3.4 mg/L In Blood (unspecified) @ Autopsy
		acetaminophen/oxycodone	2	2					oxycodone	0.25 mg/L In Blood (unspecified) @ Autopsy
		acetaminophen/oxycodone	2	2					acetaminophen	16.9 mg/L In Blood (unspecified) @ Autopsy
1846pha	33 y F				A	Ingst	Unk	3		
		heroin	1	1						
		alprazolam	2	2						
		methadone	3	3					methadone metabolite	0.054 mg/L In Blood (unspecified) @ Autopsy
		methadone	3	3					methadone	0.545 mg/L In Blood (unspecified) @ Autopsy
		citalopram	4	4					citalopram	0.044 mg/L In Blood (unspecified) @ Autopsy
		beta blocker	5	5					propranolol	0.118 mg/L In Blood (unspecified) @ Autopsy
1847p	34 y F				A	Ingst+ Unk	Unk	1		
		heroin	1	1						
		nortriptyline	2	2						
		cyclobenzaprine	3	3						
1848ai	34 y M				A	Unk	Int-A	2		
		heroin	1	1						
		ethanol	2	2						
1849h	34 y F				A/C	Ingst+ Inhal+ Unk	Int-A	1		
		amphetamine (hallucinogenic)	1	1						
		ethanol	2	2						
		heroin	3	3						
1850ai	34 y M				A	Ingst+ Par	Int-A	2		
		heroin	1	1						
		oxycodone	2	2						
		cocaine	3	3						
		citalopram	4	4						
		diphenhydramine	5	5						
		quinine	6	6						
1851ai	34 y M				A	Unk	Int-A	2		
		heroin	1	1						
		codeine	2	2						
1852ai	34 y M				A	Ingst+ Unk	Int-A	2		
		heroin	1	1						
		ethanol	2	2						
		amphetamine	3	3						
1853ai	34 y M				A	Inhal	Int-A	2		
		THC homolog, XLR-11	1	1						
		THC homolog, UR-144	2	2						
1854	34 y M				A	Ingst	Int-A	1		
		phencyclidine	1	1					phencyclidine	0.04 mg/L In Blood (unspecified) @ Autopsy
		ethanol	2	2						
1855ai	34 y F				A	Ingst+ Par	Int-A	2		
		heroin	1	1						
		quetiapine	2	2						
		carbamazepine	3	3						
		quinine	4	4						
1856ai	34 y F				A	Unk	Int-A	2		
		cocaine	1	1						
		methadone	2	2						
1857ai	34 y M				A	Unk	Int-A	2		
		heroin	1	1						
		phencyclidine	2	2						
1858p	34 y M				A	Unk	Int-A	2		
		heroin	1	1					morphine (free)	120 ng/mL In Blood (unspecified) @ Autopsy
		ethanol	2	2					ethanol	174 mg/dL In Blood (unspecified) @ Autopsy
1859ai	35 y F				A	Unk	Int-A	2		
		heroin	1	1						
		cocaine	2	2						
		sertraline	3	3						
		acetaminophen	4	4						
1860ai	35 y M				U	Unk	Int-A	2		
		methamphetamine	1	1						
1861ai	35 y M				A	Par	Int-A	2		
		heroin	1	1						
		quinine	2	2						
1862ph	35 y F				A/C	Inhal+ Par	Int-A	2		
		heroin	1	1						
		cocaine	2	2						
1863ai	35 y M				A	Ingst+ Unk	Int-A	2		
		heroin	1	1						
		methadone	2	2						
		alprazolam	3	3						
1864pa	35 y F				A	Ingst	Int-U	2		
		cocaine	1	1						
		alprazolam	2	2						
		carisoprodol	3	3						
1865ai	36 y M				A	Ingst+ Unk	Int-A	2		
		heroin	1	1						
		diphenhydramine	2	2						
		codeine	3	3						
		ethanol	4	4						
1866ai	36 y M				A	Par	Int-A	2		
		heroin	1	1						
		quinine	2	2						
1867p	36 y M				A	Inhal	Unk	2		
		cocaine	1	1						
		bupropion	2	2						
1868p	36 y M				U	Par	Int-A	1		
		heroin	1	1						
		cocaine	2	2						
1869h	37 y F				A	Ingst	Int-S	3		
		amphetamine	1	1						
		alprazolam	2	2						
		opioid	3	3						
1870ai	37 y F				U	Ingst+ Unk	Int-A	2		
		methamphetamine	1	1						
		ethanol	2	2						
1871ai	37 y M				A	Ingst+ Par	Int-A	2		
		heroin	1	1						
		oxycodone	2	2						
		tramadol	3	3						
		clonazepam	4	4						
		codeine	5	5						
1872ai	37 y F				A	Ingst+ Unk	Int-A	2		
		heroin	1	1						
		amitriptyline	2	2						
1873p	37 y M				U	Ingst+ Unk	Unk	2		
		heroin	1	1						
		alprazolam	2	2						
1874ai	38 y M				A	Unk	Int-A	2		
		heroin	1	1						
		cocaine	2	2						
		citalopram	3	3						
		quinine	4	4						
1875ai	38 y F				A	Ingst+ Par	Int-A	2		
		heroin	1	1						
		cocaine	2	2						
		sertraline	3	3						
		doxylamine	4	4						
		diphenhydramine	5	5						
		quinine	6	6						
		codeine	7	7						
1876pha	38 y F				A	Ingst	Int-A	2		
		amphetamine (hallucinogenic) *	2	1						
		bupropion *	1	1					bupropion	9400 ng/mL In Whole Blood @ Autopsy
		propranolol	3	3						
1877h	38 y F				U	Ingst	Int-S	1		
		caffeine	1	1						
		diphenhydramine	2	2						
1878ai	38 y M				A	Unk	Int-A	2		
		heroin	1	1						
		diphenhydramine	2	2						
		codeine	3	3						
1879ai	38 y M				A	Ingst+ Par	Int-A	2		
		heroin	1	1						
		methadone	2	2						
		trazodone	3	3						
		oxycodone	4	4						
		quinine	5	5						
		codeine	6	6						
1880ai	38 y M				A	Par	Int-A	2		
		heroin	1	1						
		quinine	2	2						
1881ai	39 y M				A	Par	Int-A	2		
		heroin	1	1						
		cocaine	2	2						
		diphenhydramine	3	3						
		codeine	4	4						
		quinine	5	5						
1882ai	39 y M				U	Ingst	Int-A	2		
		phentermine	1	1						
		morphine	2	2						
1883ai	39 y F				A	Ingst+ Par	Int-A	2		
		heroin	1	1						
		trazodone	2	2						
		citalopram	3	3						
		bupropion	4	4						
		cocaine	5	5						
		ethanol	6	6						
1884ai	39 y M				U	Inhal	Int-A	2		
		phencyclidine	1	1						
1885ai	39 y M				A	Unk	Int-A	2		
		heroin	1	1						
		cocaine	2	2						
		dextromethorphan	3	3						
		quinine	4	4						
1886pa	39 y M				A	Inhal	Int-A	2		
		methamphetamine	1	1						
		marijuana	2	2						
1887h	40 y M				A/C	Ingst	Unk	2		
		gamma-hydroxybutyric acid	1	1						
		cadmium	2	2					cadmium	61.6 mcg/L In Urine (quantitative only) @ Unknown
1888ai	40 y M				A	Unk	Int-A	2		
		heroin	1	1						
		cocaine	2	2						
		quinine	3	3						
1889ai	40 y M				A	Unk	Int-A	2		
		heroin	1	1						
1890ai	40 y F				U	Unk	Int-A	2		
		cocaine	1	1						
1891ai	40 y M				U	Unk	Int-A	2		
		heroin	1	1						
1892ai	40 y M				A	Ingst+ Unk	Int-A	2		
		heroin	1	1						
		cocaine	2	2						
		oxycodone	3	3						
		citalopram	4	4						
		hydrocodone	5	5						
		acetaminophen	6	6						
		ethanol	7	7						
1893ai	40 y M				A	Unk	Int-A	2		
		heroin	1	1						
		cocaine	2	2						
		codeine	3	3						
1894ai	40 y F				A	Ingst+ Unk	Int-A	2		
		heroin	1	1						
		cocaine	2	2						
		oxycodone	3	3						
		acetaminophen	4	4						
		diphenhydramine	5	5						
1895ai	40 y M				A	Ingst+ Unk	Int-A	2		
		heroin	1	1						
		doxylamine	2	2						
		citalopram	3	3						
		ethanol	4	4						
1896ai	40 y F				U	Unk	Int-A	2		
		methamphetamine	1	1						
1897p	40 y M				A	Ingst+ Unk	Int-A	1		
		heroin	1	1						
		ethanol	2	2						
		bite (rodent)	3	3						
1898pa	40 y M				U	Ingst+ Inhal+ Aspir+ Unk	Int-A	2		
		cocaine	1	1					benzoylecognine	0.12 mg/L In Blood (unspecified) @ Unknown
		opioid	2	2						
		phencyclidine	3	3						
		ethanol	4	4					ethanol	123 mg/dL In Blood (unspecified) @ Unknown
1899ai	41 y M				A	Ingst+ Par	Int-A	2		
		cocaine	1	1						
		heroin	2	2						
		amitriptyline	3	3						
		bupropion	4	4						
1900ai	41 y M				A	Unk	Int-U	2		
		cocaine	1	1						
		diazepam	2	2						
		clonazepam	3	3						
		alprazolam	4	4						
		citalopram	5	5						
		acetaminophen	6	6						
1901ai	41 y M				U	Ingst+ Unk	Int-A	2		
		methamphetamine	1	1						
		alprazolam	2	2						
		ethanol	3	3						
1902ai	41 y M				A	Par+ Unk	Int-A	2		
		heroin	1	1						
		cocaine	2	2						
		codeine	3	3						
1903ai	41 y M				A	Ingst+ Par	Int-A	2		
		heroin	1	1						
		diphenhydramine	2	2						
		ethanol	3	3						
1904ai	41 y M				A	Unk	Int-A	2		
		heroin	1	1						
		cocaine	2	2						
		ethanol (non-beverage)	3	3						
1905ai	41 y M				U	Ingst	Int-A	2		
		methamphetamine	1	1						
1906ai	41 y F				A	Unk	Unt-G	2		
		heroin	1	1						
		diphenhydramine	2	2						
		citalopram	3	3						
1907a	42 y M				A	Unk	Int-A	1		
		methamphetamine	1	1					methamphetamine	3317 ng/mL In Urine (quantitative only) @ 0 d (pe)
		methamphetamine	1	1					methamphetamine	52.3 ng/mL In Blood (unspecified) @ Autopsy
		methamphetamine	1	1					amphetamine	618 ng/mL In Urine (quantitative only) @ 0 d (pe)
		methamphetamine	1	1					methamphetamine	89.3 ng/mL In Blood (unspecified) @ 0 d (pe)
		heroin	2	2					fentanyl	0.7 ng/mL In Blood (unspecified) @ Autopsy
		heroin	2	2					morphine	1000 ng/mL In Urine (quantitative only) @ 0 d (pe)
		heroin	2	2					hydromorphone	133 ng/mL In Urine (quantitative only) @ 0 d (pe)
		heroin	2	2					codeine	1648 ng/mL In Urine (quantitative only) @ 0 d (pe)
		heroin	2	2					codeine	19.3 ng/mL In Blood (unspecified) @ Autopsy
		heroin	2	2					fentanyl	2.8 ng/mL In Urine (quantitative only) @ 0 d (pe)
		heroin	2	2					morphine	252 ng/mL In Blood (unspecified) @ Autopsy
		heroin	2	2					6-monoacetylmorphine	310 ng/mL In Urine (quantitative only) @ 0 d (pe)
		heroin	2	2					codeine	41.6 ng/mL In Blood (unspecified) @ 0 d (pe)
		heroin	2	2					morphine	654 ng/mL In Blood (unspecified) @ 0 d (pe)
1908ai	42 y M				A	Ingst+ Unk	Int-A	2		
		heroin	1	1						
		cocaine	2	2						
		ethanol	3	3						
1909ai	42 y M				A	Par	Int-A	2		
		heroin	1	1						
		cocaine	2	2						
		methadone	3	3						
1910ai	42 y M				A	Ingst+ Unk	Int-A	2		
		heroin	1	1						
		ethanol	2	2						
1911ai	42 y M				A	Inhal+ Unk	Int-A	2		
		heroin	1	1						
		benzodiazepine	2	2						
		marijuana	3	3						
1912ai	43 y M				A	Ingst+ Unk	Int-A	2		
		heroin	1	1						
		cocaine	2	2						
		ethanol (non-beverage)	3	3						
		quinine	4	4						
1913ai	43 y M				A	Par	Int-A	2		
		heroin	1	1						
		dextromethorphan	2	2						
		codeine	3	3						
1914ai	43 y M				A	Unk	Int-A	2		
		heroin	1	1						
		cocaine	2	2						
		phencyclidine	3	3						
		dextromethorphan	4	4						
1915p	43 y F				A	Ingst	Int-A	2		
		cocaine	1	1					benzoylecognine	0.31 mg/L In Blood (unspecified) @ Autopsy
		cocaine	1	1					benzoylecognine	0.34 mg/L In Vitreous @ Autopsy
		methadone	2	2					methadone	0.06 mg/L In Blood (unspecified) @ Autopsy
		opioid	3	3						
		phencyclidine	4	4					phencyclidine	0.23 mg/L In Blood (unspecified) @ Autopsy
1916pha	43 y F				U	Ingst	Int-A	1		
		heroin	1	1					morphine (free)	370 ng/mL In Blood (unspecified) @ Unknown
		heroin	1	1					6-monoacetylmorphine	460 ng/mL In Blood (unspecified) @ Unknown
		carisoprodol	2	2					carisoprodol	0.42 mcg/mL In Blood (unspecified) @ Unknown
1917ai	44 y M				A	Unk	Int-A	2		
		heroin	1	1						
		cocaine	2	2						
		citalopram	3	3						
		hydrocodone	4	4						
		doxylamine	5	5						
1918ai	44 y F				U	Ingst+ Unk	Unk	2		
		methamphetamine	1	1						
		codeine	2	2						
		oxycodone	3	3						
		fluoxetine	4	4						
		diazepam	5	5						
1919ai	44 y M				A	Ingst+ Unk	Int-A	2		
		cocaine	1	1						
		ethanol	2	2						
1920ai	44 y M				A	Inhal	Int-A	2		
		heroin	1	1						
		alprazolam	2	2						
		cocaine	3	3						
		amitriptyline	4	4						
		diphenhydramine	5	5						
		oxycodone	6	6						
		quinine	7	7						
		codeine	8	8						
1921ai	44 y M				U	Unk	Int-A	2		
		methamphetamine	1	1						
		amphetamine	2	2						
1922ai	44 y M				U	Unk	Int-A	2		
		methamphetamine	1	1						
1923ai	44 y M				A	Unk	Int-A	2		
		heroin	1	1						
		oxycodone	2	2						
		dextromethorphan	3	3						
		ethanol	4	4						
1924ai	44 y M				U	Unk	Int-A	2		
		cocaine	1	1						
		acetone	2	2						
		cyclobenzaprine	3	3						
1925ai	44 y M				A	Ingst+ Unk	Int-A	2		
		heroin	1	1						
		methadone	2	2						
		trazodone	3	3						
		ethanol	4	4						
1926pha	44 y M				A	Ingst	Int-A	2		
		amphetamine (hallucinogenic)	1	1						
		methadone	2	2					methadone	0.2 mg/L In Serum @ 3 h (pe)
		ethanol	3	3					ethanol	0.01 g/dL In Serum @ 0 m (pe)
		benzodiazepine	4	4						
1927ai	44 y M				A	Inhal+ Par+ Unk	Int-A	2		
		heroin	1	1						
		cocaine	2	2						
		dextromethorphan	3	3						
		oxycodone	4	4						
		hydrocodone	5	5						
		acetaminophen	6	6						
		codeine	7	7						
1928ai	44 y F				U	Unk	Int-A	2		
		methamphetamine	1	1						
		morphine	2	2						
		fentanyl	3	3						
1929ai	44 y F				U	Unk	Int-A	2		
		methamphetamine	1	1						
1930ai	45 y M				A	Ingst+ Par	Int-A	2		
		heroin	1	1						
		trazodone	2	2						
		citalopram	3	3						
		doxepin	4	4						
		quinine	5	5						
		codeine	6	6						
1931ai	45 y M				U	Unk	Int-A	2		
		cocaine	1	1						
1932ai	45 y M				A	Ingst+ Unk	Int-A	2		
		cocaine	1	1						
		ethanol	2	2						
1933ai	45 y M				A	Inhal	Int-A	2		
		heroin	1	1						
		fluoxetine	2	2						
		diphenhydramine	3	3						
		codeine	4	4						
1934ai	45 y M				A	Unk	Int-A	2		
		heroin	1	1						
		cocaine	2	2						
		oxycodone	3	3						
		codeine	4	4						
1935ai	45 y M				A	Ingst+ Par	Int-A	2		
		heroin	1	1						
		tramadol	2	2						
		oxycodone	3	3						
		ethanol	4	4						
1936ph	45 y M				A	Unk	Int-U	2		
		heroin	1	1						
1937p	45 y F				A	Unk	Int-A	1		
		methamphetamine	1	1						
		ethanol	2	2						
1938ai	46 y F				U	Unk	Int-A	2		
		cocaine	1	1						
1939ai	46 y M				A	Unk	Int-A	2		
		heroin	1	1						
		cocaine	2	2						
		ethanol (non-beverage)	3	3						
1940ai	46 y M				A	Unk	Int-A	2		
		heroin	1	1						
		cocaine	2	2						
1941ai	46 y M				A	Unk	Int-A	2		
		heroin	1	1						
		diazepam	2	2						
		oxycodone	3	3						
1942ai	46 y M				A	Unk	Int-A	2		
		heroin	1	1						
		clonazepam	2	2						
		quetiapine	3	3						
		citalopram	4	4						
		acetaminophen	5	5						
		buspirone	6	6						
1943ai	46 y M				A	Ingst+ Par	Int-A	2		
		heroin	1	1						
		cocaine	2	2						
		diphenhydramine	3	3						
		dextromethorphan	4	4						
		quinine	5	5						
		codeine	6	6						
		ethanol	7	7						
1944ai	46 y M				A	Ingst+ Par	Int-A	2		
		heroin	1	1						
		codeine	2	2						
		ethanol	3	3						
1945ai	46 y M				A	Ingst+ Par	Int-U	2		
		heroin	1	1						
		paroxetine	2	2						
		hydroxyzine	3	3						
		doxylamine	4	4						
		dextromethorphan	5	5						
		ethanol	6	6						
1946ai	47 y F				A	Unk	Int-A	2		
		heroin	1	1						
		diphenhydramine	2	2						
1947ai	47 y F				U	Unk	Int-A	2		
		methamphetamine	1	1						
		acetaminophen/hydrocodone	2	2						
1948ai	47 y M				U	Unk	Int-A	2		
		methamphetamine	1	1						
1949ai	47 y M				U	Ingst+ Par	Int-A	2		
		heroin	1	1						
		acetaminophen/hydrocodone	2	2						
1950ai	47 y M				A	Ingst+ Unk	Int-A	2		
		cocaine	1	1						
		phenobarbital	2	2						
1951h	47 y M				U	Unk	Int-A	2		
		cocaine/heroin	1	1						
1952ai	47 y M				A	Par+ Unk	Int-A	2		
		heroin	1	1						
		clonazepam	2	2						
1953ai	47 y M				A	Ingst+ Unk	Int-A	2		
		heroin	1	1						
		ethanol	2	2						
1954ai	47 y M				A	Ingst+ Inhal	Int-A	2		
		heroin	1	1						
		codeine	2	2						
		ethanol	3	3						
1955ai	48 y F				A	Inhal	Int-A	2		
		cocaine	1	1						
1956ai	48 y M				A	Ingst+ Unk	Int-A	2		
		cocaine	1	1						
		ethanol (non-beverage)	2	2						
		primidone	3	3						
1957ai	48 y M				A	Ingst+ Par	Int-A	2		
		heroin	1	1						
		olanzapine	2	2						
		quinine	3	3						
		codeine	4	4						
1958ai	48 y M				A	Unk	Int-A	2		
		heroin	1	1						
		methadone	2	2						
		ethanol	3	3						
1959ai	48 y M				U	Unk	Int-A	2		
		methamphetamine	1	1						
1960ai	48 y M				A	Unk	Int-A	2		
		cocaine	1	1						
		oxycodone	2	2						
		diphenhydramine	3	3						
		acetaminophen	4	4						
1961	48 y M				U	Unk	Unk	2		
		cocaine	1	1						
1962ai	48 y M				A	Ingst+ Unk	Int-A	2		
		heroin	1	1						
		ethanol	2	2						
1963ph	48 y M				A	Ingst	Int-A	2		
		cocaine	1	1						
		amphetamine	2	2						
		marijuana	3	3						
1964ai	49 y M				A	Unk	Int-A	2		
		heroin	1	1						
		methadone	2	2						
1965ai	49 y F				A	Par	Int-A	2		
		heroin	1	1						
		chlordiazepoxide	2	2						
		quinine	3	3						
1966ai	49 y M				A	Unk	Int-A	2		
		heroin	1	1						
		cocaine	2	2						
		citalopram	3	3						
		dextromethorphan	4	4						
1967ai	49 y F				A	Unk	Int-A	2		
		heroin	1	1						
		clonazepam	2	2						
		cocaine	3	3						
		nortriptyline	4	4						
		sertraline	5	5						
		cyclobenzaprine	6	6						
		diphenhydramine	7	7						
1968ai	49 y M				A	Ingst+ Unk	Int-A	2		
		heroin	1	1						
		ethanol (non-beverage)	2	2						
		cocaine	3	3						
1969ph	49 y M				A/C	Inhal	Int-A	2		
		THC homolog	1	1						
		drug, unknown	2	2						
1970ai	49 y F				A	Unk	Int-A	2		
		heroin	1	1						
		quetiapine	2	2						
		sertraline	3	3						
1971ai	49 y M				A	Ingst+ Unk	Int-A	2		
		heroin	1	1						
		cocaine	2	2						
		benzodiazepine	3	3						
		quinine	4	4						
		ethanol	5	5						
1972ai	49 y M				A	Unk	Int-A	2		
		heroin	1	1						
		cocaine	2	2						
		diphenhydramine	3	3						
		dextromethorphan	4	4						
		bupropion	5	5						
		codeine	6	6						
1973ai	49 y M				A	Ingst+ Par	Int-A	2		
		heroin	1	1						
		oxycodone	2	2						
		alprazolam	3	3						
		quinine	4	4						
		ethanol	5	5						
1974ph	49 y M				A	Ingst	Int-S	2		
		heroin	1	1						
		methamphetamine	2	2					methamphetamine	374 ng/mL In Blood (unspecified) @ Autopsy
1975ai	49 y F				A	Unk	Int-A	2		
		heroin	1	1						
		amitriptyline	2	2						
		clonazepam	3	3						
		alprazolam	4	4						
		sertraline	5	5						
		quinine	6	6						
1976ai	49 y M				U	Unk	Int-A	2		
		methamphetamine	1	1						
		methadone	2	2						
		quetiapine	3	3						
		alprazolam	4	4						
1977ai	49 y M				U	Unk	Int-A	2		
		methamphetamine	1	1						
1978pa	49 y F				U	Unk	Int-A	1		
		cocaine	1	1					cocaine	180 ng/mL In Whole Blood @ Autopsy
		cocaine	1	1					benzoylecognine	710 ng/mL In Whole Blood @ Autopsy
		fentanyl	2	2					fentanyl	18 ng/mL In Whole Blood @ Autopsy
		fentanyl	2	2					norfentanyl	7.2 ng/mL In Whole Blood @ Autopsy
		marijuana	3	3					delta-9-carboxy-thc	6.5 ng/mL In Whole Blood @ Autopsy
		zonisamide	4	4					zonisamide	30 mcg/mL In Whole Blood @ Autopsy
1979ai	50 y F				A	Ingst+ Unk	Int-A	2		
		heroin	1	1						
		citalopram	2	2						
		bupropion	3	3						
		diphenhydramine	4	4						
		quinine	5	5						
		ethanol	6	6						
1980ph	50 y M				A	Par	Int-A	1		
		heroin	1	1						
1981ai	50 y F				A	Unk	Int-A	2		
		heroin	1	1						
		quinine	2	2						
1982ai	50 y M				U	Unk	Int-A	2		
		methamphetamine	1	1						
1983ai	50 y M				A	Unk	Int-A	2		
		heroin	1	1						
1984ai	50 y M				A	Ingst+ Unk	Int-A	2		
		heroin	1	1						
		citalopram	2	2						
		ethanol	3	3						
1985ai	50 y F				A	Ingst+ Unk	Int-A	2		
		cocaine	1	1						
		diphenhydramine	2	2						
		acetaminophen	3	3						
1986ai	50 y M				A	Ingst+ Unk	Int-A	2		
		cocaine	1	1						
		ethanol	2	2						
1987ai	50 y M				A	Ingst+ Unk	Int-A	2		
		heroin	1	1						
		levetiracetam	2	2						
		ethanol	3	3						
1988ai	50 y F				A	Ingst+ Par	Int-A	2		
		heroin	1	1						
		ethanol	2	2						
1989a	50 y M				A	Ingst+ Unk	Int-S	1		
		heroin	1	1						
		methadone	2	2					methadone	0.1 mg/L In Blood (unspecified) @ Autopsy
		oxycodone	3	3					oxycodone	0.08 mg/L In Blood (unspecified) @ Autopsy
		alprazolam	4	4					alprazolam	0.04 mg/L In Blood (unspecified) @ Autopsy
		verapamil	5	5					verapamil	6.5 mg/L In Blood (unspecified) @ Autopsy
		acetaminophen	6	6						
1990ai	51 y M				A	Unk	Int-A	2		
		heroin	1	1						
		diphenhydramine	2	2						
		dextromethorphan	3	3						
		codeine	4	4						
		lidocaine	5	5						
1991ai	51 y F				A	Ingst+ Unk	Int-A	2		
		heroin	1	1						
		cocaine	2	2						
		oxycodone	3	3						
		trazodone	4	4						
		alprazolam	5	5						
		cyclobenzaprine	6	6						
		quinine	7	7						
		codeine	8	8						
		ethanol	9	9						
1992ai	51 y M				A	Ingst+ Inhal	Int-A	2		
		heroin	1	1						
		ethanol	2	2						
		chlorpheniramine	3	3						
1993ai	51 y M				A	Ingst+ Par	Int-A	2		
		heroin	1	1						
		citalopram	2	2						
		ethanol	3	3						
1994ai	51 y M				A	Par+ Unk	Int-A	2		
		heroin	1	1						
		chlordiazepoxide	2	2						
1995ai	51 y M				A	Unk	Int-A	2		
		cocaine	1	1						
1996h	51 y F				C	Unk	Unk	2		
		cocaine	1	1						
		hyperthermia	2	2						
1997p	51 y M				C	Ingst+ Par	Int-A	1		
		methamphetamine	1	1						
		non-powder, unknown	2	2						
1998ai	52 y M				A	Ingst+ Unk	Int-A	2		
		heroin	1	1						
		levetiracetam	2	2						
		fluoxetine	3	3						
		ethanol	4	4						
1999ai	52 y M				A	Unk	Int-A	2		
		heroin	1	1						
2000ai	52 y M				U	Unk	Int-A	2		
		methamphetamine	1	1						
2001ai	52 y F				U	Unk	Int-A	2		
		cocaine	1	1						
		methamphetamine	2	2						
2002ai	52 y M				A	Par+ Unk	Int-A	2		
		heroin	1	1						
		cyclobenzaprine	2	2						
		diltiazem	3	3						
		quinine	4	4						
		codeine	5	5						
2003ai	52 y M				A	Ingst+ Par	Int-A	2		
		heroin	1	1						
		cocaine	2	2						
		phencyclidine	3	3						
		ethanol	4	4						
2004ai	52 y M				A	Ingst+ Par	Int-A	2		
		heroin	1	1						
		metoprolol	2	2						
2005	52 y F				U	Ingst	Int-S	2		
		amphetamine	1	1						
2006ai	53 y M				A	Unk	Int-A	2		
		heroin	1	1						
		cocaine	2	2						
		hydroxyzine	3	3						
2007ai	53 y M				A	Par+ Unk	Int-A	2		
		heroin	1	1						
		cocaine	2	2						
		diazepam	3	3						
		fluoxetine	4	4						
		codeine	5	5						
		quinine	6	6						
2008ai	53 y M				A	Ingst+ Par	Int-A	2		
		heroin	1	1						
		diazepam	2	2						
		quinine	3	3						
		ethanol	4	4						
2009ai	53 y F				U	Unk	Int-A	2		
		methamphetamine	1	1						
2010ai	53 y F				A	Ingst+ Par	Int-A	2		
		heroin	1	1						
		paroxetine	2	2						
2011ai	53 y F				A	Ingst+ Unk	Int-A	2		
		heroin	1	1						
		doxepin	2	2						
		fluoxetine	3	3						
		ethanol	4	4						
2012ai	53 y F				A	Unk	Int-A	2		
		heroin	1	1						
		ethanol	2	2						
2013	53 y M				A	Ingst+ Unk	Int-U	2		
		cocaine	1	1						
		ethanol	2	2					ethanol	536 mg/dL In Blood (unspecified) @ Unknown
2014pha	53 y F				A/C	Unk	Int-A	1		
		cocaine	1	1						
		heroin	2	2						
2015ai	54 y M				A	Unk	Int-A	2		
		heroin	1	1						
		oxycodone	2	2						
		diazepam	3	3						
		levetiracetam	4	4						
		citalopram	5	5						
		dextromethorphan	6	6						
		quinine	7	7						
		ethanol	8	8						
2016ai	54 y M				A	Par	Int-A	2		
		heroin	1	1						
		cocaine	2	2						
2017ai	54 y M				U	Unk	Int-A	2		
		methamphetamine	1	1						
2018ai	54 y M				A	Ingst+ Par	Int-A	2		
		heroin	1	1						
		ethanol (non-beverage)	2	2						
		quinine	3	3						
2019ai	54 y M				A	Unk	Int-A	2		
		heroin	1	1						
		methadone	2	2						
		promethazine	3	3						
		diphenhydramine	4	4						
		clonazepam	5	5						
		quinine	6	6						
		codeine	7	7						
2020ai	54 y M				U	Unk	Int-A	2		
		cocaine	1	1						
2021ai	54 y M				A	Unk	Int-A	2		
		heroin	1	1						
		clonazepam	2	2						
		phenytoin	3	3						
		zolpidem	4	4						
		promethazine	5	5						
		codeine	6	6						
2022ai	54 y M				A	Unk	Int-A	2		
		heroin	1	1						
		cocaine	2	2						
		chlordiazepoxide	3	3						
		quinine	4	4						
2023ai	54 y F				U	Ingst+ Unk	Int-A	2		
		methamphetamine	1	1						
		methadone	2	2						
2024ai	54 y F				U	Unk	Int-A	2		
		methamphetamine	1	1						
2025ai	54 y M				A	Ingst+ Par	Int-A	2		
		heroin	1	1						
		metoprolol	2	2						
		ethanol	3	3						
2026h	54 y M				A/C	Unk	Int-A	3		
		cocaine	1	1						
2027ai	55 y M				U	Unk	Int-A	2		
		cocaine	1	1						
2028ai	55 y M				U	Unk	Int-A	2		
		methamphetamine	1	1						
2029ai	55 y F				A	Unk	Int-A	2		
		heroin	1	1						
		cocaine	2	2						
		ethanol (non-beverage)	3	3						
2030	55 y F				U	Ingst+ Unk	Int-A	2		
		heroin	1	1					morphine	0.14 mg/L In Blood (unspecified) @ Autopsy
		opioid	2	2						
		ethanol	3	3					ethanol	87 mg/dL In Blood (unspecified) @ Unknown
2031ai	55 y M				U	Unk	Int-A	2		
		methamphetamine	1	1						
2032ai	55 y M				A	Ingst+ Unk	Int-A	2		
		heroin	1	1						
		chlordiazepoxide	2	2						
		ethanol	3	3						
2033ai	55 y F				A	Ingst+ Unk	Int-A	2		
		heroin	1	1						
		diltiazem	2	2						
		ethanol	3	3						
2034	55 y M				A	Ingst	Int-S	1		
		methylenedioxymethamphetamine (MDMA)	1	1						
		Hydromorphone	2	2						
2035ai	56 y F				A	Ingst+ Unk	Int-A	2		
		heroin	1	1						
		fluoxetine	2	2						
2036ai	56 y M				A	Ingst+ Par	Int-A	2		
		heroin	1	1						
		cocaine	2	2						
		methadone	3	3						
		ethanol	4	4						
2037ai	56 y M				A	Ingst+ Inhal	Int-A	2		
		heroin	1	1						
		cocaine	2	2						
		codeine	3	3						
		ethanol	4	4						
2038ai	56 y M				A	Ingst+ Par	Int-A	2		
		heroin	1	1						
		ethanol	2	2						
2039ai	56 y M				A	Par	Int-A	2		
		heroin	1	1						
		codeine	2	2						
		quinine	3	3						
2040pha	56 y M				A	Ingst+ Inhal	Int-A	2		
		amyl-butyl nitrites	1	1						
		drug, unknown	2	2						
		ethanol	3	3					ethanol	37 mg/dL In Blood (unspecified) @ Unknown
2041ai	57 y M				A	Unk	Int-A	2		
		heroin	1	1						
		oxycodone	2	2						
		diphenhydramine	3	3						
		doxylamine	4	4						
		codeine	5	5						
2042ai	57 y M				A	Unk	Int-A	2		
		heroin	1	1						
		cocaine	2	2						
2043ai	57 y M				U	Unk	Int-A	2		
		cocaine	1	1						
2044ai	57 y M				A	Unk	Int-A	2		
		heroin	1	1						
		alprazolam	2	2						
		diazepam	3	3						
		trazodone	4	4						
		paroxetine	5	5						
		citalopram	6	6						
		quinine	7	7						
		ethanol	8	8						
2045ai	57 y M				A	Unk	Int-A	2		
		cocaine	1	1						
2046ai	57 y M				U	Unk	Int-A	2		
		methamphetamine	1	1						
		diazepam	2	2						
2047ai	57 y F				A	Ingst+ Unk	Int-A	2		
		heroin	1	1						
		diazepam	2	2						
		quinine	3	3						
		ethanol	4	4						
2048ai	57 y M				A	Par	Int-A	2		
		heroin	1	1						
		quinine	2	2						
2049ai	58 y M				A	Unk	Int-A	2		
		heroin	1	1						
		cocaine	2	2						
		diphenhydramine	3	3						
		propranolol	4	4						
		mirtazapine	5	5						
		codeine	6	6						
2050ai	58 y M				A	Ingst+ Unk	Int-A	2		
		heroin	1	1						
		hydrocodone	2	2						
		trazodone	3	3						
		quetiapine	4	4						
		codeine	5	5						
		acetaminophen	6	6						
2051ai	58 y M				A	Unk	Int-A	2		
		heroin	1	1						
		trazodone	2	2						
		dextromethorphan	3	3						
		codeine	4	4						
2052ai	58 y M				A	Ingst+ Unk	Int-A	2		
		heroin	1	1						
		ethanol (non-beverage)	2	2						
		diphenhydramine	3	3						
		dextromethorphan	4	4						
		methamphetamine	5	5						
		quinine	6	6						
2053ai	58 y M				A	Par+ Unk	Int-A	2		
		heroin	1	1						
		cocaine	2	2						
		diltiazem	3	3						
		quinine	4	4						
2054ai	58 y M				U	Unk	Int-A	2		
		methamphetamine	1	1						
2055ai	58 y M				A	Unk	Int-A	2		
		cocaine	1	1						
2056ai	58 y F				A	Ingst+ Unk	Int-A	2		
		heroin	1	1						
		ethanol	2	2						
2057ai	58 y M				A	Unk	Int-A	2		
		heroin	1	1						
		cocaine	2	2						
2058ai	59 y M				U	Unk	Int-A	2		
		cocaine	1	1						
2059ai	59 y M				U	Unk	Int-A	2		
		cocaine	1	1						
		acetaminophen/hydrocodone	2	2						
		verapamil	3	3						
		amitriptyline	4	4						
		cyclobenzaprine	5	5						
		alprazolam	6	6						
2060ai	59 y M				A	Par	Int-A	2		
		heroin	1	1						
		sertraline	2	2						
		cocaine	3	3						
2061pha	59 y F				U	Unk	Int-U	3		
		cocaine	1	1					benzoylecognine	1540 ng/mL In Blood (unspecified) @ Unknown
		cocaine	1	1					ecgonine methyl ester	36.3 ng/mL In Blood (unspecified) @ Unknown
		fentanyl	2	2						
		fentanyl (transdermal)	3	3						
		naproxen	4	4						
[2062a]	59 y F				C	Ingst	AR-D	3		
		dimethylamylamine	1	1						
2063ai	60 y M				A	Inhal	Int-A	2		
		cocaine	1	1						
2064ai	60 y M				U	Unk	Int-A	2		
		methamphetamine	1	1						
		morphine	2	2						
2065ai	60 y M				A	Ingst+ Par	Int-A	2		
		heroin	1	1						
		quinine	2	2						
		ethanol	3	3						
2066ai	61 y M				A	Ingst+ Par	Int-A	2		
		heroin	1	1						
		methadone	2	2						
		cocaine	3	3						
		diphenhydramine	4	4						
		quinine	5	5						
		ethanol	6	6						
2067ai	61 y M				A	Ingst+ Unk	Int-A	2		
		heroin	1	1						
		ethanol	2	2						
2068ai	61 y M				A	Ingst+ Unk	Int-A	2		
		heroin	1	1						
		cocaine	2	2						
		quinine	3	3						
		ethanol	4	4						
2069pa	63 y M				A	Ingst	Int-U	1		
		heroin	1	1					morphine	10000 ng/mL In Urine (quantitative only) @ Autopsy
		heroin	1	1					6-monoacetylmorphine	777 ng/mL In Urine (quantitative only) @ Autopsy
		fentanyl	2	2					fentanyl	1.3 ng/mL In Urine (quantitative only) @ Autopsy
		fentanyl	2	2					norfentanyl	11.8 ng/mL In Urine (quantitative only) @ Autopsy
2070ai	64 y M				A	Ingst+ Par	Int-A	2		
		heroin	1	1						
		amitriptyline	2	2						
		quinine	3	3						
		codeine	4	4						
		ethanol	5	5						
2071ai	64 y F				A	Unk	Int-A	2		
		heroin	1	1						
2072ai	65 y M				U	Unk	Int-A	2		
		methamphetamine	1	1						
		acetaminophen/hydrocodone	2	2						
2073ai	65 y F				U	Unk	Int-A	2		
		methamphetamine	1	1						
2074ai	66 y M				A	Ingst+ Unk	Int-A	2		
		heroin	1	1						
		chlordiazepoxide	2	2						
		diphenhydramine	3	3						
2075ai	68 y F				U	Unk	Int-A	2		
		methamphetamine	1	1						
2076ai	68 y M				A	Unk	Int-A	2		
		heroin	1	1						
		cocaine	2	2						
		diltiazem	3	3						
		codeine	4	4						
2077ai	68 y M				A	Par	Int-A	2		
		heroin	1	1						
		quinine	2	2						
2078ai	75 y M				A	Unk	Int-A	2		
		cocaine	1	1						
2079ai	88 y M				U	Unk	Int-A	2		
		methamphetamine	1	1						
		amphetamine	2	2						
[2080pha]	20 + y M				U	Ingst	Int-A	1		
		cocaine	1	1					benzoylecognine	2700 ng/mL In Serum @ Unknown
		cocaine	1	1					cocaine	2900 ng/mL In Serum @ Unknown
See Also case 5, 6, 11, 28, 43, 49, 70, 162, 235, 240, 244, 267, 268, 273, 275, 346, 407, 420, 437, 447, 457, 465, 493, 497, 499, 500, 518, 527, 536, 546, 579, 584, 587, 590, 591, 592, 597, 601, 619, 637, 644, 650, 654, 655, 663, 677, 679, 682, 685, 695, 699, 702, 706, 745, 750, 753, 770, 780, 783, 784, 788, 791, 794, 795, 808, 809, 825, 828, 833, 864, 867, 877, 882, 905, 907, 910, 912, 919, 928, 955, 961, 974, 988, 1007, 1092, 1119, 1155, 1164, 1167, 1177, 1185, 1187, 1198, 1203, 1226, 1232, 1241, 1252, 1281, 1290, 1294, 1299, 1317, 1323, 1342, 1351, 1368, 1449, 1491, 1504, 1516, 1566, 1567, 1576, 1589, 1597, 1600, 1609, 1615, 1621, 1631, 2083, 2086										
**Topical Preparations**										
2081	87 y M				A	Ingst	Unt-T	2		
		methyl salicylate	1	1						
										
**Unknown Drug**										
2082ai	14 y M				U	Ingst	Unk	2		
		drug, unknown	1	1						
2083ha	14 y M				A	Unk	Int-A	2		
		drug, unknown	1	1						
		drug, unknown	2	2						
		ketamine	3	3					norketamine	390 ng/mL In Serum @ Unknown
		ketamine	3	3					ketamine	460 ng/mL In Serum @ Unknown
		marijuana	4	4					delta-9-carboxy-thc	34 ng/mL In Serum @ Unknown
2084ai	19 y F				U	Unk	Unk	2		
		drug, unknown	1	1						
2085p	21 y M				A	Ingst	Int-S	2		
		drug, unknown	1	1						
2086	25 y M				U	Unk	Unk	1		
		drug, unknown	1	1						
		amphetamine	2	2						
2087pai	27 y F				A	Unk	Unt-U	3		
		drug, unknown	1	1						
2088ha	28 y F				A	Unk	Int-U	2		
		drug, unknown	1	1						
2089ai	28 y F				U	Unk	Int-A	2		
		drug, unknown	1	1						
2090	28 y F				A	Par	AR-D	2		
		drug, unknown	1	1						
2091pha	29 y M				A	Inhal	Int-A	1		
		drug, unknown	1	1						
		oxycodone	2	2					oxycodone	0.031 mg/L In Blood (unspecified) @ Autopsy
		oxycodone	2	2					oxycodone	0.049 mg/L In Blood (unspecified) @ Unknown
		oxycodone	2	2					oxycodone	0.47 mg/L In Gastric (stomach content) @ Autopsy
		alprazolam	3	3					alprazolam	0.06 mg/L In Blood (unspecified) @ Autopsy
		alprazolam	3	3					alprazolam	0.078 mg/L In Blood (unspecified) @ Unknown
		fentanyl	4	4					fentanyl	10.8 mcg/L In Blood (unspecified) @ Autopsy
2092p	29 y F				U	Unk	Int-S	2		
		drug, unknown	1	1						
2093ph	30 y M				A	Ingst	Int-S	2		
		drug, unknown	1	1						
		ethanol	2	2						
2094	31 y M				U	Ingst	Int-A	1		
		drug, unknown	1	1						
2095ai	33 y M				U	Unk	Int-A	2		
		drug, unknown	1	1						
2096h	34 y F				A	Ingst+ Unk	Int-U	2		
		drug, unknown	1	1						
		acetaminophen	2	2					acetaminophen	104 mcg/mL In Serum @ Unknown
		emtricitabine/tenofovir	3	3						
2097	35 y M				A	Ingst	Unk	2		
		drug, unknown	1	1						
2098p	36 y M				A	Ingst	Int-A	2		
		drug, unknown	1	1						
		ethanol	2	2						
2099ph	38 y F				U	Ingst	Int-U	1		
		drug, unknown	1	1						
2100a	43 y F				U	Ingst	Int-S	2		
		drug, unknown	1	1						
		alprazolam	2	2						
		diazepam	3	3						
2101ha	44 y F				A	Ingst	Unk	2		
		drug, unknown	1	1						
2102ph	45 y M				U	Unk	Int-S	2		
		drug, unknown	1	1						
		beta blocker	2	2						
2103p	45 y M				A	Par	Int-S	2		
		drug, unknown	1	1						
		oxycodone	2	2						
2104h	45 y F				U	Inhal	Int-S	2		
		drug, unknown	1	1						
		paint (aerosol)	2	2						
2105	47 y M				A	Unk	Unk	3		
		drug, unknown	1	1						
2106h	48 y F				U	Unk	Int-S	3		
		drug, unknown	1	1						
		acetaminophen/opioid	2	2					acetaminophen	149 mcg/mL In Unknown @ Unknown
		ethanol	3	3						
2107ph	53 y F				A	Ingst	Int-S	3		
		drug, unknown	1	1						
2108h	53 y M				A/C	Ingst	Int-S	1		
		drug, unknown	1	1						
		diltiazem	2	2						
		nifedipine	3	3						
		metoprolol	4	4						
		hydralazine	5	5						
2109p	56 y F				U	Ingst	Int-S	3		
		drug, unknown	1	1						
2110	59 y F				A	Ingst	Int-S	2		
		drug, unknown	1	1						
		quetiapine	2	2						
		duloxetine	3	3						
		clonazepam	4	4						
		vitamins (multiple)/iron	5	5						
2111ai	70 y F				U	Ingst	Int-A	2		
		drug, unknown	1	1						
2112	85 y F				A	Ingst	Int-S	2		
		drug, unknown	1	1						
See Also case 70, 175, 179, 451, 454, 546, 656, 713, 714, 915, 1029, 1193, 1260, 1336, 1349, 1461, 1484, 1577, 1603, 1633, 1766, 1771, 1780, 1791, 1969, 2040										
**Veterinary Drugs**										
2113	31 y M				A	Ingst+ Aspir	Int-S	1		
		veterinary drug, unknown	1	1						
See Also case 1643										

Listing of 2,477 (1,218 Direct + 1,259 Indirect) fatalities classified as Relative Contribution to Fatality category = 1-Undoubtedly responsible, 2-Probably responsible, or 3-Contributory). **Annual Report ID:** Bracketed [case number] = Narrative provided for this case in Appendix C **i =** Indirect case; identified through other sources (news feeds, medical examiner data, or other) about which no inquiry to the PC was made, **p =** prehospital cardiac and/or respiratory arrest, **h =** hospital records reviewed, **a =** autopsy report reviewed. **Age Gender: y =** years, **m =** months, **d =** days, **F =** female, **M =** male, **F-Pregnant =** pregnant, **U =** unknown **Chronicity: C =** chronic exposure, **A =** acute exposure, **A/C =** acute on chronic, **U =** unknown **Route: Aspir =** Aspiration (with ingestion), **B-S =** Bite/sting, **Derm =** Dermal, **Ingst =** Ingestion, **Inhal =** Inhalation/nasal, **Oc =** Ocular, **Ot =** Otic, **Oth =** Other, **Par =** Parenteral, **Rec =** Rectal, **Unk =** Unknown, **Vag =** Vaginal **Reason: AR-D =** Adverse reaction – Drug, **AR-F =** AR – Food, **AR-O =** AR – Other, **Int-A =** Intentional – Abuse, **Int-M =** Int – Misuse, **Int-S =** Int – Suspected Suicide, **Int-U =** Int – Unknown, **Oth-C =** Other – Contamination/tampering, **Oth-M =** Oth – Malicious, **Oth-W =** Oth – Withdrawal, **Unk =** Unknown reason, **Unt-B =** Unintentional – Bite/sting, **Unt-E =** Unt – Environmental, **Unt-F =** Unt—Food poisoning, **Unt-G =** Unt— General, **Unt-M =** Unt – Misuse, **Unt-O =** Unt – Occupational, **Unt-T =** Unt—Therapeutic error, **Unt-U** = Unt—Unknown **RCF (Relative Contribution to Fatality):** 1 = Undoubtedly responsible, 2 = Probably responsible, 3 = Contributory. Provided by the RPC for Indirect cases and the AAPCC Fatality Review Team for the direct (non-Indirect) cases.

**Table 22A.**
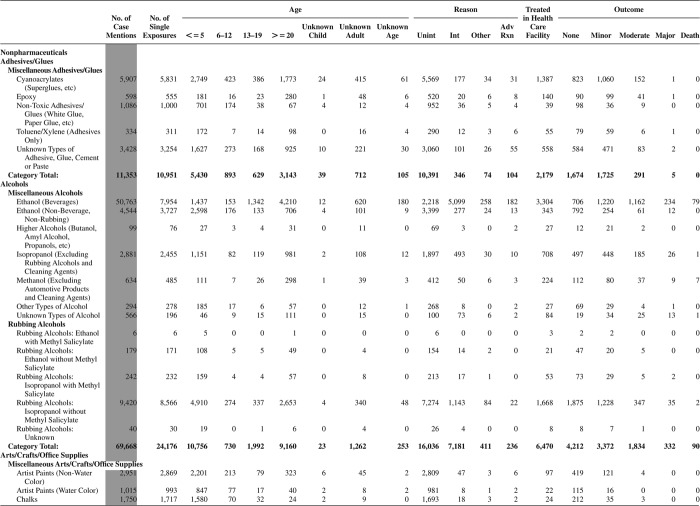
Demographic profile of SINGLE SUBSTANCE Nonpharmaceuticals exposure cases by generic category.

**Figure:**
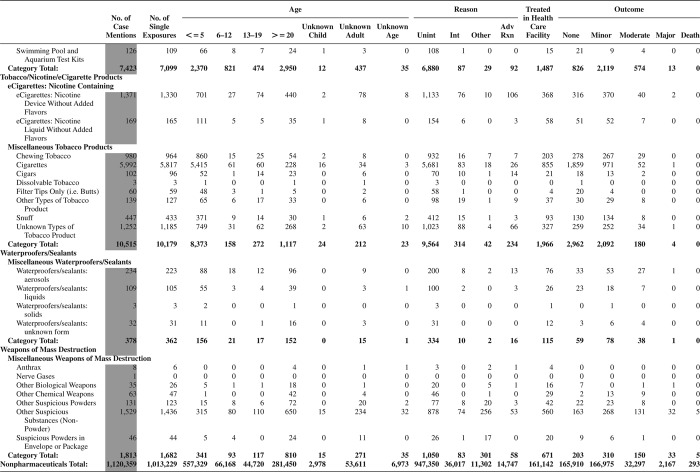


**Figure:**
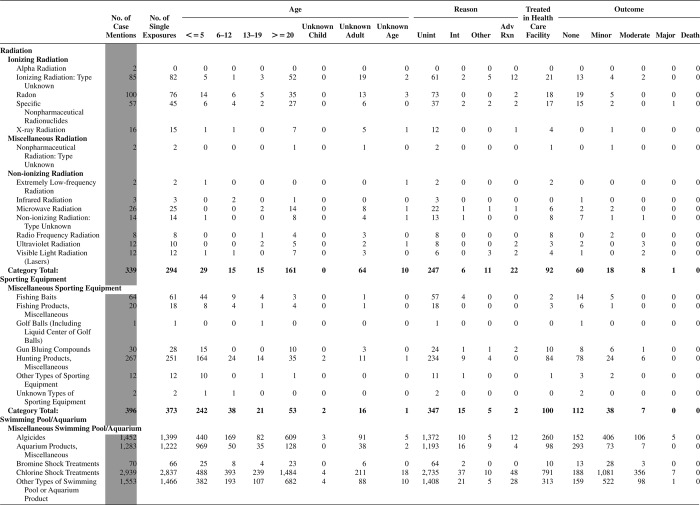


**Figure:**
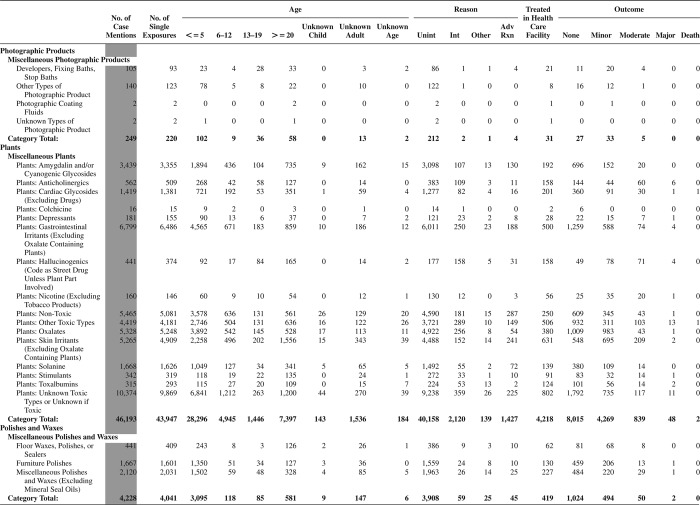


**Figure:**
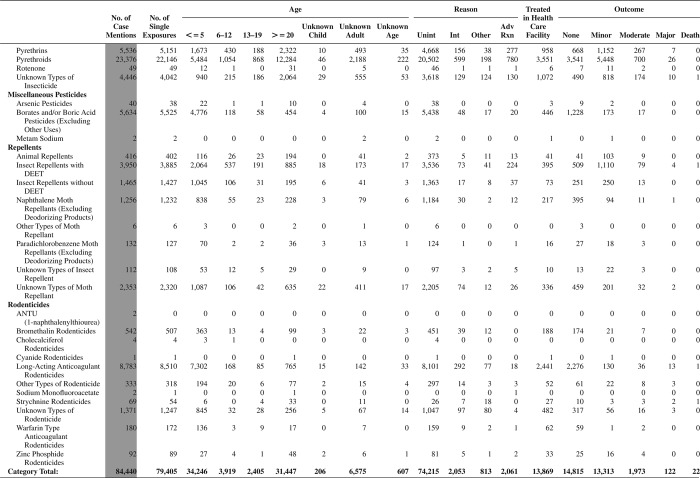


**Figure:**
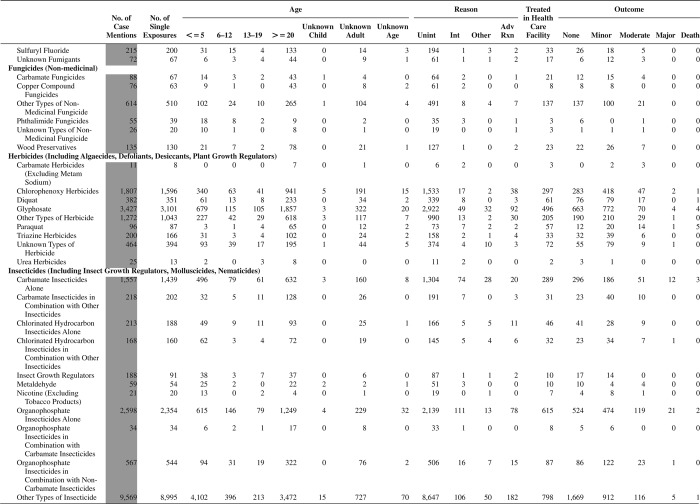


**Figure:**
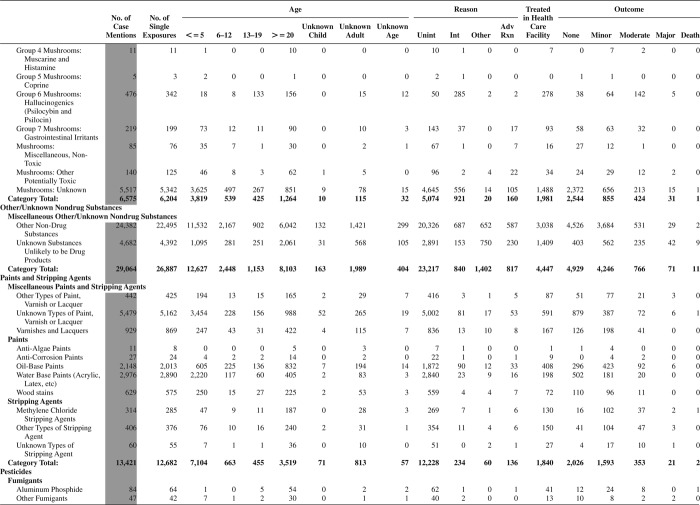


**Figure:**
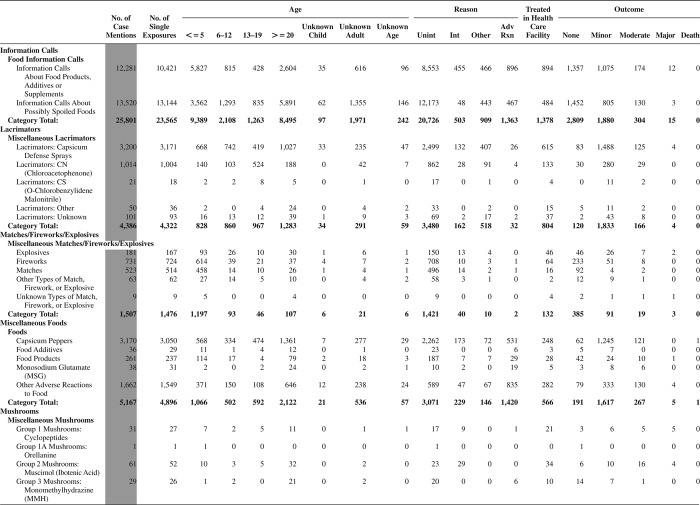


**Figure:**
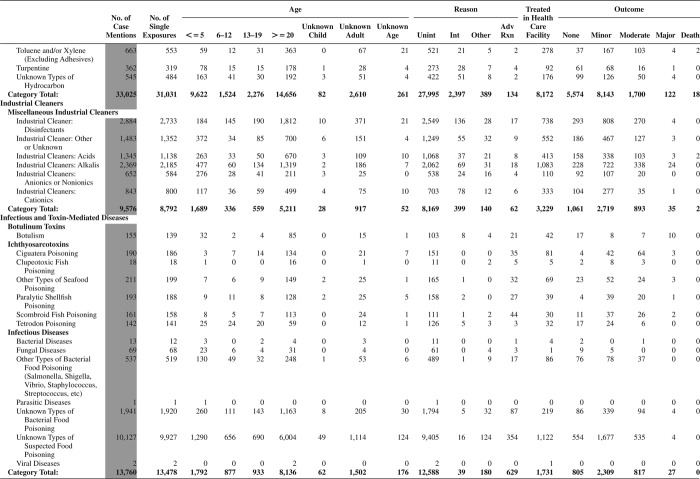


**Figure:**
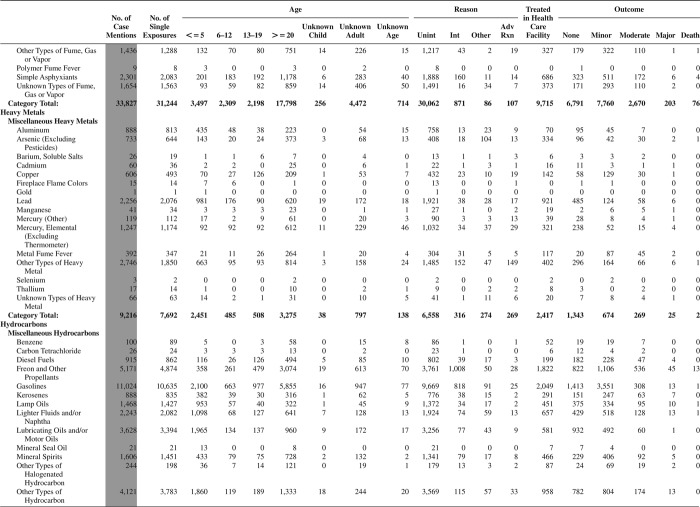


**Figure:**
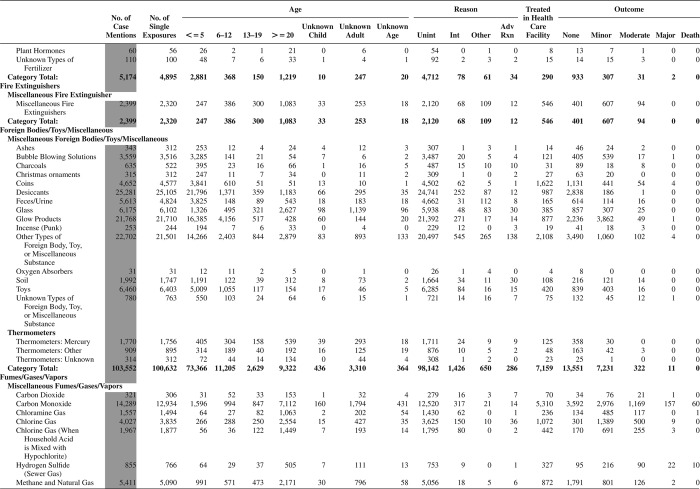


**Figure:**
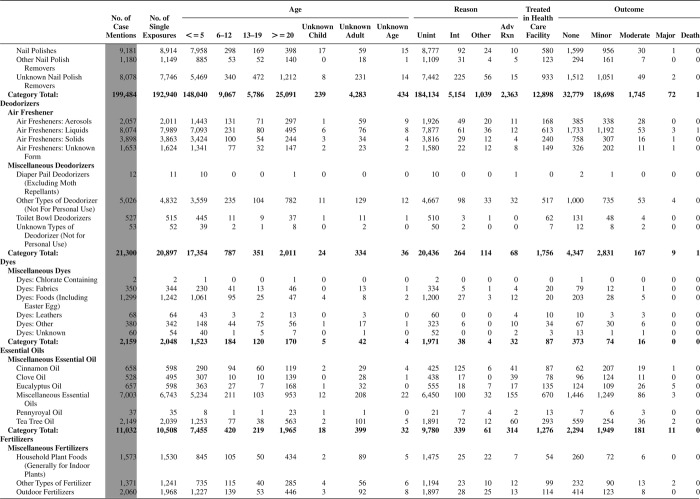


**Figure:**
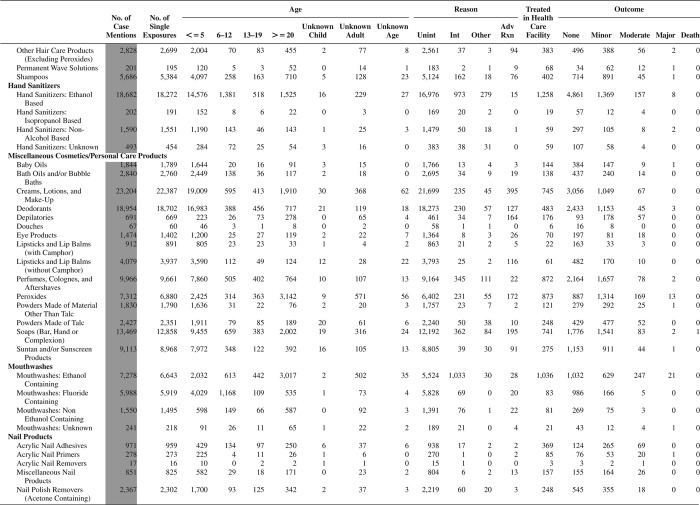


**Figure:**
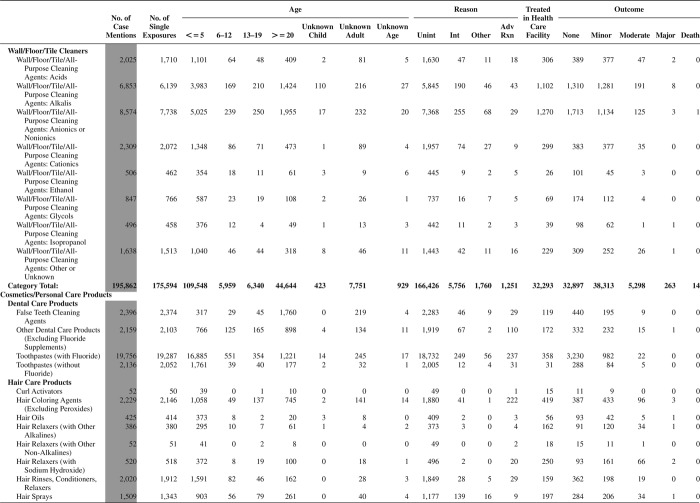


**Figure:**
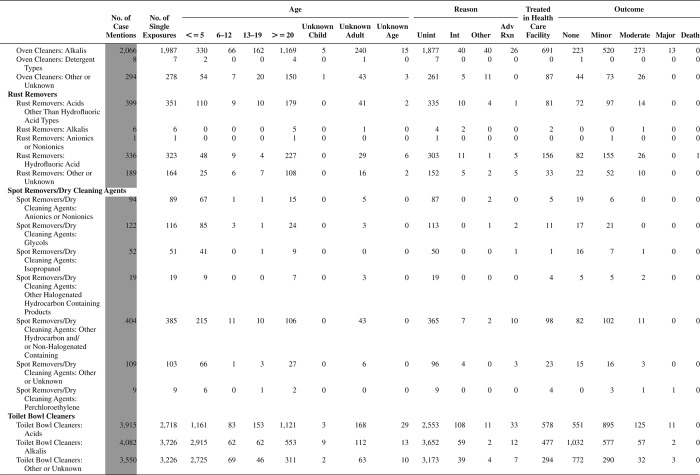


**Figure:**
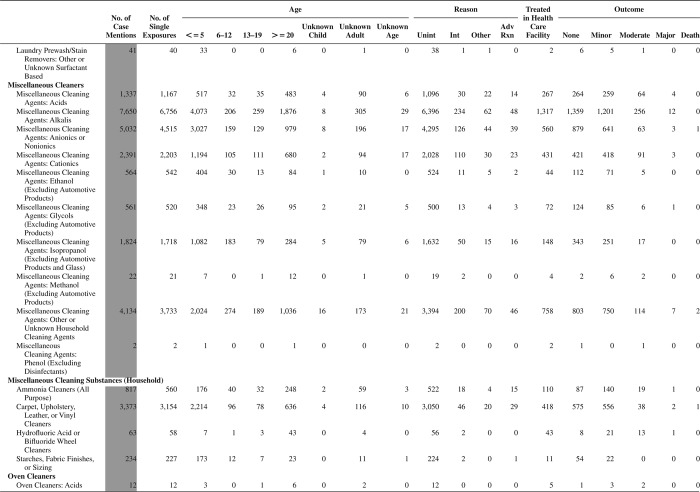


**Figure:**
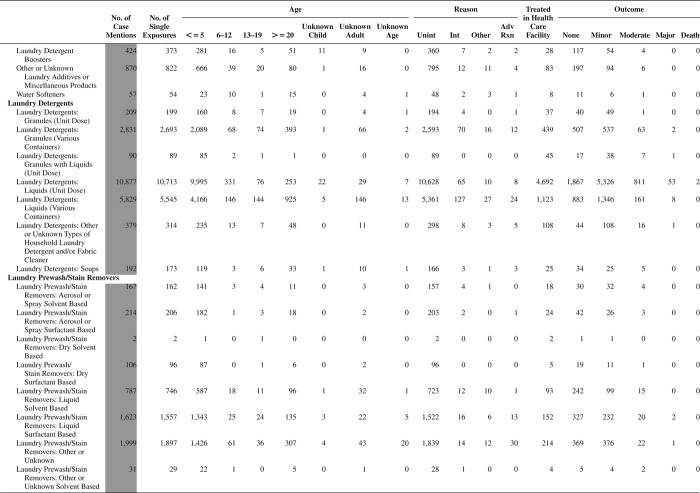


**Figure:**
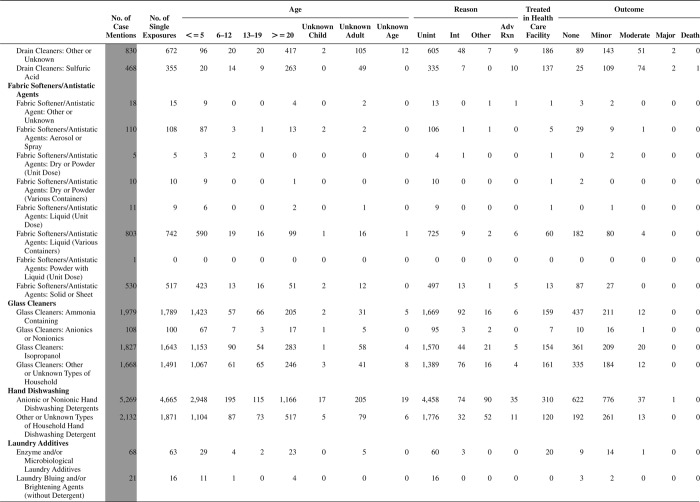


**Figure:**
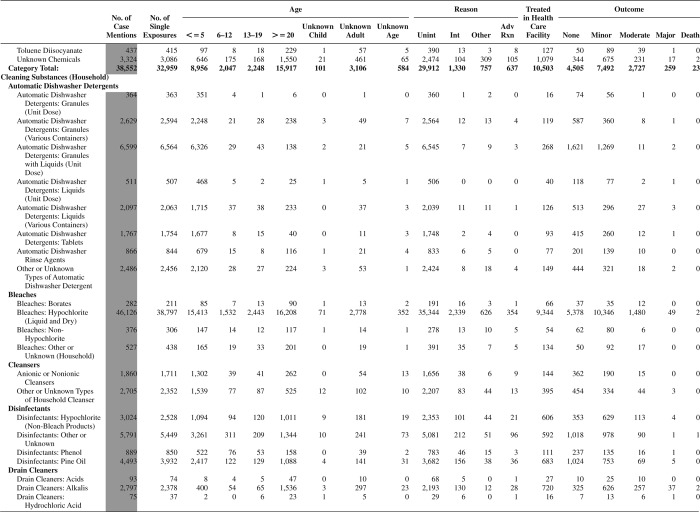


**Figure:**
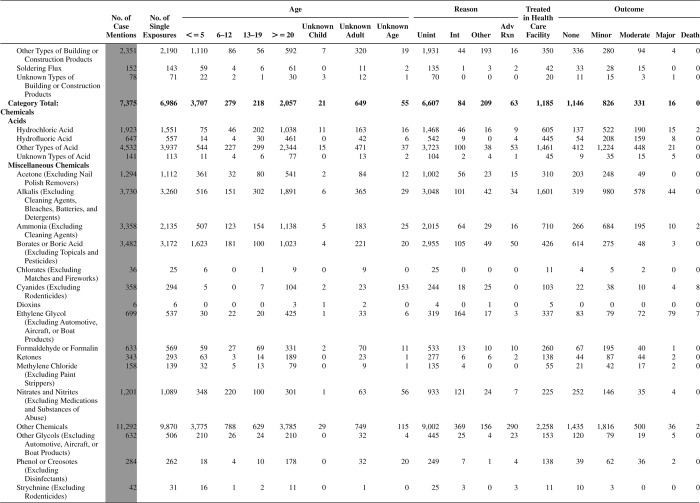


**Figure:**
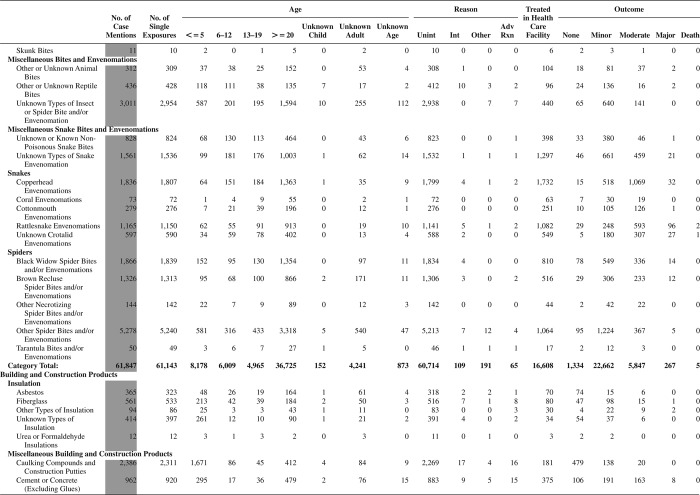


**Figure:**
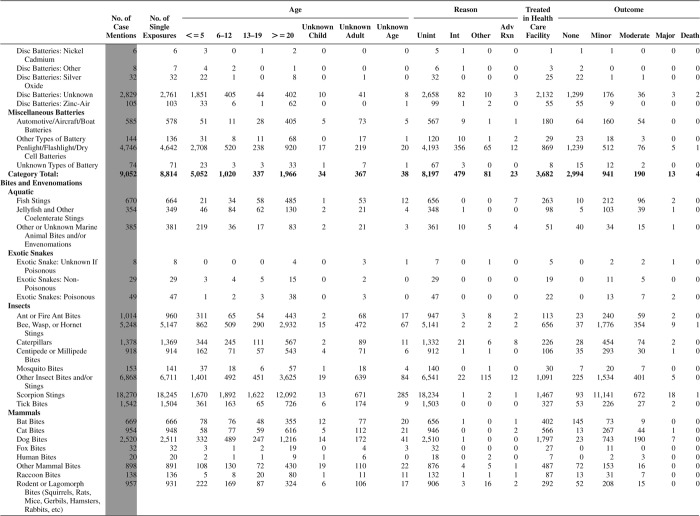


**Figure:**
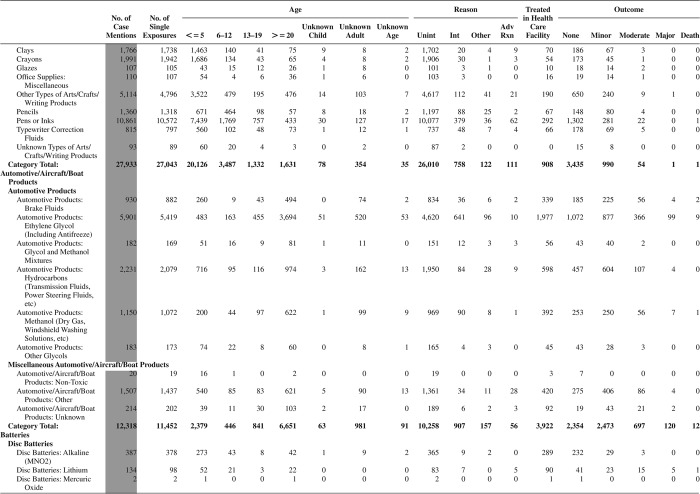


**Table 22B.**
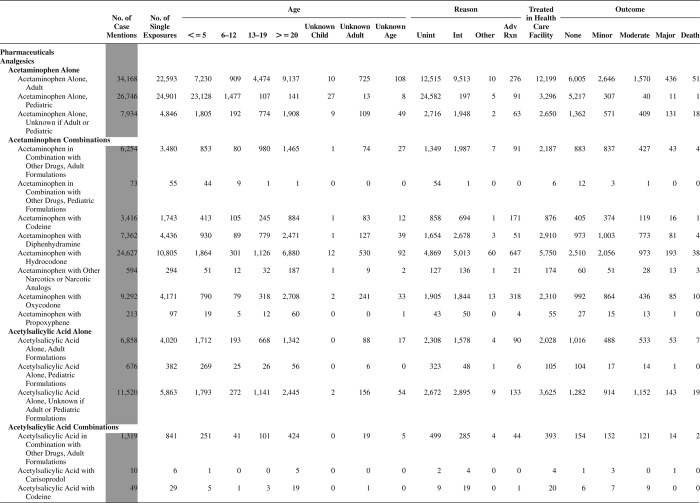
Demographic profile of SINGLE SUBSTANCE Pharmaceuticals exposure cases by generic category.

**Figure:**
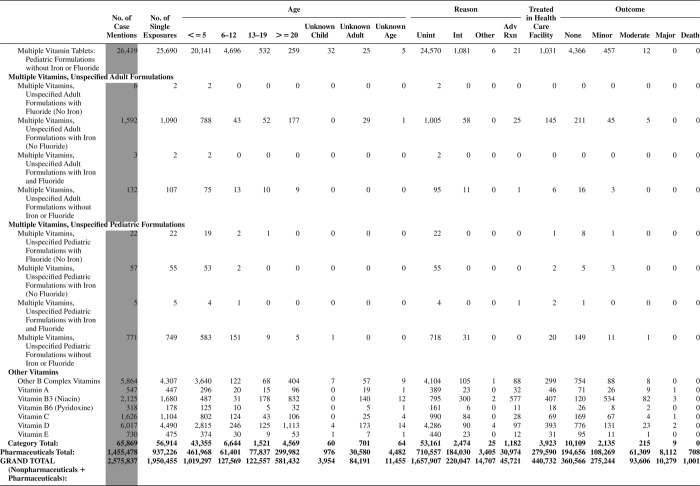


**Figure:**
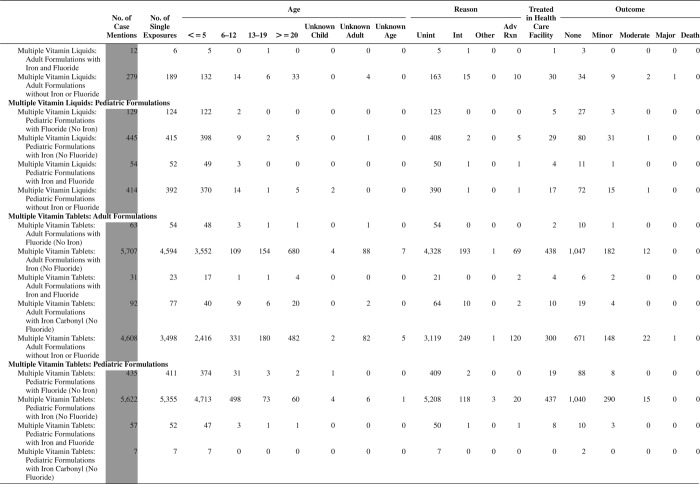


**Figure:**
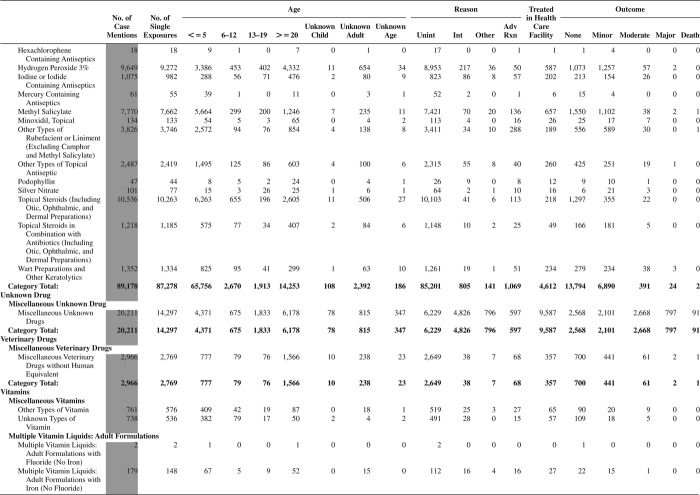


**Figure:**
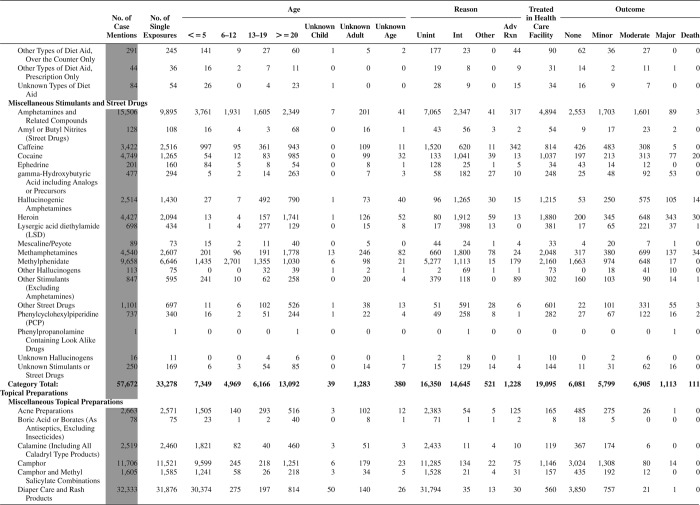


**Figure:**
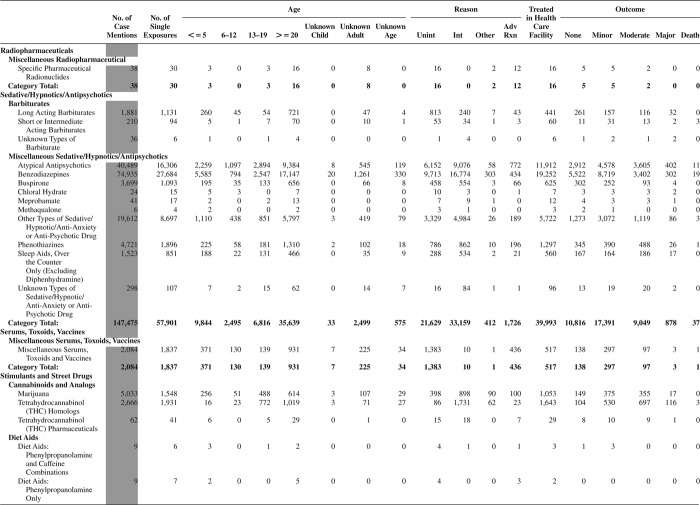


**Figure:**
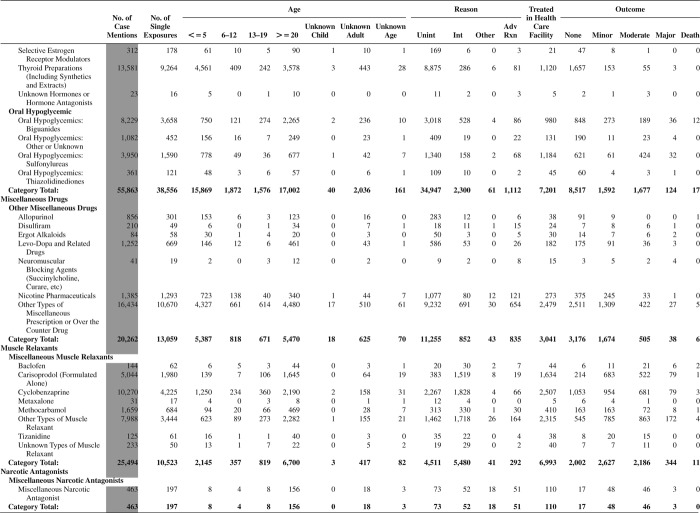


**Figure:**
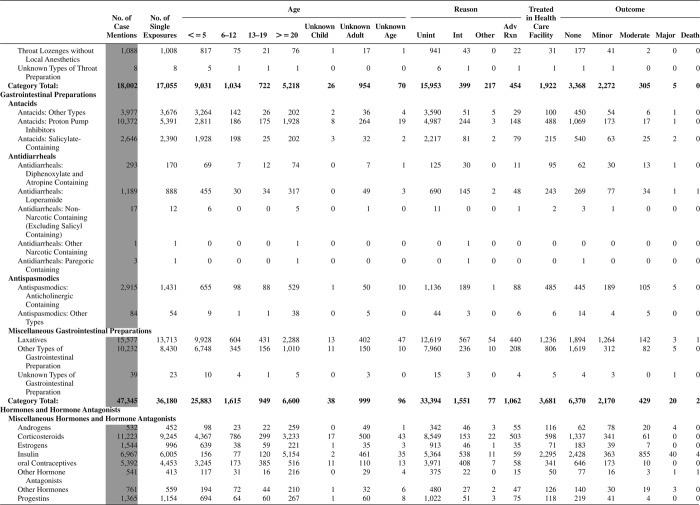


**Figure:**
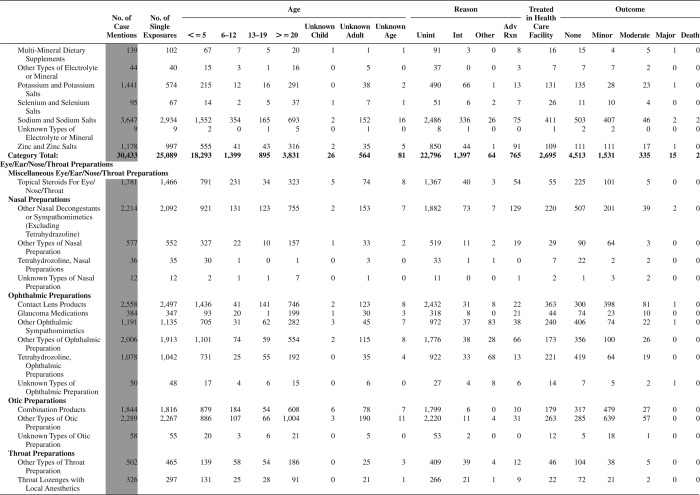


**Figure:**
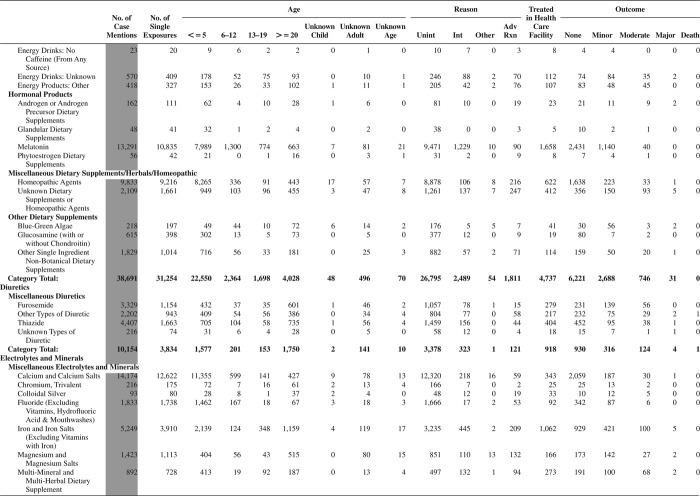


**Figure:**
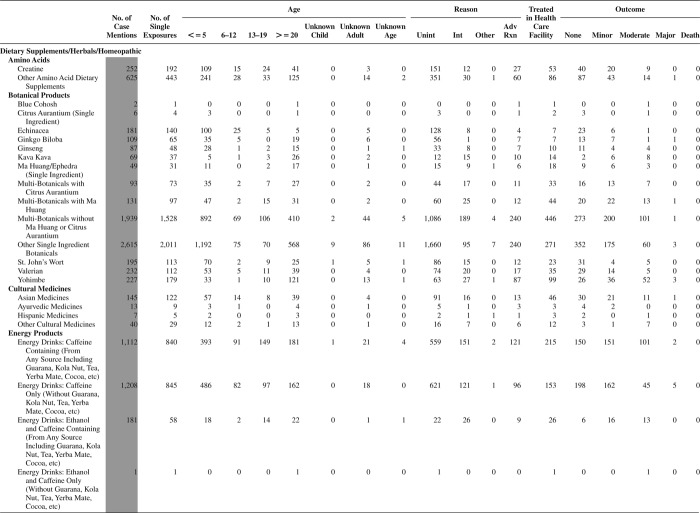


**Figure:**
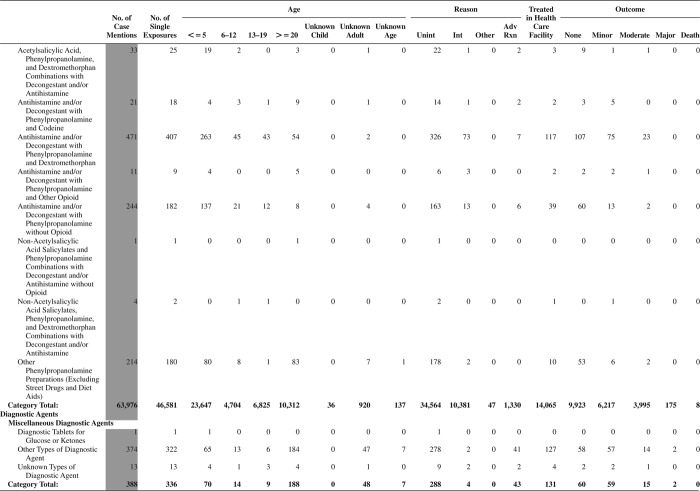


**Figure:**
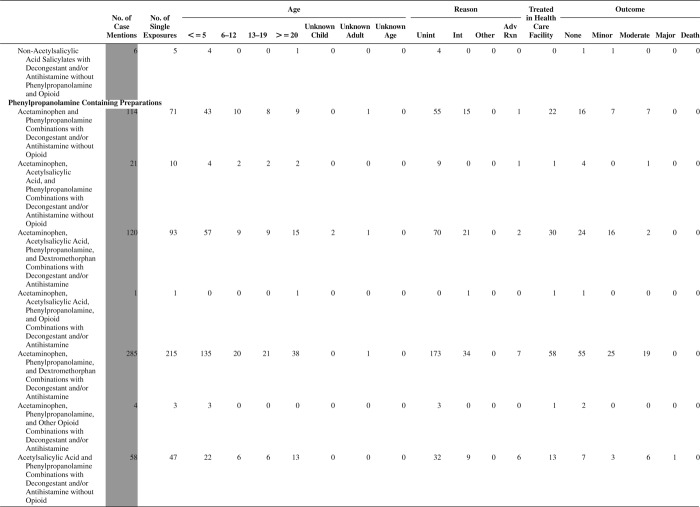


**Figure:**
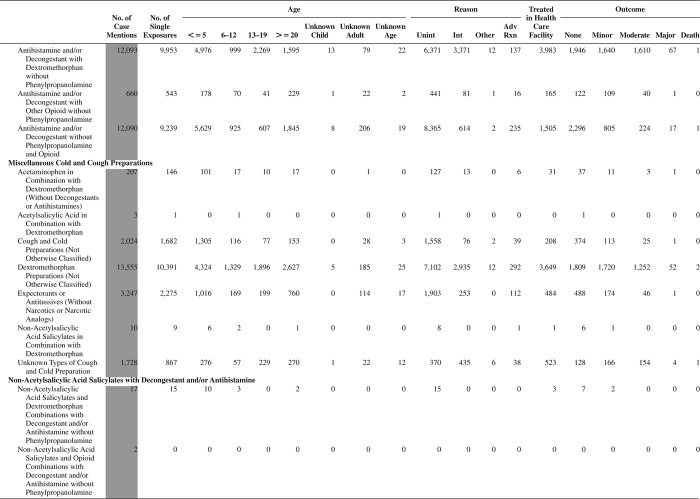


**Figure:**
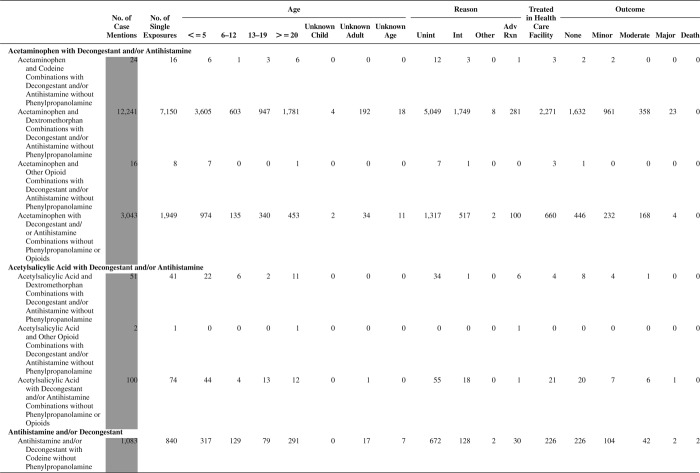


**Figure:**
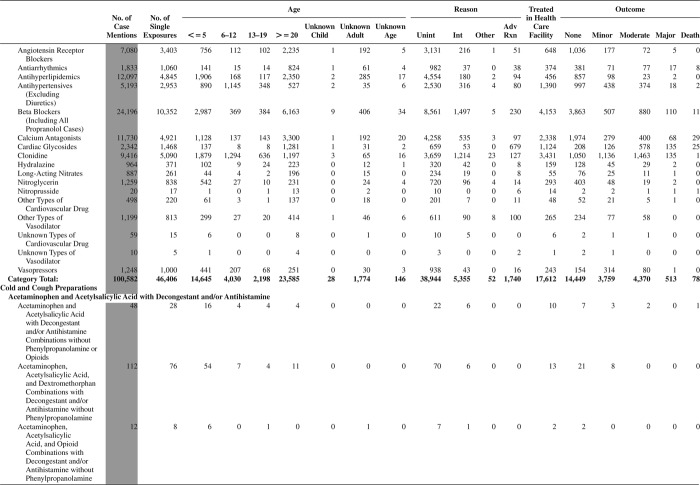


**Figure:**
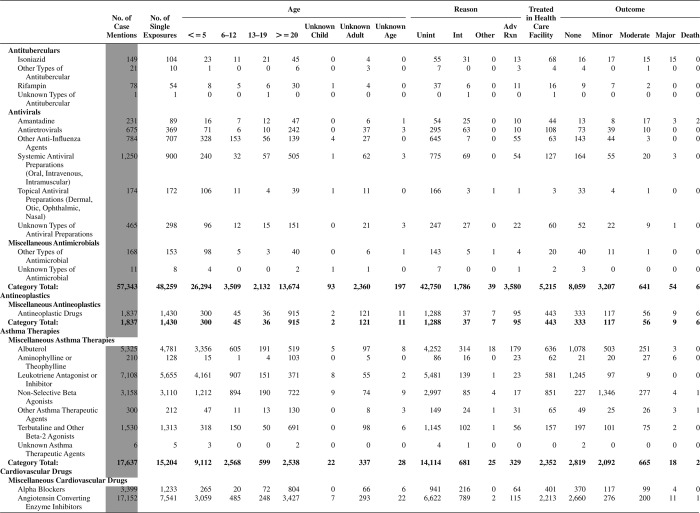


**Figure:**
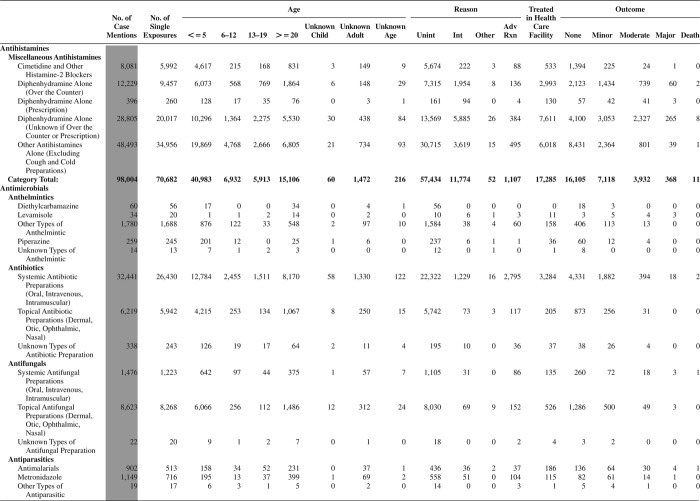


**Figure:**
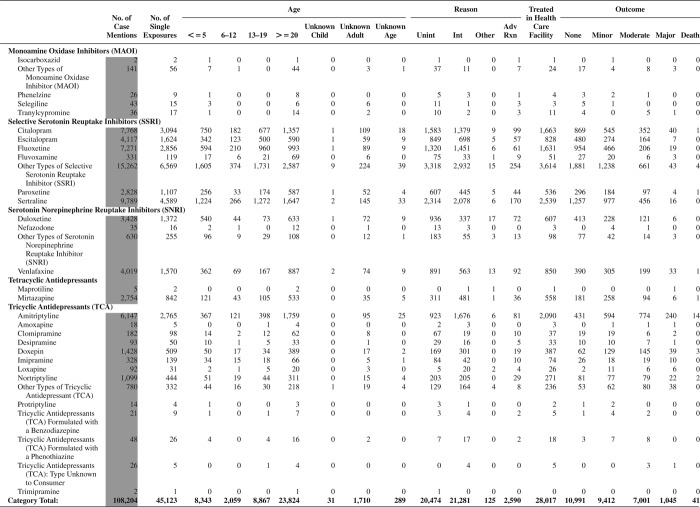


**Figure:**
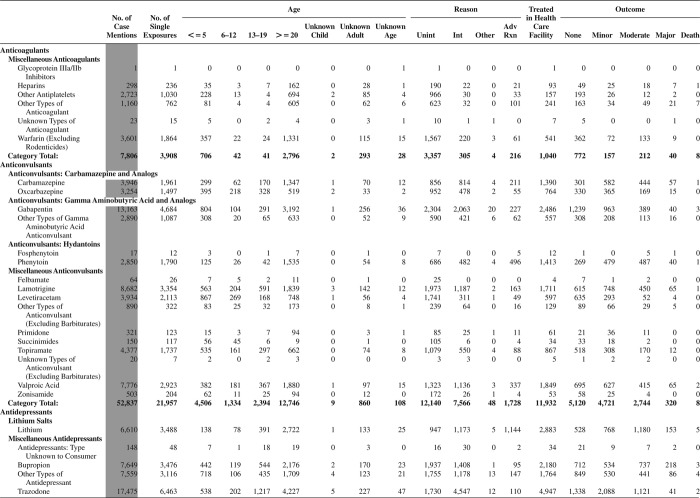


**Figure:**
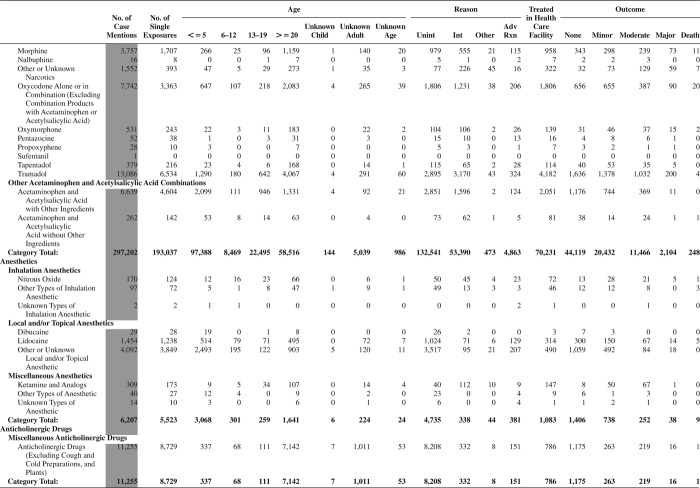


**Figure:**
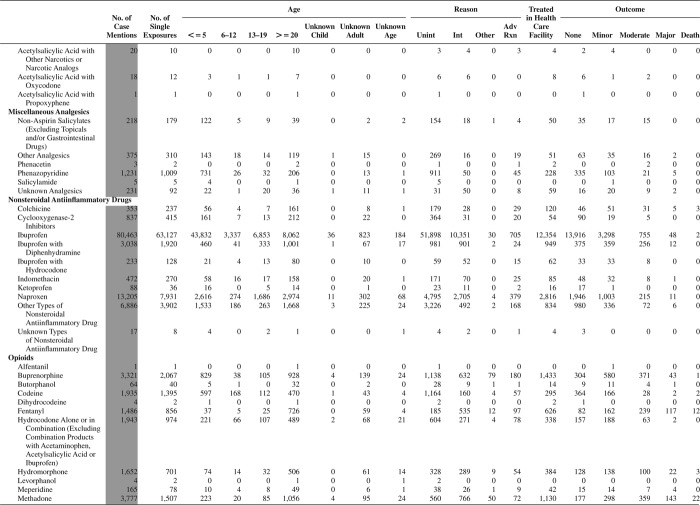


There were 925 deaths (indirect) and 1,552 deaths. Of these 2,477 cases, 2,113 were judged to be exposure-related fatalities (RCF = 1-Undoubtedly responsible, 2-Probably responsible, or 3-Contributory). The remaining 361 cases were judged as follows: 84 as RCF—probably not responsible; 34 as 5-clearly not responsible; and 246 as 6-unknown.

Deaths are sorted in Table 21 according to the category, substance deemed most likely responsible for the death (Cause Rank), and then patient age. The Cause Rank permits the PC to judge 2 or more substances as indistinguishable in terms of cause, for example, 2 substances which appear equally likely to have caused the death could have Substance Rank of 1, 2 and Cause Rank of 1, 1. Additional agents implicated are listed below the primary agent in the order of their contribution to the fatality.

As shown in Table 5, a single substance was implicated in 89.1% of reported human exposures, and 10.9% of patients were exposed to 2 or more drugs or products. The exposure-related fatalities involved a single substance in 538 cases (44.2%), 2 substances in 295 cases (24.2%), 3 in 152 cases (12.5%), and 4 or more in the balance of the cases.

In Table 21, the Annual Report ID number [bracketed] indicates that the abstract for that case is included in Appendix C. The letters following the Annual Report ID number indicate: i = Death, Indirect report (occurred in 895, 42.4% of cases), p = prehospital cardiac and/or respiratory arrest (occurred in 462 of 2,113, 21.9% of cases), h = hospital records reviewed (occurred in 497, 23.5% of cases), and a = autopsy report reviewed (occurred in 1,230, 58.2% of cases). The distribution of NPDS RCF was as follows: 1 = Undoubtedly responsible in 572 cases (27.1%), 2 = Probably responsible in 1,344 cases (63.6%), and 3 = Contributory in 197 cases (9.3%). The denominator for these Table 21 percentages is 2,113.

### All fatalities—all ages

Table 4 presents the age and gender distribution for these 1,218 exposure-related fatalities (excluding death, indirect). The age distribution of reported fatalities shows an increase in deaths in children (< 20 years old) compared with that of the past years, with 99 cases representing 8.1% of fatalities, an absolute increase of 26 child fatalities and a 35.6% increase in that age group. The age distribution of reported fatalities in adults (age, ≥ 20 years) is similar to that of prior years with 1,115 of 1,218 (91.5%) fatal cases occurring in that age group and 4 (0.3%) of fatalities occurring in unknown age patients. While children ≤ 5 years were involved in the majority of exposures, the 29 deaths in this group comprised just 2.4% of the exposure-related fatalities. However, it is noted that this represented a 38% increase in fatalities over 2012. While most (67.2%) of the fatalities occurred in 20- to 59-year-old individuals, the percentage is slightly decreased from prior years.

Table 21 lists each of the 2,113 human fatalities (including death, indirect report) along with all of the substances involved for each case. Please note that the substance listed in column 3 of Table 21 (alternate name) was chosen to be the most specific generic name based upon the Micromedex Poisindex product name and generic code selected for that substance. Alternate names are maintained in the NPDS for each substance involved in a fatality. The cross-references at the end of each major category section in Table 21 list all cases that identify this substance as other than the primary substance. This alternate name may not agree with the AAPCC generic categories used in the summary tables (including Table 22).

Table 18 lists the top 25 minor generic substance categories associated with reported fatalities and the number of single substance exposure fatalities for that category—miscellaneous sedative/hypnotics/antipsychotics, miscellaneous cardiovascular drugs, opioids, and miscellaneous stimulants and street drugs lead this list followed by miscellaneous alcohols, acetaminophen combinations, acetaminophen alone, selective serotonin reuptake inhibitors, and miscellaneous fumes/gases/vapors. Note that Table 18 is sorted by all substances to which a patient was exposed (i.e., a patient exposed to an opioid may have also been exposed to 1 or more other products) and shows single-substance exposures in the right-hand column.

The first-ranked substance (Table 21) was a pharmaceutical in 1,710 (80.9%) of the 2,113 fatalities. These 1,710 first-ranked pharmaceuticals included:

- 690 analgesics (110 acetaminophen/hydrocodone, 109 methadone,106 acetaminophen, 98 oxycodone, 58 morphine, 34 salicylate, 26 fentanyl, 23 tramadol, and 20 opioid)
- 414 stimulants/street drugs [255 heroin, 56 methamphetamine, 52 cocaine, and 15 amphetamines (hallucinogenic)]
- 174 cardiovascular drugs (30 verapamil, 28 amlodipine, 18 cardiac glycoside, 15 diltiazem, 16 metoprolol, 11 carvedilol, and 11 propranolol)
- 133 antidepressants (34 amitriptyline, 20 bupropion, 14 venlafaxine, 10 doxepin, 10 citalopram, and 8 lithium)
- 100 sedative/hypnotic/antipsychotics (23 alprazolam, 20 quetiapine, 7 zolpidem, 6 benzodiazepine, and 5 diazepam)

The exposure was acute in 1,183 (56.0%), A/C = acute on chronic in 282 (13.3%), C = chronic exposure in 98 (4.6%), and U = unknown in 550 (26.0%).

A total of 1,204 tissue concentrations for 1 or more related analytes were reported in 582 cases. Most of these (1,197) involved fatalities with RCF = 1–3, and are listed in Table 21, while all tissue concentrations are available to the member centers through the NPDS Enterprise Reports. These 128 analytes included the following: 234 acetaminophen, 94 ethanol, 73 salicylate, 52 carboxyhemoglobin, 34 morphine, 27 alprazolam, 26 digoxin, 25 diphenhydramine, 25 oxycodone, 22 hydrocodone, 22 lithium, 22 methadone, 19 benzoylecgonine, and 19 morphine (free).

Route of exposure was as follows: ingestion only in 1,322 cases (62.6%), inhalation/nasal in 135 cases (6.4%) and parenteral in 78 cases (3.7%). Most other routes were combination routes or unknown.

The intentional exposure reason was: abuse in 863 cases (40.8%), suspected suicide in 691 cases (32.7%), and misuse in 48 cases (2.3%). Unintentional exposure reason was: environmental in 90 cases (4.3%), therapeutic error in 37 cases (1.8%), and misuse in 6 cases (0.3%). Adverse drug reaction was the reason in 47 (2.2%).

### Pediatric fatalities—age ≤ 5 years

Although children younger than 6 years were involved in the majority of exposures, they comprised 51 of 2,477 (2.1%) of fatalities. These numbers are similar to those reported since 1985 (Table 19A, all RCFs and includes indirect deaths). Table 8 (RCF 1–3, excludes indirect deaths) shows the percentage fatalities in children ≤ 5 years related to total pediatric exposures was 29/1,049,475 = 0.00276%. By comparison, 1,115/833,563 = 0.13% of all adult exposures involved a fatality. Of these 29 pediatric fatalities, 24 (82.8%) were reported as unintentional and 3 (10.3%) were coded as resulting from malicious intent (Table 8).

The 33 fatalities in children ≤ 5 years in Table 21 (includes death, indirect reports, and RCF 1–3) included 14 pharmaceuticals and 19 nonpharmaceuticals. The first-ranked substances associated with these fatalities included smoke (9), disc battery (2), hydromorphone (2), methadone (2), amitriptyline (2), and 16 other substances (1 each).

### Pediatric fatalities—ages 6–12 years

In the age range 6–12 years, there were 6 reported fatalities, 4 of which were unintentional environmental, 1 was intentional suspected suicide, and 1 was intentional abuse (Table 8). The 11 fatalities listed in Table 21 (includes death, indirect reports, and RCF 1–3) included 7 smoke, 2 carbon monoxide, 1 freon, and 1 methadone.

### Adolescent fatalities—ages 13–19 years

In the age range of 13–19 years, there were 64 reported fatalities, an increase of 19 (42%) and included 57 intentional, 3 unintentional, 2 adverse reaction, and 2 unknown reason (Table 8). The 78 fatalities listed in Table 21 (includes death, indirect reports and RCF 1–3) included 67 pharmaceuticals and 11 nonpharmaceuticals. The first-ranked pharmaceuticals associated with these fatalities included heroin (4), acetaminophen (3), methadone (3), oxycodone (3), drug, unknown (3), acetaminophen/hydrocodone (2), diphenhydramine (2), metformin (2), alprazolam (2), quetiapine (2), amphetamine (hallucinogenic), 2C-E (2), methamphetamine (2), methylenedioxymethamphetamine (MDMA) (2), THC homolog (2), 4-acetoxy-N,N-dimethyltryptamine (2), amphetamine (2), amphetamine (hallucinogenic) (2) and the remainder with1 substance each. The first ranked nonpharmaceutical associated with these fatalities included: cyanide (3), carbon monoxide (2),ethanol (1), methanol (1), freon (1), substance (non-drug) unknown (1), aldicarb (1), and dinitrophenol (1).

### Pregnancy and Fatalities

A total of 31deaths of pregnant women have been reported from the years 2000 through 2013. The majority (27 of 31) were intentional exposures (misuse, abuse, or suspected suicide). There was 1 death in pregnant women reported to NPDS in 2013.

### AAPCC Surveillance Results

A key component of the NPDS surveillance system is the variety of monitoring tools available to the NPDS user community. In addition to AAPCC national surveillance definitions, 35 PCs utilize NPDS as part of their surveillance programs. The Centers for Disease Control and Prevention (CDC), 6 state health departments and 1 state police department run surveillance definitions in NPDS. Since Surveillance Anomaly 1, generated at 2:00 pm EDT on 17 September 2006, over 230,000 anomalies have been detected. More than 1,500 were confirmed as being of public health significance with PCs working collaboratively with their local and state health departments and in some instances the CDC on the public health issues identified.

At the time of this report, 353 surveillance definitions run continuously, monitoring case and clinical effects volume and a variety of case-based definitions from food poisoning to nerve agents. These definitions represent the surveillance work by many PCs, state health departments, the AAPCC, and the Health Studies Branch, Division of Environmental Hazards and Health Effects, National Center for Environmental Health, Centers for Disease Control and Prevention (CDC).

Automated surveillance continues to remain controversial as a viable methodology to detect the index case of a public health event. Uniform evaluation algorithms are not available to determine the optimal methodologies.(9) Less controversial is the benefit to situational awareness that NPDS can provide.(10) Typical NPDS surveillance data detects a response to an event rather than an event prediction. This aids in situational awareness and resilience during and after a public health event.

A current example of the involvement of the PC system and NPDS can be seen in the following. In January 2010, the AAPCC introduced two generic codes for electronic cigarettes (e-cigarettes): one for the e-cigarette delivery system and one for the liquid nicotine refills. As the amount of nicotine in e-cigarettes and their refills were not initially regulated by the Food and Drug Administration or any states, they could represent a unique poisoning hazard. As the refills were not required to be sold in child resistant containers, the potentially large amount of nicotine in these products (some containing over 100 mg/ml) could potentially produce serious toxicity in both adults and children, if inhaled, swallowed or spilled on the skin. And although flavored cigarettes have been banned by the FDA since September 2009, there were no restriction on e-cigarette flavorings. Flavors such as black cherry, café mocha, peanut butter cup, and ice cream potentially represent an additional attraction to children.

The first exposure to an e-cigarette product was noted in September 2010, with the first child exposure in November 2010. A gradual increase in the number of exposures occurred until the beginning of 2013 when a dramatic increase in the number of exposures to e-cigarettes and their refills was seen (Figure 6). The total number of nonpharmaceutical nicotine exposures has increased, driven primarily by exposures to e-cigarette products. E-cigarette exposure calls peaked in April 2014 and comprised 35% of all nicotine-related single exposure calls. In children, e-cigarettes now account for roughly 25% of exposures, while in other age groups, e-cigarettes exposures have surpassed other tobacco products and account for as many as 65% of exposures. E-cigarette exposures in children under age 5 have serious outcomes in only 1.9% of cases compared with 5.3% in other ages. A decline in exposures has been seen since April 2014, possibly reflecting increased scrutiny on e-cigarettes and increased state and local regulation. Please note that the data for 2014 are considered preliminary since the 2014 database is not locked.

**Figure 5.**
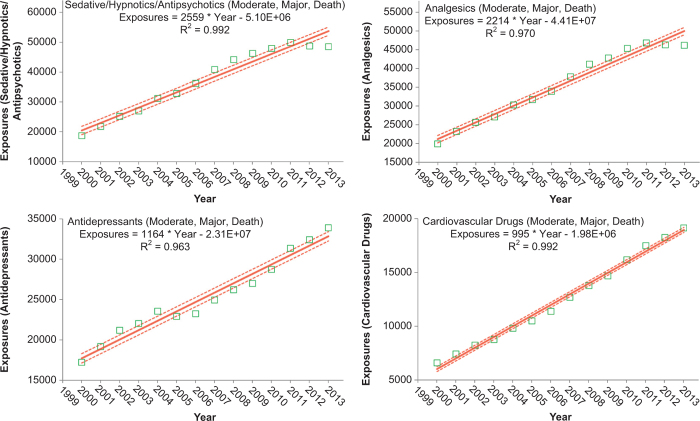
**Substance Categories with the Greatest Rate of More Serious Exposure Increase (Top 4)**. Solid lines show least-squares linear regressions for More Serious Human Exposure Calls per year for that category (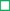). Broken lines show 95% confidence interval on the regression. More Serious Exposures include Medical Outcome of Moderate, Major and Death (colour version of this figure can be found in the online version at www.informahealthcare.com/ctx).

**Figure 6.**
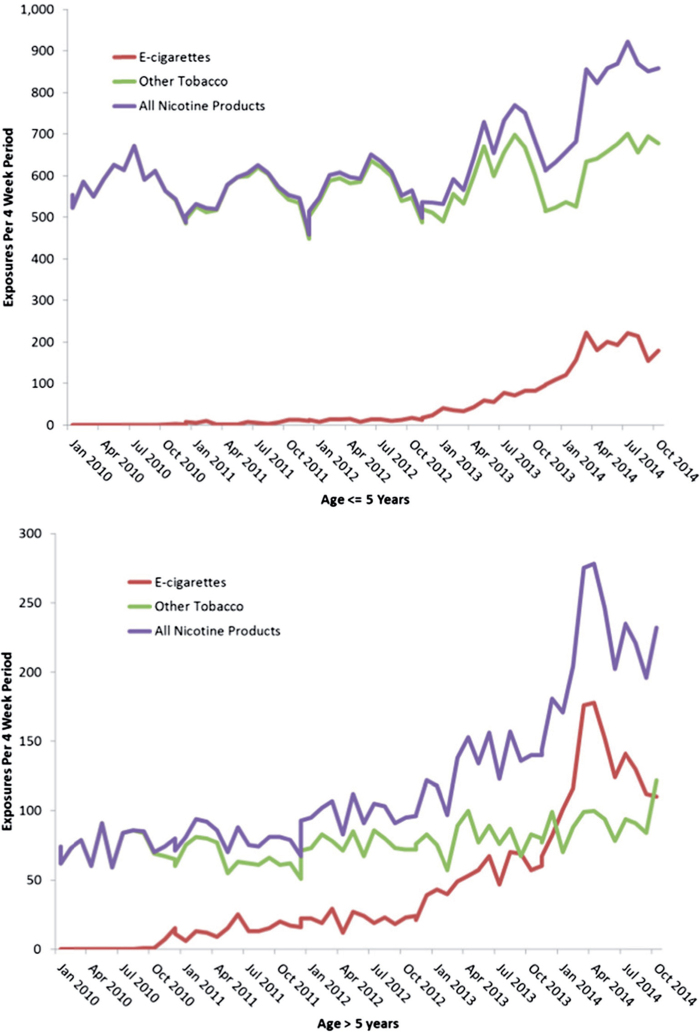
**E-cigarette product exposures, January 2010–October 2014.** The figures show the number of calls received per 4-week period by age group for single-substance human poison exposure calls to an e-cigarette device or refill (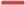 E-cigarette), traditional tobacco products such as cigarettes, snuff, and chewing tobacco (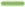 Other Tobacco) and the sum of the two groups (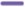 All Nicotine Products) since January 2010. Pharmaceutical nicotine products are excluded (colour version of this figure can be found in the online version at www.informahealthcare.com/ctx).

## Discussion

The exposure cases and information requests reported by PCs in 2013 do not reflect the full extent of PC efforts which also include poison prevention activities and public and health care professional education programs.

NPDS exposure data may be considered as providing “numerator data”, in the absence of a true denominator; that is, we do not know the number of actual exposures that occur in the population. NPDS data include only those exposures which are reported to PCs.

NPDS 2000–2013 call volume data clearly demonstrate a continuing decrease in total exposure calls. This decline has been apparent and increasing since mid-2007, and reflects the decreasing use of the PC for less severe exposures. However, in contrast, during this same period, exposures with a more severe outcome (death, major, moderate) and HCF calls have continued a consistent increase. Possible contributors to the declining PC access include declining US birth rates (especially since exposure rates are much higher in children ≤ 5 years of age), increasing use of text rather than voice communication, and increased use of and reliance on internet search engines and web resources. To meet our public health goals, PCs will need to understand and meet the public's 21st-century communication preferences. We are concerned that failure to respond to these changes may result in a retro-shift with more people seeking medical care for exposures that could have been managed at home by a PC. Likewise, minor exposures may progress to more severe morbidity and mortality because of incorrect internet information or no PC management. The net effect could be more severe poisoning outcomes because fewer people took advantage of PC services, with a resultant increased burden on the national health care infrastructure as may be reflected in the increased number of cases managed in a health care facility this year.

NPDS statistical analyses indicate that all analgesic exposures including opioids and sedatives are increasing year over year. This trend is shown in Table 17B and Figure 5. NPDS data mirror CDC data that demonstrates similar findings.(10) Thus, NPDS provides a real-time view of these public health issues without the need for data source extrapolations.

One of the limitations of NPDS data has been the perceived lack of fatality case volume compared with that of other reporting sources. However, when change over time is studied, NPDS is clearly consistent with other public health fatality analyses. One of the issues leading to this concern is the fact that medical record systems seldom have common output streams. This is particularly apparent with the various electronic medical record systems available. It is important to build a federated approach similar to the one modeled by NPDS to allow data sharing, for example, between hospital emergency departments and other medical record systems including medical examiner offices nationwide. Enhancements to NPDS can promote interoperability between NPDS and electronic medical records systems to better trend poison-related morbidity and mortality in the United States and internationally.

## Summary

Unintentional and intentional exposures continue to be a significant cause of morbidity and mortality in the United States. The near real-time, always current status of NPDS represents a national public health resource to collect and monitor US exposure cases and information calls.

Changes in encounters in 2013 shown in Figures 1, 3, and 4 include the following:

- total encounters (all exposure and information calls) decreased by 9.3%;
- all information calls decreased 21.4%, drug ID calls decreased 26.8%, and human exposures decreased 3.8%;
- HCF information calls decreased 8.5% and HCF exposures decreased 0.1% notwithstanding an overall steady increase since 2000;
- human exposures with less serious outcomes decreased 4.1%, while those with more serious outcomes (minor, moderate, major or death) increased 0.4% notwithstanding an overall 4.5% yearly increase since 2000;
- The categories of substance exposures in cases with more serious outcomes increasing most rapidly are as follows: sedative/hypnotics/antipsychotics, followed by analgesics, antidepressants, and cardiovascular drugs.

These data support the continued value of PC expertise and the need for specialized medical toxicology information to manage the more severe exposures, despite a decrease in calls involving less severe exposures. PCs must consider newer communication approaches that match current public communication patterns in addition to the traditional telephone calls.

The continuing mission of NPDS is to provide a nationwide infrastructure for public health surveillance for all types of exposures, public health event identification, resilience response, and situational awareness tracking. NPDS is a model system for the nation and global public health.

## Disclaimer

The American Association of Poison Control Centers (AAPCC; http://www.aapcc.org) maintains the national database of information logged by the country’s regional poison centers (PCs) serving all 50 United States, Puerto Rico, and the District of Columbia. Case records in this database are from self-reported calls: they reflect only information provided when the public or health care professionals report an actual or potential exposure to a substance (e.g., an ingestion, inhalation, or topical exposure), or request information/educational materials. Exposures do not necessarily represent a poisoning or overdose. The AAPCC is not able to completely verify the accuracy of every report made to member centers. Additional exposures may go unreported to PCs and data referenced from the AAPCC should not be construed to represent the complete incidence of national exposures to any substance(s).

## Appendix A – Acknowledgments

The compilation of the data presented in this report was supported in part through the US Centers for Disease Control and Prevention AAPCC Contract 200-2011-41767.

The authors wish to express their profound appreciation to the following individuals who assisted in the preparation of the manuscript: Katherine W. Worthen and Laura J. Rivers.

The authors express their sincere gratitude to the staff at the AAPCC Central Office for their support during the preparation of the manuscript: Stephen Kaminski, JD, Executive Director, Beth Copes and the entire staff.

### Poison Centers (PCs)

We gratefully acknowledge the extensive contributions of each participating PC and the assistance provided by the many health care providers who provided comprehensive data to the PCs for inclusion in this database. We especially acknowledge the dedicated efforts of the specialists in poison information (SPIs) who meticulously coded 3,060,122 calls made to US PCs in 2013.

As in previous years, the initial review of reported fatalities and development of the abstracts and case data for NPDS was the responsibility of the staff at the 57 participating PCs. Many individuals at each center participated in the fatality case preparation. These toxicology professionals and their centers are:

**Alabama Poison Center**

Perry Lovely, MD, ACMT

John Fisher, PharmD, DABAT, FAACT

Lois Dorough BSN, RN, CSPI

**Arizona Poison and Drug Information Center**

Keith Boesen, PharmD, CSPI

F. Mazda Shirazi, MS, MD, PhD, FACEP, FAMCT

**Arkansas Poison & Drug Information Center**

Henry F. Simmons, Jr., MD

Pamala R. Rossi, PharmD

Howell Foster, PharmD, DABAT

**Banner Good Samaritan Poison and Drug Information Center**

Daniel Brooks, MD

Frank LoVecchio, DO, MPH

Jane Klemens, RN, CSPI

Sharyn Welch, RN

Rebecca Hilder, RN, CSPI

Diane Glogan, RN

**Blue Ridge Poison Center**

Christopher P. Holstege, MD

Nathan P. Charlton, MD

William Rushton, MD

Luke Hardison, MD

**California Poison Control System—Fresno/Madera Division**

Richard J. Geller, MD, MPH

**California Poison Control System—Sacramento Division**

Timothy Albertson, MD, PhD

Justin Lewis, PharmD, CSPI

**California Poison Control System—San Diego Division**

Richard F. Clark, MD

Lee Cantrell, PharmD

Alicia B. Minns, MD

Janna Villano, MD

Charles O’Connell, MD

**California Poison Control System—San Francisco**

Suad A. Al-Abri, MD

Ilene Anderson, PharmD

Jo Ellen Dyer, PharmD

Hallam Gugelmann, MD

Sandra Hayashi, PharmD

Raymond Ho, PharmD

Susan Kim-Katz, PharmD

Beth Manning, PharmD

Kathryn Meier, PharmD

Kent R. Olson, MD

Freda Rowley, PharmD

Ben Tsutaoka, PharmD

**Carolinas Poison Center**

Michael C. Beuhler, MD

Anna Rouse Dulaney, PharmD

Christine M. Murphy, MD

William Kerns II, MD

**Central Ohio Poison Center**

Hannah Hays, MD

Marcel J. Casavant, MD, FACEP, FACMT

Henry Spiller, MS, DABAT, FAACT

Jason Russell, DO

Devin Wiles DO

Kaitlyn Day

**Central Texas Poison Center**

Ryan Morrissey, MD

S. David Baker, PharmD, DABAT

**Children's Hospital of MI Regional Poison Center**

Cynthia Aaron, MD

Lydia Baltarowich, MD

Aimee Nefcy, MD

Bram Dolcourt, MD

Susan C. Smolinske, PharmD

Matthew Hedge, MD

Andrew King, MD

Keenan Bora, MD

**Cincinnati Drug and Poison Information Center**

Shan Yin, MD, MPH

Sara Pinkston, RN

**Connecticut Poison Center**

Charles McKay, MD, ABMT

Mary Kay Balboni, RN, CSPI

Bernard C. Sangalli, MS, DABAT

**Florida/USVI Poison Information Center—Jacksonville**

Thomas Kunisaki, MD, FACEP, ACMT

**Florida Poison Information Center—Miami**

Jeffrey N. Bernstein, MD

Richard S. Weisman, PharmD

**Florida Poison Information Center—Tampa**

TamasPeredy, MD, FAACT

Charisse Webb, RN, CSPI

Aryne Patterson, RN, CSPI

Judy Turner, RN, CSPI

Pamela Eubank, RN, CSPI

**Georgia Poison Center**

Robert J. Geller, MD

Brent W. Morgan, MD

Ziad Kazzi, MD

Stella Wong, DO

Gaylord P. Lopez, PharmD

Stephanie Hon, PharmD

Adam Pomerleau, MD

Justin Arnold, DO

Alaina Steck, MD

Melissa Halliday, MD

Molly Boyd, MD

**Hennepin Regional Poison Center**

Deborah L. Anderson, PharmD

Jon B. Cole, MD

Katherine Katzung, MD

JoAn Laes, MD

Benjamin S. Orozco, MD

David J. Roberts, MD

Laurie Willhite, PharmD, CSPI

**Illinois Poison Center**

Michael Wahl, MD

Sean Bryant, MD

**Indiana Poison Center**

James B. Mowry, PharmD

Gwenn Christianson, MSN, CSPI

R. Brent Furbee, MD

**Iowa Poison Control Center**

Sue Ringling, RN

Linda B. Kalin, RN

Edward Bottei, MD

**Kentucky Regional Poison Control Center**

George M. Bosse, MD

Ashley N. Webb, MSc, PharmD, DABAT

**Louisiana Poison Center**

Mark Ryan, PharmD

Thomas Arnold, MD

**Maryland Poison Center**

Suzanne Doyon, MD, FACMT

Mingzohn (Ellen) Tsay, PharmD

**Mississippi Poison Control Center**

Robert Cox MD, PhD, DABT, FACMT

Christina Parker, RN, CSPI

**Missouri Poison Center at SSM Cardinal Glennon**

**Children's Medical Center**

Rebecca Tominack, MD

Shelly Enders, PharmD, CSPI

**National Capital Poison Center**

Cathleen Clancy, MD, FACMT

Nicole Reid, RN, BA, BSN, MEd, CSPI

**Nebraska Regional Poison Center**

Prashant Joshi, MD

Ronald I. Kirschner, MD

**New Jersey Poison Information and Education System**

Steven M. Marcus, MD

Bruce Ruck, PharmD

**New Mexico Poison and Drug Information Center**

Steven A. Seifert, MD, FAACT, FACMT

Brandon Warrick, MD

Susan Smolinske, PharmD, DABAT

**New York City Poison Control Center**

Maria Mercurio-Zappala, MS, RPh

Robert S. Hoffman, MD

Lewis Nelson, MD

Rana Biary, MD

Nicholas Connors, MD

Mai Takematsu, MD

Betty Chen, MD

Lauren Shawn, MD

Hong Kim, MD

**North Texas Poison Center**

Brett Roth MD, ACMT, FACMT

Melody Gardner, RN, MSN, MHA, CCRN

**Northern Ohio Poison Center**

Lawrence S. Quang, MD

Adrianne Grendzynski, RN, BSN, CSPI

Danielle Richardson, RN, BSN, CSPI

Susan Scruton, RN, BSN, CSPI

**Northern New England Poison Center**

Karen E. Simone, PharmD, DABAT, FAACT

**Oklahoma Poison Control Center**

William Banner, Jr., MD, PhD, ABMT

Scott Schaeffer, RPh, DABAT

**Oregon Poison Center**

Zane Horowitz, MD

Sandra L. Giffin, RN, MS

**Palmetto Poison Center**

William H. Richardson, MD

Jill E. Michels, PharmD

**Pittsburgh Poison Center**

Michael Lynch, MD

Rita Mrvos, BSN

**Puerto Rico Poison Center**

José Eric Dîaz-Alcalá, MD

Andrés Britt, MD

Elba Hernández, RN

**Regional Center for Poison Control and Prevention Serving Massachusetts and Rhode Island**

Michele M. Burns, MD, MPH

Dennis Wigandt, PharmD

Rebecca Bruccoleri, MD

Diana Felton, MD

**Regional Poison Control Center—Children's of Alabama**

Erica Liebelt, MD, FACMT

Michele Nichols, MD

Sherrel Kirkland, RN, CSPI

Ann Slattery DrPH DABAT

Diane Smith, RN, CSPI

**Rocky Mountain Poison & Drug Center**

Alvin C. Bronstein MD, FACEP

Beau Braden, DO, MPH

Dazhe Cao, MD

Janetta L. Iwanicki, MD

Eric J. Lavonas, MD

Sam Wang, MD

Shireen Banerjji, PharmD, DABAT

Carol Hesse RN, CSPI

Regina R. Padilla

**South Texas Poison Center**

Cynthia Abbott-Teter, PharmD

Douglas Cobb, RPh

Miguel C. Fernandez, MD

George Layton, MD

C. Lizette Villarreal, MA

**Southeast Texas Poison Center**

Wayne R. Snodgrass, MD, PhD, FACMT

Jon D. Thompson, MS, DABAT

Jean L. Cleary, PharmD, CSPI

**Tennessee Poison Center**

John G. Benitez, MD, MPH

Donna Seger, MD

**Texas Panhandle Poison Center**

Shu Shum, MD

Jeanie E. Jaramillo, PharmD

Cristie Johnston, RN, CSPI

**The Poison Control Center at the Children's Hospital of Philadelphia**

Fred Henretig, MD

Kevin Osterhoudt, MD

Jeanette Trella, PharmD

**University of Kansas Hospital Poison Control Center**

Tama Sawyer, PharmD, DABAT

Stephen Thornton, MD

**Upstate NY Poison Center**

Jeanna M. Marraffa, PharmD

Nicholas Nacca, MD

Rachel Schult, Pharm.D.

Christine M. Stork, PharmD

Ross Sullivan, MD

Timothy Wiegand, MD

**Utah Poison Control Center**

B. Zane Horowitz, MD

**Virginia Poison Center**

Rutherfoord Rose, PharmD

Kirk Cumpston, DO

Brandon Wills, DO

Michelle Troendle, MD

**Washington Poison Center**

William T. Hurley, MD, FACEP, FACMT

Curtis Elko, PharmD

David Serafin, CPIP

Tom Martin, MD, MPH, FACEP

**West Texas Regional Poison Center**

Hector L. Rivera, RPh, CSPI

Stephen W. Borron, MD, MS, FACEP, FACMT

Salvador H. Baeza, PharmD, DABAT

**West Virginia Poison Center**

Elizabeth J. Scharman, PharmD, DABAT, BCPS, FAACT

Anthony F. Pizon, MD, ABMT

**Wisconsin Poison Center**

David D. Gummin, MD

Amy E. Zosel, MD

### AAPCC Fatality Review Team

The Lead and Peer review of the 2013 fatalities was carried out by the 39 individuals listed here including four who reviewed the pediatric cases [Peds]. The authors and the AAPCC wish to express our appreciation for their volunteerism, dedication, hard work, and good will in completing this task in a limited time.

Alfred Aleguas Jr, PharmD, DABAT, Florida Poison Information Center- Tampa

Andy King, MD, Children’s Hospital of Michigan RPCC, Detroit

Amy Zosel, MD, Wisconsin Poison Center

Anna Rouse Dulaney*, PharmD, DABAT, Carolinas Poison Center

Ann-Jeannette Geib, MD, FACEP, FACMT, Assistant Professor of Emergency Medicine, Rutgers Robert Wood Johnson Medical School, New Brunswick, NJ

Ashley Webb, MSc, PharmD, DABAT, Director, Kentucky Regional PCC

Bernard C Sangalli*, MS, DABAT, Connecticut Poison Center

Charles McKay, MD, Associate Medical Director, Connecticut Poison Control Center, University of Connecticut School of Medicine

Curtis Elko*, PharmD, CSPI, Washington Poison Center, Seattle

Cynthia Lewis-Younger, MD, MPH, Vancouver, Washington

Daniel E Brooks*, MD, Banner Good Samaritan Medical Center, Phoenix

David D Gummin, MD, Wisconsin Poison Center

Diane Calello, MD, FAAP, FACMT, New Jersey Poison Information and Education System [Peds]

Elizabeth J Scharman, PharmD, DABAT, BCPS, FAACT, West Virginia Poison Center

Frank LoVecchio, DO, Banner Poison and Drug and Information Center, Phoenix, AZ

Gar Chan, MD, FACEM, Launceston General Hospital, Tasmania, Australia

Hannah Hays, MD, Central Ohio Poison Center, Columbus, OH

Henry Spiller*, MS, DABAT, FAACT, Central Ohio Poison Center, Columbus OH

Jan Scaglione*, PharmD, DABAT, Cincinnati Drug and Poison Information Center

Jeffrey S Fine, MD, NYU School of Medicine/Bellevue Hospital [Peds]

Jennifer Lowry, MD, Division of Clinical Pharmacology, Toxicology, and Therapeutic Innovations, Children’s Mercy Hospital, Kansas City, MO [Peds]

Jill E Michels, PharmD, DABAT, Managing Director, Palmetto Poison Center, SC

John McDonagh, MD, Hartford, CT

Kathy Hart, MD, Connecticut Poison Control Center

L Keith French, MD, Oregon Poison Center

Maria Mercurio-Zappala, RPh, MS, DABAT, FAACT, NYC PCC

Mark Su, MD, MPH, FACEP, FACMT, Director, New York City Poison Control Center, New York, NY

Mike Levine*, MD, Banner Good Samaritan Medical Center, Phoenix

Nathanael McKeown*, DO, Oregon Poison Center

Rachel Gorodetsky, PharmD, D’Youville College School of Pharmacy, University of Rochester Medical Center

Rais Vohra, MD, California Poison Control System, Fresno/Madera

Robert Goetz*, PharmD, DABAT, Cincinnati Drug and Poison Information Center

Shana Kusin, MD, Department of Emergency Medicine, OHSU

Sophia Sheikh MD, Department of Emergency Medicine, University of Florida College of Medicine-Jacksonville

Steven M Marcus*, MD, NJ Poison Information and Education System, NJ Medical School, of the School of Biomedical and Health Sciences of Rutgers University, The State University of NJ [Peds]

Susan Smolinske, PharmD, Children’s Hospital of Michigan RPCC, Detroit

Timothy Wiegand, MD, Director of Toxicology, University of Rochester, Medical Center and Strong Memorial Hospital; Consultant Toxicologist, SUNY Upstate Poison Center

Tom Martin, MD, Medical Director, Utah Poison Control Center

William Hurley, MD, Washington Poison Center, Seattle

* These reviewers further volunteered to read the top ranked 200 abstracts and judged to publish or omit each.

### AAPCC Micromedex Joint Coding Group

Chair: Elizabeth J. Scharman, Pharm.D., DABAT, BCPS, FAACT

Alvin C. Bronstein, MD, FACEP, FACMT

Rick Caldwell

Christina Davis, PharmD

Sandy Giffin, RN, MS

Kendra Grande, RPh

Katherine M. Hurlbut, MD

Wendy Klein-Schwartz, PharmD, MPH

Fiona McNaughton

Susan C. Smolinske, PharmD

### AAPCC Rapid Coding Team

Chair: Alvin C. Bronstein, MD, FACEP, FACMT

Elizabeth J. Scharman, Pharm.D., DABAT, BCPS, FAACT

Jay L. Schauben, PharmD, DABAT, FAACT

Susan C. Smolinske, PharmD

### AAPCC Surveillance Team

NPDS surveillance anomalies are analyzed daily by a team of 9 medical and clinical toxicologists working across the country in a distributed system. These dedicated professionals interface with the Health Studies Branch, National Center for Environmental Health, Centers for Disease Control and Prevention (HSB/NCEH/CDC) and the PCs on a regular basis to identify anomalies of public health significance and improve NPDS surveillance systems:

Alvin C. Bronstein, MD, FACEP, FACMT - Director

Alfred Aleguas, Pharm D, DABAT

S. David Baker, PharmD, DABAT

Douglas J. Borys, PharmD, DABAT

John Fisher, PharmD, DABAT, FAACT

Jeanna M. Marraffa, PharmD, DABAT

Maria Mercurio-Zappala, RPH, MS, DABAT, FAACT

Henry A. Spiller, MS, DABAT, FAACT

Richard G. Thomas, Pharm D, DABAT

### Regional Poison Center (PC) Fatality Awards

Each year the AAPCC and the Fatality Review team recognized several regional PCs for their extra effort in their preparation of fatality reports and prompt responses to reviewer queries during the review process. The awards were presented at the October 2014, North American Congress of Clinical Toxicology meeting in New Orleans, LA.

First Center to Complete all Cases (30-Dec 2013, last of their 17 cases)

West Virginia Poison Center (Charleston)

Largest Number with Autopsy Reports (44 of 73 cases) Carolinas Poison Center (Charlotte)

Highest Percentage with Autopsy Reports (88% of 8 cases) Oklahoma Poison Control Center (Oklahoma City)

Largest Number of INDIRECT cases (507 of 925 total cases reported for 2013)

Maryland Poison Center (Baltimore)

Highest Overall Quality of Reports (12.0 of possible 22 for 1 case)

Texas Panhandle Poison Center (Amarillo)

Greatest improvement in Overall Quality of Reports (7.67 increase from last year)

Texas Panhandle Poison Center (Amarillo)

Most Abstracts Published in last year's Annual report (12 of the 70 published narratives)

Carolinas Poison Center (Charlotte)

Most Helpful Regional Poison Center Staff (based on survey of AAPCC review team)

Carolinas Poison Center (Charlotte)

- - - Honorable Mention

Banner Poison Drug and information Center (Dan Brooks)

## Appendix B—Data Definitions

### Reason for Exposure

NPDS classifies all calls as either EXPOSURE (concern about an exposure to a substance) or INFORMATION (non-exposed human or animal). A call may provide information about one or more exposed person or animal (receptors).

Specialists in poison information (SPIs) coded the reasons for exposure reported by callers to PCs according to the following definitions:

Unintentional general: All unintentional exposures not otherwise defined below.

Environmental: Any passive, non-occupational exposure that results from contamination of air, water, or soil. Environmental exposures are usually caused by manmade contaminants.

Occupational: An exposure that occurs as a direct result of the person being on the job or in the workplace.

Therapeutic error: An unintentional deviation from a proper therapeutic regimen that results in the wrong dose, incorrect route of administration, administration to the wrong person, or administration of the wrong substance. Only exposures to medications or products used as medications are included. Drug interactions resulting from unintentional administration of drugs or foods which are known to interact are also included.

Unintentional misuse: Unintentional improper or incorrect use of a nonpharmaceutical substance. Unintentional misuse differs from intentional misuse in that the exposure was unplanned or not foreseen by the patient.

Bite/sting: All animal bites and stings, with or without envenomation, are included.

Food poisoning: Suspected or confirmed food poisoning; ingestion of food contaminated with microorganisms is included.

Unintentional unknown: An exposure determined to be unintentional, but the exact reason is unknown.

Suspected suicidal: An exposure resulting from the inappropriate use of a substance for reasons that are suspected to be self-destructive or manipulative.

Intentional misuse: An exposure resulting from the intentional improper or incorrect use.

Contaminant/tampering: The patient is an unintentional victim of a substance that has been adulterated (either maliciously or unintentionally) by the introduction of an undesirable substance.

Malicious: Patients who are victims of another person’s intent to harm them.

Withdrawal: Inquiry about or experiencing of symptoms from a decline in blood concentration of a pharmaceutical or other substance after discontinuing therapeutic use or abuse of that substance.

Adverse Reaction Drug: Unwanted effects due to an allergic, hypersensitivity, or idiosyncratic response to the active ingredient(s), inactive ingredient(s) or excipient of a drug, chemical, or other drug substance when the exposure involves the normal, prescribed, labeled or recommended use of the substance.

Adverse Reaction Food: Unwanted effects due to an allergic, hypersensitivity, or idiosyncratic response to a food substance.

Adverse Reaction Other: Unwanted effects due to an allergic, hypersensitivity, or idiosyncratic response to a substance other than drug or food.

Unknown Reason: Reason for the exposure cannot be determined or no other category is appropriate.

### Medical Outcome

No effect: The patient did not develop any signs or symptoms as a result of the exposure.

Minor effect: The patient developed some signs or symptoms as a result of the exposure, but they were minimally bothersome and generally resolved rapidly with no residual disability or disfigurement. A minor effect is often limited to the skin or mucus membranes (e.g., self-limited gastrointestinal symptoms, drowsiness, skin irritation, first-degree dermal burn, sinus tachycardia without hypotension, and transient cough).

Moderate effect: The patient exhibited signs or symptoms as a result of the exposure that were more pronounced, more prolonged, or more systemic in nature than minor symptoms. Usually, some form of treatment is indicated. Symptoms were not life-threatening, and the patient had no residual disability or disfigurement (e.g., corneal abrasion, acid-base disturbance, high fever, disorientation, hypotension that is rapidly responsive to treatment, and isolated brief seizures that respond readily to treatment).

Major effect: The patient exhibited signs or symptoms as a result of the exposure that were life-threatening or resulted in significant residual disability or disfigurement (e.g., repeated seizures or status epilepticus, respiratory compromise requiring intubation, ventricular tachycardia with hypotension, cardiac or respiratory arrest, esophageal stricture, and disseminated intravascular coagulation).

Death: The patient died as a result of the exposure or as a direct complication of the exposure.

Not followed, judged as a nontoxic exposure: No follow-up calls were made to determine the outcome of the exposure because the substance implicated was nontoxic, the amount implicated was insignificant, or the route of exposure was unlikely to result in a clinical effect.

Not followed, minimal clinical effects possible: No follow-up calls were made to determine the patient's outcome because the exposure was likely to result in only minimal toxicity of a trivial nature. (The patient was expected to experience no more than a minor effect.)

Unable to follow, judged as a potentially toxic exposure: The patient was lost to follow-up, refused follow-up, or was not followed, but the exposure was significant and may have resulted in a moderate, major, or fatal outcome. Unrelated effect: the exposure was probably not responsible for the effect.

Confirmed nonexposure: this outcome option was coded to designate cases where there was reliable and objective evidence that an exposure initially believed to have occurred, but actually never occurred (e.g., all missing pills are later located). All cases coded as confirmed nonexposure are excluded from this report.

Death, indirect report: Death, indirect report are deaths that the poison center acquired from medical examiner or media, but did not manage nor answer any questions about the death.

### Relative Contribution to Fatality (RCF)

The definitions used for the Relative Contribution to Fatality (RCF) classification by the Case Review Team (CRT) were as follows:

**Undoubtedly responsible**—In the opinion of the CRT, the clinical case evidence establishes beyond a reasonable doubt that the substances actually caused the death.

**Probably responsible**—In the opinion of the CRT, the clinical case evidence suggests that the substances caused the death, but some reasonable doubt remained.

**Contributory** —In the opinion of the CRT, the clinical case evidence establishes that the substances contributed to the death, but did not solely cause the death. That is, the substances alone would not have caused the death, but combined with other factors, were partially responsible for the death.

**Probably not responsible**—In the opinion of the CRT, the clinical case evidence establishes to a reasonable probability, but not conclusively, that the substances associated with the death did not cause the death

**Clearly not responsible**—In the opinion of the CRT, the clinical case evidence establishes beyond a reasonable doubt that the substances”did not cause this death.

**Unknown**—In the opinion of the CRT, the clinical case evidence is insufficient to impute or refute a causative relationship for the substances in this death.

## Appendix C—Abstracts of Selected Cases

### Selection of Abstracts for Publication

The abstracts included in Appendix C were selected for publication in a three-stage process consisting of qualifying, ranking, and reading. Qualifying was based on the RCF:—only RCF = 1—Undoubtedly Responsible; 2—Probably Responsible; or 3—Contributory were eligible for publication. Fatalities by indirect report were excluded beginning with the 2008 annual report. Ranking was based on the number of substances (1/N) and weighted case score. The case weighting factors were the averages chosen based on review team recommendations in 2006. Each case score was multiplied by the respective factors to obtain a weighted publication score: Hospital records * 8.8 + Postmortem * 15.2 + Blood levels * 6.9 + Quality/Completeness * 6.4 + Novelty/Educational value * 13.2. Scores were normalized (z-score) within each reviewer before the final weighting: 25% for Age Z-Score + 25% for Freq Z-Score of 1st cause rank substance + 25% for weighted case scores + 25% for 1/N + 10 for pregnant patient + 10 for patient under 3 years old.

The top-ranked abstracts (200 + ties) were each read by individual reviewers (see Appendix A) and the 2 managers (Cantilena and Spyker). Each reader judged each abstract as “publish” or “omit,” and all abstracts receiving 7 or more of 12 publish votes were selected, further edited and cross-reviewed by the two managers.

### Abstracts

Abstracts of the cases were selected (see Selection of Abstracts for Publication, above) from the human fatalities judged related to an exposure as reported to US PCs in 2013. A structured format for abstracts was required in the PC preparation of the abstracts and was used in the abstracts presented. Abbreviations, units, and normal ranges omitted from the abstracts are given at the end of this appendix.

**Case 1.** Acute methanol ingestion: undoubtedly responsible.

**Scenario/Substances:** A 17-year-old (y/o) female with no significant past medical history presented to a community hospital with shortness of breath preceded by fatigue. She developed status epilepticus unresponsive to midazolam and required endotracheal intubation. She was transferred to a tertiary care hospital.

**Physical Exam:** BP 127/78, HR 113, RR 56, T 36°C, O_2_ sat 95% on room air. She was alert and interactive, appeared dehydrated and cachectic. Severely tachypneic. Globally weak. Otherwise remainder of examination was unremarkable.

**Laboratory Data:** pH 6.8 / pCO_2_ 10 / HCO_3_ 2

lactate 2.2 mmol/L, WBC 18, Hgb 17, platelets 345. Non-contrast head CT mild cerebral edema. Ammonia 163 mcmol/L, AST, ALT, and bilirubin normal. Serum valproate not detected. Methanol 45 mg/dL, 20 h after presentation and after 3.5 h of CRRT. Lumbar puncture was unrevealing.

**Clinical Course:** Patient became progressively more hypotensive despite IV fluid resuscitation, sodium bicarbonate infusion and three vasopressors. ECMO and CRRT were initiated. Metabolic service was consulted for persistent hyperammonemia and initiated a workup for late presenting inborn error of metabolism. Patient was given cobalamin, thiamine, biotin, levocarnitine, and riboflavin. Toxicology service was then consulted for unresolving metabolic acidosis despite resuscitation and bicarbonate infusion. Patient was given fomepizole. Metabolic acidosis resolved with CRRT. However, the patient's cerebral edema worsened, progressing to uncial herniation. Based on the prognosis, the family opted for institution of comfort measures and she expired. Following her death, police investigation revealed that the patient had conducted internet search on methanol poisoning. Multiple empty bottles of windshield wiper fluid containing methanol were found at the patient's home and car.

**Autopsy Findings:** Numerous linear scars on the body were consistent with self-destructive behavior. Other gross and microscopic pathology results were unremarkable. Cause of death was methanol intoxication. Manner of death was suicide.

**Case 148.** Acute ethylene glycol (antifreeze) per feeding tube: undoubtedly responsible.

**Scenario/Substances:** A 66 y/o male reportedly instilled 100 mL of antifreeze into his GI tract via tube feeding port ∼2 h prior to arrival in ED.

**Past Medical History:** Throat cancer, human immunodeficiency virus infection.

**Laboratory Data:** Venous blood gases upon arrival in ED pH 7.42/pCO_2_ 31/pO_2_ 35/HCO_3_ 20/BE -4. Hour 5: Na 147, Cl 107, CO_2_ 20, Glu 153, BUN 17, Cr 0.7, anion gap 23, lactate 1.73 mmol/L, HCO_3_ 17, BE 4, O_2_ sat 70%. Hour 18: pH 7.55 / pCO_2_ 16 / HCO_3_ 14, anion gap 19, salicylate not detected.

**Clinical Course:** Upon arrival in ED, he was tachypneic (RR 40), BP 146/84, HR 113. Fomepizole therapy was initiated, and thiamine was administered. The patient was admitted to the ICU Hour 5. Based on the prognosis and prior history, the family opted for institution of comfort measures. Fomepizole therapy was discontinued and he expired on Day 4.

**Autopsy Findings:** Hour 6 hospital blood ethylene glycol was 1,200 mg/dL. An autopsy was not performed. Probably cause of death: ethylene glycol toxicity due to antifreeze ingestion. Manner of death: Suicide.

**Case 153.** Acute disc battery and acetaminophen ingestion: undoubtedly responsible.

**Scenario/Substances:** A 16 m/o male was brought to the ED after a week of cough. Supratherapeutic doses of acetaminophen may have been given. An X-ray showed a 20-mm coin cell-shaped foreign body in the esophagus.

**Past Medical History:** Previously healthy.

**Clinical Course:** The child was transferred to a tertiary care hospital for endoscopic removal. The battery was successfully removed, and the child was admitted to the ICU. The child developed a massive GI bleed, liver failure, acidosis, and renal failure. He was intubated, sedated, and ventilated; N-acetylcysteine and blood products were administered. The child was taken to the OR where he arrested during exploratory laparotomy. CPR was initially successful, but the child remained hypoxic and hypotensive and died.

**Autopsy Findings:** Not available.

**Case 154**. Acute scorpion sting: undoubtedly responsible.

**Scenario/Substances:** A 3 y/o boy awoke at home, crying and complaining of ear pain, and was brought to the ED.

**Laboratory Data:** Initial labs at transferred hospital in PICU,

**Clinical Course:** Patient arrived at ED talking and answering questions, but rapidly developed a grade IV scorpion envenomation with crying, excessive secretions, opsoclonus, writhing, and tachycardia. He was receiving sedatives and analgesia when he developed respiratory distress and arrested. He was intubated and treated with atropine, epinephrine, flumazenil, bicarbonate. He received five vials of scorpion antivenin post code, was intubated, transferred to a tertiary care hospital, and admitted to the PICU. Lungs were clear and he exhibited posturing. Na 146 Cl, 114, lactate 2.9, AST 221 ALT 81, CK 922, ABG (capillary)-pH 7.48/pCO_2_ 26.7/pO_2_ 61.0/HCO_3_ 19.9/BE -4.0. Repeat Venous BG-pH 7.29/pCO_2_ 36/pO_2_ 49/HCO_3_ 17/BE -10.0 on FIO_2_ 45%. CxR “normal.” He was given naloxone to rule out over sedation. Pupils were fixed and dilated, and no response panting between ventilator breaths. No other medical or genetic abnormalities were found. Patient expired on Day 2 of suspected cerebral edema.

**Autopsy Findings: “**Complications of probable scorpion sting.” Femoral blood: tryptase 3.6 ng/mL.

**Case 155**. Acute crotalid envenomation: undoubtedly responsible.

**Scenario/Substances:** A 53 y/o 57 kg male was bitten while attempting to cut the rattle off a rattlesnake, which he presumed was dead. He developed an anaphylactic reaction with cardiopulmonary arrest. He was unresponsive to CPR measures including cardioversion, was intubated, and ventilated.

**Physical Exam:** After resuscitation HR 110, BP 94/50, he had an edematous right hand with three puncture marks.

**Laboratory Data:** 5 h post bite: Na 145, K 4.1, CO_2_ 17, Glu 41, WBC 37, Hgb 19.4, Hct 58, platelets 268, CK 9,196, Cr 1.6, BUN 7, AST 2,018, ALT 1,031, Alk phos 225, troponin 4.3, albumin 2.8 g/dL, D-dimer > 20. 6.5 h post exposure, Glu 109, fibrinogen 30 mg/dL, INR 2.1, PTT 47, CK 5,000. Day 2: WBC 23.7, Hgb 15. platelets 131, INR 2.8, PTT 56.3, fibrinogen 104 mg/dL, Cr 3.8, AST 2,275, ALT 800. Day 3: WBC 18, Platelets 58, Hgb 13.9, Hct 40.8, Cr 3.2, BUN 32, INR 1.9, PTT 44, CK 5176, fibrinogen 367 mg/dL. Day 4: WBC 4.7, Platelets 42, Hgb 13.6, Hct 38.7, Cr 3.3, BUN 31, INR 1.4, PTT149, fibrinogen 564 mg/dL, AST 2,797, ALT 1,972.

**Clinical Course:** He was given dopamine, 6 vials of antivenin (Fab fragment), tetanus toxoid, epinephrine, methylprednisolone, and diphenhydramine. He was transferred to a tertiary care hospital and admitted to the ICU 3 h post exposure. He was ventilated with FiO_2_ 100% + PEEP 5 with no pupil response. Bite site slightly swollen with no apparent progression. At 20 h post bite (14 vials of antivenin) he remained on the ventilator, receiving norepinephrine IV. Pupils were pinpoint and nonreactive. The affected hand measured 19.5 cm, was ecchymotic and blistering. By 24 h post bite (26 vials antivenin), HR 123 and BP 115/63, a femoral catheter was placed and dialysis started for acute kidney injury. On Day 3 (34 vials of antivenin), there were no neurological changes. On Day 4, his entire body was mottled, and he was purple from his nipple line up. The affected arm was ecchymotic and blistered up to his bicep. Right pupil was 3 mm and left pupil 4 mm and non-reactive. EEG showed “severe brain damage”, gag reflex was absent, and he had negative dolls eye reflex. He was receiving multiple vasopressors and IV NS. On Day 5, based on the prognosis, the family opted for institution of comfort measures and he expired later that day.

**Autopsy Findings:** Not performed.

**Case 161.** Acute cyanide exposure: undoubtedly responsible.

**Scenario/Substances: A** 19 y/o male purchased several grams of NaCN and KCN salts online, collapsed at home, EMS intubated, and was transported to the ED.

**Past Medical History:** Asperger's syndrome, depression, previous suicide attempt with chloroform.

**Laboratory Data:** ABG-pH 6.91/pCO_2_ 38/pO_2_ 153/HCO_3_ 7/BE 26, WBC 20.5, Hgb 20.4, Hct 63.4, platelets 314

anion gap 25, INR 1.48, lactate 20, serum acetaminophen and salicylate not detected, lithium 0.2 mmol/L, digoxin 0.2 ng/mL, UDS negative. Serum CN ∼10 mg/L (potentially toxic > 0.5 mg/L), 1.3 mg/L (thought drawn after first dose of hydroxocobalamin).

**Clinical Course:** On arrival in the ED, he was unresponsive, GCS 3, pupils midrange and fixed. He was reintubated, remained profoundly tachycardic and hypotensive despite maximum doses of norepinephrine and dopamine. Further history from family disclosed that patient's access to cyanide salts. Initial labs were notable for profound metabolic acidosis with markedly elevated lactate. ECG showed nonspecific intra ventricular conduction delay with QRS 120 which was improved to 94–100 after sodium bicarbonate. He received hydroxocobalamin 5g x3 doses total, with repeat BP improved from systolic 40 to 60 to 70-80 then to 180–200 after third dose. HR increased to 180s after 3rd dose of hydroxocobalamin. Repeat labs showed slight improvement in acidosis and lactate; however hypotension recurred requiring a 4th dose of hydroxocobalamin with minimal improvement. Head CT showed diffuse subarachnoid hemorrhage, poorly differentiated gray-white matter with global effacement consistent with anoxic encephalopathy, and hypoxic ischemic injury. Based on the prognosis, the family opted for institution of comfort measures and he expired on Day 1.

**Autopsy Findings:** External exam and laboratory evaluation performed only due to family's religious wishes. Lumbar tap with bloody CSF with RBCs settling and residual maroon CSF. Ante mortem blood prior to hydroxocobalamin treatment screened positive for CN (∼10 mcg/mL, reporting limit 0.3 mcg/mL). Cause of death: hypoxic encephalopathy and possible subarachnoid hemorrhage complicating acute cyanide toxicity. The manner of death was suicide.

**Case 171.** Acute ammonia inhalation and ocular: contributory.

**Scenario/Substances: A** 45 y/o male was driving a semi-truck carrying anhydrous ammonia that collided with a train. There was no damage to the cab and he was alert, but soon experienced difficulty breathing. EMS found him in respiratory distress with confusion, intubated him, noted vocal cord edema, and transported him to the ED.

**Physical Exam:** In the ED, bilateral scleral and conjunctival injection, erythematous eyelids, pupils equal and reactive to light, moist oral mucosa, diminished lung sounds in right base with occasional expiratory wheezes, extremities: 1–2 + edema of right lower extremity with trace lower extremity edema on the left. BP 135/63, O_2_ sat 98% on 100% FiO_2_, T 36°C.

**Laboratory Data:** ABG-pH7.11 / pCO_2_ 82 / pO_2_ 299 / HCO_3_ 26.5, WBC 22.3, CO_2_ 19.6

**Clinical Course:** He was admitted to the ICU, eyes copiously irrigated, and ophthalmology examination completed. He was maintained on mechanical ventilation, and CxR showed bibasilar infiltrates; he received prophylactic antibiotics for presumed aspiration pneumonia. Respiratory status improved, and he was weaned from ventilator on the morning of Day 5. Later on that day, he developed increasing dyspnea, bradycardia with a decline in O_2_ sats that were unresponsive to supplemental O_2._ A code was called, the patient re-intubated, but had ventilator asynchrony and was difficult to ventilate. He became tachycardic, was on maximal IV propofol and midazolam when he had a pulmonary embolism and was suspected despite prophylactic heparin administration. Prior to obtaining a CT of the chest, he had a bradycardic episode, unresponsive to atropine, which quickly became a PEA arrest. He underwent ACLS resuscitation for 40 minutes without return of circulation. He expired on Day 6.

**Autopsy Findings:** Not performed per family.

**Case 185.** Acute cyanide ingestion: undoubtedly responsible.

**Scenario/Substances:** A 73 y/o male jeweler presented to the ED with his wife via private vehicle.

**Past Medical History:** CAD, s/p CABG and pacemaker placement.

**Laboratory Data:** ABG-pH 7.32 / pCO_2_ 18 / pO_2_ 453 / HCO_3_ 17 / BE 8, Na 148, K 3.8, Cl 115, CO_2_ 17, anion gap 16, BUN 23, Glu 94 ALT 19, AST 75, serum ethanol not detected.

**Clinical Course:** Patient was acting normally in the ED waiting room. The patient’s wife reported that he left the waiting room, telling her that he was going to get some apple juice. Upon return, he sat down and slumped over in his chair. ED staff found the patient to be apneic and pulseless and began resuscitation. He was taken to a room where standard resuscitative measures were instituted, including IV access, chest compressions, endotracheal intubation, placement on a ventilator and provision of oxygen. Initial rhythm on the monitor was VT. Return of spontaneous circulation was established. He was tremulous, unresponsive, “posturing”, skin clean and dry, gag reflex and corneal reflexes absent, pupils 5–6 mm and nonreactive A dopamine infusion was started. Inspection of his person revealed a small vial of potassium cyanide in his pocket and a suicide note around his neck stating he wanted “no code.” Further history at that time revealed that he was in need of another “cardiac surgery” and was “just done with it.” The patient received sodium nitrite and sodium thiosulfate in standard doses. Computed tomography of the brain revealed “global infarcts” and “subarachnoid hemorrhage”. The patient was admitted to the ICU where he was declared that his brain was dead the next day, and life support was withdrawn.

**Autopsy Findings:** Autopsy included hemorrhagic gastritis, marked cerebral edema, cerebellar tonsillar herniation and infarct, cerebral venous sinus thrombosis. Postmortem specimens of heart blood were negative for amphetamines, barbiturates, carisoprodol, cocaine, opiates, and THC metabolite. Hospital blood lidocaine was > 1, 000 mg/mL, believed secondary to use lidocaine during ACLS resuscitation. Premortem blood from the hospital was positive for cyanide (qualitative). Urine specimen and postmortem blood specimens were negative for cyanide. Cause of death: cyanide intoxication. Manner of death: suicide.

**Case 186.** Acute potassium aluminum sulfate parenteral: undoubtedly responsible.

**Scenario/Substances:** A 78 y/o 88 kg male received 10 g potassium aluminum sulphate in 1 L D5W IV instead of per urethral catheter. He received 600 ml of the solution IV in 3–4 h after which patient felt cold and became tachycardic and dyspneic.

**Past Medical History:** Hematuria, prostate cancer.

**Physical Exam:** BP 132/82, HR 114, RR 18, T 97.3F, Urine cherry in color, urine output total volume 600 ml.

**Laboratory Data:** ABG-pH 7.54 / pCO_2_ 30 / pO_2_ 359, O_2_ sat 100% on ventilator. Na 136, K 4.0, BUN 9-17, Cr 0.89-1.26, Hgb 10.1, Hct 28, platelets 222, INR 2.2-2.8.

**Clinical Course:** CxR showed pulmonary embolism. He was twice successfully resuscitated following cardiac arrest. He intubated and sedated in the ICU, completed first dose of IV deferoxamine 1g in 1 L at 15 mg/kg/hr and hemodialysis. He received a second dialysis and deferoxamine treatment on Day 2. Attempts were made to wean patient off sedation on Day 3, but he became agitated and sedation was restarted. His BP became labile and norepinephrine was started. He was found to have blood clots in his urinary catheter. Based on the prognosis, the family opted for institution of comfort measures and he expired on Day 3

**Autopsy Findings:** Not available

**Case 199.** Acute hypochlorite parenteral: probably responsible.

**Scenario/Substances:** This 63 y/o male had just completed a hemodialysis run on his home dialysis machine. He forgot to disconnect himself from the machine before putting bleach into the machine to clean it and infused ∼60 ml of sodium hypochlorite bleach into his dialysis catheter. He “felt funny” and called EMS. He had a cardiac and respiratory arrest during transport, CPR was begun, intubation was attempted, and he was transported to the ED. He received multiple rounds of epinephrine and atropine enroute to the ED.

**Past Medical History:** Multiple surgical procedures, including right and left nephrectomies, partial ureterectomy, adrenalectomy, parathyroidectomy, arteriovenous fistula, autogenous arteriovenous fistula, and insertion of a tunneled centrally inserted central venous catheter. He had seasonal allergies, smoked cigarettes daily, used alcohol 1–2 times a month.

**Physical Exam:** The patient was unresponsive. His skin was cool. No detectable BP or HR.

**Laboratory Data:** ABG-pH 7.20/pCO_2_ 73/pO_2_ 11/HCO_3_ 28.2/BE -1, O_2_ sat 8%, Na 141, K 5.7, Glu 139, Ca (ionized) 1.06, total CO_2_ 30.

**Clinical Course:** In the ED, he was in PEA: CPR was resumed at 15 min post-arrest. He was intubated and a femoral line was placed. He received IV fluids, epinephrine (7 mg total), calcium, and sodium bicarbonate. He expired ∼1 hour after the accidental bleach exposure occurred.

**Autopsy Findings:** Not performed.

**Case 206.** Acute laundry detergent (pod) ingestion: undoubtedly responsible.

**Scenario/Substances:** A 7 m/o male bit into a laundry detergent pod and the contents entered his mouth. The child was crying with occasional cough and became somnolent. EMS was notified and transported the child. Vomiting occurred en route to the ED.

**Past Medical History:** Recent upper respiratory tract and urinary tract infections treated with cefdinir, but did not complete the course because of runny red stools.

**Physical Exam:** Somnolent with upper airway wheezing and retractions; moderate respiratory distress. HR 170, RR 30, T 37°C, O_2_ sats in the 80s% on RA and improved with supplemental oxygen. His palate and pharyngeal cavity had visible red spots.

**Laboratory Data:** ABG-pH 6.50 / pCO_2_ 70.5 / pO_2_ 27, Na 156, K 2.8, Cl 126. CxR right upper lobe infiltrate.

**Clinical Course:** During transfer preparations in the ED, the patient experienced a seizure. He was more lethargic with agonal breathing in the 50's. An interosseus catheter was placed, and he was endotracheally intubated; 3 h after exposure, the patient experienced a cardiac arrest and could not be resuscitated.

**Autopsy Findings:** Mild hyperemia of the oropharynx and tracheal without evident burns or ulcerations. There was a small amount green brown gastric content. There was significant asymmetric pulmonary congestion on right and some cerebral edema. UDS was negative. Central post- mortem blood propylene glycol of 33 mg/dl; gastric contents: propylene glycol of 370 mg/dL. No ethylene or diethylene glycol detected. The death was determined to be accidental exposure to laundry soap detergent.

**Case 209.** Acute magnets and carbaryl ingestion: undoubtedly responsible.

**Scenario/Substances:** A 19 m/o female was examined in the ED for complaints of vomiting and diarrhea, instructions for supportive care were given, and the patient was released. The next day she was found unresponsive by her mother. EMS and police were called, bystander CPR was performed and she was transported to the ED.

**Past Medical History:** Good general health

**Clinical Course:** On arrival to the ED, the patient had expired, but PALS was performed. Blood was noted in the nose and mouth, but no other signs of trauma were noted. ABG-pH 6.50/pCO_2_ 46/pO_2_ 36, Na 155, K 6.2, Glu 20 Hgb 3.6. Skeletal survey to rule out abuse was performed post-mortem in the ED did not reveal any acute or healing fractures. Portal venous gas and pneumatosis intestinalis was noted. Seven small metallic spherical radio dense foreign bodies were present within the posterior medial aspect of the left abdomen in a linear fashion. EMS and police reported that the child's room was covered in a while powder. The mother stated that the powder was carbaryl insecticide, which had been placed in the room at an unknown time.

**Autopsy Findings:** Cause of death was listed as ischemic bowel due to spherical magnets found in the small intestine, causing pressure necrosis when the magnets presumably adhered to one another with a portion of small bowel between them. Other conditions related to the death were bed sharing and unsafe sleep surface. No evidence of serious trauma was noted externally. Internal examination revealed the seven above-mentioned magnets to be within the bowel in a linear formation. The stomach and esophagus were normal, while the small bowel proximal to the magnets was hyperemic. Small bowel distal to the magnets was normal in appearance.

Femoral blood was drawn and analyzed. Carbaryl was NOT detected in blood. Ketamine was detected at 7.0 mcg/mL, but this was administered in the ED during intubation. Nor-ketamine was not detected. Heart blood was negative for ethanol. Vitreous electrolytes: Na, 140; K, 18; Cl, 131; Ca, 1.6; Mg, 0.92; Glu, 78; lactate, 21 mmol/L; urea nitrogen, 10; Cr 0.8.

Powder samples × 3 were assessed: all 3 samples were positive for carbaryl and 1-naphthalenol.

**Case 224**. Acute carbon monoxide inhalation: undoubtedly responsible.

**Scenario/Substances:** An 11 y/o male was found dead in bed in pool of emesis in a hotel room. His mother was found on the bathroom floor, unconscious suffering from severe CO toxicity. The source was determined to be a retrofitted swimming pool heater that vented very close to the window with a faulty exhaust line that leaked into the room as well. Very high levels of CO were noted when the pool heater was turned on later. Two deaths occurred in the same hotel room 2 months earlier, initially attributed to “heart attacks”, but were later determined to be due to carbon monoxide.

**Laboratory Data:** Postmortem COHb level from aortic blood was reported as > 60%.

**Autopsy Findings:** Autopsy demonstrated pulmonary edema and congestion. Petechiae were distributed over head and neck. Cause of death was carbon monoxide toxicity, with the manner being accidental.

**Case 283.** Acute hydrogen sulfide inhalation: undoubtedly responsible.

**Scenario/Substances:** A 53 y/o male collapsed inside an asphalt truck container and was pulled out by his son. His son also experienced symptoms. The tank was believed to contain hydrogen sulfide. EMS found that the patient had agonal breathing, intubated him with a laryngeal tube, and removed his clothing prior to transport to the ED.

**Past Medical History:** Hypertension.

**Physical Exam:** Upon arrival to the ED, the patient was unconscious with seizure-like movements. The laryngeal tube was exchanged for endotracheal intubation during which a large amount of emesis occurred resulting in aspiration. He was given hydroxocobalamin. On arrival, BP 130/80, HR 87, and O_2_ sat 82% on 100% FiO_2_. The urine was found to be in deep purple after the hydroxocobalamin treatment.

**Laboratory Data:** Initial ABG-pH 7.07 / pCO_2_ 58.0 / pO_2_ 60 / HCO_3_ 10.0, K 3.4, Cl 108, CO_2_ 18, BUN 16, Cr 1.4, Glu 146, Ca 8.1, AST 108, ALT 65. CK 807, INR 1.1, troponin I 0.5, and methemoglobin 0.8%.

**Clinical Course:** The patient was sedated using propofol, midazolam and fentanyl, and mechanically ventilated. He was given IV fluids and antibiotics. On hour 12, the patient became hypotensive, tachycardic, developed ECG changes consistent with an anterior wall myocardial infarction, and developed a PEA arrest. He was resuscitated with CPR and epinephrine, sodium bicarbonate, and calcium gluconate. He required post-arrest epinephrine and norepinephrine infusions. Post-arrest: pH 7.11, lactate 14.7, troponin I 3.5. He developed a T 38.7°C. The patient had a second cardiac arrest at Hour 21 and could not be resuscitated.

**Autopsy Findings:** Left ventricular hypertrophy and nephrosclerosis. No drug or chemical levels detected. The death was determined to be from an accidental exposure to hydrogen sulfide.

**Case 316**. Acute carbon monoxide inhalation: undoubtedly responsible.

**Scenario/Substances:** A 72-year-old female was found unresponsive and on respiratory arrest in her hotel room bed by housekeeping. CPR was initiated. She was intubated and taken to the local ED. Resuscitation attempts were unsuccessful and she expired. Her husband was found dead in the bathtub. The hotel room was not assessed for the presence of any gases.

**Past Medical History:** hypertension and atrial fibrillation.

**Autopsy Findings:** The ME initially assumed the patient and her husband died of overdoses. An autopsy showed pulmonary edema and mild cardiomegaly. Toxicology revealed a COHb of > 60%. Results were finalized 6 weeks after the deaths, and 1 week prior to an 11-year-old male dying of carbon monoxide toxicity in the same hotel room. An investigation determined the heater for the hotel's indoor pool was below the hotel room where all 3 deaths occurred and the heater exhaust was not functioning properly.

**Case 318.** Acute carbon monoxide inhalation: undoubtedly responsible.

**Scenario/Substances:** A 73-year-old male was found dead in the bathtub of his hotel room by housekeeping. CPR was initiated, but he was pronounced dead at the scene. His wife was found unresponsive in the bed. The hotel room was not assessed for the presence of any gases.

**Autopsy Findings:** Pulmonary edema, severe atherosclerosis, and cardiomegaly. The ME initially assumed that the patient and his wife died of overdoses. Toxicology revealed a COHb of > 60%. Results were finalized 6 weeks after the death, and 1 week prior to an 11-year-old dying of carbon monoxide toxicity in the same hotel room. An investigation determined the heater for the hotel's indoor pool was below the hotel room where all 3 deaths occurred and the heater exhaust was not functioning properly.

**Case 342.** Lead and ethanol ingestion: undoubtedly responsible.

**Scenario/Substances:** A 73 y/o male made and drank his own moonshine, and developed altered mental status the evening before presentation, and began having seizures at home. EMS intubated him, gave several doses of benzodiazepines, and transported him to the ED.

**Past Medical History:** His wife had been recently hospitalized and intubated secondary to lead encephalopathy thought to be caused by drinking homemade moonshine. She recovered with chelation to near baseline. She and the entire family were counseled to discontinue the use of this moonshine.

**Physical Exam:** In the ED, he was in status epilepticus, intubated, sedated. He was afebrile, BP 127/98, HR 80.

**Laboratory Data:** ABG-pH 7.36 / pCO_2_ 33 / pO_2_ 153 / HCO_3_ 19,

Bilirubin 0.8, AST 35, ALT 23, Alk phos 41, blood lead > 160 mcg/dL.

**Clinical Course:** The patient was sedated, placed on high dose antiepileptic agents and started on dimercaprol followed by Ca disodium EDTA. Despite maximal therapy, the patient remained in status epilepticus, and was treated with phenytoin, levetiracetam, propofol, midazolam, and phenobarbital. He continued to have subtle twitching during the hospitalization and seizure activity on his EEG. Repeat blood lead: 95 mcg/dL at 48 h after the initiation of chelation and 60 mcg/dL at 96 h. Despite continued therapy, the patient made no neurologic recovery. When propofol sedation was reduced, the patient would again start to seize. On Day 7, he became hemodynamically unstable with hypotension and bradycardia. Based on the prognosis, the family opted for institution of comfort measures and he expired on Day 9.

**Autopsy Findings:** Not available.

**Case 355.** Chronic freon inhalation: undoubtedly responsible.

**Scenario/Substances:** A 33 y/o male was huffing compressed Freon in the woods throughout the day with frequent loss of consciousness. He was found passed out in the woods and brought to the ED by EMS.

**Past Medical History:** Chronic back pain, reconstructive surgery following a motor vehicle accident, anxiety and depression. History of huffing including a case of pneumonitis 1 year earlier resulting from chronic huffing of compressed air.

**Laboratory Data:** Na 137, Cl 97, CO_2_ 17, anion gap 23, BUN 23, Cr 1.5, Glu 220, AST 53, CK 1,000, troponin 0.24, Ca 5.1, Ca (ionized) 0.6, WBC 20.

**Clinical Course:** On ED arrival, the patient was agitated, HR in the 140s. He was dehydrated but afebrile. He was given IV fluids, lorazepam, and promethazine. Within 2 h of arrival in the ED, he lost consciousness and began to seize. He developed VT and was electrically cardioverted to a sinus rhythm with HR 110. Calcium was administered. Labs showed albumin 3.8, ALT 22, Mg 1.0, CKMB 20.9, and Phos 1.7. Repeat Ca 5.3, repeat CK 2,245. The patient had another seizure ∼3 h later and developed VF, received defibrillation twice, was then intubated and transferred to the ICU. At that time he remained tachycardic, HR 106, BP 96/69, RR 20. Propofol infusion was started and he received electrolyte replacement. The patient expired ∼9 h post ED arrival.

**Autopsy Findings:** No autopsy was performed. Coroner concluded the death was due to fatal cardiac arrhythmias as a result of prolonged huffing of fluorinated hydrocarbons.

**Case 367.** Acute lamp oil ingestion/aspiration: probably responsible.

**Scenario/Substances: A** 15 m/o 12-kg male ingested/ aspirated torch fuel at home. EMS transported the patient to the ED.

**Clinical Course:** In the ED, the patient required oral intubation, was placed on oscillator ventilation, and arrangements were made for transfer for ECMO. Initial BP was “unstable”, pH 6.8, “CO_2_ in the 100's”, ABG-pH 7.183 / pO_2_ 64 / CO_2_ 57.9 / HCO_3_ 21.3 / BE 7. His status deteriorated during transfer to the tertiary care hospital. On arrival in the PICU, O_2_ sats 50–60%, O_2_ sat100% after ECMO. BP 94/42, HR “140's”, T 37.6°C. EEG showed no activity. After aggressive treatment over a course of 4 days, an EEG was done and showed no activity. Brain death was declared Day 4.

**Autopsy Findings:** Not available.

**Case 368.** Acute gasoline ingestion/aspiration: undoubtedly responsible.

**Scenario/Substances:** A 17-month-old male ingested gasoline, choked, vomited, and rapidly developed severe respiratory distress. EMS found him coughing, tachypnea and dyspneic and transported him to the ED. Supplemental oxygen was provided in ambulance, O_2_ sat 90%, but the child deteriorated and required intubation by EMS en route.

**Laboratory Data:** CxR showed “white-out” of lungs.

**Clinical Course:** In the ED O_2_ sat fell to 70%, and PEEP was added; he was transferred by air to a tertiary care hospital where he suffered a bradycardic arrest ∼7 h after ingestion initially responsive to atropine, epinephrine, and sodium bicarbonate. He arrested again a short time later and could not be resuscitated.

**Autopsy Findings:** Not available.

**Case 369.** Acute hydrofluoric acid ingestion: undoubtedly responsible.

**Scenario/Substances:** A 2 y/o male presented to the ED 30 min after ingesting a mouthful of automotive wheel cleaner. The substance had been stored in a water bottle, and was given to him by his grandmother, who thought she was giving the child a bottle of water.

**Physical Exam:** He presented awake and alert, but was drooling.

**Laboratory Data:** Initial laboratory work included a Ca, 8.1; K, 3.0; and venous pH, 7.21. Several h later Ca 2.6.

**Clinical Course:** Initial treatment consisted of IV calcium gluconate. Approximately 3 h after ED arrival, the patient had a cardiac arrest. He was resuscitated and given additional calcium. He was transferred to a tertiary children's hospital where he was aggressively treated with IV calcium, and suffered a terminal cardiac arrest ∼7 h after ingestion.

**Autopsy Findings:** Not performed.

**Case 377.** Acute dinitrophenol ingestion: undoubtedly responsible.

**Scenario/Substances:** A 19 y/o male purchased dinitrophenol on the internet as a weight loss supplement, took 1 dose (quantity unknown) in the morning, and began feeling unwell late that day and sought care at the ED.

**Past Medical History:** No reported serious, chronic medical problems. No psychiatric history.

**Laboratory Data:** ABG-pH 7.46, Cr 1.4, Phos 6, other electrolytes unremarkable, lactate 2.9 mmol/L, salicylates 27, serum acetaminophen and ethanol not detected.

**Clinical Course:** Upon arrival to the ED, the patient was awake and conversant, HR 120–140, and hypertensive. He was given IV fluids and lorazepam. Mental status declined over the following 2 h, HR increased to 170s, systolic BP 100, T 38.1°C, RR 45, and O_2_ sat 99% on room air. He received additional IV fluids and IV lorazepam. Methemoglobin was not detected: respiratory and mental status continued to worsen requiring intubation and external cooling measures which were initiated. The patient suffered an asystolic cardiac arrest, ACLS was initiated, but resuscitation was unsuccessful. During the resuscitation T was > 42.71°C (the upper limit on the thermometer).

**Autopsy Findings:** not available.

**Case 380.** Acute-on-chronic dinitrophenol and diphenhydramine ingestion: probably responsible.

**Scenario/Substances:** A 28 y/o male was using dinitrophenol 200 mg a day for weight loss, ingested 4 g in a suicide attempt.

**Past Medical History:** Obesity

**Physical Exam:** Awake but “groggy” and diaphoretic on presentation, BP 156/74, HR 174, T 37.9°C, RR 40.

**Laboratory Data:** None provided

**Clinical Course:** The patient was given lorazepam IV for agitation. Due to the expected high lethality of DNP, lipid emulsion infusion was given. Prior to transfer to a transferred to a tertiary care hospital, HR 184, BP 163/62, and T 38.4°C. The patient was extremely agitated during transport and 7 hospital personal were required to manage him. He had a cardiac arrest soon after arrival at the tertiary care hospital from which he could not be resuscitated.

**Autopsy Findings:** Post mortem blood was negative for cocaine, amphetamines, THC and toxic alcohols. 2, 4-dinitrophenol was not detected (specific HP-TLC assay). Trace amounts of diphenhydramine (within the therapeutic concentration) were found. ME final diagnosis: death probably due to 2, 4-dinitrophenol toxicity.

**Case 384**. Acute DEET (insect repellent) ingestion: undoubtedly responsible.

**Scenario/Substances:** A 37 y/o male obtained and ingested a 6 ounce bottle of DEET insect repellant. Patient had a witnessed seizure and EMS was summoned. Patient had a VT cardiac arrest enroute to hospital. He received 20 min of CPR and received epinephrine, sodium bicarbonate, dextrose, naloxone and atropine with return of spontaneous circulation. He was intubated and given oxygen prior to arrival at the ED.

**Past Medical History:** Developmental delay (profound, lived in a group home), PICA, and cardiomegaly.

**Physical Exam:** BP 84/60, HR 96, RR 18, O_2_ sat 100% on 100% FiO_2_, T33.5°C. Head atraumatic, pupils fixed and dilated at 8 mm, oroendotracheal tube in place, multiple abrasions on anterior chest with some oozing of blood, no bowel sounds, and urinary catheter in place with grossly bloody urine without clots.

**Laboratory Data:** ABG-pH 7.15/pCO_2_ 42.1/pO_2_ 172/HCO_3_ 13.9, lactate 9.1, PT 22.9, INR 2, AST 404, ALT 397

serum acetaminophen, ethanol and salicylate not detected, UDS negative, ECG (initial): sinus tachycardia with intraventricular conduction delay, no ST/T wave changes, QTc 507, ECG #2: sinus rhythm at ventricular rate, normal axis, QTc 537.

**Clinical Course:** Patient was placed on a hypothermia protocol, given NS 2 L bolus and admitted to the ICU where a norepinephrine infusion was started. Over the following 48 h hypothermia and tachycardia resolved and BP was stabilized with pressors but patient remained completely unresponsive. Cerebral flow study demonstrated no flow, EEG demonstrated diffuse background with little appreciable brain activity, and non-contrast brain MRI showed cerebral edema, transtentorial and tonsillar herniations. On Day 3, the patient was declared brain dead.

**Autopsy Findings:** Not performed.

**Case 389.** Acute malathion ingestion: undoubtedly responsible.

**Scenario/Substances:** A 49 y/o man intentionally drank a bottle of malathion. EMS was called and transported the patient to the ED.

**Past Medical History:** Alcoholism, COPD, hypertension and depression.

**Physical Exam:** Upon arrival to the ED, the patient was unresponsive with posturing movements, lungs clear, bowel sounds normal, pupils 2 mm and reactive. BP 246/112, HR157, RR 30, O_2_ sat 92%, T (oral) 36°C.

**Laboratory Data:** Upon transfer to the referral hospital: ABG-pH 7.26 / pCO_2_ 33 / pO_2_ 495 / HCO_3_ 15.0, Na 142, K 3.3, Cl 107, CO_2_ 16, BUN 3, Cr 1.2. On Days 3, 4, and 5: Cr 1.1, 1.7 and 3.9 respectively. WBC peaked on Day 4 at 26.

**Clinical Course:** He was endotracheally intubated, sedated, and mechanically ventilated using midazolam, fentanyl, and propofol. Diarrhea was treated with a total of 7 mg of atropine and pralidoxime (2 g of IV push and an infusion at 8 mg/kg/hr). All body fluids had a strong chemical odor. On Day 2, the patient had no spontaneous neurological activity despite being weaned from all sedation. Bronchoscopy showed aspiration pneumonia. The patient developed progressive hypotension and tachycardia requiring vasopressors, became acidotic and anuric. He died on Day 5.

**Autopsy Findings:** Bronchopneumonia, left ventricular hypertrophy, liver steatosis, BPH, and diverticulosis coli. Antemortem blood: malathion concentration of 0.12 mg/L and a naloxone concentration of 0.14 mg/L, no other drugs detected. The death was determined to be due to intentional malathion poisoning.

**Case 395.** Acute paraquat ingestion: undoubtedly responsible.

**Scenario/Substances:** A 66 y/o male, upon returning to his vehicle after exercising, picked up a bottle of blue-green liquid that he thought was a sports drink and swallowed a large mouthful. He realized that this was an herbicide obtained from a friend and reported to the ED for evaluation. At that time, was not able to provide the name of the herbicide.

**Past Medical History:** Hypertension, hypercholesterolemia, and anxiety, no history of smoking tobacco or lung disease.

**Physical Exam:** Upon initial presentation to the ED, he complained of throat pain, nausea, and “feeling bad all over”. At that time, BP 186/106, HR 86, and no respiratory distress. He became diaphoretic and vomited a blue-green liquid. After vomiting, BP 129/58, RR 16, O_2_ sat 100% on room air, ECG normal.

**Clinical Course:** Within an hour of exposure, the herbicide was determined to be paraquat, concentration unknown. Although his vitals normalized, he was admitted overnight for persistent vomiting for which he received multiple doses of ondansetron. No activated charcoal was administered for fear of aspiration. Nearly 48 h after observation admission, he was discharged. He returned to the hospital that evening complaining his throat felt swollen and made it difficult to breathe. He was discharged from the ED on antibiotics and steroids. On the following day, he presented to a tertiary care center for a sore throat, swollen tongue, and persistent hiccups, treated with chlorpromazine. His mouth appeared irritated similar to a caustic injury. While in the ED, it was discovered that the patient was having renal failure with an elevated BUN 76 and Cr and 7.2. His O_2_ sat was 92% on room air, and he had oliguria despite administration of a large of amount of IV fluids. His O_2_ sat dropped into the 80s, and he was admitted to the ICU where they initiated oxygen at 3 L via CPAP. The patient developed severe, painful oral sores and swelling. The initial steroids were stopped and intense oral care started. The next morning, he was intubated and FiO_2_ was changed from 100% O_2_ to nitric oxide at 28–30% O_2_ and started on n-acetylcysteine, methylprednisolone, 1 g every 24 hrs, cyclophosphamide (he received only 3 doses), MES sodium salt and vitamin C. CVVH was begun. During the next several days, his oral sores continued to be severe with excessive bleeding with care, BUN peaked at 108 and Cr 12. and he was continued on nitric oxide therapy although FiO_2_ was frequently as high as 40% as the treatment team attempted to maintain O_2_ sat above 80%. Numerous CxR's showed infiltrates and atelectasis, and his lung sounds became coarse and diminished at the bases. He was sedated and started on tube feedings and electrolyte replacement while he continued on dialysis. Two weeks after the exposure, he began producing thick, creamy, blood-tinged secretions from his lungs. They were unable to wean sedation due to agitation, tachypnea, hypertension, and decreasing O_2_ sats. Cultures from his lungs showed several pathogens including pseudomonas. He was treated with antibiotics and antifungals. The patient continued to deteriorate, was paralyzed, nitric oxide was stopped, and FiO_2_ was increased to 100%. The patient expired 3 weeks after the ingestion.

**Autopsy Findings:** The coroner's reported that the patient's wife claimed that there were 2–3 ounces missing from the bottle. However, because the patient immediately sought care and had no evidence of suicide intent, the ingestion was ruled an accident and no further investigation or autopsy was performed.

**Case 396.** Acute-on-chronic carbamate insecticide ingestion: probably responsible.

**Scenario/Substances:** A 69 y/o male had an argument with his significant other and stated he was going to kill himself. He was later found with a can of the carbaryl, unresponsive, sweating, with signs of defecation and urination. Upon arrival, EMS noted rhonchi and rales that were audible without a stethoscope. The patient was intubated using rapid sequence with succinylcholine and transported him to the ED. A red bottle of carbaryl was found in the kitchen sink.

**Past Medical History:** Aortic stenosis, s/p valve repair, implanted pacemaker. Medications included atorvastatin, clobetasol, lisinopril, magnesium, and metoprolol. History of alcohol abuse, a prior suicide attempt, a daughter committed suicide “years ago.”

**Physical Exam:** Unresponsive, BP 112/64, HR 75 (paced rhythm), intubated.

**Laboratory Data:** pH 7.246-7.456, Hgb 17.7-19.1, WBC 31.1, BUN 27, Cr 3.5, Glu 129, bilirubin 2.9, AST 70, ALT 34, Na 141-149, K 3.1-4.4, CL 111-119, CO_2_ 13-17, troponin 0.978, lactate 10.2 mmol/L, Mg 1.4, INR 1.12, serum acetaminophen, ethanol and salicylate not detected. Blood cultures showed no growth

**Clinical Course:** The patient was placed on ventilator, sedated with lorazepam, and had copious lung secretions needing frequent suctioning. He received 5 doses of atropine 1 mg each and 1 dose of 2 mg atropine. His secretions decreased with the atropine. BP 149/89, HR 84, RR 18, O_2_ sat 95%. He opened his eyes, and was placed on propofol. His BP dropped 102/65, HR 75 (paced), secretions and diarrhea increased. He became more active without muscle fasciculations but developed renal failure. Based on the prognosis, the family opted for institution of comfort measures and he expired on Day 2.

**Autopsy Findings:** Not available.

Case 397. Acute paraquat ingestion: undoubtedly responsible.

**Scenario/Substances:** A 70 y/o female who drank from an iced tea bottle later was found to contain paraquat. She was brought to the ED 30–45 min later.

**Laboratory Data:** Glu 130, BUN 17, Cr 1.2, AST 28, ALT 22.

**Clinical Course:** She presented to the ED awake, alert and vomiting. Vital signs were said to be “stable”. At Hour 24 vomiting had stopped, the patient was taking a liquid diet, but had increasing pain in the throat with swallowing or talking. On Day 2, she had increased oral discomfort, BUN 22, Cr 2.4. In subsequent days, BUN and Cr increased, throat and substernal pain continued, and extensive bilateral pulmonary infiltrates were associated with decreasing O_2_ sats. On Day 5, she was intubated and placed on a ventilator on. On Day 8, BUN 67, Cr 4.4. Day 9 hemodialysis was initiated, but pulmonary function continued to decline, and life support was discontinued on Day 14 and she died.

**Autopsy Findings:** Autopsy was not performed, but the state Department of Pesticide Regulation obtained the iced tea bottle from which the patient had ingested the liquid and confirmed the presence of a diluted paraquat solution.

**Case 400.** Acute mitragynine, paroxetine and lamotrigine ingestion: probably responsible.

**Scenario/Substances:** The 36-y/o male had a generalized tonic-clonic seizure and was found down at home by his family. EMS found the patient pulseless and apneic, intubated him, and initiated ∼30 min of CPR in the field. The patient received epinephrine and naloxone en route. He was found with empty bottles of lamotrigine, paroxetine, and an empty packet labeled “Da Pimp Bomb” with ingredients described as pure kratom.

**Past Medical History:** Depression, polysubstance abuse, history of suicidal ideation.

**Physical Exam:** After return of spontaneous circulation: unresponsive on ventilator, BP 106/63, HR 118, T 34.3°C, O_2_ sat 96%. Pupils dilated but sluggishly reactive, heart tachycardic, lungs with coarse breath sounds, abdomen soft and nontender, GCS 3T with 1 + reflexes bilaterally and no clonus.

**Laboratory Data:** Initial labs:

INR 1.42, lactate 16 mmol/L, serum acetaminophen and salicylate not detected,

**Clinical Course:** Upon arrival in the ED, he was found to be in asystole and received sodium bicarbonate, epinephrine, magnesium, Ca chloride, lipid emulsion, and TPA. After 40 min of CPR spontaneous circulation returned. ECG showed wide complex tachycardia with large terminal R wave in aVR that narrowed after additional sodium bicarbonate. The patient underwent a cooling protocol until Day #4 when he underwent evaluation by neurology and critical care and was declared brain dead. The body was released for organ donation the same day.

**Autopsy Findings:** Diagnoses included marked cerebral edema consistent with anoxic brain injury, with multifocal brainstem hemorrhage, multiple small recent pulmonary infarcts and pulmonary emboli, and recent thrombosis in prosthetic venous plexus. The autopsy revealed no other anatomic cause of death. Laboratory testing showed a qualitative positive screen for mitragynine and 7-OH mitragynine only. Cause of death was severe hypoxic encephalopathy complicating apparent mitragynine toxicity. The packet of the suspect drug was analyzed by law enforcement and found to contain only mitragynine. The manner of death is accident by the report.

**Case 401**. Acute cardiac glycoside ingestion: probably responsible.

**Scenario/Substances:** A 74 y/o male blended 7–9 oleander leaves with water in a blender and drank it as suicidal gesture. A couple of hours later his wife found him having nausea and vomiting, and brought him to the ED.

**Past Medical History:** Depression, GERD, chronic pain, atrial fibrillation, pacemaker, hypertension, and hyperglycemia. Patient did not have a history of taking digoxin.

**Laboratory Data:** Serum digoxin, 3.23 ng/mL.

**Clinical Course:** Awake, alert, and oriented x 3, BP 131/61, HR 60 (paced), RR: 20, O_2_ sat 95%. Patient was given antiemetics, activated charcoal and digoxin immune Fab and admitted overnight for observation and monitoring. On Day 2, digoxin 1.9 ng/mL, still with nausea which was treated with antiemetics. On Day 3, the patient became tachypneic (RR 37), BP 104/30 HR 60 (paced). He received IV fluid bolus. He developed hyperkalemia, WBC 30.6, and decreased renal function and started having episodes of VF. ACLS was started. The patient was defibrillated twice and given epinephrine, bicarbonate, and atropine. During the code, the family determined that he would not want to be resuscitated, opted for institution of comfort measures, and he expired.

**Autopsy Findings:** Not performed.

**Case 404.** Acute buprenorphine/naloxone (sublingual) ingestion: undoubtedly responsible.

**Scenario/Substances:** A 5 y/o female ingested a buprenorphine/ naloxone tablet belonging to her caregiver (her aunt). Within 1 h, the child was drowsy and nauseous. The caregiver declined repeated medical advice to bring the child to the ED. The child was later discovered unresponsive, lying on her bed and was pronounced dead at the scene

**Autopsy Findings:** Autopsy showed pulmonary edema. Iliac blood free buprenorphine was 2.5 ng/mL, and free norbuprenorphine was 4.3 ng/mL. Vitreous ethanol level was 19 mg/dL. Cause of death: buprenorphine intoxication. Manner of death: homicide, owing to failure of caregiver to follow medical advice.

**Case 495.** Chronic acetaminophen ingestion: undoubtedly responsible.

**Scenario/Substances:** A 27 y/o 71-kg female presented to the ED with complaints of stomach pain and was admitted. She reported received 2.6 g acetaminophen on Day 1 and 2.95 g on Day 2. On Day 3, she had an episode of loss of consciousness, hypoglycemia, and a possible seizure. It was later determined that she had been taking acetaminophen/oxycodone and acetaminophen (5 bottles) over the past several months. Her mother had passed away 5 months prior, she lost her job and had been having suicidal thoughts for which she had seeing a psychiatrist. Needles and syringes were found in her purse.

**Past Medical History:** Anxiety, depression, possible substance abuse, and gastric bypass surgery previous year. Medications: sucralfate, misoprostol, pantoprazole, hydromorphone, and acetaminophen/oxycodone.

**Laboratory Data:** ABG-pH 7.18 / pCO_2_ 18 / pO_2_ 256 / HCO_3_ 6.8 on the ventilator., WBC 18.6, Hgb 10.9, Hct 32, platelets 235, Day 1: AST 27, ALT 54. Day 2: PT 12.9, INR1.1, BUN 5, Cr 0.5. Day 3: acetaminophen 123 mcg/mL, AST 2,074, ALT 1,355, bilirubin 3.4, albumin 2.6 g/dl, INR 5.9, ammonia 55, BUN 5, Cr 1, Glu 179, lactate 4.8. UDS negative for opiates. Day 4: AST 7,073, ALT 3,676, bilirubin 4.5, INR 8.9, ammonia 112, Day 5: AST 4,239, ALT 3,208, bilirubin 5.1, INR > 10, acetaminophen not detected.

**Clinical Course:** Vital signs (on ventilator): BP 123/65, pulse, 98, T, 37 degrees C, RR 15-16. She was moving all extremities, and pupils were 3mm, equal and reactive.

She was started on N-acetylcysteine (NAC) on Day 3 of admission and loaded with 10,500 mg and was scheduled to receive 50 mg/kg over the subsequent 4 h, the NAC dosing was then increased to 15 mg/kg/h and she was started on D10W infusion. On Day 3, she was transferred to a tertiary care hospital. She became hypotensive and received norepinephrine, vasopressin, and phenylephrine. Her transaminases continued to increase along with her INR. At this point, her family declared her a do-not-resuscitate (DNR). She was given phytonadione on Day 4, however, was having no active bleeding. On this same day, her NAC dose level was decreased despite being advised to maintain the current dose due to her critical clinical status and lack of indication for using the limited dose. Day 5 BP 105/50, HR 124, RR 11 on pressure support, T 38.1°C, O_2_ sat 95%. NAC was discontinued on Day 6. Based on the prognosis, the family opted for institution of comfort measures and she expired on Day 6.

**Autopsy Findings:** Not available.

**Case 607.** Acute salicylate ingestion: undoubtedly responsible.

**Scenario/Substances:** A 36 y/o male wrote suicide notes, ingested 500 tablets of 325 mg aspirin, and was presented to the ED ∼3 h later.

**Past Medical History:** Depression related to the death of his wife 2 years ago.

**Clinical Course:** The patient had nausea with hematemesis in the ED, and salicylate level was 84 mg/dL. He was transferred to a second hospital where his salicylate was 94 mg/dL, ABG-pH 7.45/pCO_2_ 27/pO_2_ 113/HCO_3_ 19, K 4.3, and Cr 1.3. He was transferred to a tertiary care hospital for hemodialysis. His ABGs showed a mixed respiratory alkalosis with metabolic acidosis. Sodium bicarbonate was given. He was admitted to the ICU and experienced nausea, vomiting, and diarrhea for 2–3 h. He became confused, agitated, and combative. A repeat salicylate drawn an estimated 9 h after ingestion was 108 mg/dL. At 11.5 h after ingestion ABG-pH 7.22 / pCO_2_ 38 / pO_2_ 88 / HCO_3_ 16. The renal team started dialysis, but the patient abruptly developed QRS widening and went into asystole. ACLS resuscitation was unsuccessful, and he died ∼12 h after ingestion.

**Autopsy Findings:** Not performed

**Case 1057.** Chronic colchicine ingestion: probably responsible.

**Scenario/Substances:** A 78 y/o male with multiple medical problems was discharged on colchicine for gout. He took as many as 15 tablets (0.6 mg each) over a period of 3–4 days. There was no evidence of an acute self-harm intent. He developed profuse diarrhea (7–8 stools/day) and weakness, and was brought back to the ED.

**Past Medical History:** Gout, end-stage renal disease on hemodialysis, hypertension, hypokalemia, leukopenia, thrombocytopenia, peptic ulcer disease, myocardial infarction, congestive heart failure, anemia, syncope, cardiogenic shock with PEA and VT arrest, methicillin-sensitive *S. aureus* (MSSA) sepsis. Medications included colchicine, allopurinol, aspirin, amiodarone, amlodipine, calcitriol, divalproex, pantoprazole, sevelamer, and simvastatin.

**Physical Exam:** He was frail-appearing but oriented, BP 94/73, HR 88, RR 27, O_2_ sat 93%, T 37.4°C.

Lungs clear, normal cardiac exam, and no abdominal distension.

**Laboratory Data:** Hgb 9.2, Hct 28.5, WBC 1.7, platelets 32,

AST 69, ALT 31, bilirubin 0.8, INR 1.7, troponin 0.4, lactate 7.4 mmol/L, CK 109. CxR showed R lung base opacity with small bilateral pleural effusion, and repeated CxR showed pulmonary edema.

**Clinical Course:** Patient continued to be hypotensive despite fluid resuscitation and multiple vasopressors and inotropes. He was intubated and placed on a ventilator. He had a junctional bradycardia with escape rhythm, and his ECG showed a new LBBB. He was treated with CVVH and a bicarbonate drip. He was given antibiotics for possible sepsis. He also received filgrastim for his leukopenia. His lactate level peaked at 27.9 mmol/L. He developed hepatic failure with peak AST 3,495, ALT 1,676, bilirubin 7.1, CK rose to 3,500. He died from multi-organ failure 24 h after admission.

**Autopsy Findings:** The ME reported colchicine 4.0 ng/mL from premortem hospital blood (1 hour after arrival in the ED).

**Case 1085.** Acute salicylate ingestion: undoubtedly responsible.

Scenario: An 11 m/o male was given a medicine bottle to play with by his parents and was later found with the open bottle of enteric coated 325 mg salicylic acid. The patient had orange residue on his face, 1 intact tablet was removed from his mouth by a family member, and he was brought to the ED.

**Laboratory Findings:** The 6-hour salicylate level was 107 mg/dL and would later peak at 123 mg/dl. Na 146, K 2.6, anion gap 29, Glu 712, BUN 13, Cr 1.2.

**Clinical Course:** In the ED, the patient was alert and age appropriate. HR 154, RR 30, T 37°C, O_2_ sat 100% on room air. Family initially reported that, at most, 7 tablets were unaccounted for. He vomited thrice with 2 aspirin tablets visible in the emesis. He was given activated charcoal, IV fluids, and sodium bicarbonate 40 meq/hr. He was admitted to the PICU where he became severely tachycardic (HR 221), tachypneic (RR 45) and hyperthermic (T 38.5°C). He experienced electrolyte abnormalities including hypokalemia, hypernatremia, and hyperglycemia. On Day 2, the patient was intubated in preparation for transfer to a HCF that could provide hemodialysis when he went into cardiac arrest and expired.

**Autopsy Findings:** Petechial hemorrhages of the heart, thymus, and brain. The brain had non-volumetric subdural and subarachnoid hemorrhages. The salicylate concentration of antemortem blood 7 h post ingestion was 850 mg/L (85 mg/dL). The manner and cause of death was accidental ingestion resulting in salicylate toxicity.

**Case 1088.** Acute methadone ingestion: undoubtedly responsible.

**Scenario/Substances:** Aunt of a 19 m/o female was watching the child while mom attended a recovery group meeting. When mom arrived home she noticed the child was tired, so she put her down for a nap. When mom went to wake child, she noticed her lips were blue so she took her to the ED.

**Laboratory Data:** UDS positive for methadone.

**Clinical Course:** Upon arrival to ED, child's skin was ashen and oxygen was given. UDS came back positive to methadone, naloxone was given, her color improved, and she became more alert. Continuous naloxone infusion was started at 25 mcg/kg/min. She was protecting her own airway. The next day, child developed respiratory depression, apnea, and her HR dropped to 80's. She was intubated using rapid sequence intubation with fentanyl, her HR improved, and naloxone infusion was continued. That evening, she went into acute respiratory failure and suffered a cerebral herniation. Emergency craniotomy was performed and drain inserted, but pressures in her brain remained high. Epinephrine, norepinephrine, and vasopressin were used for pressure support. She developed diabetes insipidus. Continuous EEG showed no activity. She was determined to be brain dead, and the organs were donated.

**Autopsy Findings:** Acute necrosis of brain tissue related to methadone toxicity. Pre-mortem: methadone 248 ng/mL, EDDP 13 ng/mL.

**Case 1096.** Acute sevoflurane inhalation: undoubtedly responsible.

**Scenario/Substances:** A 37 y/o male nurse anesthetist was found at home hooked up to an anesthesia machine with sevoflurane. Patient was found in cardiopulmonary arrest, was resuscitated, and intubated. Initial post-resuscitation rhythm was atrial flutter with rapid ventricular response. He had seizure-like activity and was given phenytoin

**Past Medical History:** Insomnia (reported to be using his anesthesia machine for sleep)

**Laboratory Data:** Initial Ca (ionized) 1.02. Toxicology screen for drugs of abuse and toxic alcohols was negative.

**Clinical Course:** He received Ca IV for low Ca, a calcium channel blocker IV for his atrial flutter, and was placed on 48 h post-resuscitation hypothermia protocol. BP 101/58, HR 89, O_2_ sat 100 % on O_2_, T 32°C. Head CT was consistent with anoxic brain injury. He remained paralyzed with cis-atracurium, received propofol for seizure and sedation, and was receiving norepinephrine for pressure support. After 2 EEGs, he was declared brain dead and his organs were made available for donation.

**Autopsy Findings:** Sevoflurane from blood drawn at admission 5.9 mcg/mL (upper reporting limit is 0.10 mcg/mL). Post mortem phenytoin 12 mcg/mL. No other injuries or pathology were found on autopsy.

**Case 1100.** Acute lidocaine parenteral: undoubtedly responsible.

**Scenario/Substances:** A 77 y/o female nursing home resident came to the ED for an unknown reason.

**Past Medical History:** COPD, hypertension, diabetes mellitus, seizure disorder, and s/p pacemaker placement.

**Laboratory Data:** K of 6.0 was reported, but ECG did not show signs of hyperkalemia.

**Clinical Course:** The patient was to receive 25 g dextrose and 10 U insulin for the hyperkalemia, instead she received an unknown amount (40–100 mg) of lidocaine IV.

Immediately after the bolus, she became unresponsive, possibly had a seizure, developed a wide complex bradycardia that her pacemaker did not capture, and BP 130's/80's. She received dextrose, Ca, and sodium bicarbonate to treat her hyperkalemia. She developed asystole during the next 30 min. ACLS was initiated, and the patient was given lipid emulsion, but she could not be resuscitated.

**Autopsy Findings:** Severe emphysema, dilated cardiomyopathy, and kidney disease. Lidocaine was 4.6 mg/L, cause of death was lidocaine toxicity, and type of death was accident (medication error).

**Case 1102.** Acute lidocaine ingestion: undoubtedly responsible.

**Scenario/Substances:** A healthy 13 m/o female was being cared for at home by her 16 y/o brother, while their parents were visiting her twin sister in the PICU at a tertiary care pediatric facility. This patient started having seizure activity, and her brother called 911. EMS arrived 9 min later to find her actively seizing, unresponsive, and cyanotic with shallow, agonal respirations. HR 150, RR 12, O_2_ sat 100% on room air. She was transported with bag–valve–mask ventilation.

**Past Medical History:** No prior medical problems or hospitalizations. The twins had unremarkable 1-year well baby checkup visits 1 week earlier. The patient's twin sister had been taken to the ED 2 days prior with seizures followed by cardiorespiratory arrest. She was resuscitated and transferred to the tertiary care pediatric hospital where she remained unresponsive and ventilator dependent. Neurologic and cardiac evaluations had not yielded the cause of her seizures and arrest.

**Physical Exam:** In the ED, she was dusky, foaming at the mouth, actively seizing, apneic, strong odor of stool, absent corneal reflex, abdominal distension, unresponsive, no signs of trauma, GCS 3, BP 131/99, HR 160's, apneic, O_2_ sat 86% on O_2_ via bag/mask

**Laboratory Data:** Glu 189, ECG rhythm strips: initial narrow-complex tachycardia, then narrow-complex bradycardia, then wide-complex agonal rhythm.

**Clinical Course:** In the ED, IV access was established arrival, and seizures resolved following 1 mg of lorazepam. She received 20 mg of succinylcholine for intubation. Within 3 min after these medications, she became progressively bradycardic and then pulseless. CPR was started and was intubated. She received 27 doses of epinephrine, 4 doses of atropine, 2 doses of bicarbonate, and 1 dose each of naloxone, glucagon, and calcium gluconate during the 90-min unsuccessful resuscitation. After her death, police investigated her home and found a empty bottle of viscous lidocaine 2% on the coffee table in the parlor. The medication had been prescribed to both siblings separately 3 months prior, for topical pain relief from teething. The twin sister in the PICU was found to have very high levels of lidocaine in her urine. One month later, the 16 y/o brother admitted that he had mistakenly been adding the lidocaine to the twins’ milk bottles to treat their teething pain.

**Autopsy Findings:** Autopsy failed to disclose an anatomic cause of death. Postmortem heart blood obtained 24 h after death: lidocaine was 6.4 mcg/mL, monoethylglycinexylidide (MEGX) was 4.1 mcg/mL. Both the concentrations are consistent with reported toxic levels. ME's final cause of death was most likely lidocaine toxicity and the manner of death accidental.

**Case 1109.** Chronic rivaroxaban ingestion: contributory.

**Scenario/Substances:** A 66 y/o male developed mild left upper quadrant pain, became pale, sweaty, weak, and had an episode of vomiting. EMS reported seizure-like activity lasting 15–20 seconds during transport to the ED.

**Past Medical History:** Hypertension, COPD, migraine headache, major depressive disorder, GERD, dementia, seizure disorder. S/p bilateral knee surgery, lower back surgery for degenerative disc disease, left hip fracture surgery (1 month prior). Medications: rantitidine, doxepin, memantine, lorazepam, citalopram, donepezil, levetiracetam, rivaroxaban 20mg PO daily (started 3 weeks prior), before that he was on enoxaparin)

**Physical Examination:** In the ED BP 58/39, HR 88, RR 20. The patient presented with pallor, diaphoresis, agitation, confusion, and altered mental status, and abdominal distention. Bowel sounds were present and stool was occult blood positive. Ecchymosis bilaterally in lower quadrant, left thigh area.

**Laboratory Data:** Electrolytes unremarkable, Glu 202, Hgb 10.5, Hct 33.7, WBC 10.4, PT 12.9, INR 1.2, PTT 30.

**Clinical Course:** Patient exhibited episodes of hypotension in the ED for which he was given IV fluids and placed on a low-dose phenylephrine drip; systolic BP increased to 90s–low100s. CT of chest and head was normal. CT of abdomen and pelvis showed hemoperitoneum with blood around the liver and spleen, without obvious liver or spleen lacerations and mild fusiform dilatation of the distal abdominal aorta without evidence of aneurysmal leakage. Initial Hgb was 10.5, and repeat was 7.8. Clinical impression was hemoperitoneum with hemorrhagic shock with coagulopathy from rivaroxaban. Four units of packed RBCs were given, and he was transferred to a tertiary care hospital via helicopter. During transport, infusions of packed RBCs and vasopressors continued. On arrival at the tertiary hospital, the patient was awake, alert, and pale with some abdominal distention. The patient remained normotensive with systolic BP 104 on phenylephrine. The trauma team ordered reversal of the rivaroxaban with 5,000 units of prothombin complex plus IV vitamin K. Patient was intubated 6 hour (tertiary care hospital) and received multiple blood products including packed RBCs, FFP, and prothombin complex concentrate. He remained hemodynamically unstable on pressors with low Hgb. EEG showed no activity. Based on the prognosis, the family opted for institution of comfort measures and he expired on Day 2.

**Autopsy Findings:** Not performed.

**Case 1111.** Acute-on-chronic enoxaparin subcutaneously: contributory.

**Scenario/Substances:** A 73 y/o male was inadvertently given enoxaparin Q 2 h instead of Q 12 h as prescribed s/p hip fracture complicated by deep vein thrombosis. He received a total of 320 mg subcutaneously and presented with bleeding from his gums, epistaxis, and hemoptysis.

**Past Medical History:** Alzheimer's dementia, alcoholic cardiomyopathy, cirrhosis, and anemia of chronic disease.

**Physical Exam:** Alert and oriented, BP 100/63, HR 106.

**Laboratory Data:** WBC 14.7, Hgb 8.5 g/dL, Hct 25.7 %, platelets 389, PT 16, INR 1.2, PTT 49.5.

**Clinical Course:** Patient expired from an acute GI bleed on Day 1 despite administration of FFP, IV fluids, and packed RBCs.

**Autopsy Findings:** Not performed.

**Case 1136.** Acute valproic acid ingestion: undoubtedly responsible.

**Scenario/Substances:** A 63 y/o female's sister called police for a welfare check when the patient did not show up for a scheduled visit. Police and EMS entered into the home, found the patient unresponsive with pin point pupils, and transported her to the ED.

**Past Medical History:** Bipolar disorder, anxiety, and paranoia; previous suicide attempt was with aspirin when she was 20 y/o.

**Laboratory Data:** Initial complete blood count, metabolic panel, and liver transaminases were unremarkable. Serum acetaminophen, salicylates, and ethanol levels were not detected; UDS negative. Ammonia 346, later 195 and finally, at 43 Hour 37. Valproic acid > 300 throughout her hospital course. ECG was unremarkable.

**Clinical Course:** In the ED, BP 70/40, HR 65 with a depressed level of consciousness. She was intubated and placed on mechanical ventilation. She received 3 L NS and was started on an IV norepinephrine infusion for hypotension. The patient was empirically started on levocarnitine. With maximum doses of norepinephrine, phenylephrine, and epinephrine: BP 119/87, HR 93. Hemodialysis was initiated for persistently elevated valproate, but she expired on Day 3.

**Autopsy Findings:** Acute bilateral pneumonia, acute hemorrhagic pancreatitis with retroperitoneal soft tissue hemorrhage, mild CAD, and moderate hepatic microvesicular steatosis. Antemortem blood valproic acid 970 mg/L. Cause of death: complications of valproic acid intoxication, manner of death: suicide.

**Case 1183.** Chronic lithium ingestion: undoubtedly responsible.

**Scenario/Substances:** A 35 y/o found at home by significant other, lethargic, and responsive with altered mental status.

**Past Medical History:** Bipolar disease, anxiety.

**Physical Exam:** Awake, agitated, shivering, maintaining her airway, pupils equal, and reactive to light, fine-hand tremor, hyperreflexia, and no seizure activity. BP 159/96, HR 80, RR 22, O_2_ sat 100% on room air, T 38.6°C.

**Laboratory Data:** ABG-pH 7.28 / pCO_2_ 20 / pO_2_ 103,

Serum acetaminophen, ethanol and salicylate not detected. Lithium 4.4, ECG: sinus rhythm, QRS 102, QTc 557.

**Clinical Course:** She received three doses of lorazepam IV for agitation. Renal failure and anion gap metabolic acidosis developed. She was intubated for airway protection. NS was given at 2 X maintenance rate. One hour after emergent hemodialysis ended, she became acutely bradycardic (HR 40s) and hypotensive (SBP 70) and required norepinephrine. Repeat electrocardiogram revealed sinus bradycardia with QRS of 110 and QTc 641. Vasopressors were continued with mild improvement in BP. On Day 2, she remained intubated and unresponsive not requiring any sedation. Repeat lithium was 1.4. On Day 4, the patient was declared brain dead. Based on the prognosis, the family opted for institution of comfort measures and she expired on Day 4.

**Autopsy Findings:** Not performed

**Case 1200.** Acute-on-chronic bupropion, diltiazem (extended release), and prednisone ingestion: undoubtedly responsible.

**Scenario/Substances:** A 42 y/o female was found at home with empty bottles of bupropion, diltiazem, and prednisone nearby.

**Past Medical History:** Current medications: zolpidem, clonazepam, and citalopram.

**Laboratory Data:**

Na 145, K 3.5, Cl 111, CO_2_ 21, K 3.4, Glu 593, lactate > 7 mmol/L. ABG-pH 7.27 / pCO_2_ 29.6 / pO_2_ 80.3 / HCO_3_ 13.4 on 3 L nasal cannula; serum acetaminophen and ethanol not detected, UDS positive to amphetamines and benzodiazepines.

**Clinical Course:** In ED, patient was initially lethargic but arousable and able to speak in complete sentences. She eventually became completely unresponsive with dilated pupils and hypotension (BP 60/40) which did not correct with fluid bolus. Patient started on norepinephrine with no response so glucagon 3 mg bolus was given and infusion of 5 mg/h started with BP responding to 83/36. She was also given ondansetron, pantoprazole, lorazepam, and sodium bicarbonate for her acidosis. Day 1 ECG: sinus tachycardia, rate 120, PR 126, QTc 522. Patient had two 10-sec tonic-clonic seizures, was intubated, and developed severe bradycardia and cardiac arrest. A temporary transcutaneous pacemaker was inserted. She became severely hypotensive with mottled skin, and no perfusion, coded again, and resuscitation was unsuccessful. Mouthful of blue and white granules/undissolved pills was discovered when endotracheal tube removed post-expiration.

**Autopsy Findings:** Autopsy demonstrated pill fragments in the mouth, esophagus, stomach, and small intestine along with moderate pulmonary congestion and edema. Postmortem vena cava blood bupropion > 10 mg/L, threo bupropion > 10 mg/L. Liver bupropion 14 mg/kg, threo bupropion 150 mg/kg. Antemortem blood bupropion 1.5 mg/L and threo bupropion 5.6 mg/L, diltiazem detected. Cause of death was bupropion toxicity.

**Case 1268.** Acute amitriptyline and diphenhydramine ingestion: undoubtedly responsible.

**Scenario/Substances:** A 9 m/o male was placed in his car seat to sleep the night and found unresponsive in the morning.

**Past Medical History:** Cystic mass in the lower lobe of his right lung, which was diagnosed in utero.

**Clinical Course:** Pulseless, with evidence of lividity. CPR was initiated, and an intra-osseous line was placed for fluids (25% dextrose) and 1 dose of epinephrine before resuscitation efforts were halted.

**Autopsy Findings:** A cystic mass involved the right lower lobe with microscopic findings suggestive of extra lobar sequestration. He had acute bronchopneumonia consistent with a period of obtunded survival and mild–moderate cerebral edema. Toxicology: amitriptyline 3.5 mg/L heart blood, 46 mg/kg liver), nortriptyline (1.7 mg/L heart blood, 28 mg/kg liver; diphenhydramine 1.9 mg/L heart blood, 8.3 mg/kg liver. These levels were felt to be inconsistent with exploratory ingestion by a 9-month old and not consistent with the initial history. The cause of death was ruled as amitriptyline and diphenhydramine toxicity with the manner of death being homicide.

**Case 1272**. Acute diphenhydramine ingestion: probably responsible.

**Scenario/Substances:** A 12 m/o 10-kg female was found with a bottle of mother's 50 mg diphenhydramine liquid gel caps. Most of the tabs were missing but exact amount unknown. The mother took the child to the closest ED.

**Past Medical History:** Previously healthy, no surgeries, no daily medications.

**Laboratory Data:** pH 7.2, Na 139, K 4.6, CO_2_ 22, BUN 20, Cr 0.1, Glu 99, Ca 9.2, CK 261. Serum acetaminophen and salicylate were not detected.

**Clinical Course:** Upon arrival to the ED, the patient was awake, irritable, and tachycardic. She had a seizure and received multiple doses of midazolam. ECG showed VT at a rate of 213, QRS 160. She was given 2 boluses of 2 mEq/kg sodium bicarbonate and started on an IV infusion. The QRS narrowed to 92, ST elevation was noted in leads II, AVF, V2, V6, and QTc was 420. ECG showed sinus rhythm with PVCs. Seizure activity ceased, the patient was somnolent and intubated for airway protection after vomiting. Post intubation, she developed bradycardia, and PALS protocol was initiated; 10 ml of lipid emulsion (1 mL/kg) plus PALS medications (epinephrine, atropine) were administered. Bradycardia (HR 30–40s) persisted and lipid emulsion was repeated, while PALS was in progress. Resuscitation was unsuccessful, and she was pronounced dead 4 hours after exposure.

**Autopsy Findings:** Not available.

**Case 1288.** Acute diphenhydramine ingestion: undoubtedly responsible.

**Scenario/Substances:** A 43 y/o female took 325 × 25 mg diphenhydramine, spoke to her family at noon, but family was unable to contact her later in the day. EMS arrived to find her pulseless, intubated her, and started CPR.

**Past Medical History:** Diabetes mellitus, breast cancer, CAD, chronic renal disease, hyperlipidemia, COPD, hypertension, allergies, anemia, GERD, arthritis, hypothyroidism, and seizures. She had a long history of depression and, according to her family, was refusing medical treatment. Medications included ergocalciferol and loratadine.

**Physical Exam:** BP 76/42, HR 106, RR 18, left frontal abrasion, fixed and dilated pupils, absent pulses, equal breath sounds, unresponsive, GCS 3, skin warm, dry mucous membranes, absent bowel sounds, no corneal/gag reflex.

**Laboratory Data:** ABG-pH 6.91 / pCO_2_ 68 / pO_2_ 94

Hgb 12.4, WBC 13.6, platelets 282, AST 610, ALT 520, bilirubin 0.5, INR 3.4, lactate 16.8, CK 13,801, troponin 1.89, HCG negative, UDS negative, serum positive for caffeine and diphenhydramine, acetaminophen and salicylate not detected. CxR: right upper lobe atelectasis, CT C-spine: negative, CT head: diffuse cerebral edema

ECG: QRS 122, QTc 477.

**Clinical Course:** In the ED, she was having intermittent loss of pulses. She was given bicarbonate IV push and started on a continuous infusion. Her BP remained low despite maximum epinephrine and vasopressin was added. She was given IV lipid emulsion with transient improvement in her BP and ECG. In the ICU, she developed DIC with epistaxis and oozing blood from puncture sites; Hgb 8.3; Cr 2.2; and troponin 6.8. She was given FFP and was not felt to be a candidate for hypothermia protocol. Early on Day 2, she developed asystole and expired.

**Autopsy Findings:** Diffuse bronchopneumonia, autolysis of the spleen and pancreas, and cerebral edema. Heart blood diphenhydramine 28,000 ng/mL. This level is consistent with levels reported in fatalities. Cause of death: drug overdose with complications. Manner of death was suicide.

**Case 1301.** Acute-on-chronic amantadine ingestion: probably responsible.

**Scenario/Substances:** A 65 y/o female took “a lot of red pills,” was found the next morning unresponsive with shallow respirations. EMS arrived, intubated, and transported the patient. During transport, generalized seizure activity was noted. A review of the patient’s medications led to belief that the patient had overdosed on 100-mg amantadine tablets, but amount was unknown.

**Past Medical History:** Hypothyroidism, chronic pain, reflux, hyperlipidemia, depression. Medications: amantadine, levothyroxine, pravastatin, citalopram, carvedilol, gabapentin, oxybutynin, omeprazole, sertraline, tramadol, vitamin D, and acetaminophen/hydrocodone.

**Laboratory Data:** AST 23, ALT 11, bilirubin 0.2, CK 120,

Serum acetaminophen and salicylates were not detected, UDS positive for cocaine.

**Clinical Course:** In the ED, the patient was placed on ventilator, BP 122/72, HR 68, RR 12 (ventilator). Generalized seizure activity was treated with lorazepam. Initial ECG QRS 128, QTc 540. Her K level was repleted, she was transferred to the ICU, had another seizure, and sodium bicarbonate was given IV and an infusion started at 100 mL/hour. Without sedation, she would grimace in response to sternal rub and gag on endotracheal tube when stimulated. BP 175/71, HR 61, T 36°C. ECG Day 2 normal QRS QTc of 585. Na 132, K 3.0, Cl 94, CO_2_ 28, BUN 14, Cr 1.1, Glu 88. She demonstrated intermittent bursts of VT. Sedation with fentanyl and midazolam infusions was started and then with antibiotics (vancomycin and piperacilln–tazobactam). On Day 2, the patient developed recurrent seizures and was started on with levetiracetam and valproic acid. She also had a period of hypotension that improved with saline bolus and phenylephrine. She had increasing oxygen requirement on the ventilator with FiO_2_ of 80%. Her urine output decreased and urine became dark in color. K 6.0 treated with calcium gluconate, Kayexalate, furosemide and insulin. She developed a widened QRS with bradycardia. Hemodialysis was started on Day 4 for worsening hyperkalemia. On Day 5, she became tachypneic (RR 24) and pH 7.24. Sedation was changed to propofol and reduced on Day 7, but she remained without purposeful movements. Diltiazem was initiated for cardiac ectopy. EEG on Day 10 demonstrated anoxic encephalopathy. Based on the prognosis, the family opted for institution of comfort measures and she expired on Day 11.

**Autopsy Findings**: The ME reviewed the case, but no autopsy or body viewing was performed. Cause of death was undetermined with cocaine abuse as a contributing factor and some consideration to “drug overdose”.

**Case 1307.** Acute-on-chronic methotrexate ingestion: probably responsible.

**Scenario/Substances:** A 82 y/o female received 2.5 mg of methotrexate per day instead of 2.5 mg thrice per week of methotrexate for 1 month at her extended care facility. She was admitted to the hospital with renal failure, mucositis, neutropenia, and infection. The error was discovered, and methotrexate dosing was stopped.

**Past Medical History:** Arthritis, diabetes, hypertension, colon cancer, and chronic kidney infections.

**Laboratory Data:** WBC 0.5, Hgb 9.8, Hct 28.8, RBC 3.22, Platelets 3,000, BUN 86, Cr 4.2 (1 year prior BUN 52, Cr 1.8).

**Clinical Course:** She was initially awake alert, but drowsy and slightly confused after receiving analgesics. HR 111, BP 127/69, RR 19, O_2_ sat 98% on 2L O_2_, T 37°C. She received several units of platelets daily and leucovorin 100 mg/6 h IV. Her urine output was low (60 cc per 8 hrs) on IV furosemide. Based on the prognosis, the family opted for institution of comfort measures on Day 36. By Day 37 she had developed sores all over her body, on her arms, legs and in the area of her perineum which opened up. Her WBC 4.6, platelets 22, BUN 114, Cr 4.2, K + 4.2. On Day 38, she was transferred to hospice and expired.

**Autopsy Findings:** Not done.

**Case 1318.** Chronic nitroprusside parenteral: contributory.

**Scenario/Substances:** A 23 y/o female was admitted to the ICU with acute decompensated heart failure and started on with nitroprusside infusion x 3 days with mild improvement in her condition. On Day 3, she suffered a PEA arrest with return of spontaneous circulation after 15 min of ACLS resuscitation, after which she required multiple vasopressors.

**Past Medical History:** Congestive heart failure, severe dilated cardiomyopathy of unknown cause, methamphetamine abuse.

**Physical Exam:** Lethargic, but arousable to voice, jugular venous pulses elevated to angle of jaw, bibasilar crackles and diminished breath sounds, S3 present, 2/6 systolic murmur at apex, abdominal ascites with distension and positive hepatojugular reflex, diffuse lower extremity edema and anasarca, poor capillary refill with cool fingers/toes. BP 109/69, HR 147, RR 30, O_2_ sat 98% on 3L O_2_, T 38.7°C.

**Laboratory Data:** ABG-pH 7.32 / pCO_2_ 67 / pO_2_ 36, lactate 13.7 mmol/L. UDS negative for amphetamine and methamphetamine. Blood: nitroprusside: 7,170 ng/L, cyanide (pre-treatment) 6.289 mg/L, cyanide (post-treatment) 0.128 mg/L.

**Clinical Course:** Cyanide toxicity considered, cyanide levels sent, and patient treated with 300 mg sodium nitrite and 12.5 g sodium thiosulfate, followed by second dose of 150 mg sodium nitrite and 6.25 g sodium thiosulfate. The patient did not fully recover from the PEA arrest, developed acute renal failure treated with hemodialysis, and required increasing vasopressors to maintain perfusion. Based on the prognosis, the family opted for institution of comfort measures, and she expired on Day 4.

**Autopsy Findings:** Not performed.

**Case 1381.** Unknown, amlodipine/benazepril ingestion: undoubtedly responsible.

**Scenario/Substances:** A 51-year-old female ingested unknown quantities of amlodipine/benazepril 10/20 and presented to the ED complaining of blurred vision.

**Physical Exam:** BP 122/100, HR 84, RR 14, O_2_ sat 95% on room air.

**Laboratory Data:** Ca 9.4, Mg 2.0, AST 13, ALT 18, PT 14.1,

INR 1.13, serum acetaminophen, ethanol, and salicylate not detected.

**Clinical Course:** Shortly after ED arrival, the patient became hypotensive to BP 51/38, HR 84. Calcium gluconate, glucagon, norepinephrine, bicarbonate, and atropine were given. The patient remained awake and oriented at 6 h. At Hour 7, HR 73, BP 93/59, RR 23, O_2_ sat 96% on 2L O_2_. A high-dose insulin infusion was initiated at 60 U/h, with supplemental glucose. Dobutamine and then vasopressin were administered. Attempts to wean insulin were followed by sudden hypotension. Insulin was increased to 2U/kg/h with BP 93 systolic. At Hour 52 the patient suffered a cardiac arrest, was resuscitated and had multiple episodes of bradycardia and repeated cardiac arrests. She expired on Day 3.

**Autopsy Findings:** The cause of death was polysubstance overdose. The manner was suicide.

**Case 1407**. Acute verapamil ingestion: undoubtedly responsible.

**Scenario/Substances:** A 59 y/o male was brought to the ED after his family noticed an altered level of consciousness. EMS found him hypotensive and bradycardic. They applied an external pacemaker and transported him to the ED.

**Past Medical History:** Hypertension, hyperlipidemia, migraines, and benign prostatic hypertrophy. Medications included verapamil, sumatriptan, lisinopril, topiramate, tamsulosin, terazosin, methocarbamol, pravastatin, and aspirin.

**Physical Exam:** On arrival to the ED, he was intubated for impending respiratory failure. BP 40's, HR 30's. He had a brief period of cardiac arrest which responded to CPR and epinephrine. Dopamine infusion was started, and he was admitted to the ICU for a suspected verapamil overdose.

**Laboratory Data:** ABG-pH 7.12 / pCO_2_ 48 / pO_2_ 113,

Hgb 10.9, WBC 16.8, platelets 214, lactate 6.8 mmol/dL,

UDS positive for methamphetamines, MDMA, amphetamines, and phencyclidine. Initial ECG showed complete heart block with intraventricular escape rhythm.

**Clinical Course:** In the ICU, a transvenous pacemaker was placed. Several doses of IV Ca were given, and broad-spectrum antibiotics were started. Dopamine, epinephrine, vasopressin, and sodium bicarbonate infusions were given. The poison control center recommended continued use of IV calcium, and starting high-dose insulin plus dextrose infusions. Despite normalization of his BP and HR, he remained unresponsive. Head CT showed cerebral edema most likely secondary to anoxic encephalopathy, and scan showed no brain blood flow. Neurology was consulted, and he was declared brain dead on Day 4

**Autopsy Findings:** Hospital blood was positive for caffeine, lidocaine, midazolam, topiramate, verapamil, phenylpropanolamine, amphetamine, and methamphetamine. Verapamil 1,500 ng/mL, topiramate 3.4 mcg/m, midazolam 15 ng/mL, phenylpropanolamine 22 ng/mL, amphetamine 250 ng/mL, methamphetamine 850 ng/mL. Cause of death: medication overdose along with use of controlled substance. From the laboratory results and the clinical course, it is most likely that he died as a result of acute verapamil overdose.

**Case 1411.** Acute-on-chronic diltiazem ingestion: undoubtedly responsible.

**Scenario/Substances:** A 60 y/o male ingested ∼90 diltiazem 180 mg extended release tablets, had a seizure at home (∼1 min) witnessed by family. He denied co-ingestants. An empty bottle found at the scene had been filled (90 tablets) earlier that day.

**Past Medical History:** Depression, anxiety, previous suicide attempts, diabetes, hypertension, liver cancer, COPD, diabetic neuropathy, degenerative joint disease, hyperlipidemia, multiple falls, myofascial pain dysfunction syndrome, radiculopathy to bilateral lower extremities after laminectomy, shoulder pain, transient ischemic attack, GERD, lumbago with chronic back pain, BPH, urinary retention.

**Physical Exam:** He was postictal for 10–15 min, waking minimally able to verbalize complaint of back pain, was tremulous and hypotensive. BP 70/40, HR 67, RR 18, O_2_ sat 94% on 4 L of oxygen.

**Laboratory Data**: WBC 11.5. AST 8, ALT 26. calcium 8.3,

Mg 2.1, troponin 0.01, lactic acid 7.0 mmol/l, albumin 3.3, Serum acetaminophen and salicylate not detected, UDS positive for benzodiazepines.

**Clinical Course:** Pupils were 3 mm, and he had jerky movements. He was treated with calcium gluconate, and glucagon with no response and was given lorazepam for seizures. IV fluids were started with no improvement in hypotension; norepinephrine was added with little response. ECG showed AV dissociation with accelerated junctional rhythm, QRS 96, QTc 442. He had a tonic-clonic seizure that resolved with a dose of lorazepam. After the seizure, he was alert with slurred speech. BP 95/37 (rapidly falling to 62/43), HR 64, RR 14, and O_2_ sat 93% on 4 L of oxygen. Activated charcoal was given Hour 3. He complained only of generalized weakness. Head CT was negative for acute pathology. He was treated with D50W with high-dose insulin, but it was initiated at the time that a bradysystolic cardiac arrest occurred. Hour 5.5. CPR with epinephrine, atropine was unsuccessful.

**Autopsy Findings:** Urine positive for caffeine, benzodiazepines, gabapentin, lidocaine, and nicotine. Urine oxycodone 0.17 mg/L, oxymorphone 0.022 mg/L. Urine oxycodone and oxymorphone concentrations consistent with normal use. Antemortem blood diltiazem 8.5 mg/L. ME listed the probable cause of death as diltiazem toxicity with contribution from hypertension, TIA/Stroke, COPD and diabetes, with the manner being suicide.

**Case 1501.** Acute sodium bicarbonate ingestion: undoubtedly responsible.

**Scenario/Substances: A** 33 y/o male ingested large quantities of sodium bicarbonate to cleanse his system prior to drug testing. Found unconscious in bed by his significant other and transported to the ED.

**Laboratory Data:** Initial labs: ABG-pH 7.56 / pCO_2_ 51.8 / pO_2_ 83 / HCO_3_ 44.9 / BE 20.1 on FiO_2_ 70%. Ca 11.6, Hgb 19.3, WBC 19.3,

anion gap 53, Mg 4.3, ammonia 39, serum acetaminophen, ethanol and salicylate not detected, UDS negative. Hour 6: Na 167, CK 1,602. Hour 12: Na 166, K 3.0, Glu 194, Cr 2.36, Cl, 119, CO_2_ 37, Ca 7.9. Hour 24: Na 164, K 3.4, Cl, 131, Glu 138, Cr 2.31, Ca 8.0, CK 2,979.

**Clinical Course:** In the ED seizures developed, given levetiracetam and phenytoin and intubated. Initial ECG QT/QT_C_ 300/463. BP 80/43, HR 104, RR 44, O_2_ sat 94%. He received etomidate, lidocaine, lorazepam, propofol, vecuronium, and D50W. CT showed diffuse extensive subarachnoid hemorrhage, mild, dilation of temporal horns of the lateral ventricles. Hemorrhage was thought to be owing to massive osmotic shift as a result of the sodium load. Patient was transferred to a tertiary care hospital where he received pentobarbital but was still having seizures per EEG. Received piperacillin and tazobactam for suspected aspiration. HR 130, BP 117/67, T (bladder) 39.3°C. Based on the prognosis, the family opted for institution of comfort measures and he expired on Day 3.

**Autopsy Findings:** Not available.

**Case 1546**. Unknown, carisoprodol and meloxicam ingestion: undoubtedly responsible.

**Scenario/Substances:** A 41 y/o female took unknown amounts of meloxicam and carisoprodol, was found unresponsive 1.5 h after talking to a friend. She was found to be in cardiac arrest, and transported to the ED.

**Past Medical History:** Non-insulin dependent diabetes mellitus, systemic lupus erythematosus, hypertension, bipolar disorder, fibromyalgia, and depression.

**Laboratory Data:** K 2.9, Mg 1.5, UDS positive for tricyclic antidepressants, serum acetaminophen was not detected.

**Clinical Course:** In the ED, she was intubated, and naloxone was given with no response. She was also given flumazenil, epinephrine, and dopamine. ECG showed sinus tachycardia with depression in the lateral leads. Systolic BP 160, HR 120, and she had a metabolic acidosis. She was transferred to a tertiary care hospital and admitted to the ICU. She was on norepinephrine and phenylephrine, and her urine output was characterized as “good”. She received potassium replacement for hypokalemia, was placed on post cardiac arrest cooling protocol and started on propofol with BP 97/72, HR 70, T 32.8°C. On Day 2, she was rewarmed and sedation was stopped HR 102, BP 110/65. She had no neurological activity, consistent with anoxic brain injury. Based on the prognosis, the family opted for institution of comfort measures and she expired on Day 3

**Autopsy Findings:** Ischemic brain injury, bronchocentric pneumonia and pulmonary edema. Urine from hospital admission positive for amitriptyline, diphenhydramine, nicotine, nortriptyline. Antemortem (Day 1) serum: 7-aminoclonazepam, < 0.020 mg/L; carisoprodol, < 16 mg/L; and meprobamate, 46 mg/L. Antemortem (Day 1, sample 1) blood: carisoprodol 19 mg/L, diphenhydramine < 0.25 mg/L, and meprobamate 35 mg/L. Antemortem (Day 1, sample 2) blood carisoprodol 6.7 mg/L, lamotrigine < 4.0 mg/L, nortriptyline < 0.25 mg/L, and meprobamate 43 mg/L, amitriptyline not detected. The ME-determined cause of death was hypoxic ischemic brain injury owing to meprobamate toxicity, more likely the death was due to carisoprodol toxicity with contribution from its metabolite, meprobamate since the parent compound carisoprodol was found on multiple samples as well as being initially suspect from the history.

**Case 1643.** Acute pentobarbital/phenytoin, embutramide/ mebezonium/tetracaine parenteral: undoubtedly responsible.

**Scenario/Substances:** A 59 y/o female veterinarian was found unresponsive in her veterinary clinic. She appeared to have injected herself with either pentobarbital/phenytoin or embutramide/mebezonium iodide/tetracaine solution as a suicide attempt. She was intubated in the field for respiratory arrest. She received naloxone prior to arrival at the ED with no response.

**Past Medical History:** Depression, dementia.

**Physical Exam:** Unresponsive on ventilator, BP 90/60, HR 90.

**Laboratory Data:** ABG-pH 7.3 / pCO_2_ 42 / pO_2_ 200, Na 134, K 2.9, Cl, 102, CO_2_ 24, BUN 20, Cr 1; phenytoin 6 mcg/mL, phenobarbital 4 mcg/mL, valproate 33.6 mcg/mL; serum acetaminophen and salicylate not detected.

**Clinical Course:** In the ED, she received flumazenil without response. She remained hypotensive and bradycardic, and received IV fluids and multiple vasopressors without measurable effect. She had a cardiac arrest and died on Day 3.

**Autopsy Findings:** Postmortem toxicological tests included hospital blood levels of lorazepam 18.9 ng/mL, valproic acid 19.5 mcg/mL, mirtazapine 9.7 ng/mL, caffeine-positive, pentobarbital 74.3 mcg/mL, venlafaxine 149 ng/mL, norvenlafaxine 503 ng/mL, and urine pentobarbital > 10, 000 ng/mL. Cause of death was reported as pentobarbital toxicity, and manner of death is reported as suicide.

**Case 1671.** Acute hallucinogenic amphetamine ingestion: undoubtedly responsible.

**Scenario:** A 17 y/o male snorting “bath salts” at a party experienced seizure activity, and was found unconscious. EMS-administered benzodiazepines, paralyzed, endotracheally intubated, and transported the patient to the ED. “Bath salts” were found at the party by law enforcement

**Clinical Course:** The patient had a cardiac arrest upon arrival to the ED. His pupils remained dilated and non-reactive. Upon return of spontaneous circulation, his BP was 70/40 on vasopressors, HR 75, T 37°C, RR 24 (ventilator), O_2_ sat 89% on 100% FIO_2_. ABG-pH 7.12 / pCO_2_ 48 / pO_2_ 53 / HCO_3_ 15.3, K 8.1, Hgb 5.3, Hct 15.8, CK 689, troponin I 2.3, serum acetaminophen, ethanol, and salicylate not detected. The UDS was negative for amphetamines. The patient was admitted to the ICU, place on a sodium bicarbonate drip, epinephrine, norepinephrine, phenylephrine and amiodarone. He developed DIC, and was given FFP and cryoprecipitate. Lipid emulsion therapy was given for persistent hemodynamic instability. He expired on Day 1.

**Autopsy Findings:** Evidence of DIC, diffuse organ failure, massive pulmonary edema, bilateral pleural effusions, small subdural hematoma, and anoxic brain abnormalities. Cause of death: 2, 5-dimethoxy-N-(2-methoxybenzl) phenethylamine derivative (NBOMe) toxicity. Analysis of powder residue on the patient confirmed the substance. The drug was not detected in the patient’s Hour 12 blood. amphetamines found in his blood and urine on medical examiner screens were thought to be NBOMe metabolites. Manner of death was accidental.

**Case 1687.** Acute methylenedioxy-methamphetamine (MDMA) ingestion: undoubtedly responsible.

**Scenario/Substances:** A 19 y/o female collapsed at the nightclub after ingesting “Molly”. EMS on scene noted apnea and a weak pulse, rescue breathing was performed, and she was transported to the ED.

**Physical Exam:** The patient was seizing and unresponsive.

**Laboratory Data:** Na 155, K 6.5, Cl 110, anion gap 21, BUN 14, Cr 1.99, Ca 8.0, Mg 2.5, Hgb 12, WBC 17.0, platelets 296, PT 18, INR 1.45, PTT 31.7, CK 523, CKMB 6.0, troponin 0.3. Acetaminophen and salicylate were not detected, UDS was negative. INR 10 and amylase > 1000 U/L 12 h later.

**Clinical Course:** In the ED, the patient was diaphoretic, T 39.7°C (rectal), pupils dilated, minimally responsive; was intubated, sedated with midazolam, ventilated. Phenytoin was given for “seizure-like activity”. BP 70/30. IV fluids with bicarbonate were administered with little improvement in BP so vasopressor support was initiated and she was admitted to the ICU. After 12 h, despite the use of maximal vasopressor support, her BP was 80/27, HR 100s, and urine output was minimal. Blood products and N-acetylcysteine were administered. The patient suffered a cardiac arrest and expired 17 h after admission to the hospital.

**Autopsy Findings:** Cause of death was 3, 4-methylenedoxymethamphetamine (MDMA) intoxication.

**Case 1691.** Acute hallucinogenic amphetamine (methylone) ingestion: undoubtedly responsible.

**Scenario/Substances:** A 20 y/o male and was found down while attending a concert. When seen previously by his friends, he had exhibited an increased RR.

**Past Medical History:** Good general health.

**Physical Exam:** Obtunded, GCS 3, HR 140, T normal on presentation.

**Laboratory Data:** Na 139, later 146, K 7.1, later 2.7, Cr 2.5, pH 7.02, CO_2_ 15, platelets 36, PT > 120 s, PTT > 180 s, AST 494, ALT 164, Alk phos 90, bilirubin 2.0, CK initially normal, later 8,900. lactate 4 (decreased from earlier peak)

**Clinical Course:** He was intubated for airway protection, T 41.7°C, later 38.9°C within 1 hour of treatment. The patient received N-acetylcysteine IV for fulminant hepatic failure. The patient developed bradycardic/PEA arrest 2–3 times during the initial phases of treatment which responded to ACLS. Despite aggressive supportive care, the patient experienced an uncontrollable hemorrhage on Day 1 secondary to DIC and subsequently experienced a cardiac arrest from which he could not be resuscitated.

**Autopsy Findings:** Cause of death was hyperthermia due to methylone intoxication and environmental exposure. Heart blood contained 0.71 mg/L methylone and cannabinoids were detected.

**Case 1724.** Hallucinogenic amphetamine ingestion: undoubtedly responsible.

**Scenario/Substances:** A 23 y/o male found obtunded after a reported ingestion of “Molly” while at an electronic music concert was brought to the ED by EMS.

**Physical Exam:** Obese, diaphoretic, GCS 3, pupils dilated and reactive, lower extremity rigidity. Initial BP 88/58, HR 160, T 42.7°C, respirations agonal.

**Laboratory Data:** ABG-pH 7.15 / pCO_2_ 48 / pO_2_ 141 / HCO_3_ 16, O_2_ sat 98%, Hgb 15, Hct 44, WBC 16, lactate 10,

anion gap 21, troponin 0.27, UDS negative for cocaine, barbiturates, benzodiazepines, opioids, phencyclidine, and cannabinoids.

**Clinical Course:** In the ED, the patient was intubated via rapid sequence intubation with etomidate, rocuronium, and midazolam. T was reduced from 42.7–39.4°C over ∼30 min with a cooling blanket and 6L cold NS. He remained hypotensive despite fluid resuscitation, was transferred to the ICU where he was found to pulseless. Resuscitative efforts lasting 50 min were unsuccessful.

**Autopsy Findings:** Final diagnoses: Acute intoxication with methylenedioxy-methamphetamine and methylone with hyperthermia, cardiac hypertrophy with left ventricular hypertrophy, aortic atherosclerosis, slight, obesity. Cause of death: Acute intoxication by the combined effects of methylenedioxy-methamphetamine and methylone with hyperthermia. MDMA 2.6 mg/L, methyleneoxyamphetamine 0.12 mg/L, methylone 0.22 mg/L. Manner of death: Accident (substance abuse).

**Case 1783**. Cocaine and levamisole exposure: probably responsible.

**Scenario/Substances:** A 28 y/o white female was found unresponsive, lying in her feces and urine at home, EMS was summoned by her significant other. When EMS arrived, they found the patient not breathing and pulseless. They started bagging her with a bag valve mask, performed cardio pulmonary resuscitation and within a minute were able to feel a pulse. Because of emaciation and necrotic limbs, the emergency medicine team did not attempt to place IV access or an intraosseous needle before transport.

**Past Medical History:** Chronic IV drug use including heroin, cocaine, and methamphetamine. She presented several weeks prior to another hospital with skin lesions strongly suggestive of vasculitis.

**Laboratory Data:**

anion gap measured 24, Ca 7.9, Ca (ionized) 1.22, Phos 14, Mg 3.2, Tprot 5, albumin 1.2, bilirubin 0.6, bili (direct) 0.5, Alk phos 122, AST 60, ALT 13, WBC 14.3, Hgb 4.4, platelets 51, urine pregnancy test negative, serum ethanol was not detected, UDS positive for cocaine and opiates and negative for amphetamines, benzodiazepines, and methadone.

**Clinical Course:** In the ED BP 46/24, HR 46, RR 22, GCS 3. Emaciated and poorly nourished, multiple gangrenous wounds and a decubitus ulcer. General surgery and intensive care teams were consulted to determine if the patient was treatable. The decision was made to let the patient expire. The patient died in the ICU ∼4 h after arriving at the ED.

**Autopsy Findings:** The antemortem and postmortem blood levamisole 0.28 mcg/mL. Postmortem blood benzoylecgonine 3,300 ng/mL. Death was due to complications of sepsis, complicating gangrenous wounds, most likely related to levamisole associated vasculitis. The manner of death was ruled natural, as it was the sequelae of chronic drug use.

**Case 1836.** Acute methamphetamine ingestion: undoubtedly responsible.

**Scenario/Substances:** A 32 y/o male in police custody developed a sympathomimetic toxidrome and became unresponsive after presumably body stuffing methamphetamine.

**Past Medical History:** Polysubstance abuse: amphetamines, alcohol, and marijuana.

**Physical Exam:** BP 109/87, HR 143, RR 29, T 38.7°C.

Obtunded, diaphoretic, pale, no signs of trauma; pupils 8 mm, symmetric, and reactive; lungs clear; abdomen soft, non-distended, bowel sounds present; GCS 4, not responsive to painful stimuli;

**Laboratory Data:** ABG-pH 7.24 / pCO_2_ 189 / pO_2_ 43.6 / HCO_3_ 18.5, WBC 11.9, K 4.9, CO2 15, BUN 29, Cr 2.2, lactate 4.0 mmol/L, CK 1029. Serum acetaminophen and salicylate were not detected. UDS positive for amphetamine, methamphetamine and THC metabolite. UA showed myoglobinuria.

**Clinical Course:** In the ED, he was intubated with a neuromuscular blocker, received benzodiazepines for sedation, and activated charcoal. T increased to T 43.3°C requiring placement of IV cooling device: norepinephrine was started for hypotension, sodium bicarbonate was given for acidosis, and calcium was replaced. He was admitted to the ICU with hypotension unresponsive to IV fluids and multiple vasopressors. He developed anuric renal failure, DIC and bleeding per rectum. He was given steroids, cryoprecipitate and FFP. Due to hemodynamic instability, he was considered to not be a candidate for hemodialysis or CVVH. Whole bowel irrigation was initiated: He developed shock liver with abdominal distension with increasing lactate, thought to be due to bowel infarction. Based on the prognosis, comfort measures were instituted and he expired on Day 1.

**Autopsy Findings:** Bilateral pulmonary edema; upper GI bleeding with 700 cc of coffee ground material in the stomach; hemorrhages of the pericardium, omentum, and stomach erosions; endocardial hemorrhage. Cause of death: acute methamphetamine toxicity.

**Case 2062.** Chronic dimethylamylamine ingestion: contributory.

**Scenario/Substances:** A 59 y/o female was found on the floor by her family. Family found an empty bottle of dietary supplement at her home.

**Past Medical History:** Obesity, depression, COPD and chronic pain. Medications: Benzodiazepines, acetaminophen/hydrocodone, and dietary supplement containing 1,3-dimethylamylamine (DMAA). She was evaluated by her primary care physician over the last 2 weeks for jaundice and elevated liver function tests with negative viral hepatitis panel and undetectable acetaminophen level.

**Physical Exam:** Obese, jaundiced, nonverbal but able to opens eyes to verbal stimulation. No abdominal distention. BP 157/75, HR 88, RR 10, O_2_ sat 96% room air.

**Laboratory Data:** Bilirubin 39.7, AST 933, ALT 959, Alk phos 186, INR 2.8, PTT 48, serum acetaminophen 15 mcg/mL, serum salicylate not detected, hepatitis A negative, hepatitis C negative, previous hepatitis B infection, anti-mitochondrial antibody and ANA negative, HIV negative, abdomen CT negative for mass lesions.

**Clinical Course:** Patient was found to have fulminant hepatic failure upon arrival to the ED. N-Acetylcysteine was started and continued for the duration of hospitalization. Further evaluation for cause of hepatic failure was unrevealing. Her mental status deteriorated, and she required endotracheal intubation. She was transferred to a tertiary care hospital. Despite aggressive management, there was no improvement of her hepatic synthetic function. She was unresponsive without sedation and did not qualify for liver transplantation. Based on the prognosis, comfort measures were instituted and she expired.

**Autopsy Findings:** Extensive liver necrosis (> 95%) and cholestasis. Cause of death was fulminant hepatic failure as a result of of dietary supplement containing 1,3-dimethylamylamine (DMAA).

**Case 2080.** Acute cocaine ingestion: undoubtedly responsible.

**Scenario/Substances:** A male in his 20’s was arrested by police for a possible drug deal. The patient swallowed 2 baggies cocaine during the arrest and shortly thereafter suffered a cardiac arrest in the field. The patient was reported to have had a seizure in his vehicle prior to EMS arrival. EMS found the patient in cardiac arrest, intubated, began CPR, and transported him to the ED

**Past Medical History:** Unknown

**Laboratory Data:** ABG-pH 6.46 / pCO_2_ 81 / pO_2_ 198,

lactate18 mmol/L, Ca 7.0, Hgb 8.4, platelets 98, INR = 2.51, UDS positive cocaine, serum acetaminophen, ethanol and salicylate not detected, ECG showed a LBBB.

**Clinical Course:** In the ED, normocephalic, atraumatic, pupils fixed and dilated, no pulse, no BP. Pulses returned transiently for a brief time on arrival. He received naloxone, epinephrine, and sodium bicarbonate, and was placed on a ventilator. The patient developed a PEA arrest that progressed to asystole. Despite aggressive supportive care, he was unable to be resuscitated and was declared dead.

**Autopsy Findings:** Autopsy report confirmed cause of death as massive cocaine toxicity. Patient also had THC in his system but was not attributed to cause of death. Serum cocaine at time of death was 2,900 ng/mL and benzoylecgonine 2,700 ng/mL.

### Abbreviations and Normal ranges for Abstracts

Disclaimer—all laboratories are different and provide their own normal ranges. Units and normal ranges are provided here for general guidance only. These values were taken from Harrison's (11), Goldfrank (12), or Dart (13)

Serum electrolyte summary table

serum electrolytes have units of mmol/L = mEq/L

∼ = approximately

ABG-pH/pCO2/pO2/HCO3/BE

ABG = arterial blood gases

ABG-pCO2 = partial pressure of carbon dioxide [38–42]

ABG-pH = hydrogen ion concentration [7.38–7.42]

ABG-pO2 = partial pressure of oxygen [90–100]

Base Excess = [− 2 to + 2 mmol/L]

ACLS = advanced cardiac life support, protocol for the provision of cardiac resuscitation

ADHD = attention deficit hyperactivity disorder

AF = atrial fibrillation

AICD = automatic implanted cardio defibrillator

Alk phos = alkaline phosphatase [13–100] U/L

ALT = Alanine aminotransferase [7–41] U/L = (SGPT)

AMA = against medical advice

Ammonia = [25–80] mcg/dL = [15–47] mcmol/L

amp = ampoule

APLS = advanced pediatric life support, protocol for the provision of cardiac resuscitation

ARDS = acute respiratory distress syndrome

AST = Aspartate aminotransferase [12–38] U/L = (SGOT)

AVblock = atrioventricular block

BAL —British anti-Lewisite

BE = base excess, mmol/L

Bicarbonate = [22–26] mmol/L

bili (direct) = direct bilirubin [0.1, 0.4] mg/dL

bili (indirect) = indirect bilirubin [0.2, 0.9] mg/dL

Bilirubin = total [0.3–1.3] mg/dL, direct [0.1, 0.4] mg/dL, indirect [0.2, 0.9] mg/dL

BLQ = below the limit of quantitation

BMI = body mass index

BP = Blood Pressure, systolic/diastolic, (Torr)

BPH = benign prostatic hypertrophy

BUN = see Urea nitrogen

C = degrees Centigrade

Ca (ionized) = ionized calcium, [4.5–5.6] mg/dL

Ca = calcium, [8.7–10.2] mg/dL

CABG = coronary artery bypass graft

CAD = coronary artery disease

Carbon Dioxide = CO2 [22–26] mmol/L

CIWA = Clinical Institute Withdrawal Assessment for Alcohol

CK = creatinine kinase (CPK), total: [39–238] U/L females, [51–294] U/L males

CKMB = MB fraction of CK [0.0–5.5 mcg/L = 0.0–5.5 ng/mL] Fraction of total CK activity [0–0.04 = 0–4.0%]

Cl = chloride [102–109] mmol/L

CNS = central nervous system

CO2 = carbon dioxide serum or plasma [22–26]mmol/L

COHb = carboxyhemoglobin

COPD = chronic obstructive pulmonary disease

CPR = cardiopulmonary resuscitation

Cr = creatinine [0.5–0.9] mg/dL females, [0.6–1.2] males,

CRRT = continuous renal replacement therapy

CSF = cerebrospinal fluid

CT = computed tomography (CAT scan)

CVA = cerebrovascular accident

CVVHD = continuous venovenous hemodiafiltration

CxR = chest radiograph, chest X-ray

D10W = 10% dextrose in water

D50W = 50% dextrose in water

D5NS = 5% dextrose in normal saline

D5W = 5% dextrose in water

Day = when capitalized, Day = hospital day, that is, days since admission

DIC = disseminated intravascular coagulation

Dx = diagnosis

ECG = electrocardiogram (EKG), leads = I, II, III, aVR, aVL, aVF, V1, V2, V3, V4, V5, V6

ECMO = extracorporeal membrane oxygenation

ED = emergency department, in these abstracts refers to the initial health care facility

EDDP = principal methadone metabolite, 2-ethylidene-1,5-dimethyl-3,3-diphenylpyrrolidine

EEG = electroencephalogram

EF = ejection fraction

ELISA = enzyme-linked immunosorbent assay

EMS = emergency medical services, paramedics, the first responders

ER = extended release (sustained release)

FFP = fresh frozen plasma

FiO2 = fraction of inspired oxygen

g = grams

g/dL = grams per deciliter

GCS = Glasgow Coma Score, ranges from 3 to 15

GERD = gastroesophageal reflux disease

GI = gastrointestinal

Glu = glucose, fasting [75–110] mg/dL

h = hours

HCF = health care facility

HCG = human chorionic gonadotropin test for pregnancy

HCO3 = bicarbonate

HCP = health care provider

Hct = hematocrit [35.4–44.4] females, [38.8–46.4]% males

Hgb = hemoglobin [12.0–15.8] g/dL females, [13.3–16.2] g/dL males

HIV = human immunodeficiency virus

Hour = when capitalized, Hour = hours since admission

HR = HR, beats per min

ICP = intracranial pressure

ICU = intensive care unit

IgE = immunoglobulin E

IM = intramuscular

INR = international normalized ratio (PT to control) [0.8–1-2]

IU/L = international units per Liter

IV = intravenous

K = potassium, [3.5–5] mmol/L

kg = kilogram

L = Liter

Lactate = lactic acid [4.5–14.4] mg/dL arterial, [4.5–19.8] mg/dL venous

LBBB = left bundle branch block on ECG

Leukocyte count = white blood count [3.54–9.06] 103/mm3

m/o = months old

MAP = mean arterial pressure

mcg/dL = micrograms per deciliter

mcg/L = micrograms per Liter

mcg/min = micrograms per minute

mcg/mL = micrograms per milliliter

mcmol/L = micromoles per liter

MDA = 3,4-methylenedioxyamphetamine

MDMA = methylenedioxymethamphetamine (ecstasy, molly)

ME = medical examiner

mEq = milliequivalents

mEq/L = milliequivalents per liter

Mg = magnesium [1.5–2.3] mg/dL

mg = milligrams

mg/dL = milligrams per deciliter

mg/kg = milligrams per kilogram

mg/L = milligrams per liter

min = minutes

ml = milliliter

mmol/L = millimoles per liter

mosm/kg = milliosmoles per kilogram

mosm/L = milliosmoles per liter

MRI = magnetic resonance imaging

ms = milliseconds

Narrative Headers:

Scenario/substances: concise narrative of EMS and pre-HCF events

Past medical history: available relevant past medical history

Physical examination: initial physical examination if available

Laboratory data: initial results, give units except for units given in abbreviations

Clinical course: concise narrative of HCF & beyond with outcome

Autopsy findings: = medical examiner and/or autopsy results

NG = nasogastric

ng/mL = nanograms per milliliter

not detected = analyte below the level of quantitation, negative

NPO = nil per os, nothing by mouth

NS = normal saline

O2 sat = oxygen percent saturation [94–100]% at sea level

OR = operating room

Osm = osmole

PALS = pediatric advanced life support

PC = poison center (= PCC, or poison control center)

PCC = prothrombin complex concentrate

PCP —primary care provider

PEA = pulseless electrical activity

PEEP = positive end expiratory pressure

PICU = pediatric intensive care unit

Platelets = platelet count [150–400] x109/L

PO = per os (“by mouth” in Latin)

Potassium = [3.5–5] mmol/L

ppm = parts per million

PR = P-R interval [120–200] msec on the ECG

prn = as needed

PT = prothrombin time, INR is preferred, but PT may be used if INR is not available

PTA = Prior to admission

PTT = partial thromboplastin time [26.3–39.4] sec

PVC = prematureventricular contraction

QRS = ECG QRS complex duration [60–100] msec

QT = Q to T interval on the ECG waveform, varies with HR

QTc = QT interval corrected for HR, usually QTcB = QT/RR½ (Bazett correction) 1–15 y-o [< 440] msec, adult male [< 430] msec, adult female [< 450] msec

RBBB = right bundle branch block on ECG

RBC = red blood cell(s)

RR = respiratory rate, breaths per minute

s/p = status post

sec = seconds

SL = sublingual

SVT = supraventricular tachycardia

Synthetic stimulant = one or more of the products (6-APB, bath salts, plant food, Bliss, Ivory Wave, Purple Wave, Vanilla Sky, et al.) or chemicals (3,4 methylenedioxypyrovalerone [MDPV], 6-(2-aminopropyl)benzofuran [6-APB], butylone, desoxypipradrol [2-DPMP], ethylone, flephedrone, naphyrone, mephedrone, methylenedioxypyrovalerone, methylone, methcathinone, et al)

T (oral) = temperature (oral) [36.4, 37.2]°C

T (rectal) = temperature (rectal) [36.4, 37.2]°C

T (tympanic) = temperature (tympanic) [36.4, 37.2]°C

t-bili = total bilirubin

THC = tetrahydrocannabinol

THC homolog = one or more of the products (Blaze, Dawn, herbal incense, K2, Red X, spice, et al) or chemicals (cannabicyclohexanol, CP-47,497, JWH-018, JWH-073, JWH-200, et al)

TPN = total parenteral nutrition

Tprot = total protein

Troponin I = normal range [0–0.08] ng/mL, cut-off for MI > 0.04 ng/mL

U = units

U/dL = units per deciliter

U/L = units per liter

U/mL = units per milliliter

UA = urinalysis

UDS = urine drug screen

Urea nitrogen (BUN) = [6–17] mg/dL

VBG = venous blood gases

VF = ventricular fibrillation

VT = ventricular tachycardia

WBC = white blood count, see leukocyte count

WNL —within normal limits

y/o = years old
